# Enantioselective Pictet–Spengler-Type Reaction via a Helically Chiral Anion as an Access to 4,5-Dihydropyrrolo[1,2‑*a*]quinoxaline Scaffolds

**DOI:** 10.1021/acs.joc.5c00638

**Published:** 2025-07-01

**Authors:** Martin Nigríni, Filip Uhlík, Ivana Císařová, Jan Veselý

**Affiliations:** † Department of Organic Chemistry, Faculty of Science, 37740 Charles University, Hlavova 2030, 128 43 Prague, Czech Republic; ‡ Department of Physical and Macromolecular Chemistry, Faculty of Science, Charles University, Hlavova 2030, 128 43 Prague, Czech Republic; § Department of Inorganic Chemistry, Faculty of Science, Charles University, Hlavova 2030, 128 43 Prague, Czech Republic

## Abstract

We report an enantioselective Pictet–Spengler-type reaction enabled by a cost-effective and readily available helically chiral cyclopentadiene (PCCP) catalyst. This methodology, conducted under mild reaction conditions, facilitates the synthesis of a novel class of chiral 4,5-dihydropyrrolo­[1,2-*a*]­quinoxalines (DHPQs) characterized by enhanced resistance to aromatization due to intramolecular hydrogen bonding. Additionally, the protocol exhibits broad substrate compatibility and demonstrates significant synthetic versatility.

## Graphical abstract

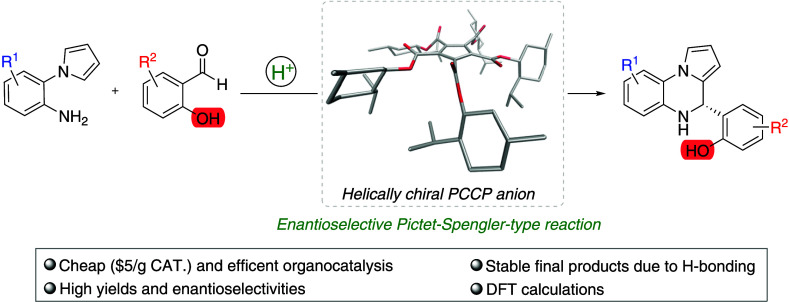

## Introduction

Asymmetric synthesis plays a pivotal role in the construction of complex heterocyclic compounds, which are crucial in the development of bioactive molecules and pharmaceutical agents. Dihydropyrrolo­[1,2-*a*]­quinoxalines (DHPQs) are of particular interest due to their diverse biological activities. Racemic DHPQ derivatives can exhibit various pharmacological effects, including potential applications such as agricultural fungicides (Figure 1, **I**),^1^ anti-HIV agents (**II**),^2^ anticancer compounds (**III**),^3^ estrogen receptor modulators (**IV**),^4^ Nogo receptor modulators (**V**),^5^ cannabinoid type 1 receptor (CB1R) antagonists (**VI**),^6^ and cystic fibrosis transmembrane conductance regulator (CFTR) chloride channel inhibitors (**VII**).^7^ Given their broad therapeutic potential, the development of efficient and selective synthetic methods for the DHPQ core remains highly desirable.

**Figure 1:**
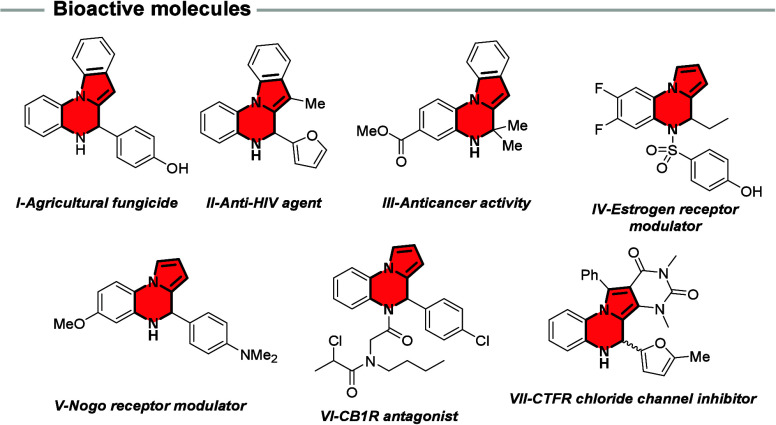
Representative biologically active racemic dihydropyrrolo­[1,2-*a*]­quinoxalines (DHPQs).

To date, various DHPQ derivatives were prepared as racemates using the Brønsted,^8^ Lewis acid,^9^ or iodine^10^ catalyzed Pictet–Spengler-type reaction. Nevertheless, only a few asymmetric methods were disclosed for preparing DHPQs derivatives.^11–16^ A notable advancement in the area of asymmetric Lewis acid catalysis was achieved by the Tian group,^11^ who reported the synthesis of 4-substituted DHPQs with good enantioselectivity using a chiral boron Lewis acid catalyst (Figure 2).

**Figure 2:**
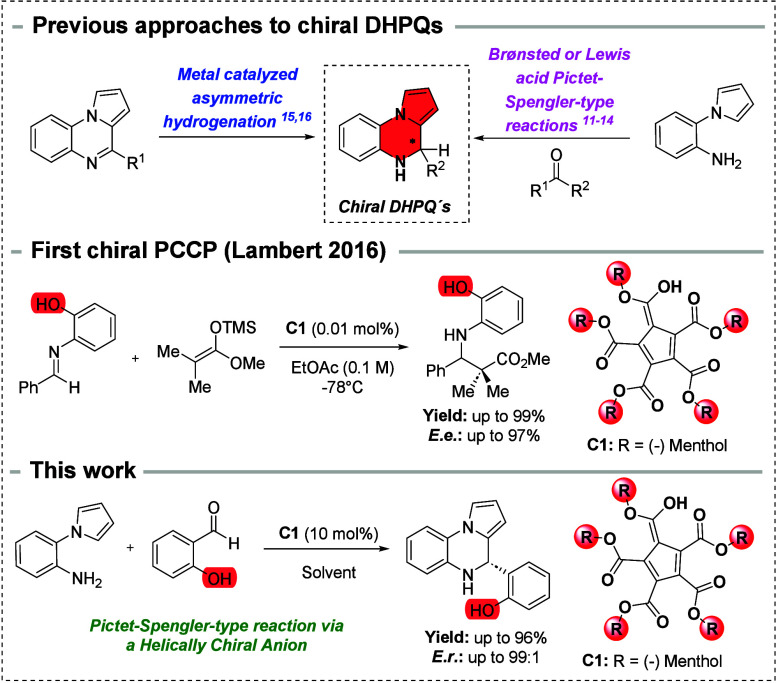
Reported strategies for the synthesis of chiral dihydropyrrolo­[1,2-*a*]­quinoxalines (DHPQs) derivatives and application of PCCP.

Further developments led to the synthesis of quaternary carbon-centered DHPQs via Brønsted acid catalysis. Zhang and co-workers developed the enantioselective synthesis of DHPQs from indolyl anilines and pyruvates under catalysis with H_8_–BINOL-type imidodiphosphoric acid.^12^ Soon after, Lin et al. and Zhou et al. showed that SPINOL-derived phosphoric acids or BINOL-derived phosphoramidates can efficiently catalyze Pictet–Spengler reaction of 2-(pyrrolyl)­anilines with α-ketoamides^13^ and aldehydes,^14^ respectively. Another approach for constructing chiral DHPQs was additionally reported by Zhou, who developed an elegant procedure based on iridium-catalyzed asymmetric hydrogenation of pyrrolo­[1,2-*a*]­quinoxalines.^15,16^ Despite these advances, the need for novel and efficient strategies for the synthesis of enantiomerically pure DHPQs remains urgent. This is particularly true in view of increasing demands for precise control over the absolute configuration of pharmacologically active molecules, which is essential for optimizing therapeutic efficacy and reducing drug toxicity.^17^ A promising alternative to existing Brønsted acidic catalytic systems involves the use of chiral pentacarboxycyclopentadienes (PCCPs). In 2016, Lambert and colleagues evaluated the newly synthesized PCCP catalysts in the well-established Mukaiyama–Mannich reaction of imines and silyl ketene acetals (Figure 2).^18^ These catalysts offer a cost-effective solution to the challenges of asymmetric synthesis, particularly when compared to the traditionally employed chiral phosphoric acids.^19–23^ Drawing inspiration from the previous works and our continuous interest in the construction of enantiomerically pure heterocycles,^24–26^ we aimed to establish a practical, cost-effective method for synthesizing enantiomerically pure DHPQs. Herein, we present the asymmetric synthesis of DHPQs from readily available starting materials via the Pictet–Spengler-type reaction utilizing chiral cyclopentadienes as catalysts.

## Results and Discussion

We initially started our investigation with a reaction of 2-(1*H*-pyrrol-1-yl)­aniline **1a** and benzaldehyde **2a** in the presence of chiral pentacarboxycyclopentadiene (**C1**) in 1 mL of toluene at room temperature. Unfortunately, the reaction provided the desired 4,5-dihydropyrrolo­[1,2-*a*]­quinoxaline (**3a**) in 90% yield as a racemate (Table 1, entry 1). Inspired by our previous work^24^ on aminalization reaction, we switched benzaldehyde **2a** to isovaraldehyde **2b** as a reaction partner in reaction with **1a** (Entry 2). In this case, we were able to synthesize product **3b** at room temperature in higher enantioselectivity (71:29 *e.r.*) and the same yield. Considering these observations, we lowered the temperature to −78 °C, but the enantiomeric purity of **3b** was only slightly increased (85:15 *e.r.*, entry 3).

**Table 1:**
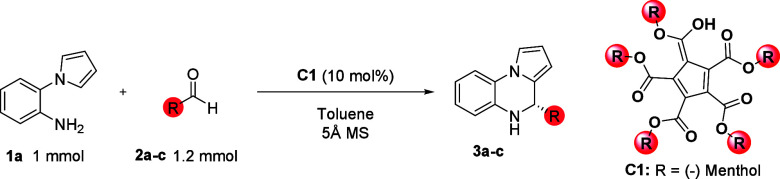
Condition Optimization

**Entry**	**Concentration [1a]**	**R**	**Temp [°C]**	**Time [h]**	**Yield**3a-c [%]^a^	E.r. [%]^b^
1	0.1 M	Ph (**2a**)	25	48	90	50:50
2	0.1 M	(CH_3_)_2_CHCH_2_ (**2b**)	25	48	90	71:29
3	0.1 M	(CH_3_)_2_CHCH_2_	–78	24	90	85:50
4	0.1 M	2-OHC_6_H4 (**2c**)	25	5	94	80:20
5	0.1 M	2-OHC_6_H_4_	–45	24	94	91:9
6	0.1 M	2-OHC_6_H_4_	–55	24	90	93:7
7	0.1 M	2-OHC_6_H_4_	–65	24	90	96:4
8	0.1 M	2-OHC_6_H_4_	–78	24	94	97:3
9	0.05 M	2-OHC_6_H_4_	–78	48	85	75:25
10	0.2 M	2-OHC_6_H_4_	–78	24	94	97:3
11	0.2 M	2-OHC_6_H_4_	–65	24	94	97:3
12	**0.2 M**	2-OHC_6_H_4_	**–55**	**18**	**94**	97:3
13	0.2 M	2-OHC_6_H_4_	–45	15	94	96:4

a Isolated yields after column chromatography.

b IC column (heptane/*iso*-propanol, 90:10, 1 mL/min).

Considering those results, it was necessary to revise the reaction process. Lambert’s reports indicated that the chiral pentacarboxycyclopentadiene catalyst (**C1**) exhibited optimal efficacy when the hydroxy group was positioned in the *ortho* position, either next to the imine or oxocarbenium.^18,19^ Thus, we tested salicylaldehyde **2c** as a reaction partner with (1*H*-pyrrol-1-yl)­aniline **1a** in the presence of catalyst **C1** in 1 mL of toluene at room temperature (entry 4).

As expected, the reaction provided the final 4,5-dihydropyrrolo­[1,2-*a*]­quinoxaline **3c** in high yield (93%) and with good enantiocontrol (80:20 *e.r.*). Based on this result, we chose salicylaldehyde **2c** for further reaction condition optimizations. Interestingly, the solvent investigation revealed that the model reaction tolerates various solvents, providing the desired product in high yields. Unfortunately, product **3c** was not obtained in higher enantiomeric purity in any case. For complete optimization studies, please see the Supporting Information (SI). Inspired by our previous investigation on aminalization reaction,^24^ we performed the model reaction at −45 °C to investigate the effect of lower temperature on enantiomeric outcome. Interestingly, the reaction between **1a** and **2c** at −45 °C in toluene provided the final product in high yield with high enantiocontrol (91:9 *e.r.*). Encouraged by these results, we continue testing lowered reaction temperatures in the range from −55 up to −78 °C (entries 6–8). The highest enantiocontrol was observed for the reaction performed at −78 °C, when the desired product **3c** was obtained in high isolated yield (94%) with high enantiomeric purity (97:3 *e.r.*). Additionally, we investigated the concentration dependence of the model reaction (entries 9–13). The lowered concentration (0.05 M of **1a**) at −78 °C in toluene resulted in a significant drop of enantiocontrol (75:25 *e.r.*). On the other hand, the increased concentration to 0.2 M did not have any effect on enantioselectivity and the yield of the reaction (entry 10). Further variations in reaction conditions showed that we were able to catalyze the developed enantioselective Pictet–Spengler-type reaction at −55 °C in toluene using 0.2 M concentration to obtain the desired product **3c** in excellent yield with a high enantiomeric excess (94%, 97:3 *e.r.*).

After establishing the optimal solvent and concentration, we investigated the model reaction under catalysis of various chiral Bronsted acids (Table 2). First, we investigated PCCPs reported by the Lambert group derived from chiral 2-phenylcyclohexan-1-ol (**C2**–**C5**). Unfortunately, catalysts **C2**–**C4** were found to be significantly less efficient compared to **C1**. Conversely, **C5** derived from (−)-isopinocamphenol provided the desired 4,5-dihydropyrrolo­[1,2-*a*]­quinoxaline (**3c**) in good yield (77%) and with an acceptable enantiomeric excess (88:22 *e.r.*). Furthermore, we tested camphorsulfonic acid (*R*)**-C6**, which provided the final product **3c** in low yield (23%) and as a racemate. We also tested different chiral phosphoric acid catalysts. Catalyst (*R*)**-C7** provided the final product in good yield (72%) but with low enantiomeric excess (53:47 *e.r.*). Commonly used (*R*)**-C8** and (*R*)-**C9** catalysts were able to provide desired 4,5-dihydropyrrolo­[1,2-*a*]­quinoxaline (**3c**) in high yields (81–90%) but unfortunately with moderate enantiomeric excess (70:30–68:32 *e.r.*). Testing more acidic *N*-triflyl phosphoramide catalyst (*R*)-**C10** resulted in low yield of final product **3c** (72%) and with moderate enantiomeric excess (70:30 *e.r.*). Additionally, we also tested SPINOL-derived catalyst (*R*)**-C11**, which provided the final product **3c** in low yield (27%) and with low enantiocontrol (54:46 *e.r.*). After establishing the optimal reaction conditions using **C1** (10 mol %) in toluene (0.2M) at −55 °C we investigated the effect of catalyst loading on the reaction outcome. However, lowering the catalyst loading down to 1 mol % led to significantly decreased yield of the reaction (30%) and enantiomeric excess of the final product (51:49 *e.r.*). Thus, we decided to continue using 10 mol % of **C1** as an optimal amount of catalyst. For complete optimization studies, please see the SI.

**Table 2:**
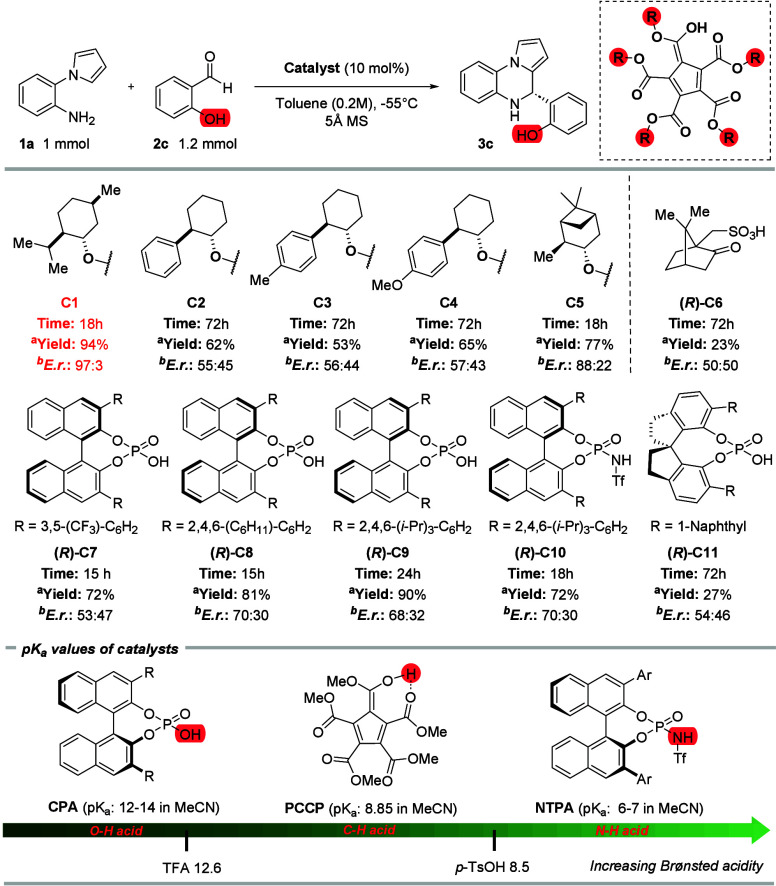
Catalyst Screening

a Isolated yields after column chromatography.

b IC column (heptane/*iso*-propanol, 90:10, 1 mL/min).

After establishing the optimal reaction conditions, we began exploring the scope of the developed Pictet–Spengler reaction with variously substituted salicylaldehydes (**2**, Figure 3). Initially, we tested the reactivity of methylated salicylaldehydes in *para* (**3d**), *meta* (**3e**), and *ortho* (**3f**) positions related to the hydroxy group. In the case of *para-*substituted salicylaldehyde (**3d**), we afforded the desired product **3d** in slightly lowered yield (70%) and with reduced enantiomeric purity (82:18 *e.r.*). Interestingly, *meta-*substituted salicylaldehyde (**3e**) provided the final product in 76% yield and with a high enantiomeric excess (93:7 *e.r.*). Unfortunately, *ortho*-substituted salicylaldehyde (**3f**) did not provide the final product. Next, we assessed the effect of the electronic properties of the salicylaldehydes **2** on the reaction outcome. Chiral 4,5-dihydropyrrolo­[1,2-*a*]­quinoxaline (**3g**) containing electron-rich methoxy group in *para*-position related to hydroxyl was prepared in high yield (85%) with a high enantiomeric excess (95:5 *e.r.*). Interestingly, regioisomeric 4,5-dihydropyrrolo­[1,2-*a*]­quinoxaline (**3h**) was synthesized in good yield (79%) but with low enantiocontrol (63:37 *e.r.*). Also, 4,5-dihydropyrrolo­[1,2-*a*]­quinoxaline (**3i**) was prepared in good yield (86%) but with low enantiocontrol (66:34 *e.r.*). Similarly, substrates containing electron-poor nitro, and cyano groups (**3j** and **3k**), were prepared in good yields (up to 72%) but nearly as racemates. This significant drop in enantiocontrol can be explained with parasitic racemic reaction catalyzed by the acidic phenolic moiety present in nitro and cyano-derived salicylaldehydes. To support our hypothesis, we performed analogous experiments without catalyst **C1**, providing final products **3j** and **3k** in similar yields (52–72%) as racemates. However, chiral 4,5-dihydropyrrolo­[1,2-*a*]­quinoxaline **3l** containing methyl ester in *para*-position was prepared in high yield (72%) with acceptable enantiomeric purity (76:24 *e.r.*). Next, we tested halogenated salicylaldehydes. In general, the reaction tolerates chlorinated (**3m**-**3n**) and brominated (**3o**-**3p**) salicylaldehydes and provides the final products in good yields (up to 95%) and with high enantiomeric control (up to 91:9 *e.r.*). Similarly, fluorinated salicylaldehydes gave the final products **3q** and **3r** in good yields (65–72%) with acceptable and high enantioselectivity (up to 92:8 *e.r.*). Interestingly, the reaction performed with an extended aromatic system using 3-hydroxy-2-naphthaldehyde delivered the corresponding product (**3s**) in excellent yield (96%) and with a high enantiomeric excess (97:3 *e.r.*). Moreover, we tested the reactivity of 1*H*-indole-2-carbaldehyde bearing pyrrole ring instead of phenol motif. The developed Pictet–Spengler reaction afforded 4,5-dihydropyrrolo­[1,2-*a*]­quinoxaline **3t** in high yield (85%) and with good enantiomeric control (84:17 *e.r.*), showing that not only *ortho*-hydroxy but also NH group can positively influence enantiocontrol of the reaction. As already mentioned, besides salicylaldehydes the developed Pictet–Spengler reaction also tolerates aliphatic aldehydes. For example, the reaction between **1a** and **2b** produced 4,5-dihydropyrrolo­[1,2-*a*]­quinoxaline **3b** in good yield (92%) with good enantioselectivity (85:15 *e.r.*). After that, we tested the scope of the developed Pictet–Spengler reaction using aliphatic and cyclic ketones. Introducing *N*-protected isatine resulted in a final spiro-compound (**3u**) in high yield (92%) but as a racemic mixture. Unfortunately, 2-hydroxyacetophenone did not provide the corresponding product **3v**. Subsequently, various *N*-substituted 2-(1*H*-pyrrol-1-yl)­anilines were tested in the studied reaction. Unfortunately, both *N*-acetylated and *N*-benzylated 2-(1*H*-pyrrol-1-yl)­anilines failed to afford the final cyclic products (**3w** and **3x**). We also investigated the reactivity of different substituents on the aniline ring. First, the derivatives bearing electron-rich methyl and methoxy group in *ortho*-position related to pyrrole substituent were tested. The desired products (**3y** and **3z**) were isolated in high yields (74% and 94%) but with lowered enantiomeric purity (63:37 and 87:13 *e.r.*). Similarly, anilines substituted in *meta* position related to pyrrole moiety with electron-rich and electron-deficient groups produced the corresponding 4,5-dihydropyrrolo­[1,2-*a*]­quinoxalines **3aa** and **3bb** in good to high yields but with only moderate enantiomeric excess (up to 84:16 *e.r.*). Additionally, anilines substituted in *para-*position to pyrrole moiety were tested. In the case of the brominated substrate, the reaction provided product **3cc** in good yield (72%) with poor enantiocontrol. Similar enantioselectivity and significantly reduced yield were observed when the aniline with electron-poor CF_3_ group was used (**3dd**, 25%, 63:37 *e.r.*).

**Figure 3:**
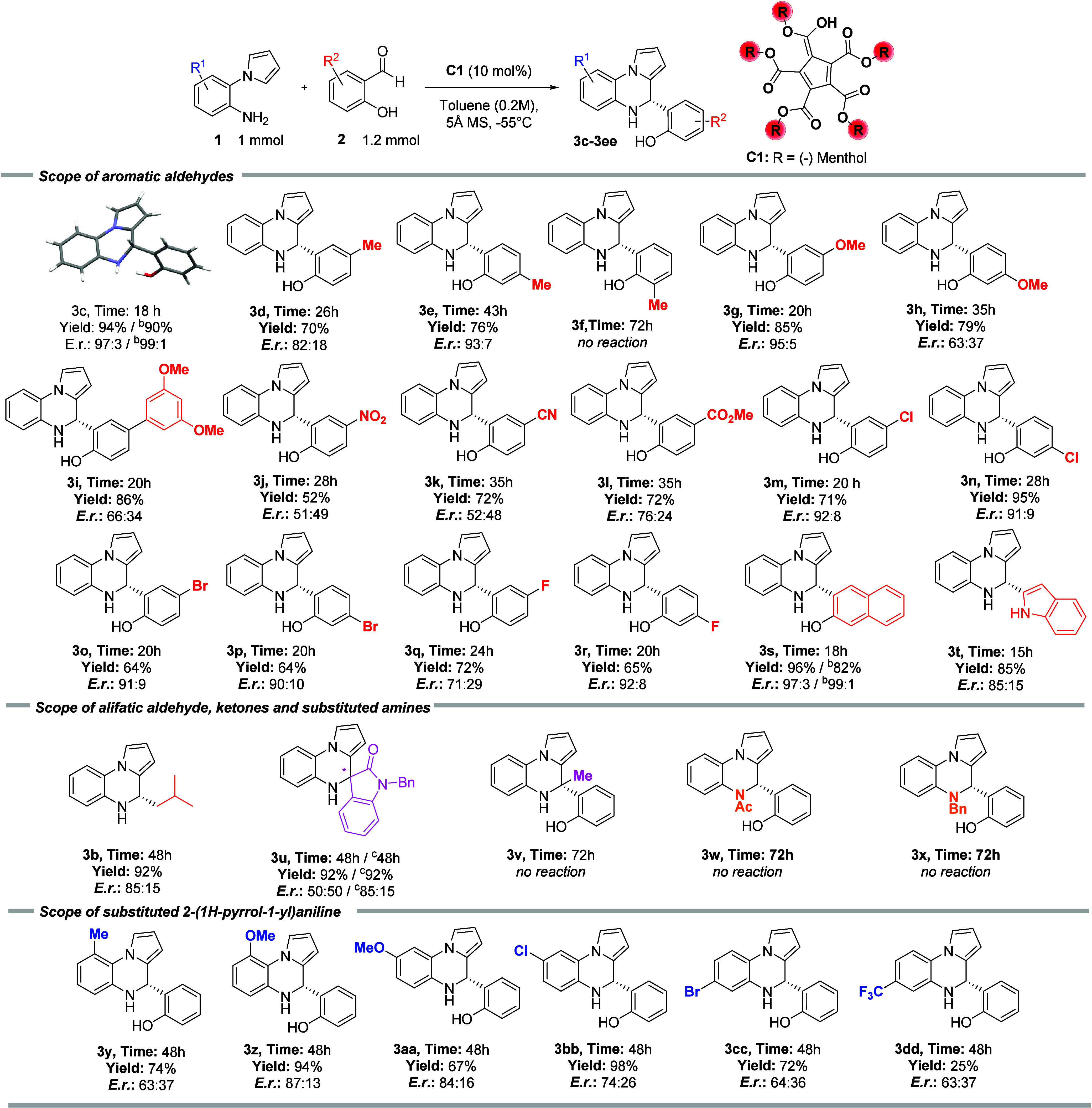
Substrate scope. ^a^Performed with **1a** (0.1 mmol), **2a**–**c** (0.12 mmol) and **C1** catalyst (10 mol %) in toluene (0.5 mL, 0.2 M) at −55 °C. Isolated yields after column chromatography and enantiomeric ration (*E.r.*) determined by HPLC analysis. ^b^Isolated yields after crystallization of reaction crude and enantiomeric ratio (*E.r.*) determined by HPLC analysis. ^c^Performed with **1a** (0.1 mmol), 1-benzylindoline-2,3-dione (0.12 mmol) and **(** ***R*** **)-C7** catalyst (10 mol %) in toluene (0.5 mL, 0.2M) at room temperature.

To demonstrate the synthetic utility of the developed Pictet–Spengler reaction, we performed a gram-scale reaction to synthesize chiral 4,5-dihydropyrrolo­[1,2-*a*]­quinoxaline **3c** using chiral pentacarboxycyclopentadiene **C1** in high yield (90%) with excellent enantioselectivity (99:1 *e.r*, Figure 4). Interestingly, we could separate final product **3c** in the scale-up process using crystallization instead of column chromatography. As an example of follow-up transformations, product **3c** was converted into derivatives **4** and **5**. Acetylation of **3c** using acetic anhydride in the presence of NaHCO_3_ gave *N*,*O*-protected product **4** in good yield (68%).

**Figure 4:**
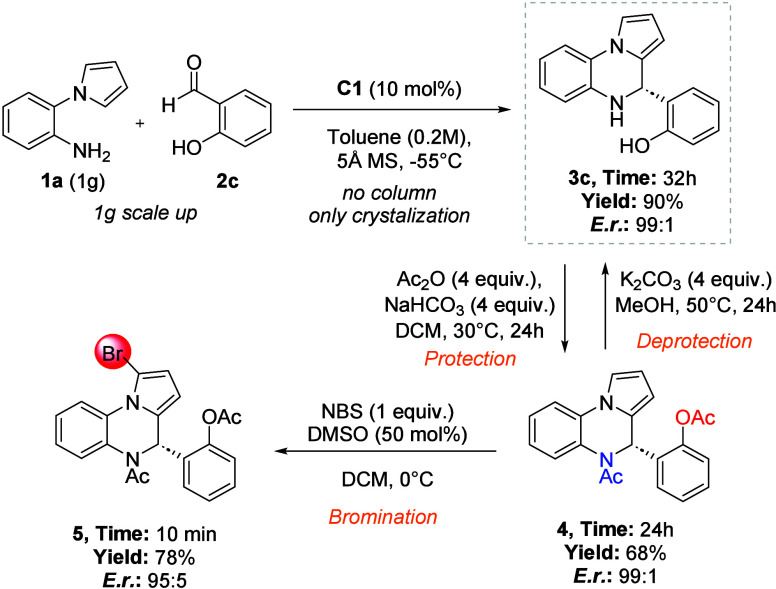
Gram-scale experiment and follow-up transformations.

Contrarily, the acetyl groups can be cleaved using K_2_CO_3_ in methanol to corresponding compound **3c** also in a good yield (82%). In both processes, the enantiomeric purity (99:1 *e.r.*) was maintained. Additionally, product **4** was selectively brominated using NBS (1 equiv) and DMSO to compound **5** in good yield with only slight change of the enantiomeric purity (95:5 *e.r.*).

The absolute configuration of **3c** and **3r** was ascertained using X-ray diffraction analysis, and the configuration of **3c** and **3r** was assigned as *S* (Figure 3, for details, see the SI). Absolute configurations of other 4,5-dihydropyrrolo­[1,2-*a*]­quinoxalines **3c** were assigned by analogy. Based on the observed absolute configuration of the product (*S*) and previous works,^19^ we proposed the reaction mechanism for enantioselective Pictet–Spengler reaction via helically chiral anion (Figure 5). Initially, acidic catalyst **C1** induces the formation of imine salt (**I**) from **1a** and **2c**. After that, the cyclization reaction proceeds through a transition state (**II**), in which the absolute stereochemistry is dictated by the helical chirality of the anion, helped by hydrogen bonding with the substrate, forming a more rigid system. We hypothesized that the nucleophilic attack is coming from the *Re*-face forming corresponding intermediate (**III**). Finally, deprotonation of the resulting intermediate (**III**) by the acid catalyst forms the final product as (*S*)-enantiomer and regenerates the catalyst **C1**. To support the proposed mechanism, we performed quantum chemistry calculations using density functional theory (DFT) with hybrid functional ωB97X-D that includes dispersion corrections and that is well suited for calculations of transition states.^31^ As the system consists in about 200 atoms, a modest split-valence basis set SVP was used for geometry optimization while the energy was refined at the stationary points of the potential energy surface (PES) using a fairly large QZVPP basis set.^32^ The calculations confirm the mechanism shown in Figure 5 (Cartesian coordinates of the key transition states II leading to both enantiomers are provided in the SI). The substrate is attached to the catalyst by a hydrogen bond via phenolic group with length 1.64 and 1.72 Å for *R-* and *S-* forms, respectivelly. The newly formed C–C bond length in the transition state is about 1.9 Å with the immaginary vibrational frequency corresponding to the barrier crossing of about 400*i* cm^–1^. The calculated energy barriers including the zero-point energy corrections differ by −2.9 kcal/mol for *R-* and *S-*forms. The corresponding Arrhenius factor at room temperature gives about 140 times faster reaction leading to *R-*form. All calculations were done using Gaussian 16 package^33^ with some PES scans automated using pyMCD.^34^

**Figure 5:**
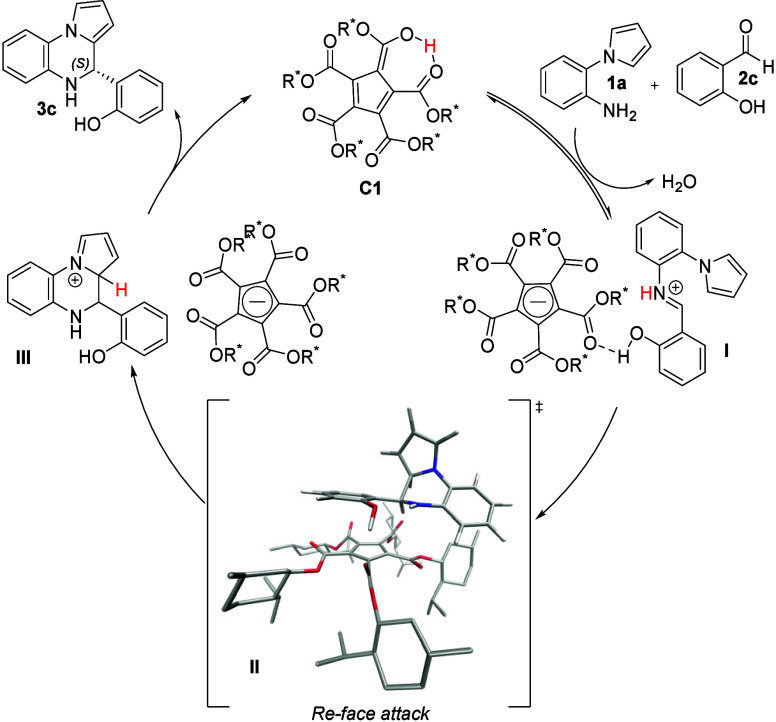
Plausible reaction mechanism.

## Conclusion

In summary, we have established a highly enantioselective Pictet–Spengler-type reaction facilitated by a cost-effective and readily accessible helically chiral cyclopentadiene (PCCP) catalyst. This protocol enables the efficient synthesis of a novel class of chiral 4,5-dihydropyrrolo­[1,2-*a*]­quinoxalines (DHPQs) in high yields (up to 96%) and with high enantioselectivities (up to 99:1 *e.r.*). The reaction exhibits broad substrate scope, mild operational conditions, and use of cheap catalyst, underscoring its potential as a powerful strategy for constructing structurally and functionally diverse chiral chiral 4,5-dihydropyrrolo­[1,2-*a*]­quinoxalines (DHPQs).

## Experimental Section

General Experimental, Reaction Setup, Reagents and Solvents: All enantiomeric reactions and further transformations were performed in 4.0 mL vial. All reagents were used as supplied from commercial sources without further purification unless otherwise stated. Tetrahydrofuran, Et_2_O, DCM and toluene were purified by distillation on site under inert atmosphere via the following processes: tetrahydrofuran and Et_2_O were predried over sodium wire then distilled. DCM, toluene were distilled from calcium hydride under inert atmosphere. EtOAc and *n*-hexanes were distilled. Chromatography: Analytical thin-layer chromatography was performed using precoated Merck aluminum silica gel plates (Silica gel 60 F254). Visualization was by ultraviolet fluorescence (λ = 254 and 365 nm) and/or staining with potassium permanganate (KMnO_4_) or Ceric Ammonium Molybdate (CAM). Flash column chromatography was performed using silica gel (230–400 mesh). All ratios of eluents are quoted as v/v. Chiral HPLC Analysis: Chiral HPLC was carried out using a LC20AD Shimadzu liquid chromatograph with SPDM20A diode array detector with columns Daicel Chiralpak IA, Daicel Chiralpak IC, Daicel Chiralpak Lux-Amylose (4.6 mm × 250 mm, 5.0 μm) in a mixed solvent system of heptane and isopropanol at 25 ◦C unless otherwise stated. The racemic compounds were prepared by treating respective anilines and aldehydes with PCCP (0.2 equiv) in toluene (0.1 M) at 25 °C. Samples for measurement of chiral HPLC were prepared by dissolving of corresponding products in heptane/i-PrOH (80/20 or 80/10 v/v). Names of the compounds: were generated by the computer program Chem Draw according to the guidelines specified by the IUPAC. NMR Spectroscopy: ^1^H NMR spectra were recorded on 400 or 600 MHz Bruker spectrometers. Chemical shifts are reported in parts per million (ppm) and the spectra are calibrated to the resonance resulting from incomplete deuteration of the solvent (CDCl_3_: 7.26 ppm, DMSO-d6: δ = 2.50 ppm). ^13^C NMR spectra were recorded on the same spectrometers with complete proton decoupling using CDCl_3_ as the internal standard (^13^CDCl_3_: 77.16 ppm, DMSO-d6: δ = 39.5 ppm) ^19^F NMR spectra were recorded on same spectrometer. Data are reported as follows: chemical shift δ, multiplicity (s = singlet, d = doublet, t = triplet, q = quartet, p = pentet, bs = broad singlet, m = multiplet or combinations there of ^13^C and all other nuclides except ^1^H are singlets unless otherwise stated. ^1^H NMR signals are reported in ppm to 2 decimal places and all other nuclide signals to 1 decimal place (^13^C NMR signals are reported in ppm to 2 decimal places where first decimal is exactly same). Coupling constants are reported in Hz to a maximum of 3 significant figures. High Resolution Mass Spectrometry (HRMS): High-resolution mass spectra’s were recorded with a LCQ Fleet spectrometer and LC-QTOF at the Department of Chemistry at the Charles University. The ionization method is noted positive/negative electrospray ionization (±ESI) or Atmospheric pressure photoionization (±APPI). Samples for measurement of HRMS were prepared by dissolving of corresponding sample in methanol/CH_3_CN. The masses reported as ‘found’ and ‘calculated for’ are the mass/charge ratios. Measured values are reported to 4 decimal places and are within ±5 ppm of the calculated value. The calculated values are based on the most abundant isotope unless otherwise stated in the chemical formula. IR Spectroscopy: IR DRIFT or ATR spectra’s were recorded with Nicolet AVATAR 370 FT-IR in cm^–1^. Optical Rotations: Measured in spectrophotometric grade CHCl_3_, MeOH or DMSO on AU-Tomatica polarimeter, Autopol III using a sodium lamp (λ = 589 nm, the sodium D line). [α]_D_ values are reported at the stated temperature, with concentration in g/100 mL. Single-Crystal X-ray diffraction the molecular as well as an absolute structure of **3c** and **3r** were determined performing X-ray diffraction experiment on Bruker D8 VENTURE Kappa Duo PHOTONIII by IμS microfocus sealed tube CuKα (λ= 1.54178) at a temperature of 120(2) K. The absolute configuration of the product **3r** was confirmed through a single X-ray analysis. The absolute configurations of other products were assigned tentatively by analogy.

### General Procedure for Epoxide Opening and Kinetic Resolution of Cyclohexanol Derivatives GP1 (SM1–3)

Arylhalide (1.2 equiv., 4.9 mmol) was dissolved in dry THF (1 mL/mmol) with activated magnesium turnings (1.4 equiv., 5.7 mmol; these were washed first with 1 M HCl followed by deionized H_2_O, acetone, and diethyl ether). A drop of 1,2-dibromo ethane was added to the solution mixture. This solution was refluxed for 1 h or until all the Mg was dissolved. The reaction was then cooled down to −30 °C and CuCN (0.05 equiv., 0.2 mmol) was added at this temperature. Cyclohexene oxide (1.0 equiv., 4.1 mmol) as a solution in THF (0.1 mL/mmol) was syringe pumped into the reaction flask over 1.5 h. This reaction mixture was then warmed to room temperature and stirred for 2 h. The reaction was quenched with sat. NH_4_Cl and extracted into ether. The crude material was purified with column chromatography. The pure racemic alcohol (1.0 equiv., 1.13 mmol) and vinyl acetate (10.0 equiv., 11.3 mmol) were dissolved in MTBE (8.14 mL/mmol with respect to the alcohol). ^1^H NMR was taken prior to adding Candia Antarctica Lipase B (50 mg/mmol with respect to the alcohol). After 24 h, the reaction was checked by ^1^H NMR to monitor the conversion of alcohol. If complete or close to 50% conversion, the reaction was filtered, concentrated in vacuo, and purified via column chromatography. The acetylated alcohol has a higher R_f_ than the free alcohol. The acetylated alcohol (1.0 equiv., 0.56 mmol) was deprotected by dissolving the alcohol in MeOH (4.38 mL/mmol) and adding crushed K_2_CO_3_ (3.0 equiv, 1.68 mmol). This was stirred at room temperature for 24 h and extracted out of EtOAc-H_2_O to obtain the free alcohol. This was not further purified and used as is.

#### (1*R*,2*S*)-2-Phenylcyclohexan-1-ol (**SM1**)

The product was prepared according to previously reported procedure **GP1** to obtain the title product as a white solid (from deprotected alcohol (200 mg (1.13 mmol), 40% (80 mg) yield) and experimental data is with accordance with literature.^19^ ^1^H NMR: (400 MHz, CDCl_3_) δ 7.39–7.30 (m, 2H), 7.28–7.20 (m, 3H), 3.67 (td, *J* = 10.1, 4.4 Hz, 1H), 2.44 (ddd, *J* = 12.3, 9.9, 3.6 Hz, 1H), 2.15–2.04 (m, 1H), 1.91–1.72 (m, 4H), 1.63–1.44 (m, 1H), 1.45–1.27 (m, 3H) ppm. ^13^C­{^1^H} NMR: (125 MHz, CDCl_3_) δ 143.4, 128.9, 128.0, 127.0, 74.6, 53.4, 34.6, 33.5, 26.2, 25.2 ppm. [α]^25^_D_ = −58.1 (1.0 c, MeOH). Enantiomeric excess: (*E.r.*) 99:1; retention times t_major_ = 6.1 min and *t*_minor_ = 16.1 min determined by HPLC (Chiralpak column Lux-Amylose, 90/10 *n*-heptane/isopropanol, flow rate of 1.0 mL/min, 25 °C, λ = 230 nm).

#### (1*R*,2*S*)-2-(p-Tolyl)­cyclohexan-1-ol (**SM2**)

The product was prepared according to previously reported procedure **GP1** to obtain the title product as a white solid (from deprotected alcohol (800 mg (4.2 mmol), 47% (376 mg) yield) and experimental data is with accordance with literature.^19^ ^1^H NMR: (400 MHz, CDCl_3_) δ 7.16 (s, 4H), 3.64 (td, *J* = 10.1, 4.3 Hz, 1H), 2.40 (ddd, *J* = 12.0, 10.0, 3.6 Hz, 1H), 2.34 (s, 3H), 2.15–2.07 (m, 1H), 1.85 (dp, *J* = 9.1, 3.0 Hz, 2H), 1.81–1.72 (m, 1H), 1.60 (s, 1H), 1.57–1.26 (m, 4H) ppm. ^13^C­{^1^H} NMR: (100 MHz, CDCl_3_) δ 140.3, 136.5, 129.6, 127.9, 74.6, 52.9, 34.5, 33.5, 26.2, 25.2, 21.2 ppm. [α]^25^_D_ = −51.8 (1.0 c, MeOH). Enantiomeric excess: (*E.r.*) 99:1; retention times t_major_ = 6.1 min and *t*_minor_ = 16.1 min determined by HPLC (Chiralpak column Lux-Amylose, 90/10 *n*-heptane/isopropanol, flow rate of 1.0 mL/min, 25 °C, λ = 230 nm).

#### (1*R*,2*S*)-2-(4-Methoxyphenyl)­cyclohexan-1-ol (**SM3**)

The product was prepared according to previously reported procedure **GP1** to obtain the title product as a white solid (from deprotected alcohol (200 mg (0.97 mmol), 40% (80 mg)­yield) and experimental data is with accordance with literature.^19^ ^1^H NMR: (400 MHz, CDCl_3_) δ 7.18 (d, *J* = 8.6 Hz, 2H), 6.92–6.84 (m, 2H), 3.80 (s, 3H), 3.60 (td, *J* = 10.1, 4.3 Hz, 1H), 2.38 (ddd, *J* = 12.8, 10.0, 3.6 Hz, 1H), 2.11 (dq, *J* = 9.0, 2.3 Hz, 1H), 1.85 (tdd, *J* = 11.0, 6.0, 3.2 Hz, 2H), 1.80–1.71 (m, 1H), 1.62 (s, 1H), 1.56–1.25 (m, 4H) ppm. ^13^C­{^1^H} NMR: (125 MHz, CDCl_3_) δ 158.6, 135.3, 128.9, 114.3, 74.7, 55.4, 52.5, 34.5, 33.6, 26.2, 25.2 ppm. [α]^25^_D_ = −58.1 (1.0 c, MeOH). Enantiomeric excess: (*E.r.*) 99:1; retention times t_major_ = 6.1 min and *t*_minor_ = 16.1 min determined by HPLC (Chiralpak column Lux-Amylose, 90/10 *n*-heptane/isopropanol, flow rate of 1.0 mL/min, 25 °C, λ = 230 nm).

#### Tetramethyl 5-(hydroxy­(methoxy)­methylene)­cyclopenta-1,3-diene-1,2,3,4-tetracarboxylate (**PCCP**)

**PCCP** was prepared according to known procedure and experimental data is with accordance with literature.;^18,19^ ^1^H NMR: (400 MHz, CDCl_3_): δ 20.08 (s, 1H), 4.04 (s, 6H), 3.90 (s, 6H), 3.76 (s, 3H) ppm. ^13^C­{^1^H} NMR: (101 MHz, CDCl_3_) 172.4 (2C), 167.8 (2C), 163.3, 133.8 (2C), 117.8, 106.5 (2C), 55.7 (2C), 52.7 (2C), 52.0 ppm. MS: (ESI+) *m*/*z*: calc. for C_15_H_15_O_10_ [M-H]^−^: 355.1, found: 355.0.

### General Procedure for Synthesis of PCCP Catalysts GP2 (C1–C5)

All catalysts were prepared by a procedure reported by Lambert et al.^18,19^ Cyclopentadiene PCCP (1.0 equiv., 1.403 mmol), alcohol (10 equiv., 14.03 mmol), and *N*-methylimidazole (6.0 equiv., 8.42 mmol) were dissolved in toluene (0.1 M) in a flame-dried two-neck round-bottom flask. A steady flow of N_2_ and 5Å molecular sieves allowed for the removal of methanol. The reaction solution was refluxed using an oil bath for 48 h while being monitored by TLC. Upon completion, the reaction solution was cooled down to room temperature and concentrated in vacuo. The crude material was purified by silica gel column chromatography (100 → 50% Et_2_O/Toluene). The purified material was subsequently washed with 1 M HCl/CH_2_Cl_2_ (3x), dried with anhydrous magnesium sulfate, and concentrated in vacuo to yield the title product.

#### Pentakis­((1R,2S,5R)-2-isopropyl-5-methylcyclohexyl) Cyclopenta-1,3-diene1,2,3,4,5-pentacarboxylate (**C1**)

The catalyst was synthesized according to general procedure GP2, using cyclopentadiene **PCCP** (500 mg, 1.403 mmol, 1.0 equiv). The product was obtained by column chromatography (silica, 100 → 50% Et_2_O/Toluene) as a brown solid in 89% (1.22 g) yield. ^1^H NMR: (400 MHz, CDCl_3_) δ 20.30 (s, 1H), 5.11–4.53 (m, 5H), 2.65–0.50 (m, 90H) ppm. ^13^C­{^1^H} NMR: (100 MHz, CDCl3) δ 172.1, 167.0, 162.6, 134.3, 118.9, 106.5, 81.3, 77.5, 77.2, 76.8, 76.7, 75.7, 47.5, 46.3, 46.2, 41.6, 40.8, 40.3, 34.6, 34.5, 34.1, 32.0, 31.7, 31.6, 25.6, 25.44, 25.36, 23.34, 23.30, 23.2, 23.1, 22.6, 22.4, 22.3, 22.2, 21.9, 21.3, 21.0, 16.8, 16.1, 15.8. HRMS: (ESI+) *m*/*z*: [M + H]^+^ Calcd for C_60_H_97_O_10_ 977.7076, Found 977.7077.

#### Pentakis­((1*R*,2*S*)-2-phenyl-1-cyclohexyl) Cyclopenta-1,3-diene-1,2,3,4,5-pentacarboxylate (**C2**)

The catalyst was synthesized according to general procedure GP2, using cyclopentadiene **PCCP** (100 mg, 0.2807 mmol, 1.0 equiv). The product was obtained by column chromatography (silica, 100 → 50% Et_2_O/Toluene) as a white solid in 75% (227 mg) yield. ^1^H NMR: (400 MHz, CDCl_3_) δ 18.99 (s, 1H), 7.65–6.38 (m, 15H), 5.58–4.33 (m, 5H), 4.00–0.46 (m, 45H) ppm. ^13^C­{^1^H} NMR: (100 MHz, CDCl_3_) δ 170.7, 167.7, 162.0, 161.6, 161.4, 144.3, 140.5, 140.0, 138.4, 136.5, 136.4, 135.9, 135.7, 135.6, 135.5, 135.4, 132.7, 129.6, 129.3, 129.2, 129.1, 128.9, 128.1, 127.9, 127.6, 127.4, 127.4, 127.3, 117.7, 106.6, 82.8, 81.6, 78.3, 77.9, 75.5, 74.9, 54.4, 53.0, 52.3, 52.2, 49.3, 48.2, 48.0, 47.5, 34.7, 34.6, 33.5, 32.5, 32.4, 32.1, 32.1, 31.7, 31.6, 29.8, 29.5, 26.3, 26.1, 26.1, 25.7, 25.6, 25.5, 25.2, 24.9, 24.8, 24.7, 24.6, 24.3, 21.3, 21.1 ppm. HRMS: (ESI+) *m*/*z*: calc. for C_70_H_76_O_10_Na [M + Na]^+^: 1099.5336, found: 1099.5337.

#### Pentakis­((1*R*,2*S*)-2-(4-methoxyphenyl)-1-cyclohexyl) Cyclopenta-1,3-diene-1,2,3,4,5- pentacarboxylate (**C3**)

The catalyst was synthesized according to general procedure **GP2**, using cyclopentadiene **PCCP** (100 mg, 0.2807 mmol, 1.0 equiv). The product was obtained by column chromatography (silica, 100 → 50% Et_2_O/Toluene) as a white solid in 68% (234 mg) yield. ^1^H NMR: (400 MHz, CDCl_3_) δ 18.90 (s, 1H, OH), 7.28–6.63 (m, 20H, ArH), 5.14–4.90 (m, 5H, OCH), 3.82–3.61 (m, 15H, OCH3 × 5), 2.90–0.93 (m, 45H). ^13^C­{^1^H} NMR: (125 MHz, CDCl_3_) 171.5, 170.8, 170.8, 170.4, 170.4, 167.0, 166.6, 166.4, 166.2, 162.6, 158.6, 158.4, 158.2, 157.9, 157.9, 157.8, 147.5, 136.0, 135.7, 135.6, 135.6, 134.3, 133.9, 133.8, 133.4, 133.4, 129.2, 129.0, 129.0, 128.9, 128.9, 128.8, 128.6, 128.4, 120.3, 114.3, 114.0, 113.8, 113.7, 113.6, 113.5, 109.2, 82.6, 80.9, 78.4, 78.0, 77.7, 77.4, 76.5, 74.7, 55.4, 55.3, 55.3, 55.2, 55.2, 55.1, 55.1, 55.0, 55.0, 54.9, 52.5, 48.8, 48.7, 47.7, 45.8, 34.5, 33.6, 32.2, 32.1, 31.9, 31.6, 26.3, 26.0, 25.8, 25.6, 25.5, 25.2, 25.0, 24.9, 24.8, 24.7, 24.6, 24.2, 23.7, 22.8, 21.6 ppm. HRMS: (ESI+) *m*/*z*: [M + Na]^+^ Calcd for C_75_H_86_O_15_Na 1249.5864, Found 1249.5866.

#### tetrakis­((1*R*,2*S*)-2-(p-tolyl)­cyclohexyl) 5-(hydroxy­(((1*R*,2*S*)-2-(p-tolyl)­cyclohexyl)­oxy)­methylene)­cyclopenta-1,3-diene-1,2,3,4-tetracarboxylate (**C4**)

The catalyst was synthesized according to general procedure GP2, using cyclopentadiene PCCP (100 mg, 0.2807 mmol, 1.0 equiv). The product was obtained by column chromatography (silica, 100 → 50% Et_2_O/Toluene) as a white solid in 49% (158 mg) yield. ^1^H NMR: (400 MHz, CDCl_3_) δ 19.42 (s, 1H), 7.20–6.77 (m, 20H), 5.37–4.67 (m, 5H), 2.77–1.06 (m, 60H) ppm. ^13^C­{^1^H} NMR: (151 MHz, CDCl_3_) δ 170.4, 167.4, 162.5, 143.4, 128.9, 128.8, 128.6, 128.4, 128.4, 128.3, 128.2, 128.0, 127.8, 127.5, 127.0, 126.6, 126.3, 80.7, 74.6, 53.4, 49.7, 49.6, 48.5, 34.6, 33.5, 32.7, 32.3, 31.8, 31.2, 25.9, 25.7, 25.5, 25.4, 25.2, 24.9, 24.8, 24.7, 24.7, 24.3, 24.2, 24.1 ppm. HRMS: (ESI+) *m*/*z*: [M + Na]^+^ Calcd for C_75_H_86_O_10_Na 1169.6118, Found 1169.6118.

#### 1,2,3,4,5-Pentacarbo-(−)-isopinocampheoxy­cyclopentadiene (**C5**)

The catalyst was synthesized according to general procedure **GP2**, using cyclopentadiene **PCCP** (500 mg, 1.403 mmol, 1.0 equiv). The product was obtained by column chromatography (silica, 100 → 50% Et_2_O/Toluene) as a white solid in 92% (1.25 g) yield. ^1^H NMR: (400 MHz, CDCl_3_) δ 20.25 (s, 1H), 5.77–4.89 (m, 5H), 2.72–1.53 (m, 38H), 1.47–0.69 (m, 61H) ppm. ^13^C­{^1^H} NMR: δ 172.2, 166.7, 163.4, 133.9, 129.2, 128.4, 119.2, 106.8, 81.0, 76.1, 74.9, 47.6, 43.8, 43.3, 42.7, 41.4, 41.0, 38.6, 38.3, 35.5, 35.2, 33.5, 33.3, 32.3, 27.5, 24.1, 24.0, 23.9, 23.9, 20.6 ppm. HRMS: (ESI+) *m*/*z*: [M + H]^+^ Calcd for C_60_H_87_O_10_ 967.6299, Found 967.6306.

### General Procedure for Synthesis Compounds GP3 (1b–g)

Starting compounds **1a** is commercially available. Substituted 2-(1H-pyrrol-1-yl)­anilines **1b**–**1g** were prepared in the following modified method.^27^ A mixture of substituted 2-nitro-aniline (1 equiv., 6.57 mmol) and 2,5-dimethoxytetrahydrofuran (1 equiv., 6.57 mmol) in acetic acid (0.2M) were refluxed using an oil bath with vigorous stirring. After full conversion of starting material, the reaction mixture was poured on ice and extracted with ethyl acetate three times (3 × 20 mL). The combined organic layers were dried with anhydrous Na_2_SO_4_ and the solvent was removed in vacuo to afford product. The products were used directly without any purification in next step. The round-bottom flask charged with 1-(2-nitrophenyl)-1H-pyrrole (1 equiv., 6.57 mmol), NiCl_2_·6H_2_O (0.2 equiv., 1.31 mmol), and a 10/1 CH_3_CN/H_2_O mixture was cooled to 0 °C. After the mixture had been stirred for 5 min, NaBH_4_ (4 equiv., 26.28 mmol) was added portionwise. A fine black precipitate immediately formed. Then, the mixture was stirred for the next 15 min at room temperature, and the reaction later quenched with aqueous NH_4_Cl. The reaction mixture was filtered through Celite and washed with excess MeOH. The MeOH was removed under high vacuum to afford the crude product. The residue obtained was dissolved in EtOAc and washed with water (thrice). The organic layer was collected and dried over Na_2_SO_4_. The solvent was removed on a rotary evaporator under reduced pressure and purified by silica gel column chromatography using a 5/1 hexane/EtOAc mixture, which gave the corresponding aniline compounds (**1b**–**g**).

#### 3-Methyl-2-(1H-pyrrol-1-yl)­aniline (**1b**)

The compound was synthesized according to general procedure **GP3**, using 2-methyl-6-nitroaniline (1g, 6.57 mmol, 1 equiv). The product was obtained by column chromatography (silica, 5/1 hexane/EtOAc) as a white solid in 70% yield. ^1^H NMR: (400 MHz, CDCl_3_) δ 7.10 (t, *J* = 7.8 Hz, 1H), 6.71 (s, 1H), 6.70–6.65 (m, 3H), 6.37 (t, *J* = 2.1 Hz, 2H), 3.85 (s, 2H), 2.01 (s, 3H) ppm. ^13^C­{^1^H} NMR: (101 MHz, CDCl_3_) δ 143.3, 137.1, 128.9, 127.3, 121.6, 120.4, 113.7, 109.5, 17.3 ppm. HRMS: (ESI) *m*/*z*: [M + H]^+^ Calcd for C_10_H_10_^35^ClN_2_ 193.0527, Found 193.0528.

#### 4-Methoxy-2-(1H-pyrrol-1-yl)­aniline (**1c**)

The compound was synthesized according to general procedure **GP3**, using 5-methoxy-2-nitroaniline (1g, 5.947 mmol, 1 equiv). The product was obtained by column chromatography (silica, 5/1 hexane/EtOAc) as a white solid in 70% yield. ^1^H NMR: (400 MHz, CDCl_3_): δ 6.86 (d, *J* = 2.1 Hz, 2H), 6.82–6.74 (m, 3H), 6.35 (t, *J* = 2.2 Hz, 2H), 3.76 (s, 3H), 3.45 (s, 2H) ppm. ^13^C­{^1^H} NMR: (101 MHz, CDCl_3_): δ 152.7, 135.4, 128.3, 121.8, 117.5, 114.9, 112.6, 109.6, 56.0 ppm. HRMS: (ESI) *m*/*z* [M + H]^+^ Calcd for C_11_H_13_N_2_O 189.1022, Found 189.1022.

#### 4-Chloro-2-(1H-pyrrol-1-yl)­aniline (**1d**)

The compound was synthesized according to general procedure **GP3**, using 5-chloro-2-nitroaniline (1g, 5.795 mmol, 1 equiv). The product was obtained by column chromatography (silica, 5/1 hexane/EtOAc) as a white solid in 70% yield. ^1^H NMR: (400 MHz, CDCl_3_): δ 7.18–7.10 (m, 2H), 6.82 (t, *J* = 2.1 Hz, 2H), 6.77 (d, *J* = 8.5 Hz, 1H), 6.34 (t, *J* = 2.1 Hz, 2H), 4.02 (s, 2H) ppm. ^13^C­{^1^H} NMR: (101 MHz, CDCl_3_): δ 140.2, 128.5, 127.2, 123.2, 121.7, 117.4, 110.1 (2C) ppm. HRMS: (ESI) *m*/*z* [M + H]^+^ Calcd for C_10_H_10_^35^ClN_2_ 193.0527, Found 193.0528.

#### 3-Methoxy-2-(1H-pyrrol-1-yl)­aniline (**1e**)

The compound was synthesized according to general procedure **GP3**, using 2-methoxy-6-nitroaniline (1g, 5.947 mmol, 1 equiv). The product was obtained by column chromatography (silica, 5/1 hexane/EtOAc) as a white solid in 70% yield. ^1^H NMR (400 MHz, CDCl_3_): δ 7.12 (t, *J* = 8.2 Hz, 1H), 6.71 (t, *J* = 2.2 Hz, 2H), 6.50–6.16 (m, 4H), 3.73 (s, 3H), 3.66 (s, 2H) ppm. ^13^C­{^1^H} NMR: (101 MHz, CDCl_3_): δ 156.5, 144.8, 129.2, 122.3, 116.5, 109.2 (2C), 108.5, 101.3, 56.0 ppm. HRMS: (ESI) *m*/*z*: [M + H]^+^ Calcd for C_11_H_13_N_2_O 189.1022, Found 189.1022.

#### 2-(1H-Pyrrol-1-yl)-5-(trifluoromethyl)­aniline (**1f**)

The compound was synthesized according to general procedure **GP3**, using 2-nitro-4-(trifluoromethyl)­aniline (1g, 4.851 mmol, 1 equiv). The product was obtained by column chromatography (silica, 5/1 hexane/EtOAc) as a white solid in 70% yield. ^1^H NMR: (400 MHz, CDCl_3_): δ 7.27–7.22 (m, 1H), 7.06 (d, *J* = 8.7 Hz, 2H), 6.86 (t, *J* = 2.1 Hz, 2H), 6.38 (t, *J* = 2.1 Hz, 2H), 4.20 (s, 2H) ppm. ^13^C­{^1^H} NMR: (101 MHz, CDCl_3_): 142.4, 131.4–130.1 (q, J = 32.2 Hz, 1 C), 130.0, 127.6, 126.0–122.3 (d, J = 270.6 Hz, 1 C), 121.6, 115.2–115.1 (q, J = 3.7 Hz, 1 C), 113.0–112.9 (q, J = 3.7 Hz, 1 C), 110.3 ppm. ^19^F NMR: (376 MHz, CDCl_3_): δ −62.79 ppm. HRMS (ESI) *m*/*z*: [M + H]^+^ Calcd for C_10_H_10_^35^ClN_2_ 193.0527, Found 193.0528.

#### 5-Bromo-2-(1H-pyrrol-1-yl)­aniline (**1g**)

The compound was synthesized according to general procedure **GP3**, using 4-bromo-2-nitroaniline (1g, 4.608 mmol, 1 equiv). The product was obtained by column chromatography (silica, 5/1 hexane/EtOAc) as a white solid in 70% yield. ^1^H NMR: (400 MHz, CDCl_3_): δ 7.00 (d, *J* = 8.3 Hz, 1H), 6.95 (d, *J* = 2.1 Hz, 1H), 6.89 (dd, *J* = 8.3, 2.1 Hz, 1H), 6.79 (t, *J* = 2.1 Hz, 2H), 6.35 (t, *J* = 2.1 Hz, 2H), 3.80 (s, 2H) ppm. ^13^C­{^1^H} NMR: (101 MHz, CDCl_3_): δ 143.5, 128.5, 126.5, 122.0, 121.7, 121.3, 118.7, 109.9 ppm. HRMS: (ESI) *m*/*z*: [M + H]^+^ Calcd for C_10_H_10_^79^BrN_2_ 237.0021, Found 237.0021.

#### N-(2-(1H-Pyrrol-1-yl)­phenyl)­acetamide (**1h**)

The compound **1h** was prepared via known procedure.^28^ 2-(1H-pyrrol-1-yl)­aniline (1g, 6.32 mmol, 1 equiv) and acetic anhydride (excess) was stirred at room temperature in DCM (20 mL). The crude was stirred at room temperature. After full conversion of starting material, the reaction was extracted with DCM (3 × 50 mL). The combined organic layers were washed with water (50 mL), solution of NaHCO_3_ (50 mL) and dried with anhydrous Na_2_SO_4_ and the solvent was removed in vacuo. The product was obtained by column chromatography (silica, 5/1 hexane/EtOAc) as a white solid in 70% (886 mg) yield. ^1^H NMR (400 MHz, CDCl_3_): δ 8.36 (d, *J* = 8.3 Hz, 1H), 7.38 (td, *J* = 7.9, 1.6 Hz, 1H), 7.27 (dd, *J* = 7.6, 1.5 Hz, 1H), 7.16 (td, *J* = 7.6, 1.4 Hz, 1H), 6.99 (s, 1H), 6.79 (t, *J* = 2.1 Hz, 2H), 6.40 (t, *J* = 2.1 Hz, 2H), 2.04 (s, 3H) ppm. ^13^C­{^1^H} NMR: (101 MHz, CDCl_3_) δ 168.5, 133.8, 130.7, 128.8 126.9, 124.3, 122.1, 121.7, 110.6, 24.9 ppm. HRMS: (ESI+) *m*/*z*: [M + H]^+^ Calcd for C_12_H_13_N_2_O 201.1022, Found 201.1020.

### Preparation of Starting Aldehydes (**2**)

Starting aldehydes **2b, 2c, 2d, 2e, 2f, 2g, 2h, 2i, 2j, 2k, 2l, 2m, 2n, 2o, 2p, 2q, 2r, 2t** and **2v** is commercially available.

#### 4-Hydroxy-3′,5′-dimethoxy-[1,1’-biphenyl]-3-carbaldehyde (**2i**)

The compound **2i** was prepared via known procedure and experimental data is with accordance with literature.^30^ 5-Bromosalicylaldehyde (1 g, 4.97 mmol, 1 equiv), K_2_CO_3_ (2.06 g, 14.9 mmol, 3 equiv), boronic acid (1.0 g, 5.47 mmol, 1.1 equiv), PPh_3_ (13 mg, 0.05 mmol, 1 mol %), and Pd­(OAc) (11 mg, 0.05 mmol, 1 mol %) were taken in DMF:H_2_O (1:1) (12 mL). The mixture was stirred at room temperature under an atmosphere of nitrogen for 24 h. The reaction mixture was acidified using HCl (1N) on an ice bath, followed by extraction with ethyl acetate (3 × 50 mL). The extracts were combined, dried (MgSO_4_), and the solvent was removed under vacuum. The crude solid was purified by flash chromatography to isolate the desired aldehyde **2i**. The product was obtained by column chromatography (silica, 5/1 hexane/EtOAc) as a yellow solid in 45% (577 mg) yield. ^1^H NMR (400 MHz, CDCl_3_): δ 11.01 (s, 1H), 9.97 (s, 1H), 7.75 (d, *J* = 7.7 Hz, 2H), 7.11–7.01 (m, 1H), 6.67 (d, *J* = 2.2 Hz, 2H), 6.47 (t, *J* = 2.3 Hz, 1H), 3.86 (s, 6H) ppm. ^13^C­{^1^H} NMR: (101 MHz, CDCl_3_): δ 196.8, 161.4, 161.3, 141.7, 135.9, 133.4, 132.1, 120.8, 118.2, 105.2, 99.3, 55.6 ppm.

#### 3-Hydroxy-2-naphthaldehyde (**2s**)

The compound **2s** was prepared via known procedure and experimental data is with accordance with literature.^29^ 3-methoxy-2-naphthaldehyde (372 mg, 2 mmol, 1 equiv) was dissolved in dry DCM (20 mL) under argon atmosphere. The excess of AlCl_3_ was added portion wise. After full conversion of starting material, the reaction was quenched by water (50 mL) and extracted with DCM (3 × 20 mL). The combined organic layers were dried with anhydrous Na_2_SO_4_ and the solvent was removed in vacuo to afford **2s**. The product was obtained by column chromatography (silica, 5/1 hexane/EtOAc) as a yellow solid in 70% (241 mg) yield. ^1^H NMR (400 MHz, CDCl_3_): δ 10.32 (s, 1H), 10.09 (s, 1H), 8.15 (s, 1H), 7.87 (dd, *J* = 8.3, 1.2 Hz, 1H), 7.72 (dd, *J* = 8.4, 1.2 Hz, 1H), 7.57 (ddd, *J* = 8.2, 6.8, 1.3 Hz, 1H), 7.38 (ddd, *J* = 8.1, 6.8, 1.2 Hz, 1H), 7.29 (s, 1H) ppm. ^13^C­{^1^H} NMR: (101 MHz, CDCl_3_): δ 196.8, 156.0, 138.4, 138.0, 130.4, 129.5, 127.6, 126.8, 124.6, 122.5, 112.1 ppm.

### General Procedure for Synthesis of Chiral 4,5-Dihydropyrrolo­[1,2-*a*]­quinoxalines GP4 (**3b**–**3dd**)

#### Method A

To the mixture of substituted aniline **1** (0.1 mmol, 1 equiv) and **C1** (10 mol %, 0,01 mmol) in 0.25 mL of toluene in the presence of molecular sieves (5Å) at −55 °C, substituted salicylaldehyde **2** (0.12 equiv), dissolved in 0.25 mL, was added (0.2M). The mixture was stirred until full conversion and after that directly transferred to the column and separated on silica to yield the final product **3**. **Method B:** To the mixture of substituted aniline **1** (0.1 mmol, 1 equiv) and **C1** (10 mol %, 0,01 mmol) in 0.25 mL of toluene in the presence of molecular sieves (5Å) at −55 °C, substituted salicylaldehyde **2** (0.12 equiv), dissolved in 0.25 mL, was added (0.2M). The mixture was stirred until full conversion and after that the mixture was filtered through cotton, solvent was evaporated and reaction mixture was crystallized from hexane/DCM (20:1) to yield the final product **3.**

#### (S)-4-Isobutyl-4,5-dihydropyrrolo­[1,2-*a*]­quinoxaline (**3b**)

The compound was synthesized according to general procedure **GP4** (Method A), the product was obtained by column chromatography (silica, 5/1 hexane/EtOAc) as a white solid in 92% (8.3 mg) yield. ^1^H NMR: (400 MHz, CDCl_3_) δ 7.35–7.24 (m, 1H), 7.18–7.10 (m, 1H), 6.96 (td, *J* = 7.6, 1.4 Hz, 1H), 6.82 (td, *J* = 7.6, 1.4 Hz, 1H), 6.75 (dd, *J* = 7.7, 1.3 Hz, 1H), 6.30 (t, *J* = 3.2 Hz, 1H), 6.03–5.96 (m, 1H), 4.50 (dd, *J* = 8.1, 5.3 Hz, 1H), 3.98 (s, 1H), 1.79 (tdd, *J* = 14.4, 5.8, 2.1 Hz, 1H), 1.74–1.63 (m, 2H), 1.00 (dd, *J* = 16.7, 6.4 Hz, 6H) ppm. ^13^C­{^1^H} NMR: (101 MHz, CDCl_3_): δ 135.9, 130.2, 125.8, 124.7, 119.3, 115.6, 114.8, 114.3, 110.0, 110.0, 103.8, 49.0, 44.3, 29.6, 24.7, 23.5, 22.1 ppm. IR (ATR): 3363, 3103, 2954, 2868, 1514, 1294, 737 cm^–1^. HRMS: (ESI) *m*/*z*: [M + H]^+^ Calcd for C_18_H_14_F_3_N_2_O 331.1053, Found 331.1052. [α]^25^_D_ = +5.7° (c = 0.035, DMSO). Enantiomeric excess: (*E.r.*) 85:15; retention times t_major_ = 6.0 min and *t*_minor_ = 4.9 min determined by HPLC (Chiralpak column IC, 90/10 *n*-heptane/isopropanol, flow rate of 1.0 mL/min, 25 °C, λ = 190 nm).

#### (S)-2-(4,5-Dihydropyrrolo­[1,2-*a*]­quinoxalin-4-yl)­phenol (**3c**)

The compound was synthesized according to general procedure **GP4**, the product was obtained by column chromatography (silica, 5/1 hexane/EtOAc) as a white solid in 94% (24.7 mg) yield (Method A) or using recrystallization from reaction crude (20/1 hexane/DCM) as a white crystal in 90% (23.6 mg) yield (Method B). A crystal suitable for X-ray analysis was grown by the dissolution of **3c** in a mixture of hexane/DCM (20:1), followed by standing at room temperature overnight. ^1^H NMR: (400 MHz, CDCl_3_): δ 8.25 (s, 1H), 7.39–7.35 (m, 1H), 7.31 (ddd, *J* = 8.1, 7.4, 1.7 Hz, 1H), 7.21 (ddd, *J* = 3.0, 1.6, 0.7 Hz, 1H), 7.14 (dd, *J* = 7.5, 1.7 Hz, 1H), 7.06–6.98 (m, 2H), 6.95 (dd, *J* = 8.2, 1.2 Hz, 1H), 6.91 (td, *J* = 7.4, 1.2 Hz, 1H), 6.89–6.86 (m, 1H), 6.27–6.22 (m, 1H), 5.59 (dt, *J* = 3.5, 1.4 Hz, 1H), 5.55 (s, 1H), 4.40 (s, 1H) ppm. ^13^C­{^1^H} NMR: (101 MHz, CDCl_3_): δ 156.8, 134.8, 130.3, 129.8, 128.3, 127.1, 124.8, 122.8, 122.1, 119.8, 117.7, 117.2, 115.4, 115.3, 110.8, 106.7, 56.7 ppm. IR (ATR): 3282, 2918, 2858, 1608, 1589, 1518, 1473, 1246, 947, 756 cm^–1^. HRMS: (ESI) *m*/*z*: [M + H]^+^ Calcd for C_17_H_15_N_2_O 263.1179, Found 263.1178. [α]^25^_D_ = −68.6° (c = 0.35, MeOH). M.p.: 185–190 °C. Enantiomeric excess: (*E.r.*) 99:1; retention times t_major_ = 6.1 min and *t*_minor_ = 16.1 min determined by HPLC (Chiralpak column IC, 90/10 *n*-heptane/isopropanol, flow rate of 1.0 mL/min, 25 °C, λ = 230 nm).

#### (S)-2-(4,5-Dihydropyrrolo­[1,2-*a*]­quinoxalin-4-yl)-4-methylphenol (**3d**)

The compound was synthesized according to general procedure **GP4** (Method A), the product was obtained by column chromatography (silica, 5/1 hexane/EtOAc) as a white solid in 70% (19.3 mg) yield. ^1^H NMR: (400 MHz, CDCl_3_): δ 8.00 (s, 1H), 7.40–7.34 (m, 1H), 7.21 (dd, *J* = 3.1, 1.5 Hz, 1H), 7.10 (dd, *J* = 8.3, 2.2 Hz, 1H), 7.06–6.97 (m, 2H), 6.94 (d, *J* = 2.2 Hz, 1H), 6.88–6.83 (m, 2H), 6.25 (t, *J* = 3.2 Hz, 1H), 5.61 (dt, *J* = 3.1, 1.4 Hz, 1H), 5.48 (s, 1H), 4.37 (s, 1H), 2.31 (s, 3H) ppm. ^13^C­{^1^H} NMR: (101 MHz, CDCl_3_): δ 154.4, 134.9, 130.7, 130.3, 128.9, 128.4, 127.0, 124.7, 122.5, 121.9, 117.5, 117.2, 115.4, 115.2, 110.8, 106.6, 56.6, 20.6 ppm. IR (ATR): 3273, 2914, 2856, 1608, 1520, 1518, 1473, 1246, 947, 756 cm^–1^. HRMS: (ESI) *m*/*z*: [M + H]^+^ Calcd for C_18_H_17_N_2_O 277.1335, Found 277.1336. [α]^25^_D_ = −17.1° (c = 0.35, MeOH). Enantiomeric excess: (*E.r.*) 82:18; retention times t_major_ = 6.5 min and *t*_minor_ = 22.3 min determined by HPLC (Chiralpak column IC, 90/10 *n*-heptane/isopropanol, flow rate of 1.0 mL/min, 25 °C, λ = 230 nm).

#### (S)-2-(4,5-Dihydropyrrolo­[1,2-*a*]­quinoxalin-4-yl)-5-methylphenol (**3e**)

The compound was synthesized according to general procedure **GP4** (Method A), the product was obtained by column chromatography (silica, 5/1 hexane/EtOAc) as a white solid in 76% (21 mg) yield. ^1^H NMR: (400 MHz, (CD_3_)­SO): δ 9.56 (s, 1H), 7.47 (dd, *J* = 8.0, 1.3 Hz, 1H), 7.40 (dd, *J* = 3.0, 1.6 Hz, 1H), 6.92–6.82 (m, 3H), 6.72–6.67 (m, 1H), 6.67–6.65 (m, 1H), 6.50 (dd, *J* = 8.0, 1.7 Hz, 1H), 6.19 (s, 1H), 6.17 (t, *J* = 3.2 Hz, 1H), 5.79 (s, 1H), 5.68 (ddd, *J* = 3.4, 1.5, 0.8 Hz, 1H), 2.18 (s, 3H) ppm. ^13^C­{^1^H} NMR: (101 MHz, (CD_3_)­SO): δ 154.2, 137.5, 136.8, 128.6, 127.4, 126.3, 124.5, 124.3, 119.6, 117.6, 115.7, 115.2, 114.5, 114.2, 109.8, 104.7, 47.8, 20.8 ppm. IR (ATR): cm^–1^. HRMS: (ESI) *m*/*z*: [M + H]^+^ Calcd for C_18_H_17_N_2_O 277.1335, Found 277.1336. [α]^25^_D_ = +21.4° (c = 0.35, DMSO). Enantiomeric excess: (*E.r.*) 93:7; retention times t_major_ = 6.5 min and *t*_minor_ = 21.5 min determined by HPLC (Chiralpak column IC, 90/10 *n*-heptane/isopropanol, flow rate of 1.0 mL/min, 25 °C, λ = 230 nm).

#### (S)-2-(4,5-Dihydropyrrolo­[1,2-*a*]­quinoxalin-4-yl)-4-methoxyphenol (**3g**)

The compound was synthesized according to general procedure **GP4** (Method A), the product was obtained by column chromatography (silica, 5/1 hexane/EtOAc) as a white solid in 85% (24.8 mg) yield. ^1^H NMR: (400 MHz, CDCl_3_) δ 7.77 (s, 1H), 7.36 (dd, *J* = 7.5, 1.9 Hz, 1H), 7.21 (dd, *J* = 3.1, 1.6 Hz, 1H), 7.08–6.95 (m, 2H), 6.91–6.82 (m, 3H), 6.72 (dd, *J* = 2.4, 1.0 Hz, 1H), 6.25 (t, *J* = 3.2 Hz, 1H), 5.64 (dt, *J* = 3.4, 1.3 Hz, 1H), 5.51 (d, *J* = 1.7 Hz, 1H), 4.40 (s, 1H), 3.78 (s, 3H) ppm. ^13^C­{^1^H} NMR: (101 MHz, CDCl_3_): δ 152.9, 150.5, 134.8, 128.1, 127.0, 124.8, 123.5, 122.0, 118.2, 117.2, 115.4, 115.4, 115.3, 115.6, 110.8, 106.7, 56.6, 56.0 ppm. IR (ATR): 3302, 3047, 2864, 1606, 1518, 773, 476 cm^–1^. HRMS: (ESI) *m*/*z*: [M + H]^+^ Calcd for C_18_H_17_N_2_O_2_ 293.1285, Found 293.1282. [α]^25^_D_ = −22.9° (c = 0.35, MeOH). Enantiomeric excess: (*E.r.*) 95:5; retention times t_major_ = 8.9 min and *t*_minor_ = 26.9 min determined by HPLC (Chiralpak column IC, 90/10 *n*-heptane/isopropanol, flow rate of 1.0 mL/min, 25 °C, λ = 232 nm).

#### (S)-2-(4,5-Dihydropyrrolo­[1,2-*a*]­quinoxalin-4-yl)-5-methoxyphenol (**3h**)

The compound was synthesized according to general procedure **GP4** (Method A), the product was obtained by column chromatography (silica, 5/1 hexane/EtOAc) as a white solid in 79% (23.1 mg) yield. ^1^H NMR: (400 MHz, (CD_3_)­SO) δ 9.70 (s, 1H), 7.47 (dd, *J* = 8.0, 1.3 Hz, 1H), 7.40 (dd, *J* = 3.0, 1.6 Hz, 1H), 6.91–6.81 (m, 3H), 6.69 (ddd, *J* = 8.5, 7.1, 1.8 Hz, 1H), 6.41 (d, *J* = 2.5 Hz, 1H), 6.30 (dd, *J* = 8.5, 2.5 Hz, 1H), 6.17 (t, *J* = 3.2 Hz, 2H), 5.75 (s, 1H), 5.68–5.62 (m, 1H), 3.66 (s, 3H) ppm. ^13^C­{^1^H} NMR: (101 MHz, (CD_3_)­SO) δ 159.4, 155.3, 136.8, 128.8, 128.3, 124.5, 124.4, 121.6, 117.6, 115.2, 114.5, 114.2, 109.8, 104.7, 104.3, 101.0, 54.9, 50.6, 47.6 ppm. IR (ATR): 3278, 2949, 1604, 1437, 1242, 741 cm^–1^. HRMS: (ESI) *m*/*z*: [M + H]^+^ Calcd for C_18_H_17_N_2_O_2_ 293.1285, Found 293.1282. [α]^25^_D_ = +25.7° (c = 0.035, DMSO). Enantiomeric excess (*E.r.*) 63:37; retention times t_major_ = 8.6 min and *t*_minor_ = 20.9 min determined by HPLC (Chiralpak column IC, 90/10 *n*-heptane/isopropanol, flow rate of 1.0 mL/min, 25 °C, λ = 243 nm).

#### (S)-3-(4,5-Dihydropyrrolo­[1,2-*a*]­quinoxalin-4-yl)-3′,5′-dimethoxy-[1,1′-biphenyl]-4-ol (**3i**)

The compound was synthesized according to general procedure **GP4** (Method A), the product was obtained by column chromatography (silica, 5/1 hexane/EtOAc) as a white solid in 86% (34.3 mg) yield. ^1^H NMR: (400 MHz, CDCl_3_) δ 8.34 (s, 1H), 7.54 (dd, *J* = 8.4, 2.3 Hz, 1H), 7.38 (dt, *J* = 4.2, 1.8 Hz, 2H), 7.25–7.15 (m, 2H), 7.09–6.97 (m, 3H), 6.93–6.86 (m, 1H), 6.70 (d, *J* = 2.2 Hz, 2H), 6.44 (t, *J* = 2.3 Hz, 1H), 6.26 (t, *J* = 3.2 Hz, 1H), 5.70–5.60 (m, 2H), 4.44 (s, 1H), 3.84 (s, 6H) ppm. ^13^C­{^1^H} NMR: (101 MHz, CDCl_3_): δ 161.3, 156.7, 142.9, 134.7, 132.9, 129.2, 128.9, 128.5, 128.4, 128.1, 127.1, 124.8, 122.9, 122.2, 118.1, 117.3, 115.5, 115.3, 110.9, 106.9, 105.1, 98.9, 56.9, 55.6 ppm. IR (ATR): 3302, 3059, 2935, 1593, 1506, 1421, 1149, 822 cm^–1^. HRMS: (ESI) *m*/*z*: [M + H]^+^ Calcd for C_25_H_23_N_2_O_3_ 399.1703, Found 399.1700. [α]^25^_D_ = +15.7° (c = 0.035, DMSO). Enantiomeric excess: (*E.r.*) 66:34; retention times t_major_ = 10.7 min and *t*_minor_ = 25.9 min determined by HPLC (Chiralpak column IC, 90/10 *n*-heptane/isopropanol, flow rate of 1.0 mL/min, 25 °C, λ = 209 nm).

#### (S)-2-(4,5-Dihydropyrrolo­[1,2-*a*]­quinoxalin-4-yl)-4-nitrophenol (**3j**)

The compound was synthesized according to general procedure **GP4** (Method A), the product was obtained by column chromatography (silica, 5/1 hexane/EtOAc) as a white solid in 52% (16 mg) yield. ^1^H NMR: (400 MHz, CDCl_3_) δ 7.79–7.71 (m, 2H), 7.42–7.36 (m, 1H), 7.30–7.26 (m, 1H), 7.24 (dd, *J* = 3.1, 1.5 Hz, 1H), 7.12–7.02 (m, 2H), 6.95 (dt, *J* = 4.6, 3.2 Hz, 1H), 6.27 (t, *J* = 3.3 Hz, 1H), 5.71 (s, 1H), 5.57 (dt, *J* = 3.3, 1.4 Hz, 1H) ppm. ^13^C­{^1^H} NMR: (101 MHz, CDCl_3_): δ 157.7, 149.5, 133.3, 130.2, 129.5, 127.2, 126.3, 125.1, 123.1, 117.9, 116.0, 115.5, 114.6, 113.0, 111.0, 107.1, 56.2 ppm. IR (ATR): 3413, 3269, 1612, 1510, 1321, 1254, 1201, 1072, 748 cm^–1^. HRMS: (ESI) *m*/*z*: [M + H]^+^ Calcd for C_17_H_14_N_3_O_3_ 308.1030, Found 308.1026. Enantiomeric excess: (*E.r.*) 51:49; retention times t_major_ = 15.0 min and *t*_minor_ = 18.6 min determined by HPLC (Chiralpak column IC, 90/10 *n*-heptane/isopropanol, flow rate of 1.0 mL/min, 25 °C, λ = 297 nm).

#### (S)-3-(4,5-Dihydropyrrolo­[1,2-*a*]­quinoxalin-4-yl)-4-hydroxybenzonitrile (**3k**)

The compound was synthesized according to general procedure **GP4** (Method A), the product was obtained by column chromatography (silica, 5/1 hexane/EtOAc) as a white solid in 72% (20,7 mg) yield. ^1^H NMR: (400 MHz, (CD_3_)­SO) δ 10.21 (s, 1H), 7.65 (d, *J* = 8.2 Hz, 1H), 7.59 (d, *J* = 8.2 Hz, 1H), 7.55–7.50 (m, 1H), 7.49 (s, 1H), 7.46 (dd, *J* = 3.0, 1.6 Hz, 1H), 7.34 (ddd, *J* = 8.1, 6.7, 1.2 Hz, 1H), 7.23–7.16 (m, 2H), 6.96–6.86 (m, 2H), 6.71 (ddd, *J* = 8.4, 5.6, 3.0 Hz, 1H), 6.37 (s, 1H), 6.20 (t, *J* = 3.2 Hz, 1H), 5.97 (s, 1H), 5.76 (dd, *J* = 3.7, 1.5 Hz, 1H) ppm. ^13^C­{^1^H} NMR: (101 MHz, (CD_3_)­SO) δ 153.2, 136.5, 133.7, 132.2, 127.9, 127.5, 127.4, 126.4, 126.0, 125.5, 124.6, 124.3, 122.9, 117.7, 115.2, 114.5, 114.4, 109.9, 108.8, 105.1, 48.5 ppm. IR (ATR): 3288, 3113, 2224, 1608, 1489, 1254, 752 cm^–1^. HRMS: (ESI) *m*/*z*: [M + H]^+^ Calcd for C_18_H_14_N_3_O 288.1131, Found 288.1127. Enantiomeric excess: (*E.r.*) 52:48; retention times t_major_ = 13.7 min and *t*_minor_ = 18.0 min determined by HPLC (Chiralpak column IC, 90/10 *n*-heptane/isopropanol, flow rate of 1.0 mL/min, 25 °C, λ = X nm).

#### Methyl (*S*)-3-(4,5-Dihydropyrrolo­[1,2-*a*]­quinoxalin-4-yl)-4-hydroxybenzoate (**3l**)

The compound was synthesized according to general procedure **GP4** (Method A), the product was obtained by column chromatography (silica, 5/1 hexane/EtOAc) as a white solid in 72% (23,1 mg) yield. ^1^H NMR: (400 MHz, (CD_3_)­SO) δ 10.20 (s, 1H), 7.50 (dd, *J* = 8.0, 1.3 Hz, 1H), 7.47 (d, *J* = 1.7 Hz, 1H), 7.43 (dd, *J* = 3.0, 1.6 Hz, 1H), 7.32 (dd, *J* = 8.0, 1.7 Hz, 1H), 7.14 (d, *J* = 8.0 Hz, 1H), 6.91 (td, *J* = 7.5, 7.0, 1.3 Hz, 1H), 6.86 (dd, *J* = 7.9, 1.6 Hz, 1H), 6.71 (ddd, *J* = 8.5, 7.1, 1.6 Hz, 1H), 6.38 (s, 1H), 6.18 (t, *J* = 3.2 Hz, 1H), 5.89 (s, 1H), 5.72 (dd, *J* = 3.7, 1.5 Hz, 1H), 3.80 (s, 3H) ppm. ^13^C­{^1^H} NMR: (101 MHz, (CD_3_)­SO) δ 166.0, 154.3, 136.4, 134.8, 129.4, 127.6, 127.4, 124.7, 124.1, 119.9, 117.7, 115.6, 115.1, 114.5, 114.5, 109.9, 105.0, 52.1, 47.7 ppm. IR (ATR): 3290, 3130, 2947, 1720, 1577, 1294, 1209, 982, 737 cm^–1^. HRMS: (ESI) *m*/*z*: [M + H]^+^ Calcd for C_19_H_17_N_2_O_3_ 321.1234, Found 321.1233. [α]^25^_D_ = +37.1° (c = 0.035, DMSO). Enantiomeric excess: (*E.r.*) 76:24; retention times t_major_ = 9.0 min and *t*_minor_ = 13.0 min determined by HPLC (Chiralpak column IC, 90/10 *n*-heptane/isopropanol, flow rate of 1.0 mL/min, 25 °C, λ = 195 nm).

#### (*S*)-4-Chloro-2-(4,5-dihydropyrrolo­[1,2-*a*]­quinoxalin-4-yl)­phenol (**3m**)

The compound was synthesized according to general procedure **GP4** (Method A), the product was obtained by column chromatography (silica, 5/1 hexane/EtOAc) as a white solid in 71% (21.1 mg) yield. ^1^H NMR: (400 MHz, CDCl_3_): δ 7.40–7.32 (m, 1H), 7.26–7.23 (m, 1H), 7.22 (dd, *J* = 3.2, 1.5 Hz, 1H), 7.12 (d, *J* = 2.6 Hz, 1H), 7.07–6.99 (m, 2H), 6.88 (dd, *J* = 9.0, 3.1 Hz, 2H), 6.26 (t, *J* = 3.2 Hz, 1H), 5.63 (dt, *J* = 3.5, 1.4 Hz, 1H), 5.51 (s, 1H) ppm. ^13^C­{^1^H} NMR: (101 MHz, CDCl_3_): δ 155.5, 134.3, 130.1, 129.4, 127.4, 127.1, 124.9, 124.5, 124.3, 122.4, 119.1, 117.4, 115.6, 115.3, 110.9, 106.9, 56.2 ppm. IR (ATR): 3280, 3064, 1608, 1520, 1477, 1244, 715 cm^–1^. HRMS: (ESI) *m*/*z*: [M + H]^+^ Calcd for C_17_H_13_^35^ClN_2_O 297.0789, Found 297.0788. [α]^25^_D_ = −11.4° (c = 0.35, MeOH). Enantiomeric excess: (*E.r.*) 92:8; retention times t_major_ = 92 min and *t*_minor_ = 8 min determined by HPLC (Chiralpak column IC, 90/10 *n*-heptane/isopropanol, flow rate of 1.0 mL/min, 25 °C, λ = 231 nm).

#### (*S*)-5-Chloro-2-(4,5-dihydropyrrolo­[1,2-*a*]­quinoxalin-4-yl)­phenol (**3n**)

The compound was synthesized according to general procedure **GP4** (Method A), the product was obtained by column chromatography (silica, 5/1 hexane/EtOAc) as a white solid in 95% (28.2 mg) yield. ^1^H NMR: (400 MHz, CDCl_3_) δ 8.53 (s, 1H), 7.40–7.33 (m, 1H), 7.21 (dd, *J* = 3.0, 1.5 Hz, 1H), 7.09–6.99 (m, 3H), 6.95 (d, *J* = 2.1 Hz, 1H), 6.89 (dt, *J* = 6.8, 2.2 Hz, 2H), 6.25 (t, *J* = 3.2 Hz, 1H), 5.59 (dt, *J* = 3.1, 1.4 Hz, 1H), 5.54 (s, 1H), 4.37 (s, 1H) ppm. ^13^C­{^1^H} NMR: (101 MHz, CDCl_3_): δ 157.7, 135.7, 134.4, 130.6, 127.7, 127.1, 124.9, 122.4, 121.4, 120.0, 118.1, 117.4, 115.6, 115.3, 110.9, 106.8, 56.3 ppm. IR (ATR): 3305, 3033, 2852, 1579, 1469, 1286, 1051, 891, 739 cm^–1^. HRMS: (ESI) *m*/*z*: [M + H]^+^ Calcd for C_17_H_14_^35^ClN_2_O 297.0776, Found 297.0789. [α]^25^_D_ = −48.6° (c = 0.35, MeOH). Enantiomeric excess: (*E.r.*) 91:9; retention times t_major_ = 4.7 min and *t*_minor_ = 8.2 min determined by HPLC (Chiralpak column IC, 90/10 *n*-heptane/isopropanol, flow rate of 1.0 mL/min, 25 °C, λ = 199 nm).

#### (*S*)-4-Bromo-2-(4,5-dihydropyrrolo­[1,2-*a*]­quinoxalin-4-yl)­phenol (**3o**)

The compound was synthesized according to general procedure **GP4** (Method A), the product was obtained by column chromatography (silica, 5/1 hexane/EtOAc) as a white solid in 64% (21.8 mg) yield. ^1^H NMR: (600 MHz, (CD_3_)­SO) δ 10.23 (s, 1H), 7.49 (d, *J* = 7.9 Hz, 1H), 7.42 (dd, *J* = 2.8, 1.6 Hz, 1H), 7.02 (d, *J* = 1.9 Hz, 1H), 6.95–6.88 (m, 3H), 6.85 (dd, *J* = 7.9, 1.5 Hz, 1H), 6.70 (td, *J* = 7.6, 1.5 Hz, 1H), 6.29 (s, 1H), 6.18 (t, *J* = 3.2 Hz, 1H), 5.80 (s, 1H), 5.71 (d, *J* = 3.9 Hz, 1H) ppm. ^13^C­{^1^H} NMR: (151 MHz, (CD_3_)­SO) δ 155.5, 136.4, 129.2, 129.1, 127.7, 124.6, 124.2, 121.7, 120.2, 117.7, 117.7, 115.1, 114.5, 114.4, 109.9, 104.9, 47.5 ppm. IR (ATR): 3275, 3043, 2871, 1606, 1520, 1475, 1244, 918, 739, 715 cm^–1^. HRMS: (ESI) *m*/*z*: [M + H]^+^ Calcd for C_17_H_14_^79^BrN_2_O 341.0271, Found 341.0285. [α]^25^_D_ = +4.3 ° (c = 0.035, MeOH). Enantiomeric excess: (*E.r.*) 91.9; retention times t_major_ = 4.8 min and *t*_minor_ = 8.0 min determined by HPLC (Chiralpak column IC, 90/10 *n*-heptane/isopropanol, flow rate of 1.0 mL/min, 25 °C, λ = 229 nm).

#### (*S*)-5-Bromo-2-(4,5-dihydropyrrolo­[1,2-*a*]­quinoxalin-4-yl)­phenol (**3p**)

The compound was synthesized according to general procedure **GP4** (Method A), the product was obtained by column chromatography (silica, 5/1 hexane/EtOAc) as a white solid in 64% (21.8 mg) yield. ^1^H NMR: (400 MHz, (CD_3_)­SO) δ 7.49 (dd, *J* = 8.0, 1.3 Hz, 1H), 7.42 (dd, *J* = 3.0, 1.6 Hz, 1H), 7.02 (d, *J* = 1.8 Hz, 1H), 6.95–6.89 (m, 2H), 6.89–6.82 (m, 2H), 6.70 (td, *J* = 7.5, 1.6 Hz, 1H), 6.30 (d, *J* = 1.9 Hz, 1H), 6.18 (t, *J* = 3.2 Hz, 1H), 5.80 (d, *J* = 1.7 Hz, 1H), 5.71 (dd, *J* = 3.7, 1.5 Hz, 1H) ppm. ^13^C­{^1^H} NMR: (101 MHz, (CD_3_)­SO) δ 155.5, 136.4, 129.2, 129.1, 127.7, 124.7, 124.2, 121.7, 120.2, 117.7, 117.7, 115.1, 114.5, 114.5, 109.9, 104.9, 47.5 ppm. IR (ATR): 3300, 3055, 2790, 1576, 1469, 1230, 1186, 895, 739 cm^–1^. HRMS: (ESI) *m*/*z*: [M + H]^+^ Calcd for C_17_H_14_^79^BrN_2_O 341.0271, Found 341.0285. [α]^25^_D_ = −35.7° (c = 0.35, MeOH). Enantiomeric excess: (*E.r.*) 90:10; retention times t_major_ = 4.9 min and *t*_minor_ = 9.1 min determined by HPLC (Chiralpak column IC, 90/10 *n*-heptane/isopropanol, flow rate of 1.0 mL/min, 25 °C, λ = 206 nm).

#### (S)-2-(4,5-Dihydropyrrolo­[1,2-*a*]­quinoxalin-4-yl)-4-fluorophenol (**3q**)

The compound was synthesized according to general procedure **GP4** (Method A), the product was obtained by column chromatography (silica, 5/1 hexane/EtOAc) as a white solid in 72% (21,2 mg) yield. ^1^H NMR: (600 MHz, (CD_3_)­SO) δ 9.75 (s, 1H), 7.50 (d, *J* = 7.9 Hz, 1H), 7.43 (t, *J* = 2.3 Hz, 1H), 6.94–6.89 (m, 2H), 6.87 (dd, *J* = 7.9, 1.5 Hz, 1H), 6.84 (dd, *J* = 8.9, 4.8 Hz, 1H), 6.72 (td, *J* = 6.6, 6.2, 2.8 Hz, 2H), 6.39–6.33 (m, 1H), 6.19 (t, *J* = 3.2 Hz, 1H), 5.82 (s, 1H), 5.74 (d, *J* = 3.4 Hz, 1H) ppm. ^13^C­{^1^H} NMR: (151 MHz, (CD_3_)­SO) δ 156.0, 154.5, 150.5, 136.4, 130.87 (d, *J* = 6.1 Hz), 127.6, 124.7, 124.2, 117.8, 116.07 (d, *J* = 7.7 Hz), 115.1, 114.51 (d, *J* = 7.3 Hz), 114.36 (d, *J* = 22.6 Hz), 113.42 (d, *J* = 23.5 Hz), 109.9, 105.0, 47.8 ppm. ^19^F NMR: (376 MHz, DMSO) δ −125.12 (m). IR (ATR): 3282, 3066, 2868, 1520, 1475, 1238, 926, 818, 741 cm^–1^. HRMS: (ESI) *m*/*z*: [M + H]^+^ Calcd for C_17_H_14_FN_2_O 281.1085, Found 281.1084. [α]^25^_D_ = −27.3° (c = 0.35, MeOH). Enantiomeric excess: (*E.r.*) 71:29; retention times t_major_ = 5.1 min and *t*_minor_ = 10.3 min determined by HPLC (Chiralpak column IC, 90/10 *n*-heptane/isopropanol, flow rate of 1.0 mL/min, 25 °C, λ = 230 nm).

#### (S)-2-(4,5-Dihydropyrrolo­[1,2-*a*]­quinoxalin-4-yl)-5-fluorophenol (**3r**)

The compound was synthesized according to general procedure **GP4** (Method A), the product was obtained by column chromatography (silica, 5/1 hexane/EtOAc) as a white solid in 65% (18.2 mg) yield. Crystal suitable for X-ray analysis was grown by the dissolution of **3r** in mixture of hexane/DCM (20:1), followed by standing at room temperature overnight. ^1^H NMR: (600 MHz, (CD_3_)­SO) δ 10.22 (s, 1H), 7.49 (d, *J* = 7.9 Hz, 1H), 7.42 (dd, *J* = 3.0, 1.6 Hz, 1H), 7.00 (dd, *J* = 8.6, 7.0 Hz, 1H), 6.94–6.88 (m, 1H), 6.86 (dd, *J* = 8.0, 1.5 Hz, 1H), 6.70 (td, *J* = 7.6, 1.5 Hz, 1H), 6.63 (dd, *J* = 10.8, 2.6 Hz, 1H), 6.54 (td, *J* = 8.6, 2.6 Hz, 1H), 6.27 (s, 1H), 6.17 (t, *J* = 3.2 Hz, 1H), 5.79 (s, 1H), 5.68 (d, *J* = 4.1 Hz, 1H) ppm. ^13^C­{^1^H} NMR: (151 MHz, (CD_3_)­SO) δ 162.5, 160.9, 155.7 (d, *J* = 11.0 Hz), 136.6, 128.8 (d, *J* = 10.2 Hz), 128.2, 125.8, 125.8, 124.6, 124.2, 117.7, 115.1, 114.4 (d, *J* = 19.9 Hz), 109.8, 105.4 (d, *J* = 21.2 Hz), 104.8, 102.1 (d, *J* = 23.6 Hz), 47.5 ppm. ^19^F NMR: (376 MHz, (CD_3_)­SO) δ −114.02 (ddd, *J* = 10.9, 8.6, 7.0 Hz) ppm. IR (ATR): 3282, 3066, 2868, 1520, 1475, 1336, 1238, 818 cm^–1^. HRMS: (ESI) *m*/*z*: [M + H]^+^ Calcd for C_17_H_14_FN_2_O 281.1085, Found 281.1085. [α]^25^_D_ = −47.1° (c = 0.35, MeOH). M.p.: 172–180 °C. Enantiomeric excess: (*E.r.*) 92:8; retention times t_major_ = 4.8 min and *t*_minor_ = 8.8 min determined by HPLC (Chiralpak column IC, 90/10 *n*-heptane/isopropanol, flow rate of 1.0 mL/min, 25 °C, λ = 203 nm).

#### (S)-3-(4,5-Dihydropyrrolo­[1,2-*a*]­quinoxalin-4-yl)­naphthalen-2-ol (**3s**)

The compound was synthesized according to general procedure **GP4**, the product was obtained by column chromatography (silica, 5/1 hexane/EtOAc) as a white solid in 96% (29.5 mg) yield (Method A) or using recrystallization from reaction crude (20/1 hexane/DCM) as a white crystals in 82% (25.6 mg) yield (Method B). ^1^H NMR: (400 MHz, CDCl_3_) δ 8.38 (s, 1H), 7.74 (t, *J* = 9.0 Hz, 2H), 7.63 (s, 1H), 7.46 (ddd, *J* = 8.2, 6.9, 1.3 Hz, 1H), 7.41–7.37 (m, 1H), 7.34 (ddd, *J* = 8.2, 6.8, 1.3 Hz, 1H), 7.30 (s, 1H), 7.24 (dd, *J* = 3.0, 1.6 Hz, 1H), 7.08–6.98 (m, 2H), 6.92–6.85 (m, 1H), 6.24 (t, *J* = 3.2 Hz, 1H), 5.76 (s, 1H), 5.56 (dt, *J* = 3.2, 1.4 Hz, 1H), 4.51 (s, 1H) ppm. ^13^C­{^1^H} NMR: (101 MHz, CDCl_3_): δ 154.4, 135.1, 134.5, 129.4, 128.1, 127.9, 127.8, 127.1, 126.9, 126.5, 125.1, 124.8, 123.8, 122.2, 117.3, 115.5, 115.3, 112.3, 110.9, 106.9, 56.9 ppm. IR (ATR): 3307, 3010, 1608, 1518, 1244, 816 cm^–1^. HRMS: (ESI) *m*/*z*: [M + H]^+^ Calcd for C_21_H_17_N_2_O 313.1335, Found 313.1337. [α]^25^_D_ = −4.3° (c = 0.35, MeOH). M.p.: 195–200 °C. Enantiomeric excess: (*E.r.*) 99:1; retention times t_major_ = 6.7 min and *t*_minor_ = 15.2 min determined by HPLC (Chiralpak column IC, 90/10 *n*-heptane/isopropanol, flow rate of 1.0 mL/min, 25 °C, λ = 226 nm).

#### (S)-4-(1H-Indol-2-yl)-4,5-dihydropyrrolo­[1,2-*a*]­quinoxaline (**3t**)

The compound was synthesized according to general procedure **GP4** (Method A), the product was obtained by column chromatography (silica, 5/1 hexane/EtOAc) as a white solid in 85% (24.3 mg) yield. ^1^H NMR: (600 MHz, (CD_3_)­SO) δ 11.04 (s, 1H), 7.50 (d, *J* = 7.9 Hz, 1H), 7.45 (dd, *J* = 2.9, 1.6 Hz, 1H), 7.43 (d, *J* = 7.9 Hz, 1H), 7.33 (d, *J* = 8.1 Hz, 1H), 7.03 (ddd, *J* = 8.1, 6.9, 1.2 Hz, 1H), 6.96–6.89 (m, 3H), 6.77–6.70 (m, 1H), 6.64 (d, *J* = 2.0 Hz, 1H), 6.21 (d, *J* = 2.7 Hz, 2H), 5.86 (dd, *J* = 3.6, 1.5 Hz, 1H), 5.78 (d, *J* = 1.8 Hz, 1H) ppm. ^13^C­{^1^H} NMR: (151 MHz, (CD_3_)­SO) δ 139.9, 136.3, 136.2, 127.4, 127.3, 124.6, 124.3, 120.8, 119.7, 118.8, 117.9, 115.2, 114.6, 114.6, 111.2, 109.8, 105.0, 99.3, 48.7 ppm. IR (ATR): 3392, 3055, 2852, 1612, 1456, 1336, 1132, 737 cm^–1^. HRMS: (ESI) *m*/*z* [M + H]^+^ Calcd for C_19_H_16_N_3_ 286.1339, Found 286.1338. [α]^25^_D_ = −5.7° (c = 0.35, MeOH). Enantiomeric excess: (*E.r.*) 85:15; retention times t_major_ = 7.0 min and *t*_minor_ = 8.6 min determined by HPLC (Chiralpak column IC, 90/10 *n*-heptane/isopropanol, flow rate of 1.0 mL/min, 25 °C, λ = 195 nm).

#### 1-Benzyl-5′H-spiro­[indoline-3,4’-pyrrolo­[1,2-*a*]­quinoxalin]-2-one (**3u**)

The compound was synthesized according to general procedure **GP4** (Method A), the product was obtained by column chromatography (silica, DCM) as a white solid in 92% (34.7 mg) yield known compound. ^1^H NMR: (400 MHz, CDCl_3_) δ 7.46 (dd, *J* = 7.4, 1.4 Hz, 1H), 7.40 (dd, *J* = 8.0, 1.4 Hz, 1H), 7.36–7.22 (m, 7H), 7.09 (td, *J* = 7.5, 1.0 Hz, 1H), 7.00 (td, *J* = 7.6, 1.4 Hz, 1H), 6.91 (td, *J* = 7.7, 1.4 Hz, 1H), 6.78 (dd, *J* = 7.8, 1.7 Hz, 2H), 6.26 (t, *J* = 3.2 Hz, 1H), 5.60 (dd, *J* = 3.5, 1.5 Hz, 1H), 4.97 (d, *J* = 15.6 Hz, 1H), 4.65 (dd, *J* = 15.6, 2.9 Hz, 1H), 4.08 (s, 1H) ppm. ^13^C­{^1^H} NMR: (101 MHz, CDCl_3_) δ 175.7, 143.3, 135.8, 134.2, 130.4, 129.8, 129.0, 127.9, 127.5, 126.0, 125.7, 125.5, 125.0, 123.6, 120.0, 116.2, 115.6, 114.6, 110.6, 109.6, 106.1, 106.0, 61.0, 43.8 ppm. IR (ATR): 3325, 3064, 1712, 1612, 1427, 1173, 744 cm^–1^. HRMS: (ESI) *m*/*z*: [M + Na]^+^ Calcd for C_25_H_19_N_3_ONa 400.1426, Found 400.1420. [α]^25^_D_ = +124° (c = 0.5, CHCl_3_). Enantiomeric excess: (*E.r.*) 85:15; retention times t_major_ = 24.2 min and *t*_minor_ = 29.8 min determined by HPLC (Chiralpak column IC, 95/05 *n*-heptane/isopropanol, flow rate of 1.0 mL/min, 25 °C, λ = 208 nm).

#### (S)-2-(9-Methyl-4,5-dihydropyrrolo­[1,2-*a*]­quinoxalin-4-yl)­phenol (**3y**)

The compound was synthesized according to general procedure **GP4** (Method A), the product was obtained by column chromatography (silica, 5/1 hexane/EtOAc) as a white solid in 74% (20.4 mg) yield. ^1^H NMR: (600 MHz, (CD_3_)­SO) δ 9.64 (s, 1H), 7.35 (dd, *J* = 3.0, 1.6 Hz, 1H), 7.09 (td, *J* = 7.6, 1.8 Hz, 1H), 7.06 (dd, *J* = 7.7, 1.8 Hz, 1H), 6.84 (dd, *J* = 15.1, 7.7 Hz, 2H), 6.80 (dd, *J* = 8.0, 1.7 Hz, 1H), 6.72 (t, *J* = 7.5 Hz, 1H), 6.64–6.60 (m, 1H), 6.17 (dd, *J* = 8.5, 5.3 Hz, 2H), 5.63–5.55 (m, 2H), 2.55 (s, 3H) ppm. ^13^C­{^1^H} NMR: (151 MHz, (CD_3_)­SO) δ 154.7, 139.4, 131.4, 128.2, 127.8, 127.7, 125.4, 124.7, 124.4, 122.1, 118.8, 118.4, 115.1, 114.0, 108.8, 103.5, 48.2, 20.9 ppm. IR (ATR): 3290, 2954, 1587, 1479, 1147, 752 cm^–1^. HRMS: (ESI) *m*/*z*: [M + H]^+^ Calcd for C_18_H_17_N_2_O 277.1335, Found 277.1333. [α]^25^_D_ = −20° (c = 0.35, MeOH). Enantiomeric excess: (*E.r.*) 63:37; retention times t_major_ = 5.5 min and *t*_minor_ = 13.0 min determined by HPLC (Chiralpak column IC, 90/10 *n*-heptane/isopropanol, flow rate of 1.0 mL/min, 25 °C, λ = 229 nm).

#### (*S*)-2-(9-Methoxy-4,5-dihydropyrrolo­[1,2-*a*]­quinoxalin-4-yl)­phenol (**3z**)

The compound was synthesized according to general procedure **GP4** (Method A), the product was obtained by column chromatography (silica, 5/1 hexane/EtOAc) as a white solid in 94% (27.5 mg) yield. ^1^H NMR: (400 MHz, CDCl_3_) δ 8.23 (s, 1H), 7.83 (dd, *J* = 3.3, 1.6 Hz, 1H), 7.30 (ddd, *J* = 8.2, 7.4, 1.7 Hz, 1H), 7.14 (dd, *J* = 7.5, 1.7 Hz, 1H), 7.01–6.93 (m, 2H), 6.90 (td, *J* = 7.4, 1.3 Hz, 1H), 6.67 (dd, *J* = 8.4, 1.2 Hz, 1H), 6.52 (dd, *J* = 8.0, 1.2 Hz, 1H), 6.19 (t, *J* = 3.3 Hz, 1H), 5.55 (dt, *J* = 3.5, 1.4 Hz, 1H), 5.46 (s, 1H), 4.39 (s, 1H), 3.96 (s, 3H) ppm. ^13^C­{^1^H} NMR: (101 MHz, CDCl_3_): δ 156.9, 150.3, 137.1, 130.2, 129.9, 128.6, 124.7, 122.8, 121.5, 119.7, 117.6, 117.3, 110.2, 109.0, 106.0, 105.3, 56.6, 56.1 ppm. IR (ATR): 3282, 3060, 1591, 1469, 1242, 1186, 750 cm^–1^. HRMS: (ESI) *m*/*z*: [M + H]^+^ Calcd for C_18_H_17_N_2_O_2_ 293.1285, Found 293.1283. [α]^25^_D_ = −20° (c = 0.035, MeOH). Enantiomeric excess: (*E.r.*) 87:13; retention times t_major_ = 6.9 min and *t*_minor_ = 20.0 min determined by HPLC (Chiralpak column IC, 90/10 *n*-heptane/isopropanol, flow rate of 1.0 mL/min, 25 °C, λ = 232 nm).

#### (S)-2-(8-Methoxy-4,5-dihydropyrrolo­[1,2-*a*]­quinoxalin-4-yl)­phenol (**3aa**)

The compound was synthesized according to general procedure **GP4** (Method A), the product was obtained by column chromatography (silica, 5/1 hexane/EtOAc) as a white solid in 67% (19.6 mg) yield. ^1^H NMR: (400 MHz, CDCl_3_) δ 8.70 (s, 1H), 7.29 (td, *J* = 7.8, 1.8 Hz, 1H), 7.17 (dd, *J* = 3.1, 1.6 Hz, 1H), 7.11 (dd, *J* = 7.5, 1.7 Hz, 1H), 6.95–6.91 (m, 2H), 6.89 (td, *J* = 7.4, 1.2 Hz, 1H), 6.82 (d, *J* = 8.6 Hz, 1H), 6.60 (dd, *J* = 8.6, 2.6 Hz, 1H), 6.24 (t, *J* = 3.2 Hz, 1H), 5.59 (dt, *J* = 3.2, 1.4 Hz, 1H), 5.49 (s, 1H), 4.22 (s, 1H), 3.83 (s, 3H) ppm. ^13^C­{^1^H} NMR: (101 MHz, CDCl_3_): δ 157.1, 155.5, 130.2, 129.7, 128.7, 128.1, 128.0, 122.7, 119.7, 118.2, 117.7, 115.5, 110.9, 109.6, 109.6, 106.8, 102.2, 56.9, 55.9 ppm. IR (ATR): 3292, 2993, 2833, 1622, 1489, 1236, 754 cm^–1^. HRMS: (ESI) *m*/*z* [M + H]^+^ Calcd for C_16_H_15_N_5_O 293.1271, Found 293.1281. [α]^25^_D_ = −20° (c = 0.35, MeOH). Enantiomeric excess: (*E.r.*) 84:16; retention times t_major_ = 6.0 min and *t*_minor_ = 15.5 min determined by HPLC (Chiralpak column IC, 90/10 *n*-heptane/isopropanol, flow rate of 1.0 mL/min, 25 °C, λ = 228 nm).

#### (*S*)-2-(8-Chloro-4,5-dihydropyrrolo­[1,2-*a*]­quinoxalin-4-yl)­phenol (**3bb**)

The compound was synthesized according to general procedure **GP4** (Method A), he product was obtained by column chromatography (silica, 5/1 hexane/EtOAc) as a white solid in 98% (29.1 mg) yield. ^1^H NMR: (600 MHz, (CD_3_)­SO) δ 9.71 (s, 1H), 7.63 (d, *J* = 2.4 Hz, 1H), 7.53–7.46 (m, 1H), 7.11–7.03 (m, 1H), 6.96 (dd, *J* = 7.8, 1.7 Hz, 1H), 6.92 (dd, *J* = 8.5, 2.3 Hz, 1H), 6.85 (dd, *J* = 8.5, 2.1 Hz, 2H), 6.69 (t, *J* = 7.5 Hz, 1H), 6.48 (s, 1H), 6.19 (t, *J* = 3.2 Hz, 1H), 5.86 (s, 1H), 5.73 (d, *J* = 3.3 Hz, 1H), 3.32 (s, 3H) ppm. ^13^C­{^1^H} NMR: (151 MHz, (CD_3_)­SO) δ 154.2, 135.6, 129.1, 128.3, 128.2, 127.3, 125.0, 124.1, 120.8, 118.9, 116.1, 115.2, 114.9, 114.4, 110.4, 105.2, 47.6 ppm. IR (ATR): 3371, 3060, 2866, 1591, 1510, 1319, 1242, 1097, 883, 750 cm^–1^. HRMS: (ESI) *m*/*z* [M + H]^+^ Calcd for C_16_H_15_N_5_O 293.1271, Found 293.1281. [α]^25^_D_ = −37.1° (c = 0.35, MeOH). Enantiomeric excess: (*E.r.*) 74:26; retention times t_major_ = 4.5 min and *t*_minor_ = 8.0 min determined by HPLC (Chiralpak column IC, 90/10 *n*-heptane/isopropanol, flow rate of 1.0 mL/min, 25 °C, λ = 237 nm).

#### (S)-2-(7-Bromo-4,5-dihydropyrrolo­[1,2-*a*]­quinoxalin-4-yl)­phenol (**3cc**)

The compound was synthesized according to general procedure **GP4** (Method A), the product was obtained by column chromatography (silica, 5/1 hexane/EtOAc) as a white solid in 72% (24.6 mg) yield. ^1^H NMR: (400 MHz, CDCl_3_) δ 7.33 (td, *J* = 7.8, 1.7 Hz, 1H), 7.24 (d, *J* = 8.5 Hz, 1H), 7.19 (dd, *J* = 3.1, 1.5 Hz, 1H), 7.17–7.09 (m, 3H), 7.02 (d, *J* = 2.0 Hz, 1H), 7.00–6.89 (m, 2H), 6.29 (t, *J* = 3.2 Hz, 1H), 5.65 (dt, *J* = 3.3, 1.3 Hz, 1H), 5.60 (s, 1H) ppm. ^13^C­{^1^H} NMR: (101 MHz, CDCl_3_): δ 156.3, 136.2, 130.4, 129.7, 129.5, 127.8, 125.9, 124.4, 124.2, 123.0, 120.5, 120.5, 120.4, 119.7, 117.6, 117.2, 116.4, 115.4, 111.2, 107.0, 55.8 ppm. IR (ATR): 3341, 3002, 2753, 1591, 1512, 1319, 1247, 1020, 856, 750 cm^–1^. HRMS: (ESI) *m*/*z*: [M + H]^+^ Calcd for C_17_H_14_^79^BrN_2_O 341.0284, Found 341.0284. [α]^25^_D_ = +7.3° (c = 0.035, DMSO). Enantiomeric excess: (*E.r.*) 64:36; retention times t_major_ = 5.0 min and *t*_minor_ = 7.3 min determined by HPLC (Chiralpak column IC, 90/10 *n*-heptane/isopropanol, flow rate of 1.0 mL/min, 25 °C, λ = 235 nm).

#### (S)-2-(7-(Trifluoromethyl)-4,5-dihydropyrrolo­[1,2-*a*]­quinoxalin-4-yl)­phenol (**3dd**)

The compound was synthesized according to general procedure **GP4** (Method A), the product was obtained by column chromatography (silica, 5/1 hexane/EtOAc) as a white solid in 25% (8.3 mg) yield. ^1^H NMR: (400 MHz, CDCl_3_) δ 7.56 (s, 1H), 7.42 (d, *J* = 8.3 Hz, 1H), 7.31 (td, *J* = 7.8, 1.7 Hz, 1H), 7.26–7.22 (m, 2H), 7.13 (dd, *J* = 7.5, 1.7 Hz, 1H), 7.09 (d, *J* = 1.9 Hz, 1H), 6.93 (qd, *J* = 7.9, 7.4, 1.2 Hz, 2H), 6.30 (t, *J* = 3.2 Hz, 1H), 5.67 (dd, *J* = 3.4, 1.5 Hz, 1H), 5.61 (s, 1H), 4.58 (s, 1H) ppm. ^13^C­{^1^H} NMR: (101 MHz, CDCl_3_): δ 156.3, 135.1, 130.5, 129.8, 129.2, 128.2, 127.0, 126.6, 125.4, 123.8, 122.7, 122.7, 120.2, 118.72 (t, *J* = 4.0 Hz), 117.7, 115.7, 115.2, 113.90 (t, *J* = 3.9 Hz), 111.9, 111.2, 107.6, 56.0 ppm. ^19^F NMR: (376 MHz, CDCl_3_) δ −62.28 ppm. IR (ATR): 3292, 3099, 2860, 1591, 1473, 1331, 1163, 1111, 808 cm^–1^. HRMS: (ESI) *m*/*z*: [M + H]^+^ Calcd for C_18_H_14_F_3_N_2_O 331.1053, Found 331.1052. [α]^25^_D_ = +5.7° (c = 0.035, DMSO). Enantiomeric excess: (*E.r.*) 63:37; retention times t_major_ = 4.6 min and *t*_minor_ = 4.0 min determined by HPLC (Chiralpak column IC, 90/10 *n*-heptane/isopropanol, flow rate of 1.0 mL/min, 25 °C, λ = 238 nm).

### Gram Scale Pictet–Spengler Reaction

#### (S)-2-(4,5-Dihydropyrrolo­[1,2-*a*]­quinoxalin-4-yl)­phenol (**3c**)

To the mixture of substituted aniline **1** (6.32 mmol, 1g, 1 equiv) and **C1** (10 mol %, 0,63 mmol, 618 mg) in 16 mL of toluene in the presence of molecular sieves (5Å) at −55 °C, substituted salicylaldehyde **2** (7.59 mmol, 0.926 mg, 0.12 equiv), dissolved in 16 mL, was added (0.2 M). The mixture was stirred until full conversion and after that the mixture was filtered through cotton, solvent was evaporated and reaction mixture was crystallized from hexane/DCM (20:1) to yield the final product **3c** as a white crystals in 90% (1.49 g) yield. All analytical data matched the data of identical compound prepared on a smaller scale.

### Further Transformation Products

#### (S)-2-(5-Acetyl-4,5-dihydropyrrolo­[1,2-*a*]­quinoxalin-4-yl)­phenyl Acetate (**4**)

The compound **3c** (50 mg, 0.19 mmol, 1 equiv) and Ac_2_O (78 mg, 0.76 mmol, 4 equiv) was stirred in the presence of NaHCO_3_ (64 mg, 0.76 mmol, 4 equiv) in DCM (4 mL). After full conversion of starting material the reaction crude was filtered through cotton and evaporated. The product was obtained by column chromatography (silica, 5/1 hexane/EtOAc) as a white solid in 68% (44.9 mg) yield. ^1^H NMR: (400 MHz, CDCl_3_) δ 7.49–7.41 (m, 2H), 7.27 (dd, *J* = 3.0, 1.4 Hz, 1H), 7.24 (dd, *J* = 7.9, 1.5 Hz, 1H), 7.15 (td, *J* = 7.7, 1.6 Hz, 1H), 7.09 (d, *J* = 7.9 Hz, 1H), 7.02 (td, *J* = 7.7, 1.3 Hz, 1H), 6.95 (dd, *J* = 8.0, 1.3 Hz, 1H), 6.83 (td, *J* = 7.6, 1.3 Hz, 1H), 6.40 (t, *J* = 3.2 Hz, 1H), 6.33–6.25 (m, 1H), 6.21 (dd, *J* = 3.6, 1.4 Hz, 1H), 2.42 (s, 3H), 2.12 (s, 3H) ppm. ^13^C­{^1^H} NMR: (101 MHz, CDCl_3_): δ 170.4, 168.8, 148.7, 131.5, 131.5, 129.2, 129.2, 128.5, 127.9, 127.4, 127.3, 125.8, 124.3, 123.0, 115.8, 114.8, 111.1, 107.3, 47.3, 22.4, 21.5 ppm. IR (ATR): 3411, 2922, 1765, 1660, 1504, 1196, 1039, 750 cm^–1^. HRMS: (ESI) *m*/*z*: [M + Na]^+^ Calcd for C_21_H_18_N_2_O_3_Na 369.1215, Found 369.1216. [α]^25^_D_ = +154° (c = 0.1, DMSO). Enantiomeric excess: (*E.r.*) 99:1; retention times t_major_ = 9.1 min and *t*_minor_ = 10.6 min determined by HPLC (Chiralpak column IC, 90/10 *n*-heptane/isopropanol, flow rate of 1.0 mL/min, 25 °C, λ = 233 nm).

#### (S)-2-(5-Acetyl-1-bromo-4,5-dihydropyrrolo­[1,2-*a*]­quinoxalin-4-yl)­phenyl Acetate (**5**)

The compound **4** (50 mg, 0.14 mmol, 1 equiv), NBS (26 mg, 0.14 mmol, 1 equiv) was stirred in the presence of DMSO (50 mol %) in DCM (4 mL). After full conversion the reaction crude was evaporated. The product was obtained by column chromatography (silica, 5/1 hexane/EtOAc) as a white solid in 78% (33.1 mg) yield. ^1^H NMR: (400 MHz, CDCl_3_) δ 8.22 (d, *J* = 8.3 Hz, 1H), 7.36 (s, 1H), 7.31–7.22 (m, 1H), 7.15 (td, *J* = 7.7, 1.7 Hz, 1H), 7.07 (d, *J* = 4.0 Hz, 2H), 6.93 (dd, *J* = 8.0, 1.2 Hz, 1H), 6.83 (td, *J* = 7.6, 1.3 Hz, 1H), 6.46–6.38 (m, 2H), 6.21 (d, *J* = 3.7 Hz, 1H), 2.40 (s, 3H), 2.16 (s, 3H) ppm. ^13^C­{^1^H} NMR: (101 MHz, CDCl_3_) δ 170.4, 168.2, 148.8, 131.9, 131.3, 130.4, 129.5, 129.4, 129.2, 127.7, 127.0, 125.7, 125.6, 123.1, 119.3, 115.4, 107.9, 97.2, 48.2, 22.3, 21.5 ppm. IR (ATR): 3383, 2958, 1666, 1404, 1196, 1018, 914 cm^–1^. HRMS: (ESI) *m*/*z*: [M + Na]^+^ Calcd for C_21_H_17_^79^BrN_2_O_3_Na 447.0320, Found 447.0317. [α]^25^_D_ = +211.4° (c = 0.035, DMSO). Enantiomeric excess: (*E.r.*) 95:5; retention times t_major_ = 10.3 min and *t*_minor_ = 8.9 min determined by HPLC (Chiralpak column IC, 90/10 *n*-heptane/isopropanol, flow rate of 1.0 mL/min, 25 °C, λ = 233 nm).

## Supplementary Material







## Data Availability

The data underlying this study are available in the published article and in its .

## Notes

Experimental procedures and spectral data for all prepared compounds with copies of the ^1^H NMR, ^13^C NMR, ^19^F NMR, HPLC chromatographs, crystallographic data and Cartesian coordinates of the key transition states. (PDF)
FAIR data, including the primary NMR FID files, for compounds **SM1**–**SM3**, **PCCP**, **C1**–**C5**, **1b**–**h**, **2i**, **2s**, **3b**–**3z**, **3aa**–**3dd**, **4** and **5** (ZIP)
Cartesian coordinates of the key transition states II leading to both enantiomers (XYZ)

## References

[1] Xu H., Fan L.-L. (2011). Synthesis and antifungal activities of novel 5,6-dihydro-indolo­[1,2-a]­quinoxaline
derivatives. Eur. J. Med. Chem..

[2] Lin P. T., Salunke D. B., Chen L.-H., Sun C.-M. (2011). Soluble polymer
supported divergent synthesis of tetracyclic benzene-fused pyrazino/diazepinoindoles:
an advanced synthetic approach to bioactive scaffolds. Org. Biomol. Chem..

[3] Szabó G., Kiss R., Páyer-Lengyel D., Vukics K., Szikra J., Baki A., Molnár L., Fischer J., Keserü G. M. (2009). Hit-to-lead
optimization of pyrrolo­[1,2-a]­quinoxalines
as novel cannabinoid type 1 receptor antagonists. Bioorg. Med. Chem. Lett..

[4] Sabatucci, J. P. ; Ye, F. ; Mahaney, P. E. U.S. Patent 0160815, 2006.

[5] Strittmatter, S. M. ; Gunther, E. W.O. Patent 073141, 2009.

[6] Fan L.-L., Huang N., Yang R.-G., He S.-Z., Yang L.-M., Xu H., Zheng Y.-T. (2012). Discovery of 5,6-Dihydro-indolo­[1,2-a]­quinoxaline Derivatives
as New HIV-1 Inhibitors In Vitro. Lett. Drug
Des. Disc..

[7] Tradtrantip L., Sonawane N. D., Namkung W., Verkman A. S. (2009). Nanomolar Potency
Pyrimido-pyrrolo-quinoxalinedione CFTR Inhibitor Reduces Cyst Size
in a Polycystic Kidney Disease Model. J. Med.
Chem..

[8] Kamal A., Babu K. S., Hussaini S. M. A., Srikanth P. S., Balakrishna M., Alarifi A. (2015). Sulfamic acid: an efficient
and recyclable
solid acid catalyst for the synthesis of 4,5-dihydropyrrolo­[1,2-a]­quinoxalines. Tetrahedron Lett..

[9] Verma A. K., Jha R. R., Sankar V. K., Aggarwal T., Singh R. P., Chandra R. (2011). Lewis Acid-Catalyzed
Selective Synthesis of Diversely
Substituted Indolo- and Pyrrolo­[1,2-a]­quinoxalines and Quinoxalinones
by Modified Pictet–Spengler Reaction. Eur. J. Org. Chem..

[10] Alizadeh A., Mokhtari J. (2013). Synthesis of spiro­[indoline-3,4′-pyrrolo­[1,2-a]­quinoxalin]-2-one
catalyzed by molecular iodine. J. Tetrahedron.

[11] Li Y., Su Y.-H., Dong D.-J., Wu Z., Tian S.-K. (2013). Chiral
boron Lewis acid-catalyzed asymmetric synthesis of 4,5-dihydropyrrolo­[1,2-a]­quinoxalines. RSC Adv..

[12] Fan Y.-S., Jiang Y.-J., An D., Sha D., Antilla J. C., Zhang S. (2014). H8-BINOL Chiral Imidodiphosphoric
Acids Catalyzed Enantioselective
Synthesis of Dihydroindolo–/–pyrrolo­[1,2-a]­quinoxalines. Org. Lett..

[13] Shen X., Wang Y., Wu T., Mao Z., Lin X. (2015). Triply Hydrogen-Bond-Directed
Enantioselective Assembly of Pyrrolobenzo-1,4-diazine Skeletons with
Quaternary Stereocenters. Chem.Eur. J..

[14] Wang Y., Cui L., Wang Y., Zhou Z. (2016). Stereocontrolled construction of
4,5-dihydropyrrolo­[1,2-a]­quinoxaline scaffolds via chiral phosphoramidate
catalyzed Pictet–Spengler-type reaction. Tetrahedron: Asymmetry.

[15] Hu S.-B., Zhai X.-Y., Shen H.-Q., Zhou Y.-G. (2018). Iridium-catalyzed
Asymmetric Hydrogenation of Polycyclic Pyrrolo/Indolo­[1,2-a]­quinoxalines
and Phenanthridines. Adv. Synth. Catal..

[16] Guo Q., Xie C., Zi G., Lai X., Deerberg J., Hou G. (2024). Ir-Catalyzed Asymmetric Hydrogenation of N-Fused Heteroarenes with
High Nitrogen Density: An Access to Chiral 2,5-Disubstituted 5,6-Dihydropyrrolo­[1,2-a]­[1,2,4]­triazolo­[5,1-c]­pyrazines. Org. Lett..

[17] Smith S. W. (2009). Chiral
toxicology: it’s the same thing···only different. Toxicol. Sci..

[18] Gheewala C. D., Collins B. E., Lambert T. H. (2016). An aromatic ion platform for enantioselective
Brønsted acid catalysis. Science.

[19] Gheewala C. D., Hirschi J. S., Lee W.-H., Paley D. W., Vetticatt M. J., Lambert T. H. (2018). Asymmetric Induction via a Helically Chiral Anion:
Enantioselective Pentacarboxycyclopentadiene Brønsted Acid-Catalyzed
Inverse-Electron-Demand Diels–Alder Cycloaddition of Oxocarbenium
Ions. J. Am. Chem. Soc..

[20] Sui Y., Cui P., Liu S., Zhou Y., Du P., Zhou H. (2018). Highly Enantioselective
Synthesis of Cyclic Aminals with a Cyclopentadiene-Based Chiral Carboxylic
Acid. Eur. J. Org. Chem..

[21] Yuan C., Li J., Li P. (2018). Chiral Pentacarboxycyclopentadiene-Based
Brønsted
Acid-Catalyzed Enantioselective Desymmetrization of Meso-Epoxides
by 2-Mercaptobenzothiazoles. ACS Omega.

[22] Kang Z., Wang Y., Zhang D., Wu R., Xu X., Hu W. (2019). Asymmetric Counter-Anion-Directed Aminomethylation: Synthesis of
Chiral β-Amino Acids via Trapping of an Enol Intermediate. J. Am. Chem. Soc..

[23] Li J., An S., Yuan C., Li P. (2019). Enantioselective Protonation of Silyl
Enol Ethers Catalyzed by a Chiral Pentacarboxycyclopentadiene-Based
Brønsted Acid. Synlett.

[24] Kamlar M., Reiberger R., Nigríni M., Císařová I., Veselý J. (2021). Enantioselective PCCP Brønsted acid-catalyzed
aminalization of aldehydes. Beilstein J. Org.
Chem..

[25] Bhosale V. A., Nigrini M., Dracinsky M., Cisarova I., Vesely J. (2021). Enantioselective
Desymmetrization of 3-Substituted Oxetanes: An Efficient Access to
Chiral 3,4-Dihydro-2H-1,4-benzoxazines. Org.
Lett..

[26] Nigrini M., Bhosale V. A., Cisarova I., Vesely J. (2023). Enantioenriched 1,4-Benzoxazepines
via Chiral Brønsted Acid-Catalyzed Enantioselective Desymmetrization
of 3-Substituted Oxetanes. J. Org. Chem..

[27] Ahn J., Lee S. B., Song I., Chun S., Oh D.-C., Hong S. (2021). Synthesis of 4-Aryl
Pyrrolo [1,2-α]­quinoxalines via Iron-Catalyzed
Oxidative Coupling from an Unactivated Methyl Arene. J. Org. Chem..

[28] García-Marín J., Griera M., Sánchez-Alonso P., Di Geronimo B., Mendicuti F., Rodríguez-Puyol M., Alajarín R., de Pascual-Teresa B., Vaquero J. J., Rodríguez-Puyol D. (2020). Pyrrolo­[1,2-a]­quinoxalines:
Insulin Mimetics that Exhibit Potent and Selective Inhibition against
Protein Tyrosine Phosphatase 1B. Chem. Med.
Chem..

[29] Chiurato M., Routier S., Troin Y., Guillaumet G. (2009). New Efficient
Route to Fused Aryltetrahydroindolizinones via N-Acyliminium Intermediates. Eur. J. Org. Chem..

[30] Doshi J. M., Das S. G., Tian D., Addo S. N., Srinivasan B., Hermanson D. L., Xing C. (2009). Structure-Activity Relationship and
Molecular Mechanisms of Ethyl 2-Amino-4-(2-Ethoxy-2-Oxoethyl)-6-Phenyl-4H-Chromene-3-Carboxylate
(sHA 14–1) and Its Analogues. J. Med.
Chem..

[31] Chai J.-D., Head-Gordon M. (2008). Long-range corrected hybrid density functionals with
damped atom-atom dispersion corrections. Phys.
Chem. Chem. Phys..

[32] Weigend F. (2006). Accurate Coulomb-fitting
basis sets for H to Rn. Phys. Chem. Chem. Phys..

[33] Gaussian 16, Revision C.01; Gaussian, Inc., Wallingford CT, 2019.

[34] Lee K., Kim J. H., Kim W. Y. (2023). pyMCD:
Python package for searching
transition states via the multicoordinate driven method. Comput. Phys. Commun..

