# Supplementary material for: Enantioselective Pictet–Spengler-Type Reaction via a Helically Chiral Anion as an Access to 4,5-Dihydropyrrolo[1,2‑a]quinoxaline Scaffolds
Source: J Org Chem. 2025 Jul 1;90(27):9380–93. doi: 10.1021/acs.joc.5c00638 (PMC12261325; doi:10.1021/acs.joc.5c00638)
Supplement: Supplementary file 1 [file jo5c00638_si_001.pdf]

Enantioselective Pictet-Spengler-type Reaction via a Helically Chiral Anion as an Access to 4,5-Dihydropyrrolo[1,2-*a*] quinoxaline Scaffolds.

Martin Nigríni,[a] *Filip Uhlík*,[b] *Ivana Císařová*,[c] and *Jan Veselý* [a],*

[a] Department of Organic Chemistry, Faculty of Science, Charles University, Hlavova 2030, 128 43 Prague, Czech Republic.

[b] Department of physical and macromolecular chemistry, Faculty of Science, Charles University, Hlavova 2030, 128 43 Prague, Czech Republic.

[c] Department of Inorganic Chemistry, Faculty of Science, Charles University, Hlavova 2030, 128 43 Prague, Czech Republic.

Corresponding author Email: jan.vesely@natur.cuni.cz.

Supporting Information

X-Ray Crystallography S4

Quantum Chemistry Calculations S7

Cartesian Coordinates S9

^{1}H, ^{13}C, ^{19}F NMR DATA: S23

HPLC DATA: S118

Table S1: Solvent screening of enantioselective Pictet-Spengler reaction.

Solvent screening

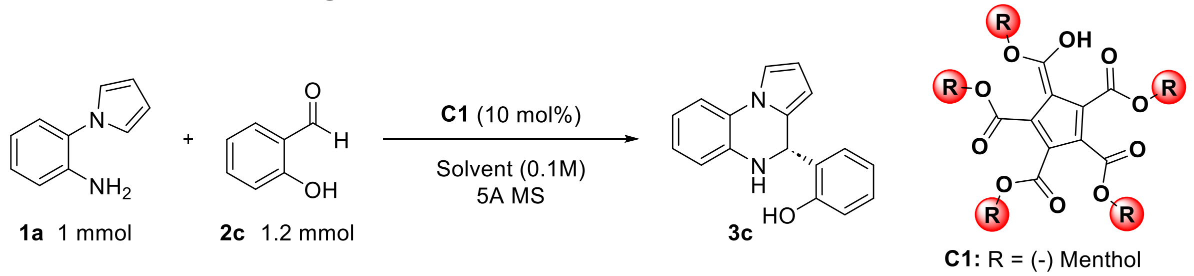

Entry	Solvent	Temperature [°C]	Time [h]	Yield 3c [%][b]	*E.e.* [%][c]
1	DCM	25	5	93	5
2	Toluene	25	5	94	60
3	Benzene	25	5	93	46
4	MTBE	25	5	90	12
5	Benzene/Furan (7:3)	-20	15	94	65
6	DCM	-45	24	93	27
7	Toluene	-45	24	94	82
8	Et_2O	-45	24	93	3
9	EtOAc	-45	24	94	5
10	$CHCl_3$	-45	24	94	0
11	THF	-45	24	94	-8
12	MTBE	-45	24	90	28
13	Toluene	-55	24	90	85
14	Toluene	-65	24	90	91
15	Toluene	-78	24	94	93
16	Toluen (0.2M)	-78	48	85	49
17	Toluene (0.05M)	-78	24	94	93
18	Toluene (0.05M)	-65	24	94	93
**19**	**Toluene (0.05M)**	**-55**	**18**	**94**	**93**
20	Toluene (0.05M)	-45	15	94	91

[a] Determined by ^{1}H NMR of crude reaction mixture. [b] Isolated yields after column chtomathography. [c] IC column (heptane/*iso*-propanol, 90:10, 1 ml/min).

Table S2: Catalyst loading of enantioselective Pictet-Spengler reaction.

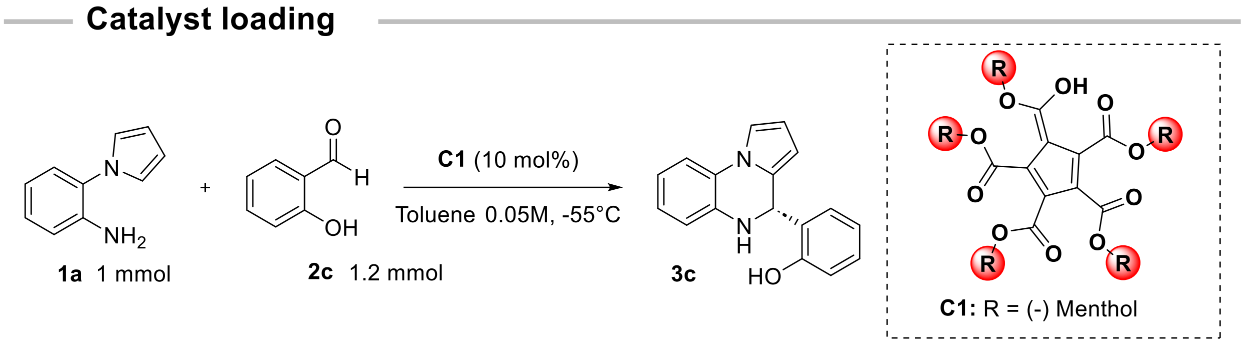

Entry	C1 mol%	Time [h]	Yield 3 [%][b]	*E.r.* [%][c]
1	20	18	92	95:5
2	15	18	93	95:5
3	10	18	94	97:3
4	5	48	72	89:11
5	2.5	48	68	70:30
6	1	72	30	51:49

[a)] Determined by ^{1}H NMR of crude reaction mixture. [b)] Isolated yields after column chtomathography. [c)] IC column (heptane/*iso*-propanol, 90:10, 1 ml/min).

X-Ray Crystallography

Crystalographic data for **3c** and **3r** were obtained on Bruker D8 VENTURE Kappa Duo PHOTONIII by IµS micro-focus sealed tube CuKα (λ= 1.54178 Å). The structures were solved by direct methods (XT) [1a] and refined by full matrix least squares based on F^2 (SHELXL2019) [1b]. The hydrogen atoms on carbon were fixed into idealized positions (riding model) and assigned temperature factors $H_{iso}(H) = 1.2\ U_{eq}$(pivot atom). The hydrogen atoms in –OH and >N-H moieties were found on difference Fourier map and refined under rigid-body assumption with assigned temperature factor $H_{iso}(H) = 1.2\ U_{eq}$(pivot atoms)). The determination of absolute structure was based of anomalous dispersion of oxygen, nitrogen and fluorine atoms.

Crystal data for **3c,** $C_{17}H_{14}N_2O$, M_r = 262.30; Monoclinic, $P2_1$ (No 4), a = 4.9621 (2) Å, b = 11.6169 (5) Å, c = 11.5674 (5) Å, β = 100.953 (2)° , V = 654.65 (5) Å^3, Z = 2, D_x = 1.331 Mg m^{-3}, temperature of sample 120(2) K, colourless plate of dimensions 0.43 × 0.13 × 0.04 mm, multi-scan absorption correction (µ = 0.67 mm^{-1}), T_{min} = 0.74, T_{max} = 0.97; a total of 8362 measured reflections (θ_{max}= = 68.2˚), from which 2370 were unique (R_{int} = 0.031) and 2327 observed according to the $I > 2\sigma(I)$ criterion. The refinement converged ($\Delta/\sigma_{max} < 0.001$) to R = 0.034 for observed reflections and w$R(F^2)$ = 0.090, GOF = 1.06 for 181 parameters and all 2370 reflections. The final difference map displayed no peaks of chemical significance ($\Delta\rho_{max}$ = 0.14, $\Delta\rho_{min}$ -0.20 e.Å^{-3}). Absolute structure parameter: 0.0(2)[2]

Crystal data for **3r,** $C_{17}H_{13}FN_2O$, M_r = 280.29; Orthorhombic, $P2_1\,2_1\,2_1$ (No 19), a = 5.0338 (3) Å, b = 12.4459 (7) Å, c = 20.9918 (12) Å, V = 1315.14 (13) Å^3, Z = 4, D_x = 1.416 Mg m^{-3}, temperature of sample 100(2) K, colourless prism of dimensions 0.28 × 0.27 × 0.14 mm, multi-scan absorption correction (µ = 0.82 mm^{-1}), T_{min} = 0.71, T_{max} = 0.90; a total of 21922 measured reflections (θ_{max}= = 79.5˚), from which 2826 were unique (R_{int} = 0.033) and 2791 observed according to the $I > 2\sigma(I)$ criterion. The refinement converged ($\Delta/\sigma_{max} < 0.001$) to R = 0.029 for observed reflections and w$R(F^2)$ = 0.0078, GOF = 1.08 for 190 parameters and all 2826 reflections. The final difference map displayed no peaks of chemical significance ($\Delta\rho_{max}$ = 0.16, $\Delta\rho_{min}$ -0.18 e.Å^{-3}). Absolute structure parameter: 0.08 (5)[2]

X-ray crystallographic data have been deposited with the Cambridge Crystallographic Data Centre under deposition numbers CCDC 2422802 and 2428515, for 3**c** and **3r**, respectively. They can be obtained free of charge from the Centre via its website (https://www.ccdc.cam.ac.uk/structures/).

Figure S1. View on molecule of **3c** with atom numbering schema displaying. Displacement ellipsoids are drawn on 30% probability level.

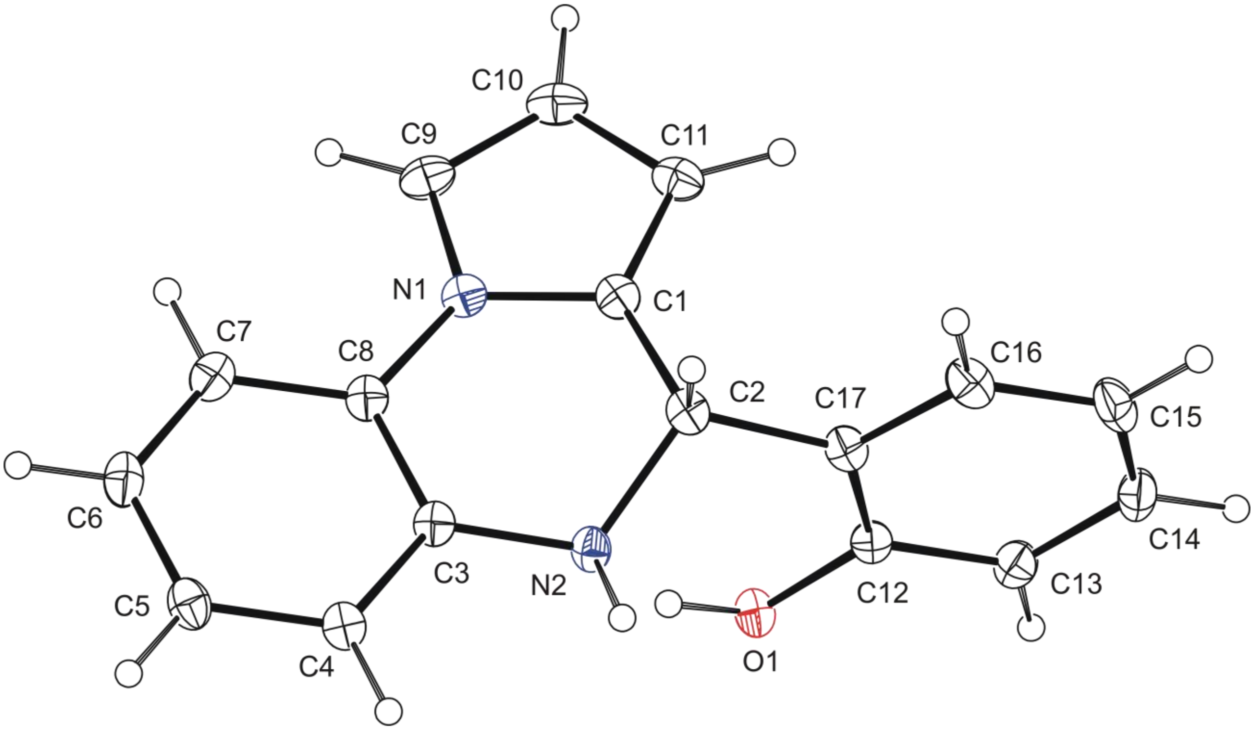

Figure S2. View on molecule of **3r** with atom numbering schema displaying. Displacement ellipsoids are drawn on 30% probability level. The chirality on C2 is (*S*).

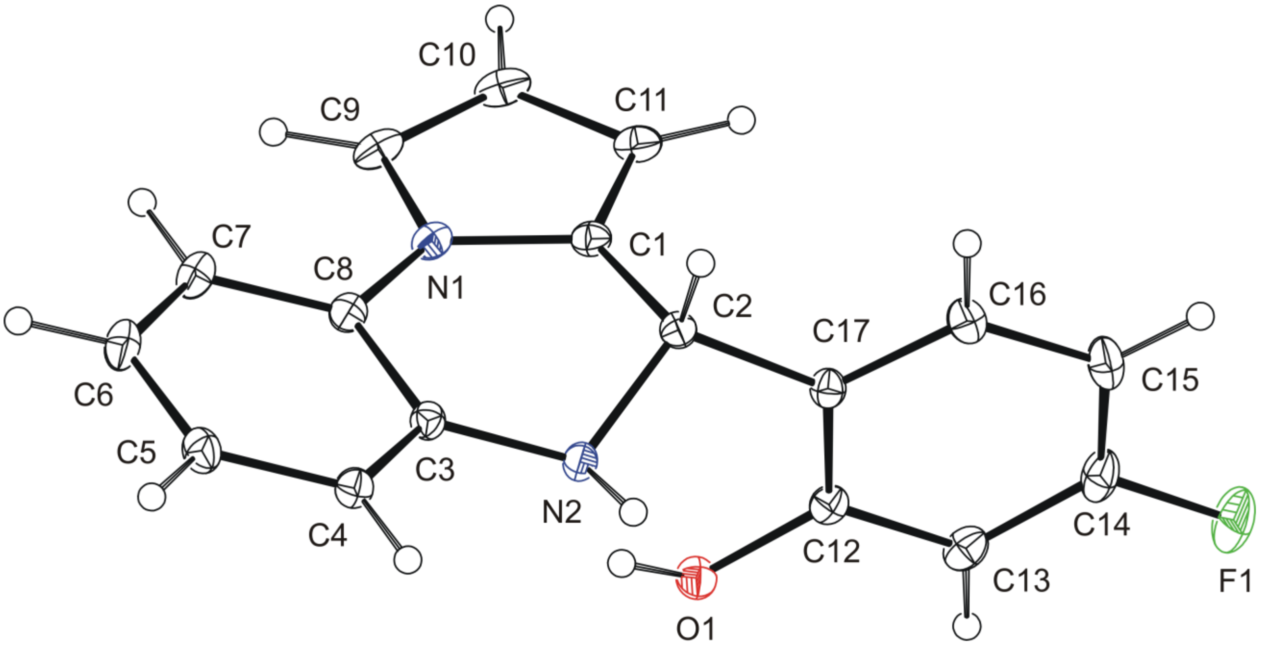

Quantum Chemistry Calculations

Quantum chemistry calculations used density functional theory (DFT) with hybrid functional ωB97X-D that includes dispersion corrections and that gives accurate results for transition states.[3] Optimization of geometry used SVP basis set while energies of the stationary points were refines using much bigger QZVPP basis set.[4] Optimization used analytically calculated gradients and started from available experimental XRD structures and estimated molecular models otherwise. The nature of stationary points was checked using eigenvalues of the mass weighted second derivatives. After locating minima corresponding to reactants and products, one or two key internal coordinates were selected and otherwise fully relaxed potential energy surface scan was performed using pyMCD.[5] The resulting estimates of the transition state geometries were then optimized until stationary points were found and the presence of exactly one imaginary frequence was checked. From the transition states we then followed intrinsic reaction coordinate back to products and reactants. All calculations were done using Gaussian 16 quantum chemistry program package.[6] Transition states leading to *R*-product (left, A) and *S*-product (right, B).

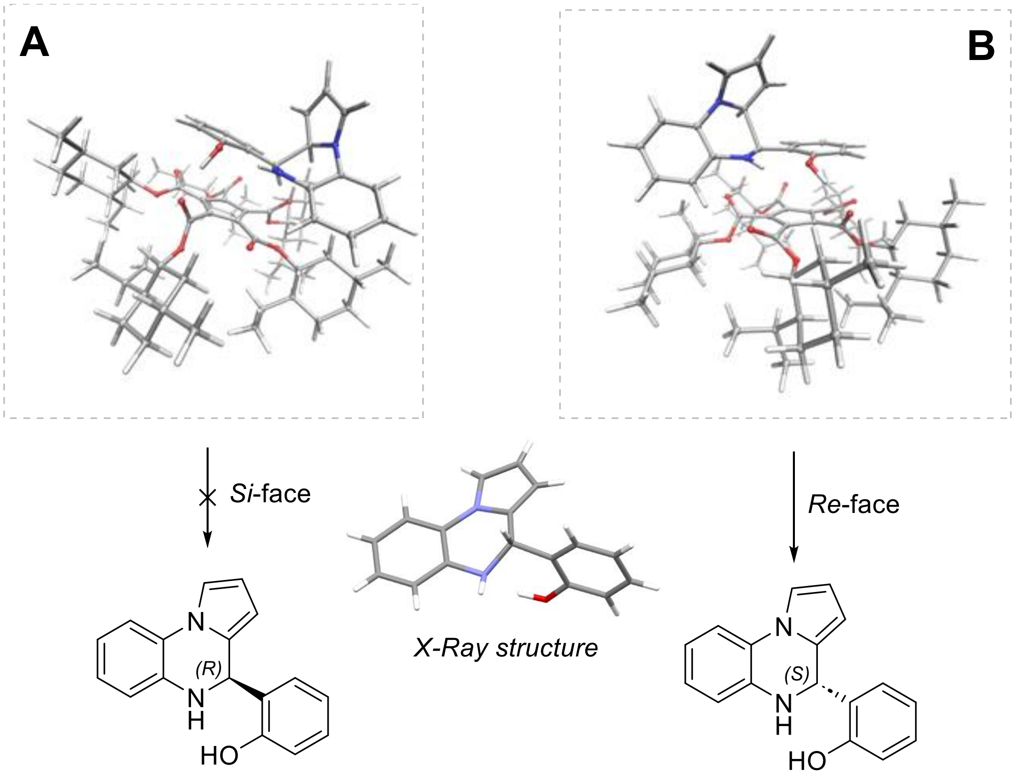

Figure S3: Transition states leading to *R*-product (left, A) and *S*-product (right, B).

Literature

1a. SHELXT: Sheldrick, G.M. (2015). *Acta Cryst*. **A71**, 3-8, b) SHELXL: Sheldrick, G.M. (2015). *Acta Cryst.* **C71**, 3-8.

2. Parsons,S., Flack, H.D. and Wagner, T. (2013) *Acta Cryst*. **B69**, 249-259.

3. Chai, J.-D.; Head-Gordon, M. Long-range corrected hybrid density functionals with damped atom-atom dispersion corrections. *Phys. Chem. Chem. Phys.* **2008**, *10,* 6615–6620. DOI: 10.1039/B810189B

4. Weigend, F. Accurate Coulomb-fitting basis sets for H to Rn, *Phys. Chem. Chem. Phys.* **2026**, *8,* 1057–1065. DOI: 10.1039/B515623H

5. Lee, K.; Jun, H. K.; Woo, Y. K. pyMCD: Python package for searching transition states via the multicoordinate driven method. *Computer Physics Communications* **2023**, *291,* 108831. DOI: 10.1016/j.cpc.2023.108831

6. Gaussian 16, Revision C.01, Gaussian, Inc., Wallingford CT, **2019**.

Cartesian Coordinates

200
R.xyz: E(wB97XD/Def2QZVPP//wB97XD/Def2SVP[n_imag=1]) = -3938.11714656 a. u.
8 -3.406896 1.467130 1.361893
8 -0.030044 3.435513 1.409872
8 2.868564 1.067153 1.100201
8 1.231098 -2.883584 -0.425726
8 -1.897097 -2.971928 0.919237
8 -3.703907 -0.537930 0.389548
8 -1.659477 3.285189 -0.131668
8 2.158550 2.364279 -0.571059
8 2.562245 -1.113100 -0.827460
8 -1.696443 -2.596766 -1.266617
6 -1.551556 0.323864 0.398591
6 -0.539607 1.327027 0.417303
6 0.688417 0.712009 0.184898
6 0.452836 -0.677223 -0.011712
6 -0.928238 -0.908121 0.133071
6 -2.952024 0.516715 0.764729
6 -5.028868 -0.683737 0.900039
1 4.981346 -0.344492 -0.718356
6 3.637531 -1.897115 -1.325507
6 -0.706022 2.777189 0.647296
6 -2.116909 4.625184 0.061062
1 2.166445 3.913073 -4.326170
6 3.148455 3.420400 -4.392527
6 -2.136916 -3.921581 -1.607379
6 1.416176 -1.680314 -0.424000
6 2.006706 1.372213 0.292690
6 -1.558557 -2.248232 -0.004826
1 7.146153 -2.480606 -3.007832
1 3.383564 6.754036 1.569373
6 3.368820 3.130932 -0.534809
1 5.676172 2.677344 -3.495424
1 5.153796 6.863049 1.731425
6 4.303459 6.163886 1.711749
1 4.775532 1.219446 -3.945979
6 6.264091 -1.940769 -2.625939
1 6.048177 -3.208834 -0.913213
1 6.432049 -0.870107 -2.842899
1 3.886791 4.178628 -4.700944
1 4.761581 -1.616402 0.484945
6 4.890632 -1.437861 -0.593433
6 1.618486 -1.044866 2.929482
6 -2.070975 -0.793194 3.891979
6 -0.850169 -1.363336 3.515579
8 -0.790429 -2.706146 3.283883
1 -1.309979 -2.878346 2.451457
6 0.276739 -0.541580 3.336852
1 0.955188 1.505940 3.225897
6 0.116299 0.846836 3.464704
1 -1.194312 2.493495 3.881078
6 -1.096933 1.408955 3.828600
1 -3.161097 1.009091 4.325155
6 -2.194764 0.578962 4.057731
1 -2.922364 -1.460952 4.035460
1 0.944160 -2.979055 2.925620

1 2.048494 -0.465035 2.105138
1 2.794758 -1.063877 7.349619
6 2.989770 -1.002141 6.281704
6 2.961813 -0.407346 4.072397
1 1.896014 0.801473 5.615408
6 2.525515 -0.057513 5.392166
7 3.801600 -1.539840 4.255961
7 1.782393 -2.405230 2.788315
1 3.341605 0.356248 3.384715
6 3.026577 -2.932936 2.480047
6 3.238404 -3.970188 1.567048
1 2.391753 -4.341454 0.989947
6 4.525982 -4.459092 1.369659
1 4.685359 -5.256770 0.641269
6 5.616376 -3.929842 2.064647
1 6.622868 -4.308158 1.879292
6 5.417695 -2.912291 2.994062
1 6.247560 -2.494807 3.567517
6 4.130339 -2.428303 3.196636
6 3.744942 -1.924335 5.533774
1 4.190627 -2.868399 5.846121
1 3.909036 -0.607674 -2.976557
1 7.304458 -1.787482 0.709421
1 3.235785 -1.922413 -5.649349
1 8.302529 -2.147367 -0.723564
1 4.889424 -3.499896 -3.199682
6 3.748386 -1.691418 -2.840067
6 2.452863 -2.041340 -3.603292
1 1.495195 -1.610792 -5.511748
1 1.632512 -1.538476 -3.065071
6 5.003009 -2.411637 -3.348329
1 1.144562 -3.707852 -4.080855
1 2.868712 -4.101395 -4.225791
1 2.104057 -3.959009 -2.613401
6 2.134072 -3.537244 -3.628752
1 7.541082 -0.548910 -0.543874
1 5.112011 -2.256937 -4.432784
6 6.154079 -2.124814 -1.109742
1 -2.947402 -4.205647 -0.916340
6 -0.955136 -4.872397 -1.437649
1 -0.099738 -4.439687 -1.979639
1 -0.666849 -4.894873 -0.377464
6 -1.225169 -6.282560 -1.957442
1 -2.053658 -6.718705 -1.367429
6 -1.685132 -6.206709 -3.413386
1 -0.853658 -5.820069 -4.030807
1 -1.918805 -7.214550 -3.794171
6 -2.899986 -5.296740 -3.556742
1 -3.738747 -5.745705 -2.997429
1 -3.222966 -5.248385 -4.609015
6 -2.647438 -3.868032 -3.049867
1 -1.815645 -3.450962 -3.647644
6 -3.877784 -2.960854 -3.298053
1 -4.117916 -3.111269 -4.366545
6 -5.114512 -3.383182 -2.501214
1 -5.972014 -2.741134 -2.755249
1 -4.946266 -3.282728 -1.417864
1 -5.413894 -4.422202 -2.701986

6 0.002807 -7.171174 -1.784742
1 -0.187492 -8.194391 -2.144099
1 0.305160 -7.232466 -0.727924
1 0.855967 -6.766198 -2.353724
6 -3.603769 -1.464060 -3.117005
1 -2.666095 -1.163734 -3.607684
1 -3.525446 -1.180952 -2.058623
1 -4.421737 -0.876032 -3.562320
6 7.395477 -1.628691 -0.376333
1 3.436565 -2.956334 -1.101668
1 -5.182049 0.078615 1.679978
6 -5.102532 -2.078370 1.510626
1 -4.354556 -2.155460 2.314333
1 -4.795285 -2.810575 0.743827
6 -6.498363 -2.429968 2.023955
1 -6.750621 -1.718118 2.832942
6 -7.522731 -2.231766 0.904179
1 -8.538095 -2.438013 1.280887
1 -7.326296 -2.971659 0.106472
6 -7.452391 -0.822844 0.320116
1 -7.728095 -0.097157 1.105013
1 -8.198995 -0.711227 -0.480945
6 -6.055933 -0.488808 -0.220658
1 -5.810899 -1.244200 -0.985193
6 -5.961655 0.884408 -0.917990
1 -4.898291 1.026783 -1.174537
6 -6.382735 2.059068 -0.035015
1 -5.777468 2.112773 0.880755
1 -7.448357 2.001260 0.239666
1 -6.238255 3.006975 -0.573511
6 -6.738973 0.890956 -2.236327
1 -6.543518 1.816937 -2.798606
1 -7.826936 0.834517 -2.070179
1 -6.452171 0.042512 -2.876943
6 -6.540036 -3.841102 2.601498
1 -7.539244 -4.083645 2.995067
1 -5.813823 -3.960662 3.420192
1 -6.293799 -4.585612 1.826639
6 2.465256 -1.453831 -5.015880
1 3.094002 2.670558 -5.196977
1 -1.652217 5.024739 0.976239
6 -3.629216 4.539032 0.231248
1 -3.850547 3.910945 1.106079
1 -4.037406 4.000725 -0.642231
6 -4.290613 5.912653 0.338651
1 -3.881476 6.415409 1.235841
6 -3.907581 6.759473 -0.876989
1 -4.345375 6.300861 -1.782598
1 -4.345901 7.767635 -0.792241
6 -2.392785 6.858648 -1.030352
1 -1.990365 7.391952 -0.152326
1 -2.139214 7.473254 -1.908215
6 -1.715896 5.484823 -1.143847
1 -2.139708 4.972663 -2.027695
6 -0.187044 5.568100 -1.359616
1 0.207459 4.545078 -1.235342
6 0.151985 6.003696 -2.786728
1 1.241699 6.001301 -2.948837

1 -0.204054 7.024732 -2.999447
1 -0.298495 5.327438 -3.529789
6 0.519938 6.459675 -0.338141
1 1.606796 6.436479 -0.502099
1 0.336046 6.118755 0.691764
1 0.206573 7.511674 -0.425024
6 -5.802435 5.803187 0.507675
1 -6.064933 5.173994 1.371720
1 -6.263240 5.349820 -0.384994
1 -6.260798 6.793836 0.652699
1 2.657954 -0.369490 -4.994426
1 4.237604 5.679756 2.698126
1 4.206656 2.435403 -0.358801
6 3.293281 4.138859 0.605146
1 3.260833 3.596017 1.560699
1 2.335062 4.673218 0.520225
6 4.457206 5.132454 0.598087
1 5.388718 4.563235 0.781043
6 4.591600 5.789180 -0.778448
1 3.711711 6.431524 -0.961028
1 5.469940 6.455155 -0.792046
6 4.702666 4.749038 -1.889338
1 5.632818 4.173032 -1.741747
1 4.797597 5.245301 -2.867810
6 3.505258 3.792077 -1.906000
1 2.591748 4.400539 -2.033694
6 3.528269 2.767052 -3.063117
1 2.745292 2.027247 -2.829888
6 4.853936 2.012708 -3.186453
1 5.145155 1.531039 -2.239712
200
S.xyz: E(wB97XD/Def2QZVPP//wB97XD/Def2SVP[n_imag=1]) = -3938.11476745 a. u.
7 -3.700961 -1.331450 4.826093
7 -2.104231 -1.722198 2.782088
6 -1.879392 -0.434554 3.194899
6 1.886928 -0.241820 3.764633
6 0.641629 -0.791272 3.433077
8 0.548568 -2.095024 3.081191
1 1.334423 -2.299909 2.510632
6 -0.496382 0.035738 3.434198
1 -1.201518 2.064437 3.613797
6 -0.333084 1.405492 3.689143
1 1.003384 3.022071 4.156202
6 0.903568 1.948411 3.993875
1 3.005153 1.517103 4.265754
6 2.017661 1.108928 4.046435
1 2.759795 -0.895836 3.754613
1 -1.295577 -2.313717 2.582007
1 -2.484339 0.285049 2.628655
1 -1.811895 -1.836338 7.471915
6 -2.308278 -1.440719 6.589177
6 -2.888365 -0.171075 4.783155
1 -1.377039 0.541269 6.259148
6 -2.085388 -0.231459 5.965969
1 -3.392420 0.751926 4.490966
6 -3.419524 -2.150540 2.606924
6 -3.885313 -2.871039 1.507889
1 -3.174553 -3.106923 0.717138

6 -5.223170 -3.252126 1.456258
1 -5.582134 -3.804611 0.585344
6 -6.111449 -2.925670 2.483200
1 -7.161244 -3.215803 2.416568
6 -5.651103 -2.238367 3.604156
1 -6.312173 -2.000779 4.439633
6 -4.314214 -1.865470 3.656076
6 -3.283274 -2.111591 5.828752
1 -3.654192 -3.131728 5.923439
8 -1.198464 -3.082843 0.112766
8 -3.210285 0.268230 0.500369
8 -0.671580 3.329350 1.253797
8 3.233997 1.658727 1.347679
8 2.639703 -2.155646 1.405422
8 0.856086 -3.085808 -0.778124
8 -2.591647 -0.793705 -1.377369
8 -1.673257 2.736534 -0.660444
8 1.973949 3.034039 0.097393
8 3.579669 -0.847151 -0.139942
6 0.066154 -1.061386 0.031680
6 -0.891684 -0.017120 0.011724
6 -0.223794 1.186097 0.283654
6 1.149341 0.901240 0.466887
6 1.321981 -0.488883 0.323300
6 -0.184142 -2.481039 -0.191940
6 0.860363 -4.508689 -0.913122
1 1.205068 5.467430 -0.029578
6 2.859200 4.136669 0.274024
6 -2.340149 -0.150184 -0.237908
6 -3.899528 -1.302728 -1.662910
1 -2.551282 3.814335 -4.925863
6 -1.624353 4.318787 -4.608535
6 4.891581 -1.367912 0.115222
6 2.225107 1.869032 0.710253
6 -0.852120 2.516697 0.376563
6 2.557427 -1.245339 0.587762
1 4.180627 7.785396 -0.400273
1 -6.263113 4.693882 0.392511
6 -2.288123 4.017386 -0.764822
1 -0.268230 5.900277 -2.588570
1 -6.051506 6.439454 0.668112
6 -5.554829 5.460134 0.748954
1 0.744229 4.681264 -3.389204
6 3.559963 6.878626 -0.490445
1 3.589250 6.465843 1.610095
1 2.814462 7.084361 -1.280585
1 -1.739505 5.395071 -4.815027
1 1.511180 5.076703 1.659498
6 2.010603 5.324422 0.710544
1 2.818908 4.582365 -1.796625
1 1.438790 7.596004 2.196370
1 5.465892 4.138032 -3.201645
1 2.532291 8.714898 1.343936
1 5.211251 5.543566 -0.142393
6 3.604238 4.399138 -1.040420
6 4.418928 3.180169 -1.529043
1 5.267544 2.383088 -3.369740
1 3.756336 2.304080 -1.435338

6 4.423644 5.688372 -0.902096
1 6.135793 1.956822 -1.010696
1 6.421544 3.693498 -0.800382
1 5.419201 2.792626 0.376918
6 5.666105 2.899170 -0.689505
1 1.162036 7.974929 0.481640
1 4.942157 5.906812 -1.848599
6 2.822910 6.614594 0.825539
1 4.792279 -2.414117 0.444989
6 5.519093 -0.537045 1.229509
1 5.414482 0.524701 0.959622
1 4.930413 -0.676642 2.148764
6 6.990257 -0.870126 1.470295
1 7.059128 -1.926617 1.793213
6 7.761373 -0.738912 0.155768
1 7.747745 0.319705 -0.160849
1 8.819557 -1.008866 0.306215
6 7.149214 -1.610797 -0.935797
1 7.254532 -2.668505 -0.638294
1 7.710859 -1.498533 -1.877070
6 5.669172 -1.294179 -1.199712
1 5.612069 -0.240551 -1.529679
6 5.104316 -2.159842 -2.353282
1 5.844748 -2.052591 -3.166882
6 5.026738 -3.650234 -2.012658
1 4.696227 -4.227889 -2.889889
1 4.298401 -3.838349 -1.208345
1 5.995221 -4.063603 -1.694972
6 7.578398 0.005307 2.572341
1 8.634384 -0.242683 2.761426
1 7.025985 -0.116109 3.516716
1 7.526030 1.069541 2.290059
6 3.764595 -1.668090 -2.913126
1 3.764352 -0.577769 -3.061029
1 2.922202 -1.909485 -2.249906
1 3.567978 -2.141431 -3.888159
6 1.943206 7.790733 1.237538
1 3.580081 3.880140 1.065837
1 -0.176376 -4.866114 -0.824974
6 1.692039 -5.094797 0.221199
1 1.213883 -4.843001 1.181278
1 2.676763 -4.597523 0.227550
6 1.871643 -6.607866 0.090752
1 0.870741 -7.074414 0.163035
6 2.434710 -6.944904 -1.292266
1 2.527284 -8.037606 -1.404954
1 3.457377 -6.532486 -1.370562
6 1.569959 -6.370806 -2.412720
1 0.575325 -6.849497 -2.379595
1 2.006116 -6.627892 -3.389911
6 1.416386 -4.849570 -2.296130
1 2.428330 -4.413590 -2.335923
6 0.615159 -4.193557 -3.440315
1 0.582637 -3.117529 -3.202057
6 -0.830072 -4.683977 -3.543117
1 -1.383957 -4.536580 -2.604741
1 -0.882155 -5.752494 -3.807768
1 -1.367297 -4.127882 -4.327074

6 1.334263 -4.335168 -4.782731
1 0.847513 -3.717548 -5.552939
1 1.325474 -5.375796 -5.145118
1 2.384522 -4.012771 -4.709446
6 2.738371 -7.164136 1.215946
1 2.842698 -8.257408 1.138231
1 2.309809 -6.932644 2.203117
1 3.749291 -6.726050 1.180440
6 4.774708 3.300658 -3.012008
1 -0.806889 3.949899 -5.246681
1 -4.478118 -1.321299 -0.725587
6 -3.665089 -2.718472 -2.177711
1 -3.129617 -3.295315 -1.409411
1 -2.977326 -2.650374 -3.038090
6 -4.943720 -3.428582 -2.614639
1 -5.608975 -3.508271 -1.733393
6 -5.667630 -2.569963 -3.651860
1 -5.050688 -2.521144 -4.567638
1 -6.621237 -3.042252 -3.939985
6 -5.917872 -1.160721 -3.123494
1 -6.597510 -1.227806 -2.256415
1 -6.445593 -0.563964 -3.882517
6 -4.628537 -0.438217 -2.699702
1 -3.960230 -0.398425 -3.580143
6 -4.870895 1.022168 -2.254770
1 -3.909313 1.395206 -1.863338
6 -5.243262 1.905351 -3.448076
1 -5.360855 2.954305 -3.135673
1 -6.197574 1.599154 -3.905354
1 -4.467857 1.874494 -4.229557
6 -5.904419 1.159083 -1.135511
1 -5.982984 2.210392 -0.818117
1 -5.616067 0.574389 -0.250738
1 -6.908538 0.848018 -1.464897
6 -4.652943 -4.837568 -3.120989
1 -4.150112 -5.443421 -2.351331
1 -3.990229 -4.805800 -4.000912
1 -5.578023 -5.357393 -3.414647
1 3.875450 3.459248 -3.627411
1 -5.360569 5.264857 1.814815
1 -1.576688 4.767450 -0.381567
6 -3.551981 4.075040 0.081010
1 -3.285111 3.894044 1.132904
1 -4.218702 3.254389 -0.230630
6 -4.265100 5.420947 -0.064776
1 -3.586328 6.201378 0.327906
6 -4.512257 5.729812 -1.543958
1 -5.251927 5.006989 -1.935406
1 -4.968644 6.727941 -1.648081
6 -3.237100 5.642711 -2.383330
1 -2.536447 6.437608 -2.070743
1 -3.480499 5.838824 -3.438107
6 -2.563630 4.272159 -2.245283
1 -3.304116 3.517865 -2.561085
6 -1.319972 4.049185 -3.134357
1 -1.081929 2.977561 -3.040556
6 -0.080076 4.816720 -2.671531
1 0.280141 4.450093 -1.699613

200
R-reactant.xyz: E(wB97XD/Def2QZVPP//wB97XD/Def2SVP[n_imag=0]) = -3938.16545940 a. u.
8 -2.945168 2.402444 1.222888
8 0.821223 3.333934 0.989575
8 2.992943 -0.082205 0.608712
8 0.377505 -3.258292 0.374653
8 -2.687436 -2.189408 1.609812
8 -3.808218 0.453292 0.506743
8 -0.817209 3.454127 -0.542165
8 2.522079 1.569118 -0.823543
8 1.705132 -2.106766 -1.031742
8 -2.512431 -2.439415 -0.596795
6 -1.504238 0.672794 0.435343
6 -0.262878 1.338116 0.251575
6 0.724852 0.363877 0.064992
6 0.101693 -0.910904 0.117444
6 -1.265358 -0.717491 0.354247
6 -2.785109 1.295126 0.756906
6 -5.126541 0.780327 0.947688
1 4.118143 -1.762503 -1.656823
6 2.578827 -3.198723 -1.312362
6 -0.016738 2.798851 0.297434
6 -0.912920 4.880786 -0.479026
1 2.820630 2.928781 -4.661290
6 3.629010 2.185060 -4.724232
6 -3.303896 -3.641821 -0.590439
6 0.722471 -2.207308 -0.137469
6 2.176189 0.573558 -0.012484
6 -2.229251 -1.830151 0.530895
1 5.601670 -4.886245 -3.204503
1 5.059276 5.464031 1.102962
6 3.898193 1.957126 -0.868548
1 5.830417 0.747640 -3.947822
1 6.783680 5.036472 1.232948
6 5.761566 4.628153 1.253572
1 4.516600 -0.429247 -4.147999
6 4.853898 -4.127502 -2.921002
1 4.952612 -4.588342 -0.832820
1 5.020354 -3.258268 -3.583216
1 4.538395 2.702987 -5.070795
1 4.091725 -2.393258 -0.014074
6 3.992100 -2.687032 -1.069223
6 1.691083 -0.450313 3.140080
6 -1.931597 0.322301 3.774679
6 -0.782222 -0.429513 3.519725
8 -0.832278 -1.754099 3.347726
1 -1.646259 -1.990485 2.810809
6 0.473056 0.226979 3.431274
1 1.493936 2.132657 3.461519
6 0.530931 1.632418 3.578490
1 -0.563397 3.449806 3.896818
6 -0.612278 2.364150 3.819654
1 -2.752635 2.275270 4.090130
6 -1.841620 1.699455 3.919775
1 -2.892282 -0.191087 3.830115
1 0.906808 -2.251508 3.006088
1 2.586045 0.159507 3.019636
1 4.357211 1.371989 6.028196

6 4.476493 0.828101 5.093978
6 4.947651 0.314116 2.945999
1 5.203720 2.382532 3.633349
6 4.924043 1.353764 3.847527
7 4.530183 -0.821879 3.598731
7 1.803889 -1.747518 2.998623
1 5.158706 0.287275 1.880375
6 2.961388 -2.513419 2.730078
6 2.758378 -3.796669 2.213966
1 1.740390 -4.120069 1.991248
6 3.838511 -4.619935 1.919077
1 3.656402 -5.612252 1.502565
6 5.139674 -4.173813 2.135909
1 5.992367 -4.810400 1.894086
6 5.347347 -2.908431 2.676087
1 6.354093 -2.542435 2.884428
6 4.273036 -2.077306 2.989032
6 4.239822 -0.513945 4.909296
1 3.898953 -1.284987 5.596494
1 2.519958 -2.751592 -3.379905
1 6.602101 -2.831535 -0.182494
1 1.205868 -4.924822 -5.053651
1 7.248102 -3.849912 -1.496238
1 3.321160 -5.582588 -2.522637
6 2.360830 -3.653071 -2.759052
6 0.917502 -4.134434 -3.028957
1 -0.453644 -4.409281 -4.698450
1 0.249208 -3.379298 -2.581857
6 3.443790 -4.672706 -3.135629
1 -0.469903 -5.730614 -2.540755
1 1.185281 -6.299008 -2.807258
1 0.753495 -5.454451 -1.289888
6 0.588889 -5.478645 -2.377588
1 6.625198 -2.221234 -1.852216
1 3.313842 -4.985876 -4.183019
6 5.070097 -3.691218 -1.470119
1 -4.134942 -3.502365 0.119933
6 -2.415759 -4.793278 -0.129506
1 -1.493202 -4.764661 -0.729224
1 -2.109921 -4.615287 0.910865
6 -3.076692 -6.163853 -0.269352
1 -3.964914 -6.187150 0.390306
6 -3.566265 -6.354035 -1.705424
1 -2.692348 -6.393135 -2.380792
1 -4.083581 -7.322072 -1.806437
6 -4.491110 -5.217932 -2.127025
1 -5.395305 -5.251537 -1.495204
1 -4.834301 -5.364635 -3.163582
6 -3.833803 -3.833738 -2.013120
1 -2.943244 -3.838395 -2.669306
6 -4.774024 -2.720429 -2.537868
1 -5.084122 -3.073153 -3.538384
6 -6.049754 -2.561544 -1.707131
1 -6.701272 -1.791383 -2.148071
1 -5.820925 -2.239598 -0.679450
1 -6.635907 -3.490094 -1.647740
6 -2.127664 -7.274895 0.169337
1 -2.604714 -8.263895 0.089728

1 -1.805441 -7.135339 1.212528
1 -1.223695 -7.283486 -0.461163
6 -4.091947 -1.363522 -2.744374
1 -3.123446 -1.472024 -3.254671
1 -3.905478 -0.842308 -1.795242
1 -4.729590 -0.714027 -3.364410
6 6.464887 -3.121100 -1.235927
1 2.347022 -4.024688 -0.622390
1 -5.074078 1.717684 1.522491
6 -5.585105 -0.358578 1.851030
1 -4.905238 -0.428786 2.714296
1 -5.472576 -1.308335 1.300031
6 -7.033058 -0.202437 2.316055
1 -7.094823 0.720931 2.922811
6 -7.947341 -0.014492 1.103312
1 -8.985719 0.146344 1.436464
1 -7.948279 -0.946640 0.509044
6 -7.488173 1.147827 0.226328
1 -7.568814 2.085057 0.804084
1 -8.163614 1.255976 -0.635793
6 -6.045887 0.966432 -0.263688
1 -6.006245 0.015523 -0.820252
6 -5.564544 2.061364 -1.238393
1 -4.506988 1.833788 -1.455362
6 -5.621538 3.475964 -0.660955
1 -5.009878 3.569675 0.247668
1 -6.654612 3.781161 -0.429050
1 -5.228059 4.197971 -1.391923
6 -6.315411 1.988241 -2.569796
1 -5.857228 2.659395 -3.312169
1 -7.369085 2.292366 -2.462390
1 -6.300001 0.968516 -2.984842
6 -7.466752 -1.375809 3.188846
1 -8.500520 -1.248717 3.545259
1 -6.815077 -1.483516 4.069550
1 -7.420280 -2.320157 2.621968
6 0.606153 -4.165314 -4.526299
1 3.353190 1.453889 -5.499741
1 -0.357909 5.226823 0.406875
6 -2.395297 5.198430 -0.328830
1 -2.764172 4.724815 0.591878
1 -2.930244 4.709689 -1.161531
6 -2.688503 6.697650 -0.348118
1 -2.167279 7.156452 0.513803
6 -2.103473 7.315077 -1.619729
1 -2.645172 6.910214 -2.494264
1 -2.268596 8.405173 -1.625214
6 -0.614010 7.010368 -1.751373
1 -0.084635 7.492458 -0.911742
1 -0.215024 7.466384 -2.670540
6 -0.311828 5.504309 -1.743860
1 -0.857736 5.047716 -2.590573
6 1.185126 5.176483 -1.944186
1 1.296126 4.092565 -1.769259
6 1.625216 5.444576 -3.384969
1 2.673687 5.143551 -3.537198
1 1.557121 6.513775 -3.642187
1 1.007805 4.884529 -4.104587

6 2.103161 5.896426 -0.955613
1 3.144927 5.578060 -1.106683
1 1.837319 5.663339 0.085987
1 2.078904 6.988687 -1.093843
6 -4.180456 6.980114 -0.203432
1 -4.586958 6.523820 0.712062
1 -4.740853 6.565209 -1.056886
1 -4.381162 8.062100 -0.165085
1 0.805765 -3.190466 -4.997184
1 5.579610 4.221879 2.260075
1 4.512452 1.057577 -0.700063
6 4.156246 2.969632 0.239837
1 3.982973 2.486944 1.214157
1 3.401645 3.766276 0.154202
6 5.565050 3.563328 0.178880
1 6.286855 2.744780 0.365789
6 5.850147 4.112370 -1.222690
1 5.204042 4.990189 -1.401890
1 6.889154 4.476371 -1.277669
6 5.601255 3.067595 -2.307260
1 6.318627 2.239397 -2.172217
1 5.806972 3.496034 -3.300668
6 4.171583 2.516013 -2.263611
1 3.477688 3.365560 -2.391469
6 3.850374 1.494992 -3.378057
1 2.894355 1.027877 -3.091992
6 4.891971 0.380090 -3.502751
1 5.138418 -0.066538 -2.527251
200
S-reactant.xyz: E(wB97XD/Def2QZVPP//wB97XD/Def2SVP[n_imag=0]) = -3938.16858862 a. u.
7 -4.420019 -0.254288 3.622252
7 -1.923929 -1.631633 2.916023
6 -1.605064 -0.370623 3.075638
6 2.061888 -0.049669 3.848991
6 0.854658 -0.655390 3.492326
8 0.787522 -1.959744 3.189968
1 1.587918 -2.184427 2.639863
6 -0.319964 0.134303 3.418464
1 -1.135429 2.131894 3.548265
6 -0.231957 1.527562 3.651459
1 1.033474 3.195595 4.119102
6 0.970715 2.117392 3.975089
1 3.076943 1.777363 4.312692
6 2.116272 1.317741 4.075653
1 2.960433 -0.665143 3.902302
1 -1.133288 -2.285048 2.875769
1 -2.393478 0.368337 2.932942
1 -3.692001 1.703137 6.149575
6 -3.963734 1.254375 5.196896
6 -4.634145 0.981162 3.057009
1 -4.433013 3.011917 3.869150
6 -4.358063 1.935960 4.009683
1 -4.905381 1.066944 2.008457
6 -3.208227 -2.165513 2.640202
6 -3.262151 -3.437992 2.066233
1 -2.328793 -3.949700 1.838003
6 -4.482740 -4.008564 1.723936
1 -4.498058 -4.998863 1.265319

6 -5.669113 -3.313026 1.941646
1 -6.628593 -3.747830 1.656350
6 -5.625382 -2.065107 2.556735
1 -6.540298 -1.514667 2.781819
6 -4.411059 -1.492941 2.928639
6 -4.007563 -0.092933 4.926102
1 -3.790643 -0.957998 5.548611
8 -0.714228 -3.189195 0.611307
8 -3.079255 -0.001447 0.537783
8 -0.658394 3.363329 1.008191
8 3.058735 2.182939 1.417059
8 2.863733 -1.974362 1.533048
8 1.139913 -3.057463 -0.636379
8 -2.353271 -1.277303 -1.155519
8 -1.878033 2.480966 -0.656973
8 1.981675 3.023715 -0.366382
8 3.802838 -0.433461 0.213545
6 0.287763 -1.066084 0.190196
6 -0.745583 -0.117419 0.063504
6 -0.175410 1.165895 0.224464
6 1.206073 1.010630 0.440836
6 1.491162 -0.370861 0.435879
6 0.161470 -2.523543 0.091432
6 1.236000 -4.480890 -0.749951
1 1.071249 5.106875 -1.459590
6 2.683703 4.266335 -0.340747
6 -2.170929 -0.430345 -0.145566
6 -3.598923 -1.972818 -1.308099
1 -2.851895 2.776509 -4.646536
6 -2.457921 3.796751 -4.520186
6 5.129705 -0.831679 0.575560
6 2.182087 2.109560 0.586084
6 -0.897409 2.446769 0.252660
6 2.760516 -1.007268 0.779817
1 3.740653 7.766710 -1.816926
1 -6.650502 2.976088 0.847877
6 -2.776427 3.590906 -0.660168
1 -2.132298 6.241128 -3.274388
1 -7.007116 4.697611 1.116830
6 -6.208426 3.940628 1.147630
1 -0.519722 5.534401 -3.491678
6 3.271608 6.770653 -1.759415
1 2.729859 6.981895 0.301516
1 2.770762 6.602512 -2.730638
1 -3.230953 4.501639 -4.867428
1 0.897591 5.277967 0.288501
6 1.625059 5.344664 -0.534795
1 3.189518 4.096548 -2.389414
1 0.384859 7.774483 -0.059216
1 6.156129 3.833323 -2.914900
1 1.549748 8.809543 -0.922652
1 4.880425 5.928963 -0.608128
6 3.742141 4.288039 -1.450564
6 4.801896 3.169813 -1.321064
1 6.239187 2.098904 -2.560677
1 4.252833 2.235793 -1.113647
6 4.338016 5.699949 -1.541634
1 6.458463 2.524541 -0.077645

1 6.414898 4.275540 -0.331194
1 5.258649 3.494996 0.792085
6 5.782847 3.388253 -0.168681
1 0.592370 7.604480 -1.816833
1 5.083955 5.739935 -2.350452
6 2.216359 6.748943 -0.650698
1 5.098550 -1.884085 0.895918
6 5.592733 0.036138 1.738006
1 5.458640 1.092515 1.457796
1 4.928769 -0.140057 2.598458
6 7.048076 -0.236831 2.119466
1 7.118067 -1.286360 2.463537
6 7.943502 -0.098097 0.885534
1 7.950904 0.960283 0.568593
1 8.984195 -0.351935 1.145135
6 7.464188 -0.970377 -0.271937
1 7.568728 -2.033551 0.009235
1 8.109625 -0.819396 -1.151675
6 6.007384 -0.686465 -0.665590
1 5.948395 0.373738 -0.970477
6 5.561465 -1.541532 -1.877912
1 6.431438 -1.544710 -2.559127
6 5.265523 -2.997813 -1.512158
1 5.106613 -3.598155 -2.421380
1 4.348814 -3.074595 -0.906338
1 6.086215 -3.466424 -0.947267
6 7.499736 0.670445 3.259504
1 8.540369 0.460629 3.551069
1 6.865481 0.539992 4.149850
1 7.439849 1.729219 2.958860
6 4.391897 -0.939346 -2.663980
1 4.563852 0.125105 -2.884003
1 3.443486 -1.015899 -2.114770
1 4.268945 -1.464792 -3.624375
6 1.127250 7.794052 -0.871855
1 3.167616 4.375922 0.642698
1 0.219208 -4.900835 -0.690106
6 2.074318 -5.010209 0.408055
1 1.547416 -4.805930 1.353335
1 3.015506 -4.436268 0.443808
6 2.383042 -6.502465 0.277365
1 1.423332 -7.052441 0.312582
6 3.020013 -6.781162 -1.086544
1 3.212145 -7.860698 -1.199107
1 4.003935 -6.279045 -1.130554
6 2.139397 -6.280340 -2.228786
1 1.189080 -6.842020 -2.215900
1 2.614469 -6.497463 -3.198028
6 1.852211 -4.778773 -2.117278
1 2.822500 -4.253966 -2.129325
6 1.017463 -4.199752 -3.280370
1 0.737408 -3.179313 -2.972759
6 -0.275222 -4.973007 -3.548398
1 -0.865263 -5.121193 -2.631235
1 -0.074820 -5.964894 -3.984297
1 -0.911493 -4.425122 -4.260722
6 1.852913 -4.068851 -4.554271
1 1.273229 -3.586630 -5.356270

1 2.180123 -5.051459 -4.931548
1 2.751495 -3.457905 -4.376990
6 3.253360 -6.991238 1.430757
1 3.451999 -8.071223 1.352584
1 2.772086 -6.803844 2.402861
1 4.224362 -6.469483 1.433087
6 5.550485 2.955273 -2.638241
1 -1.590749 3.907312 -5.189298
1 -4.141163 -1.929353 -0.349672
6 -3.205979 -3.409837 -1.629077
1 -2.609592 -3.804477 -0.792879
1 -2.535897 -3.385753 -2.505872
6 -4.399259 -4.311507 -1.936269
1 -5.042105 -4.341461 -1.036782
6 -5.227637 -3.691309 -3.061356
1 -4.627421 -3.685955 -3.989516
1 -6.117257 -4.309611 -3.265362
6 -5.647524 -2.267913 -2.708647
1 -6.300398 -2.307777 -1.819552
1 -6.256178 -1.840900 -3.520034
6 -4.453243 -1.345790 -2.417321
1 -3.810721 -1.333189 -3.317441
6 -4.872230 0.116455 -2.141074
1 -3.966888 0.646160 -1.802309
6 -5.341634 0.811930 -3.420752
1 -5.575576 1.870495 -3.228067
1 -6.253864 0.349548 -3.830406
1 -4.566131 0.779528 -4.201756
6 -5.919997 0.249326 -1.034541
1 -6.097535 1.310620 -0.804254
1 -5.587041 -0.237387 -0.105996
1 -6.888551 -0.181362 -1.333720
6 -3.953912 -5.735197 -2.253135
1 -3.372234 -6.166185 -1.422950
1 -3.313753 -5.753564 -3.150039
1 -4.816079 -6.393293 -2.442328
1 4.854718 2.750330 -3.466779
1 -5.878078 3.841070 2.192316
1 -2.231311 4.479615 -0.304385
6 -3.926472 3.282181 0.291540
1 -3.528628 3.204689 1.315524
1 -4.314714 2.284817 0.028851
6 -5.051661 4.315888 0.226268
1 -4.641494 5.285924 0.565182
6 -5.510948 4.494828 -1.222999
1 -5.982168 3.555761 -1.566639
1 -6.289920 5.272757 -1.278059
6 -4.346677 4.847467 -2.144926
1 -3.936570 5.825827 -1.839674
1 -4.706276 4.970685 -3.178457
6 -3.238207 3.789984 -2.103490
1 -3.685536 2.828105 -2.408900
6 -2.062257 4.060268 -3.066830
1 -1.284909 3.324901 -2.803500
6 -1.451686 5.454631 -2.910564
1 -1.202914 5.682106 -1.862931

^{1}H, ^{13}C, ^{19}F NMR DATA:

1H NMR (400 MHz, CDCl3): (1R,2S)-2-phenylcyclohexan-1-ol SM1.

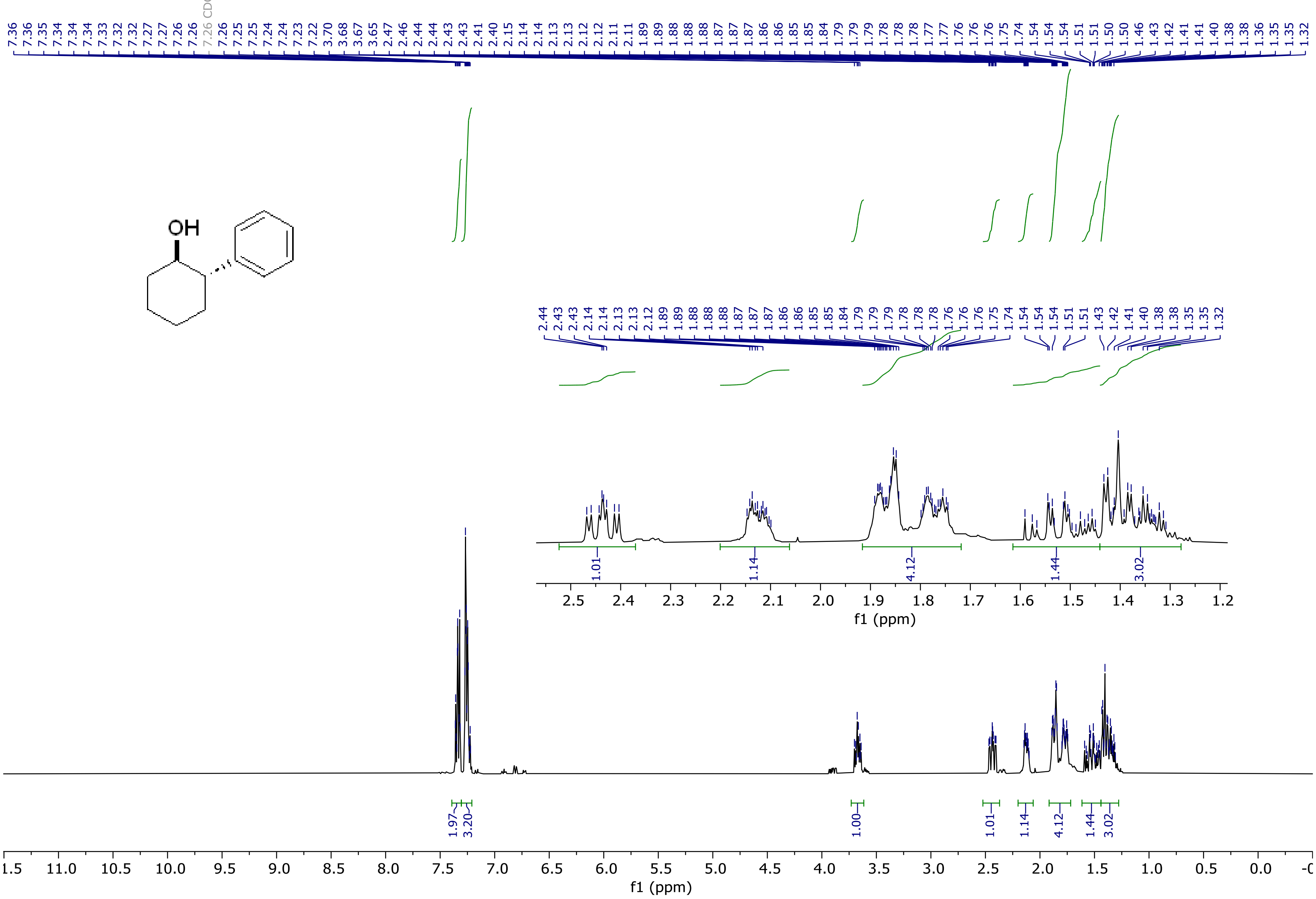

13C{1H} NMR: (1R,2S)-2-phenylcyclohexan-1-ol SM1.

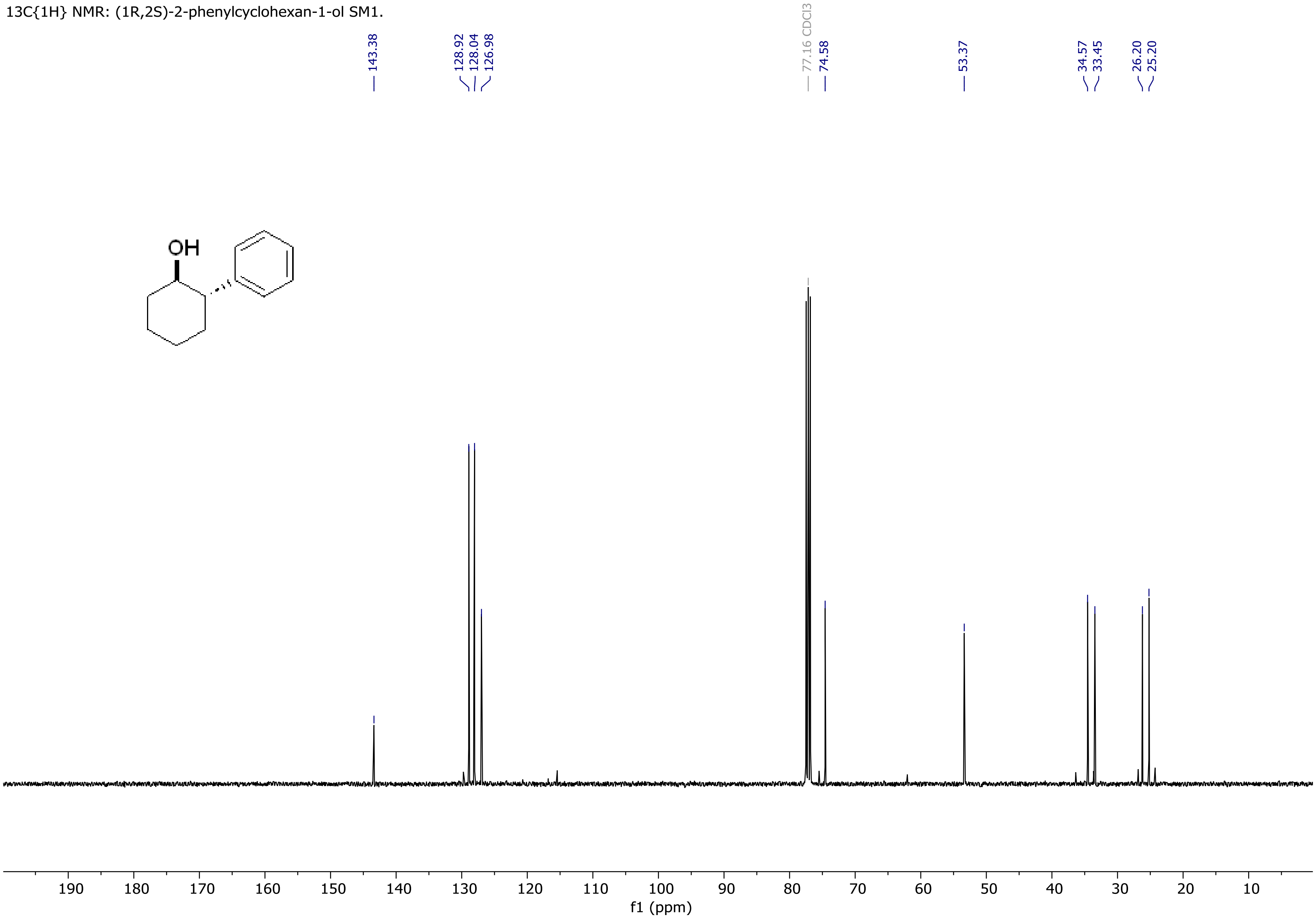

1H NMR (400 MHz, CDCl3): (1R,2S)-2-(p-tolyl)cyclohexan-1-ol SM2.

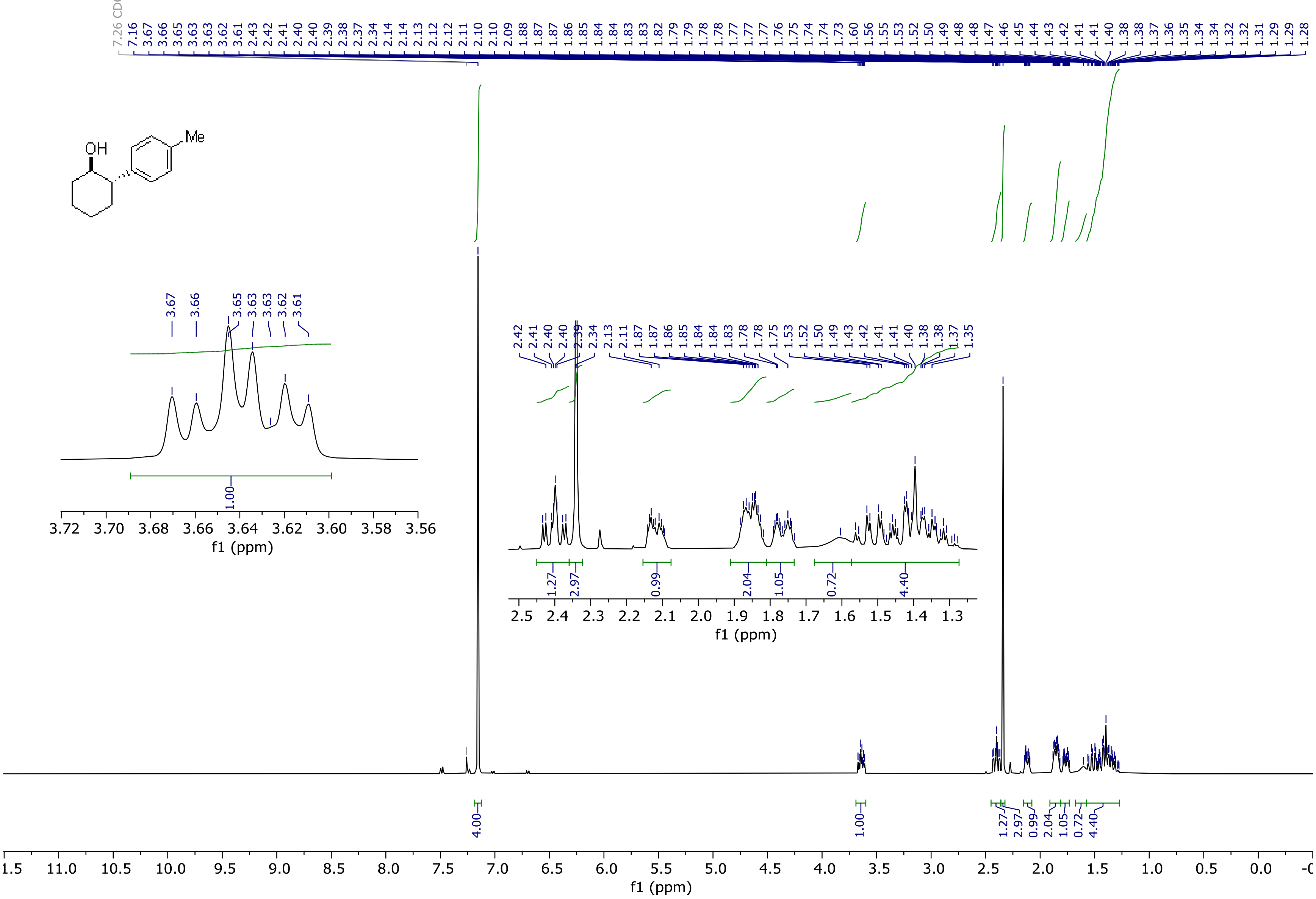

13C{1H} NMR: (101 MHz, CDCl3): (1R,2S)-2-(p-tolyl)cyclohexan-1-ol SM2.

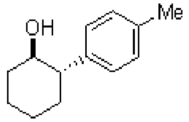

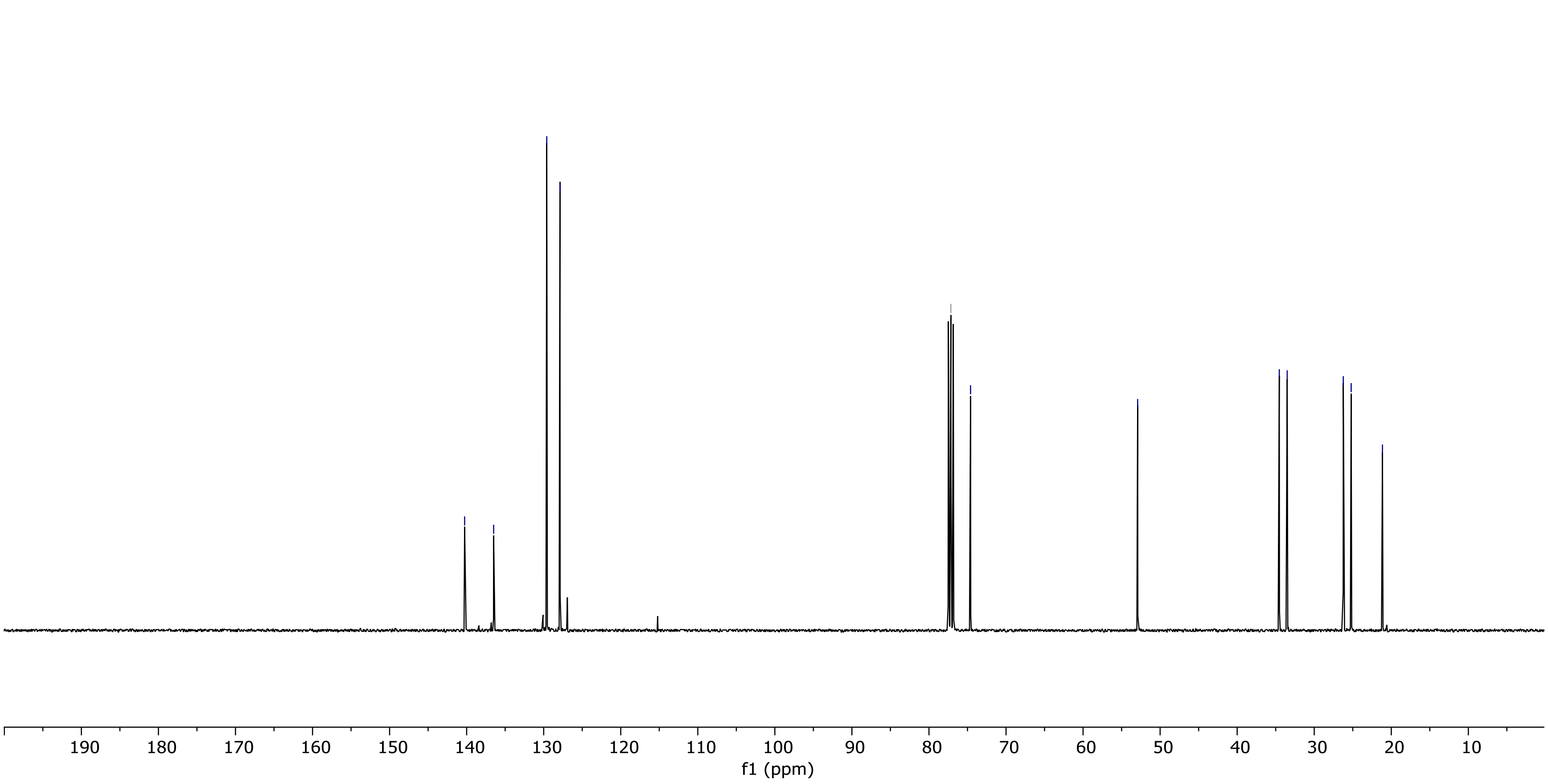

1H NMR (400 MHz, CDCl3): (1R,2S)-2-(4-methoxyphenyl)cyclohexan-1-ol SM3.

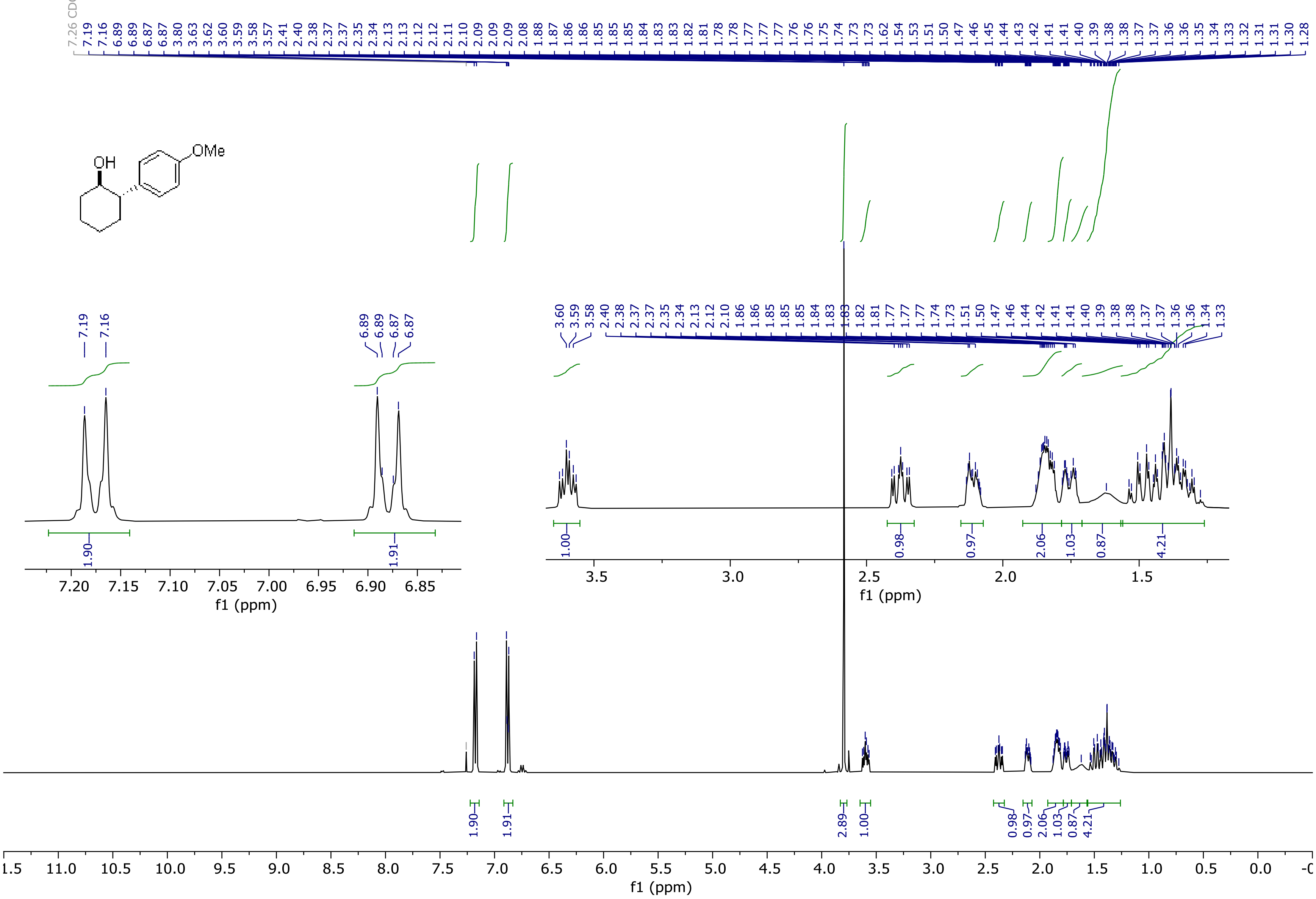

13C{1H} NMR: (101 MHz, CDCl3): (1R,2S)-2-(4-methoxyphenyl)cyclohexan-1-ol SM3.

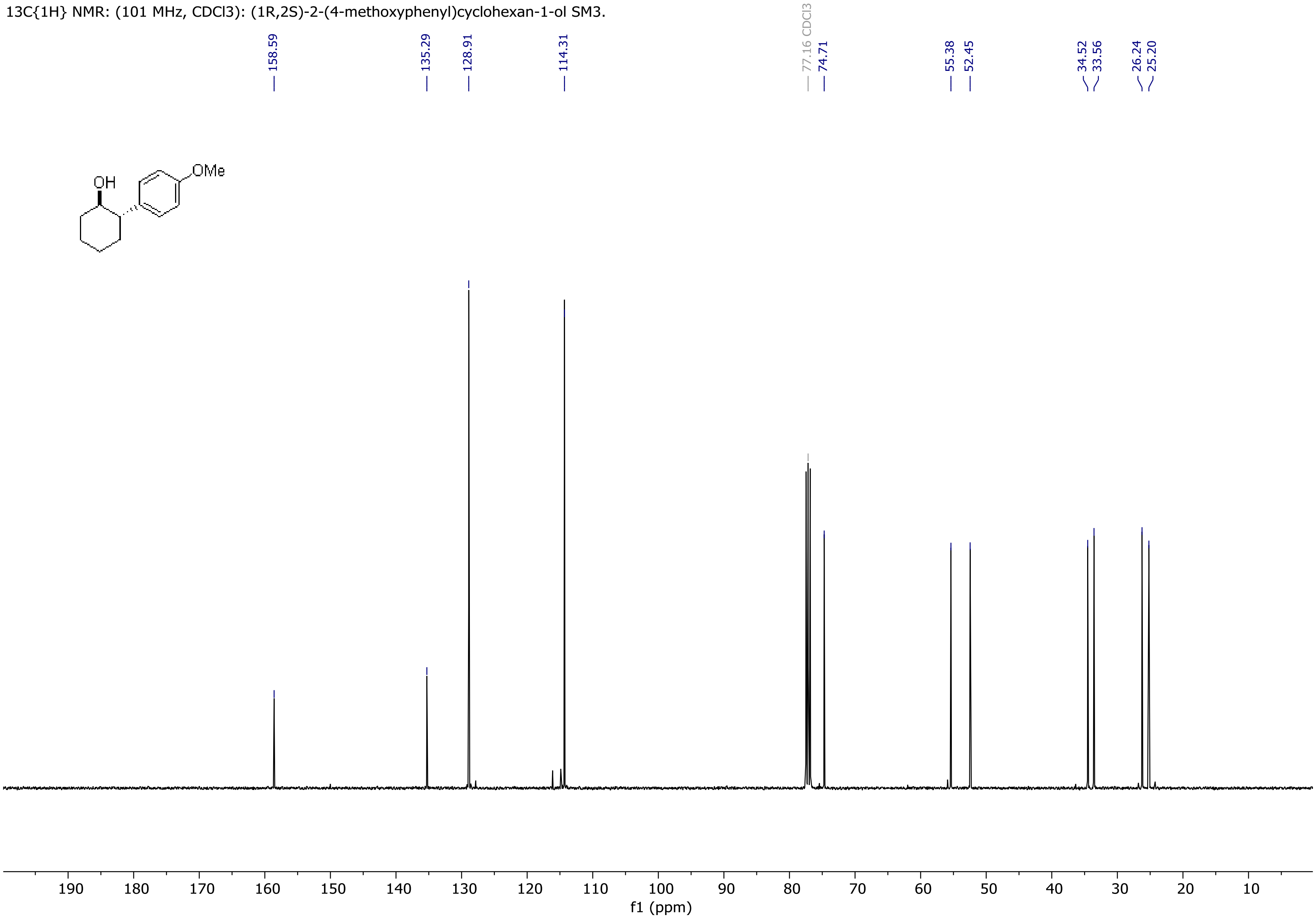

1H NMR (400 MHz, CDCl3): Tetramethyl 5-(hydroxy(methoxy)methylene)cyclopenta-1,3-diene-1,2,3,4- tetracarboxylate PCCP.

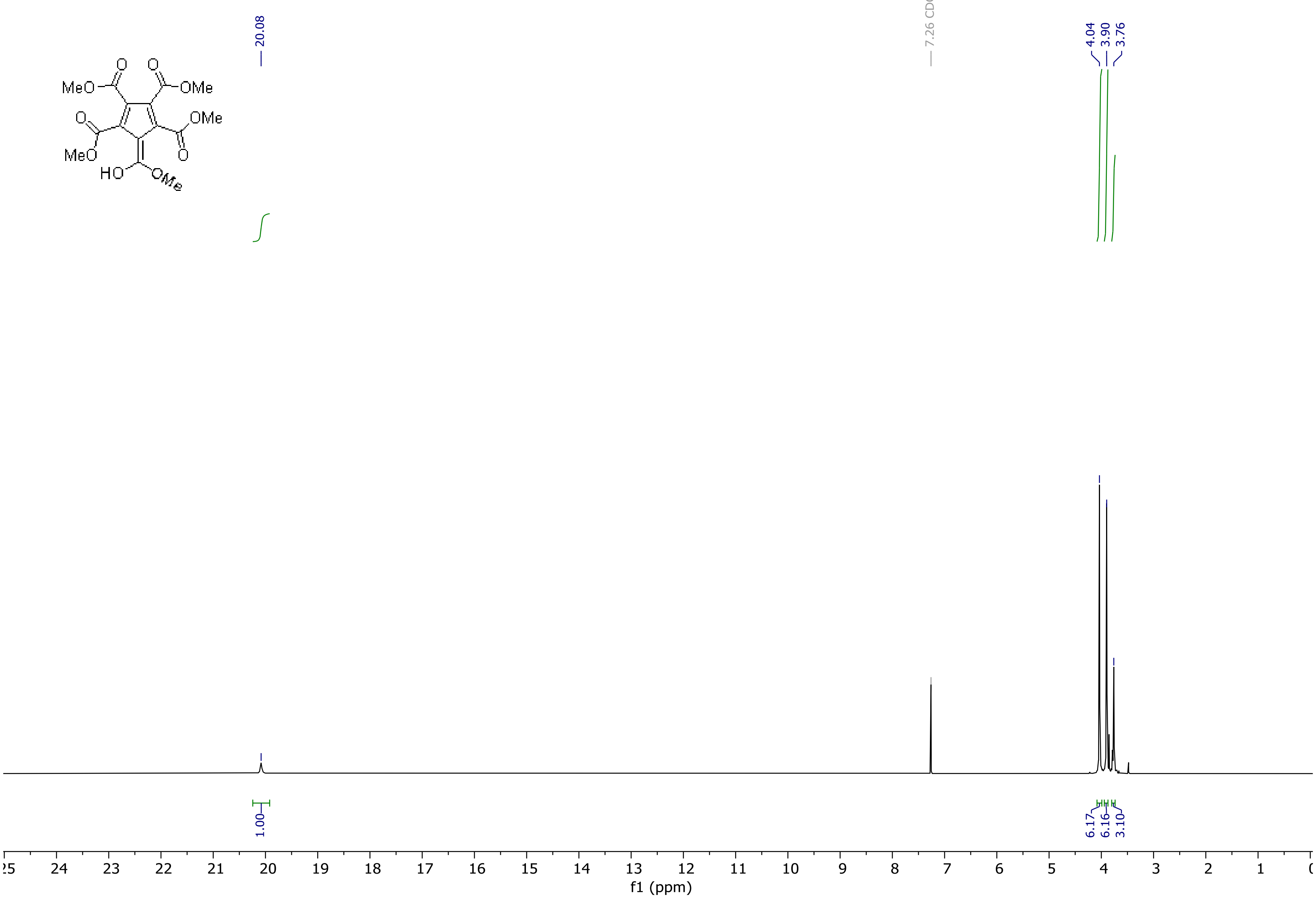

13C{1H} NMR: (101 MHz, CDCl3): Tetramethyl 5-(hydroxy(methoxy)methylene)cyclopenta-1,3-diene-1,2,3,4- tetracarboxylate PCCP.

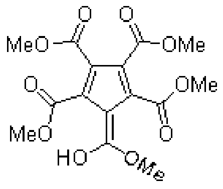

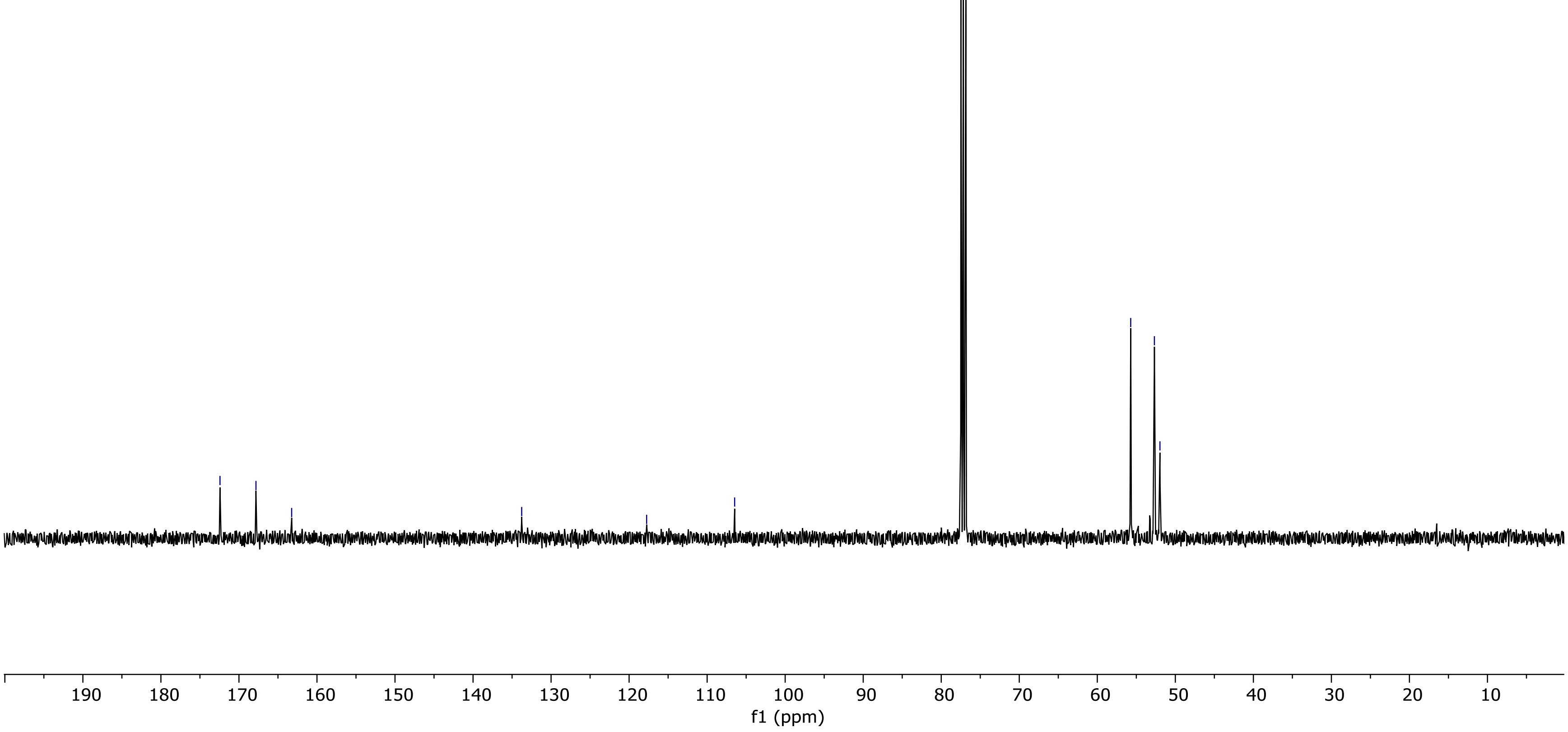

1H NMR (400 MHz, CDCl3): Pentakis((1R,2S,5R)-2-isopropyl-5-methylcyclohexyl) Cyclopenta-1,3-diene1,2,3,4,5-pentacarboxylate C1.

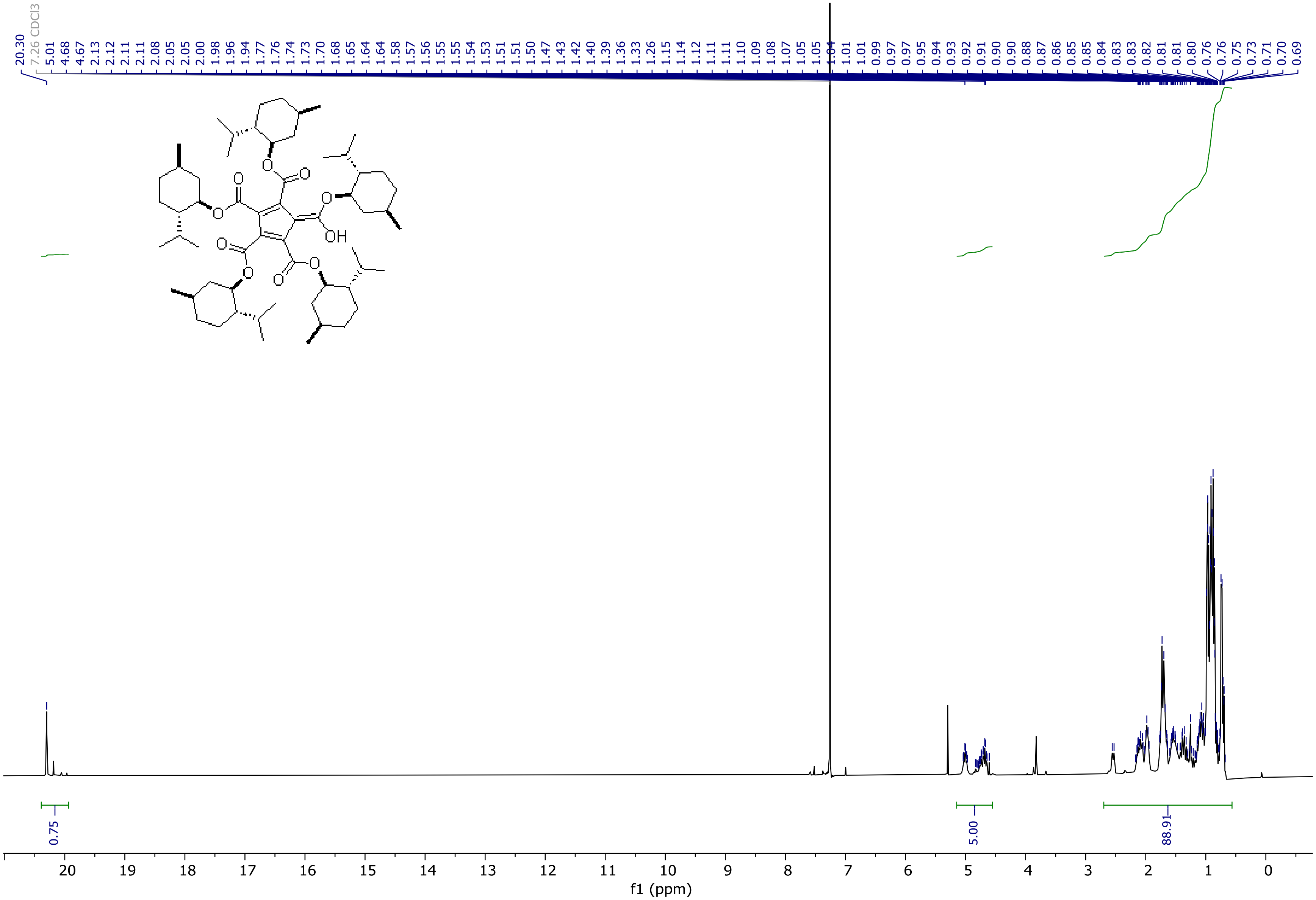

13C{1H} NMR: (101 MHz, CDCl3): Pentakis((1R,2S,5R)-2-isopropyl-5-methylcyclohexyl) Cyclopenta-1,3-diene1,2,3,4,5-pentacarboxylate C1.

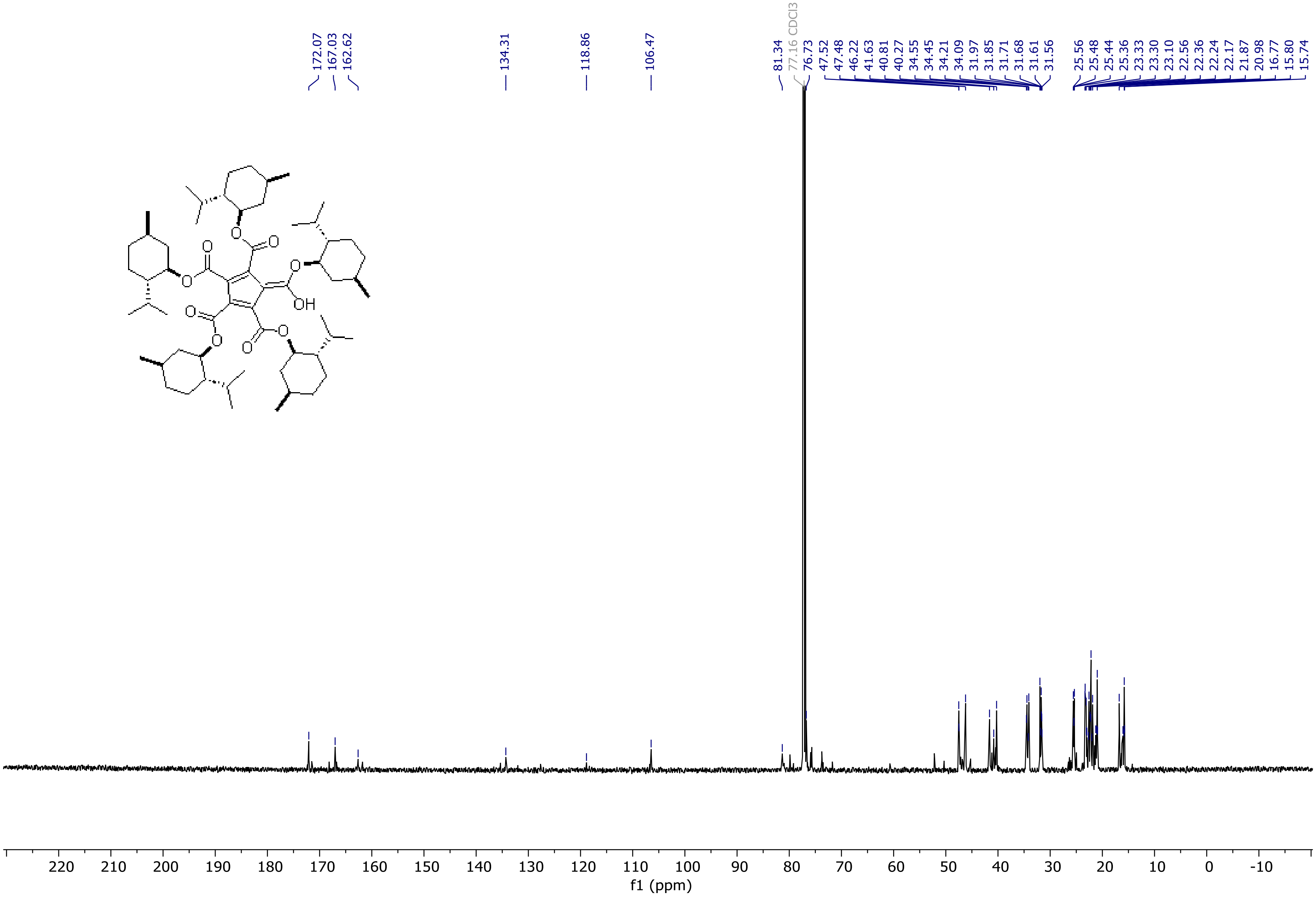

1H NMR (400 MHz, CDCl3): Pentakis((1R,2S)-2-phenyl-1-cyclohexyl) Cyclopenta-1,3-diene-1,2,3,4,5- pentacarboxylate C2.

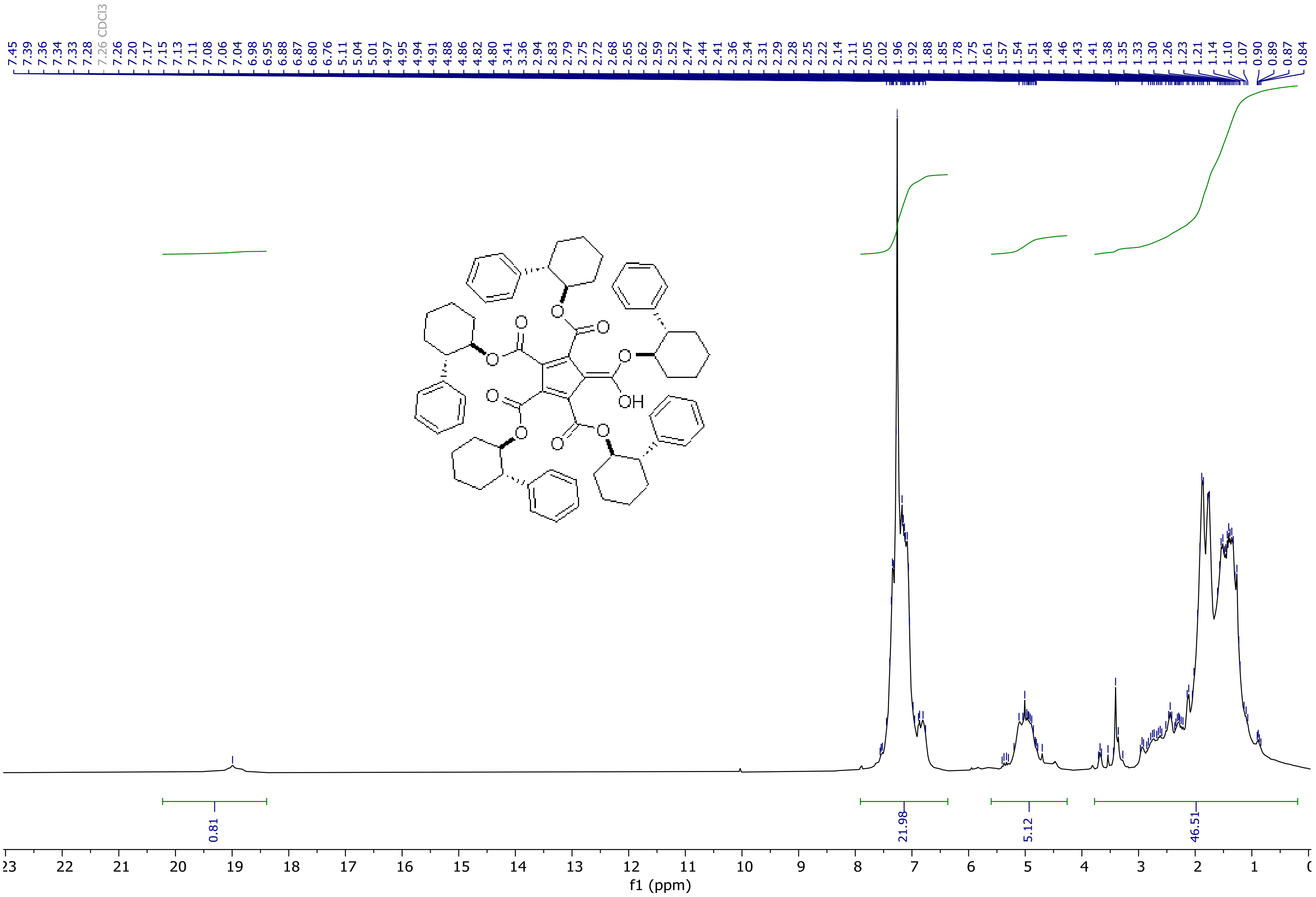

13C{1H} NMR: (101 MHz, CDCl3): Pentakis((1R,2S)-2-phenyl-1-cyclohexyl) Cyclopenta-1,3-diene-1,2,3,4,5- pentacarboxylate C2.

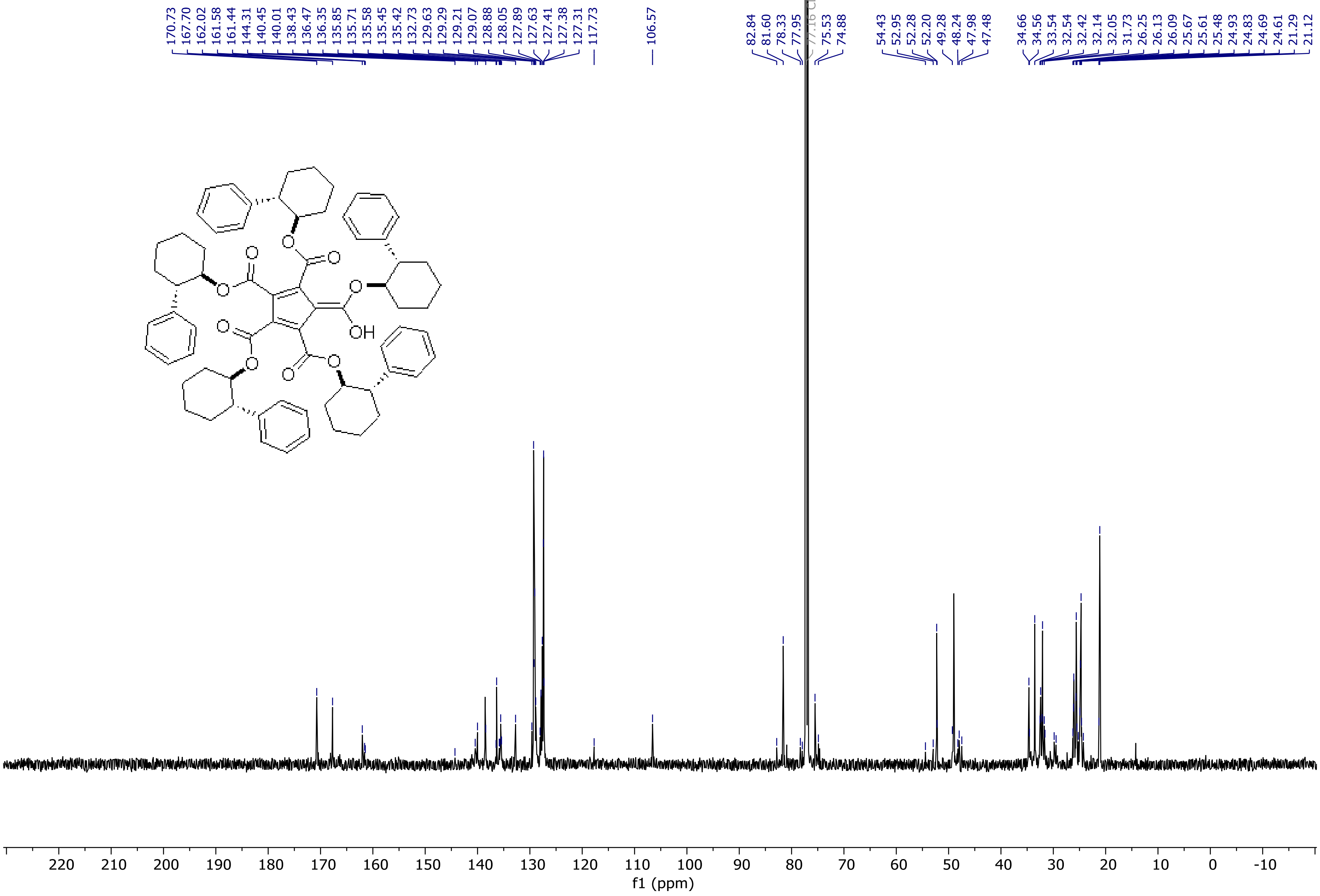

1H NMR (400 MHz, CDCl3): Pentakis((1R,2S)-2-(4-methoxyphenyl)-1-cyclohexyl) Cyclopenta-1,3-diene-1,2,3,4,5- pentacarboxylate C3.

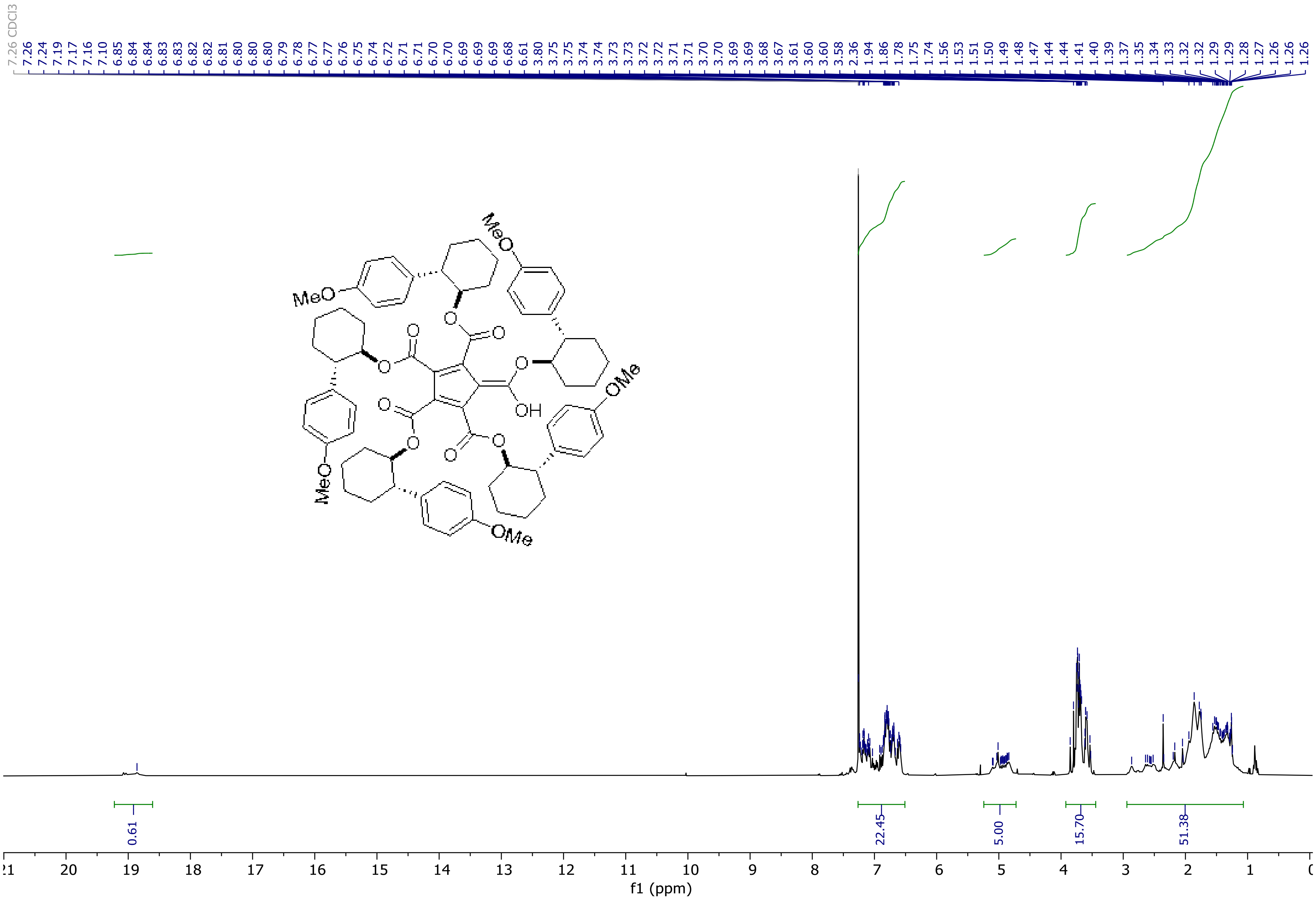

13C{1H} NMR: (101 MHz, CDCl3): Pentakis((1R,2S)-2-(4-methoxyphenyl)-1-cyclohexyl) Cyclopenta-1,3-diene-1,2,3,4,5- pentacarboxylate C3.

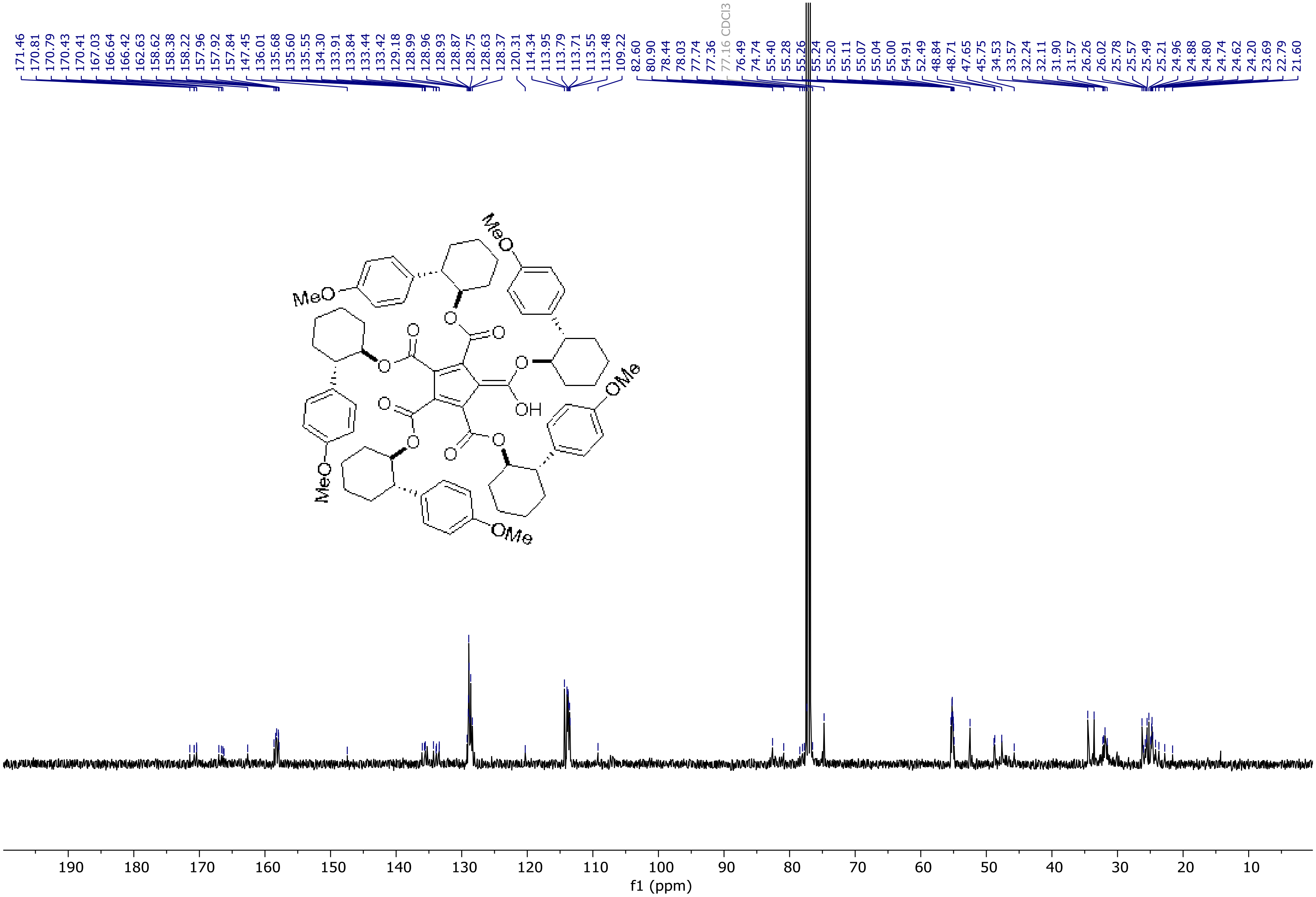

1H NMR (400 MHz, CDCl3): tetrakis((1R,2S)-2-(p-tolyl)cyclohexyl) 5-(hydroxy(((1R,2S)-2-(p-tolyl)cyclohexyl)oxy)methylene)cyclopenta-1,3-diene-1,2,3,4-tetracarboxylate C4.

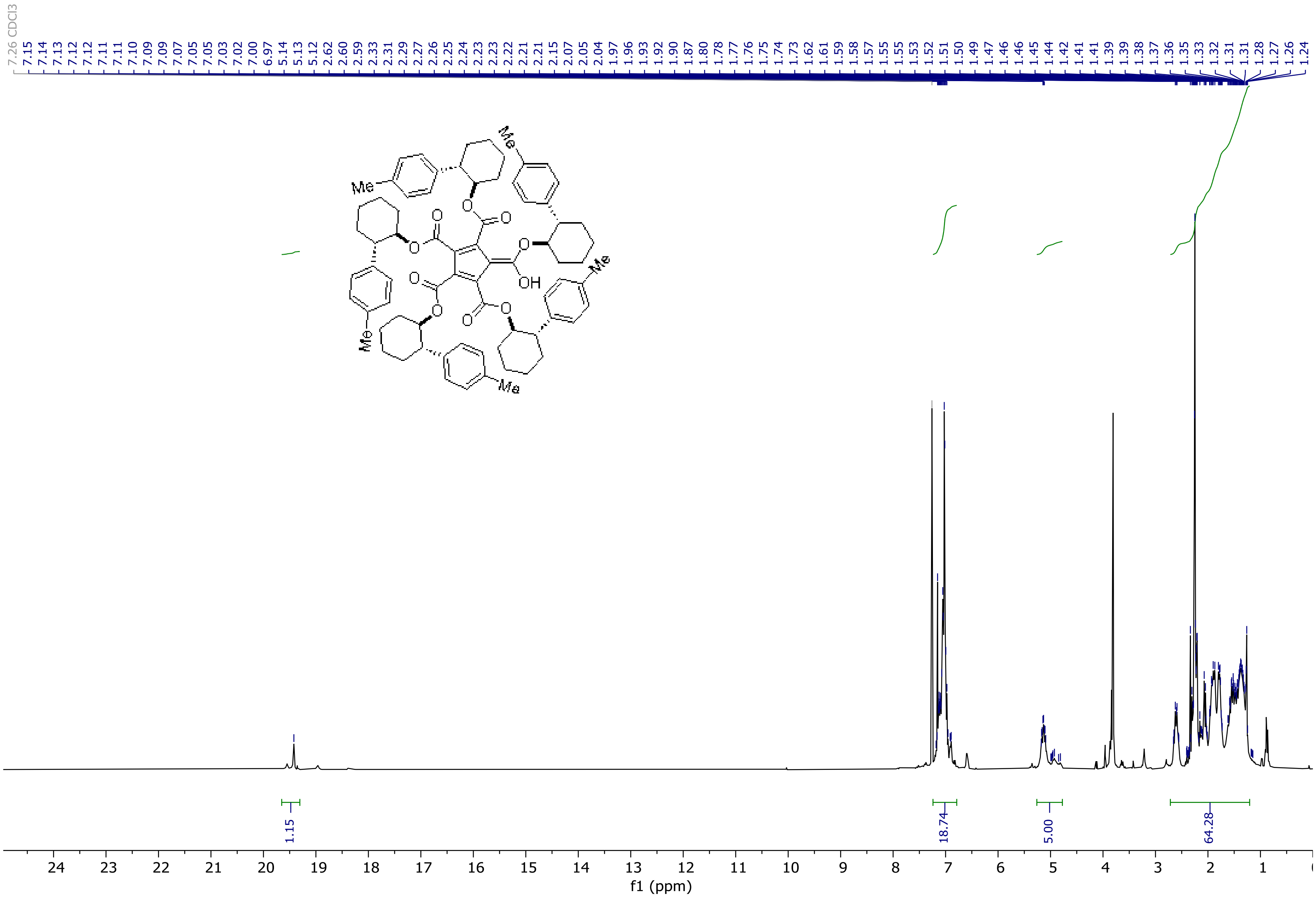

13C{1H} NMR: (101 MHz, CDCl3): Tetrakis((1R,2S)-2-(p-tolyl)cyclohexyl) 5-(hydroxy(((1R,2S)-2-(p-tolyl)cyclohexyl)oxy)methylene)cyclopenta-1,3-diene-1,2,3,4-tetracarboxylate C4.

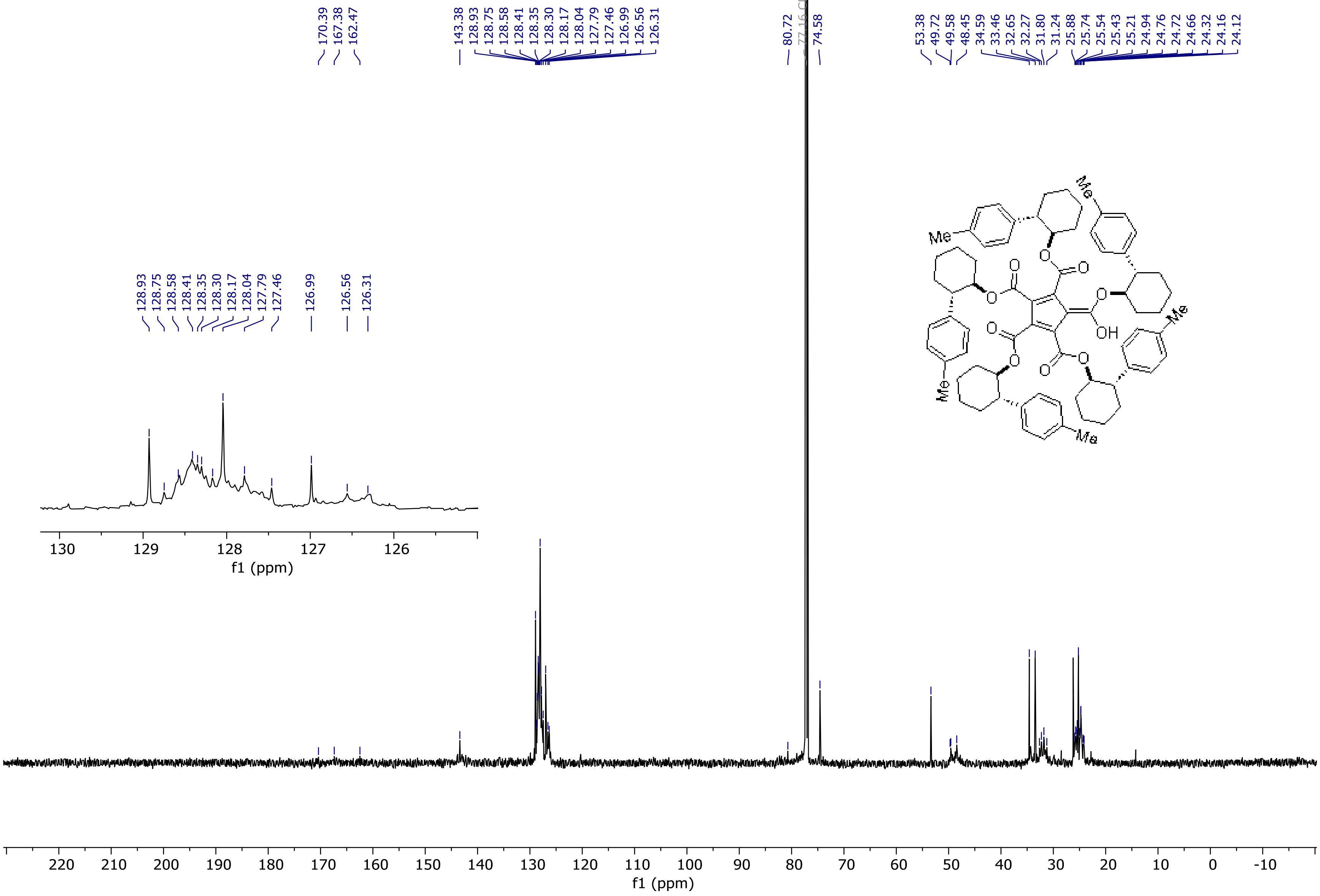

1H NMR (400 MHz, CDCl3): 1,2,3,4,5-Pentacarbo-(−)-isopinocampheoxycyclopentadiene C5.

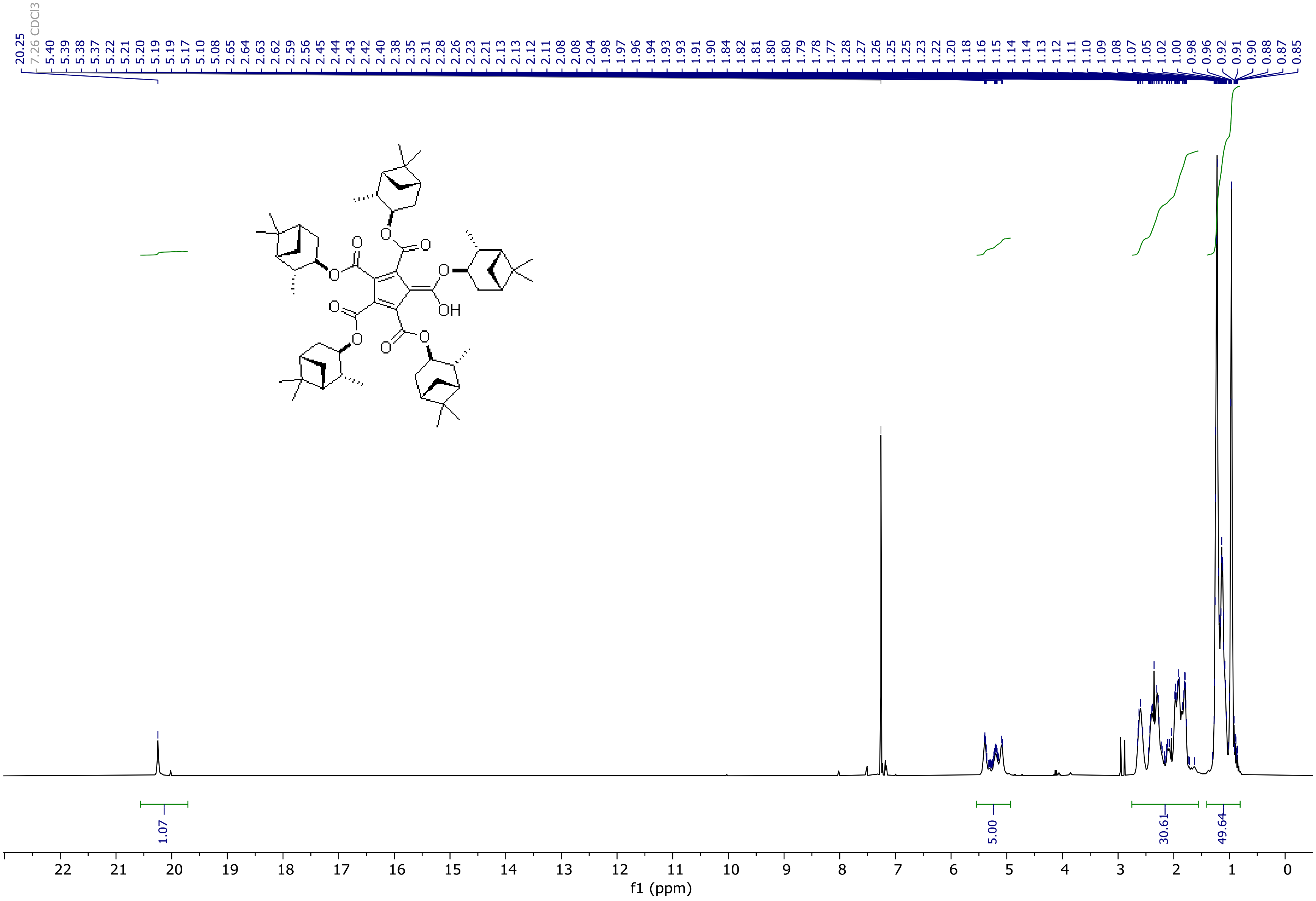

13C{1H} NMR: (101 MHz, CDCl3): 1,2,3,4,5-Pentacarbo-(−)-isopinocampheoxycyclopentadiene C5.

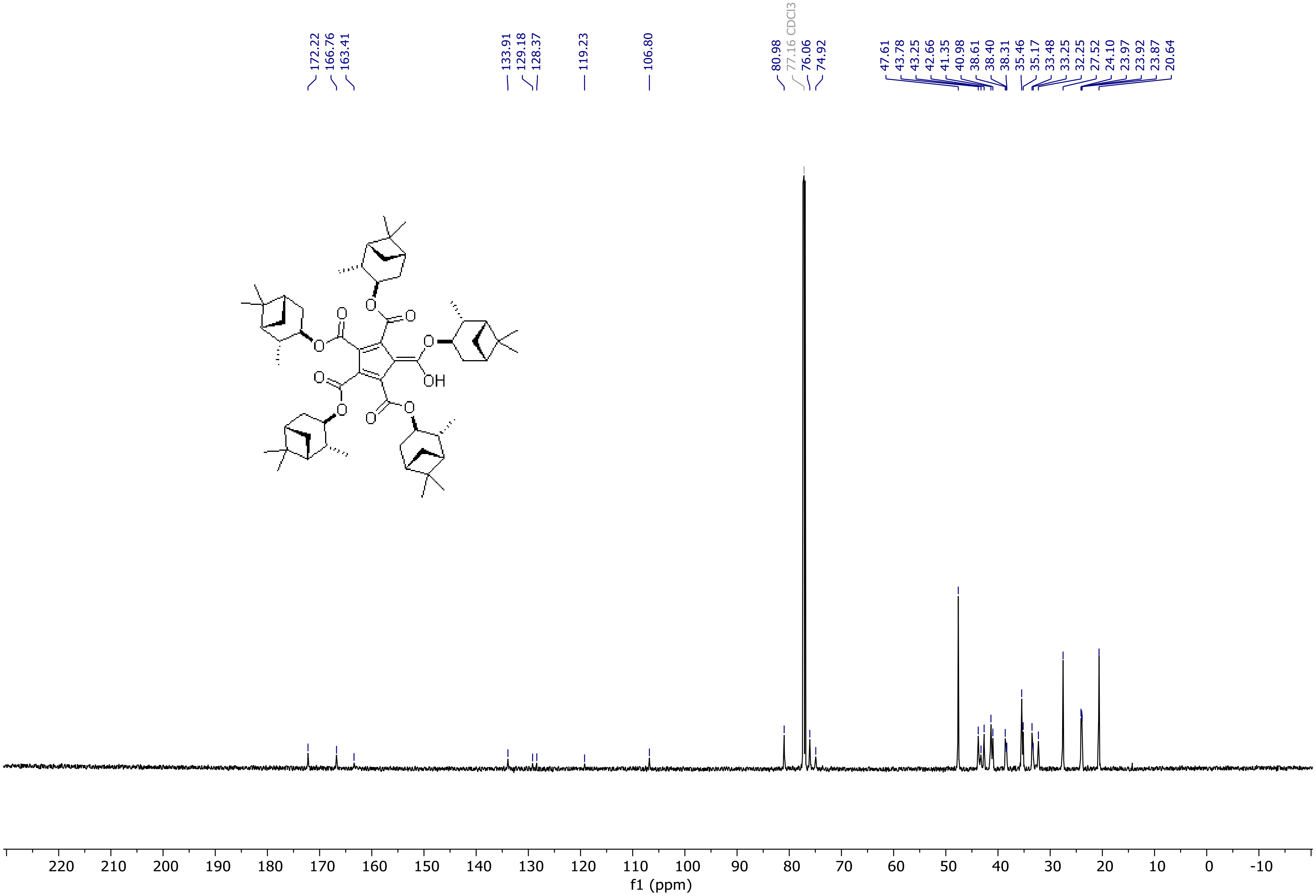

1H NMR (400 MHz, CDCl3): 3-methyl-2-(1H-pyrrol-1-yl)aniline 1b.

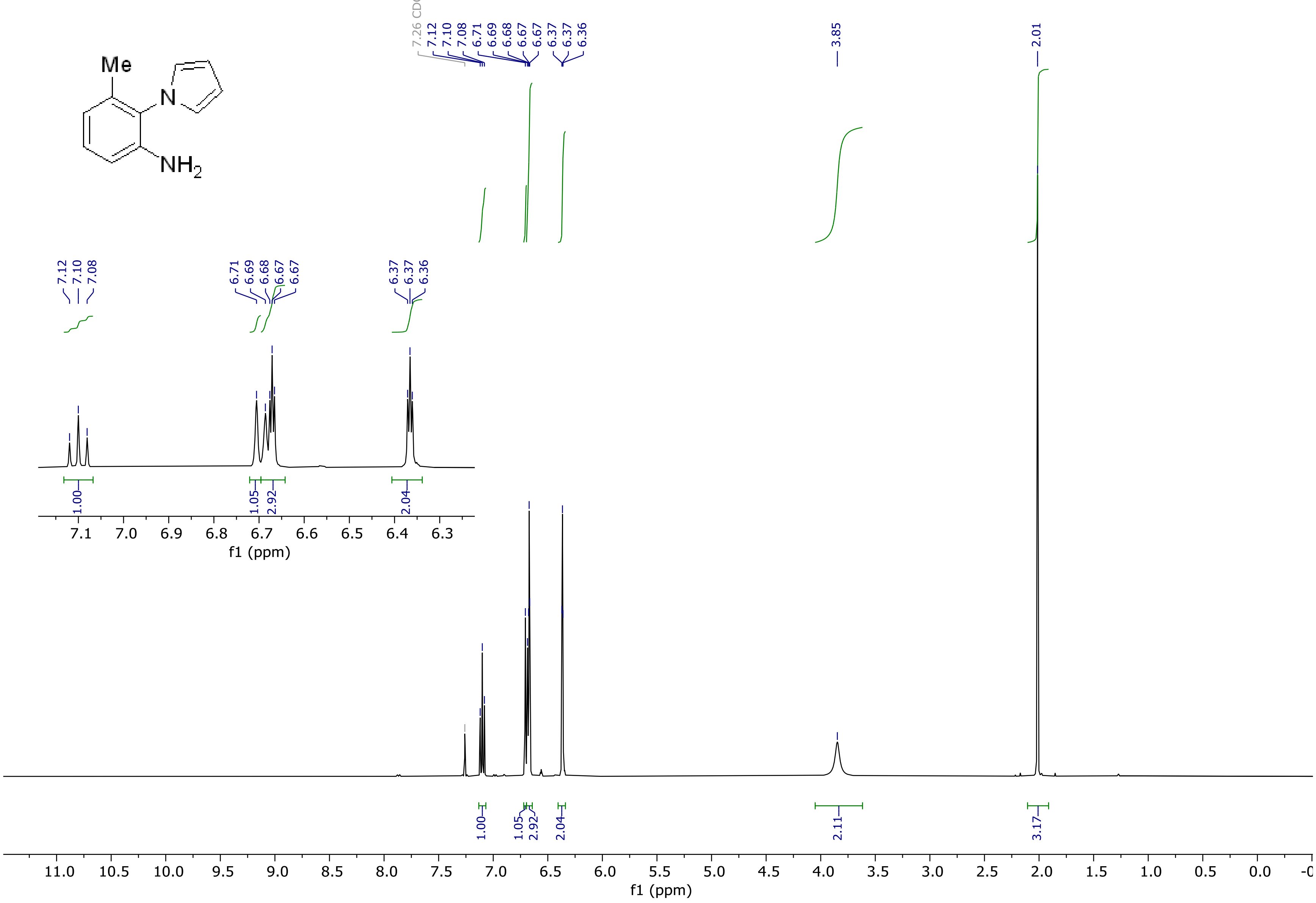

13C{1H} NMR: (101 MHz, CDCl3): 3-methyl-2-(1H-pyrrol-1-yl)aniline 1b.

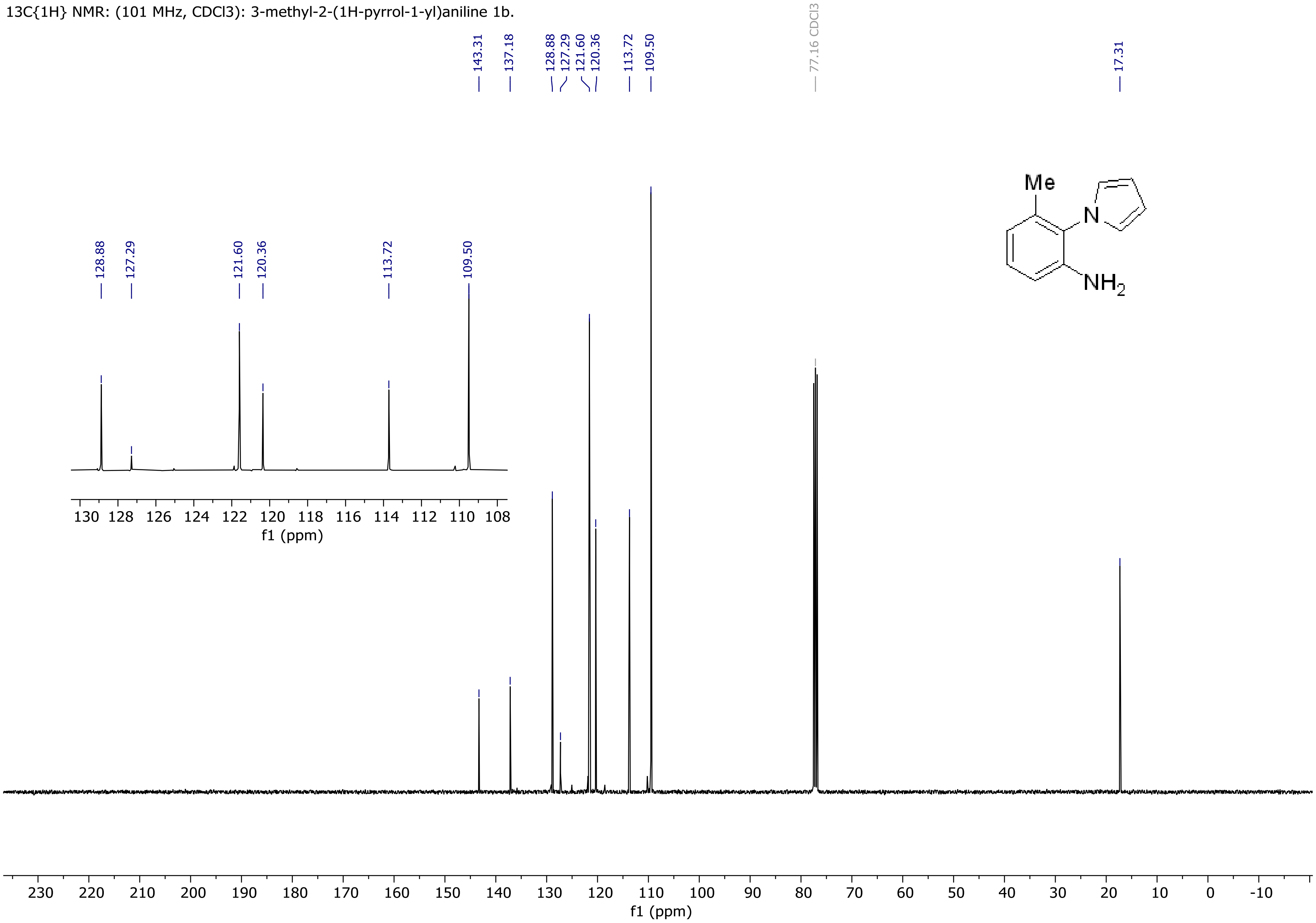

1H NMR (400 MHz, CDCl3): 4-methoxy-2-(1H-pyrrol-1-yl)aniline 1c.

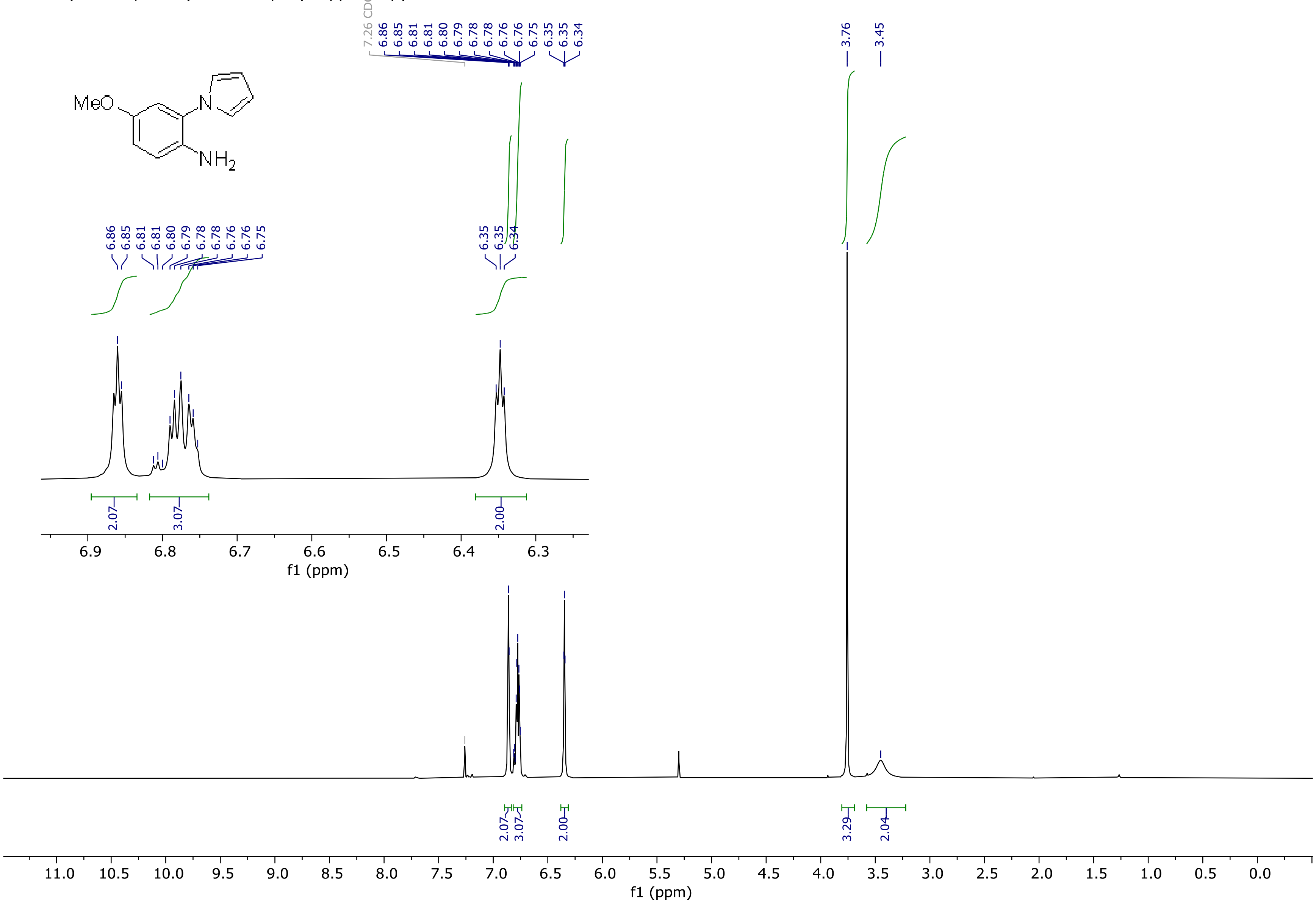

13C{1H} NMR: (101 MHz, CDCl3): 4-methoxy-2-(1H-pyrrol-1-yl)aniline 1c.

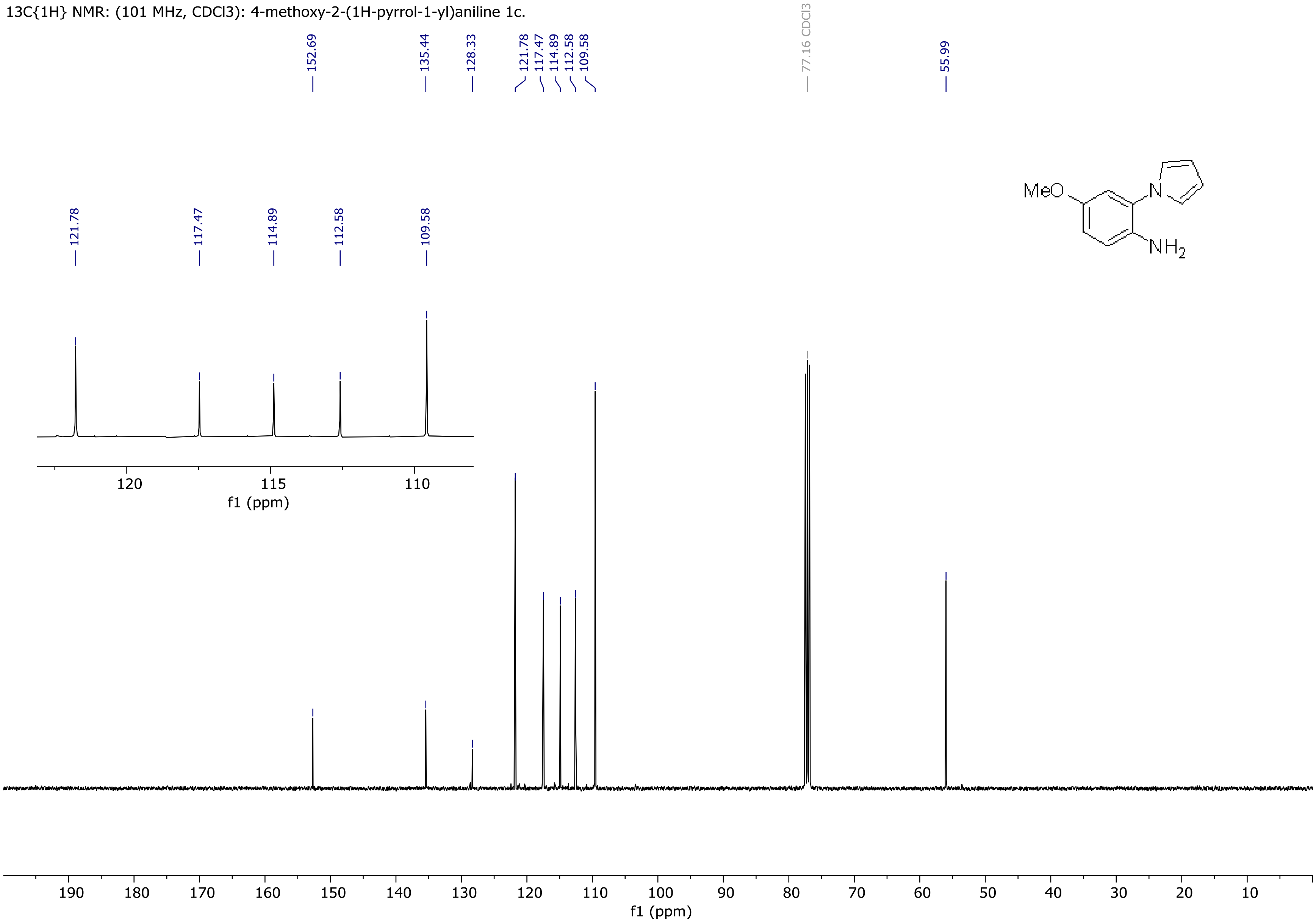

1H NMR (400 MHz, CDCl3): 4-chloro-2-(1H-pyrrol-1-yl)aniline 1d.

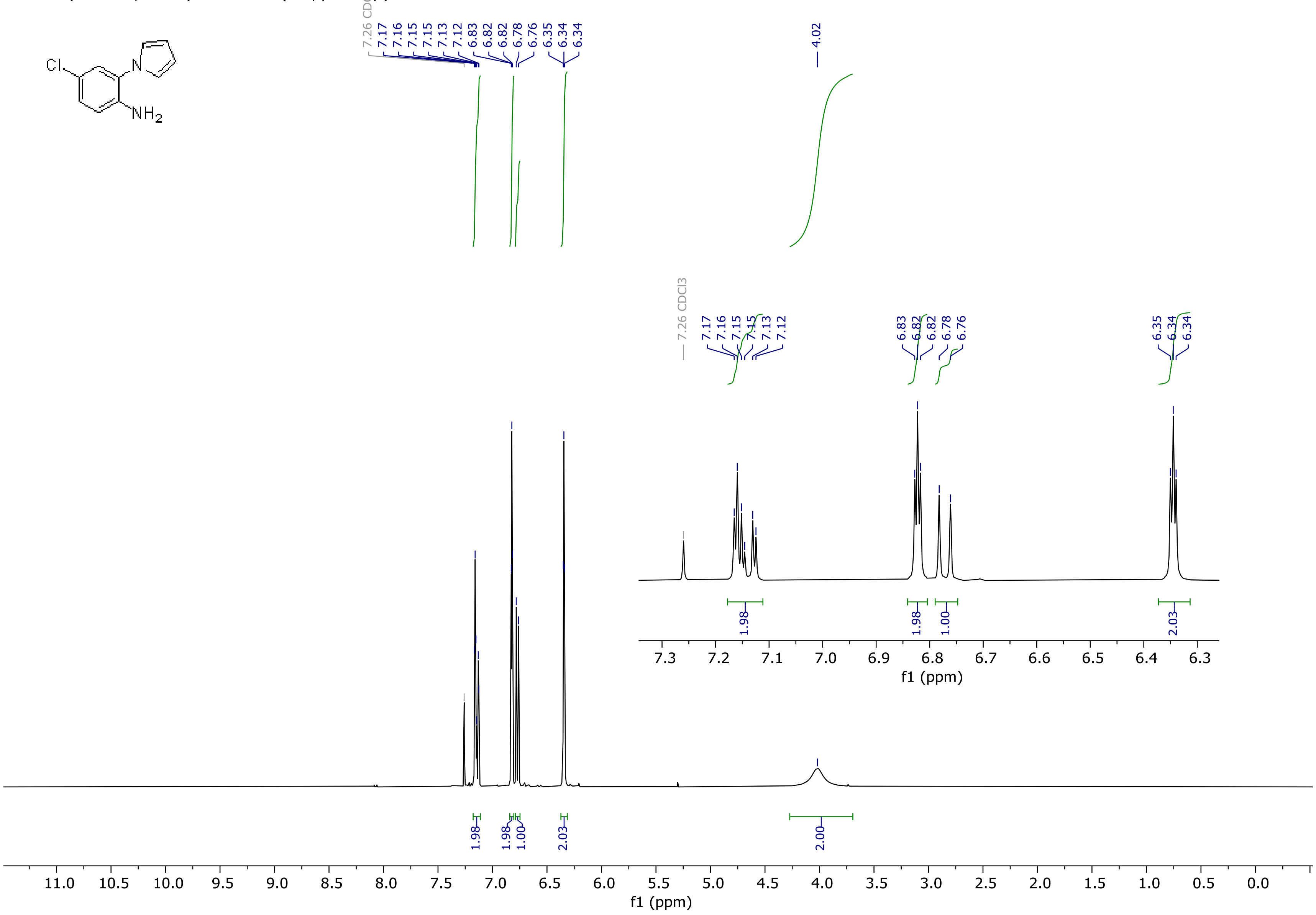

13C{1H} NMR: (101 MHz, CDCl3): 4-chloro-2-(1H-pyrrol-1-yl)aniline 1d.

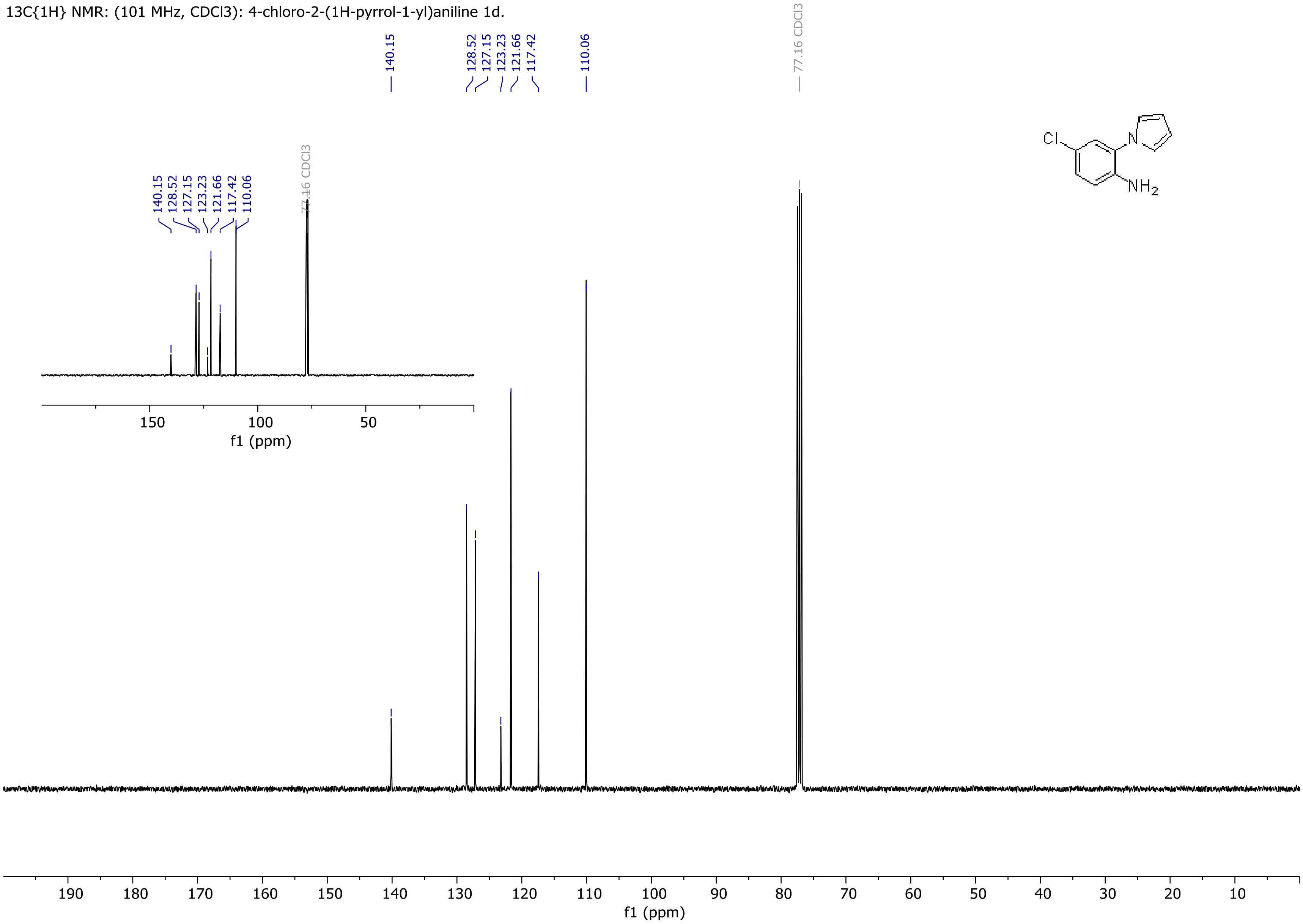

1H NMR (400 MHz, CDCl3): 3-methoxy-2-(1H-pyrrol-1-yl)aniline 1e.

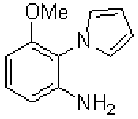

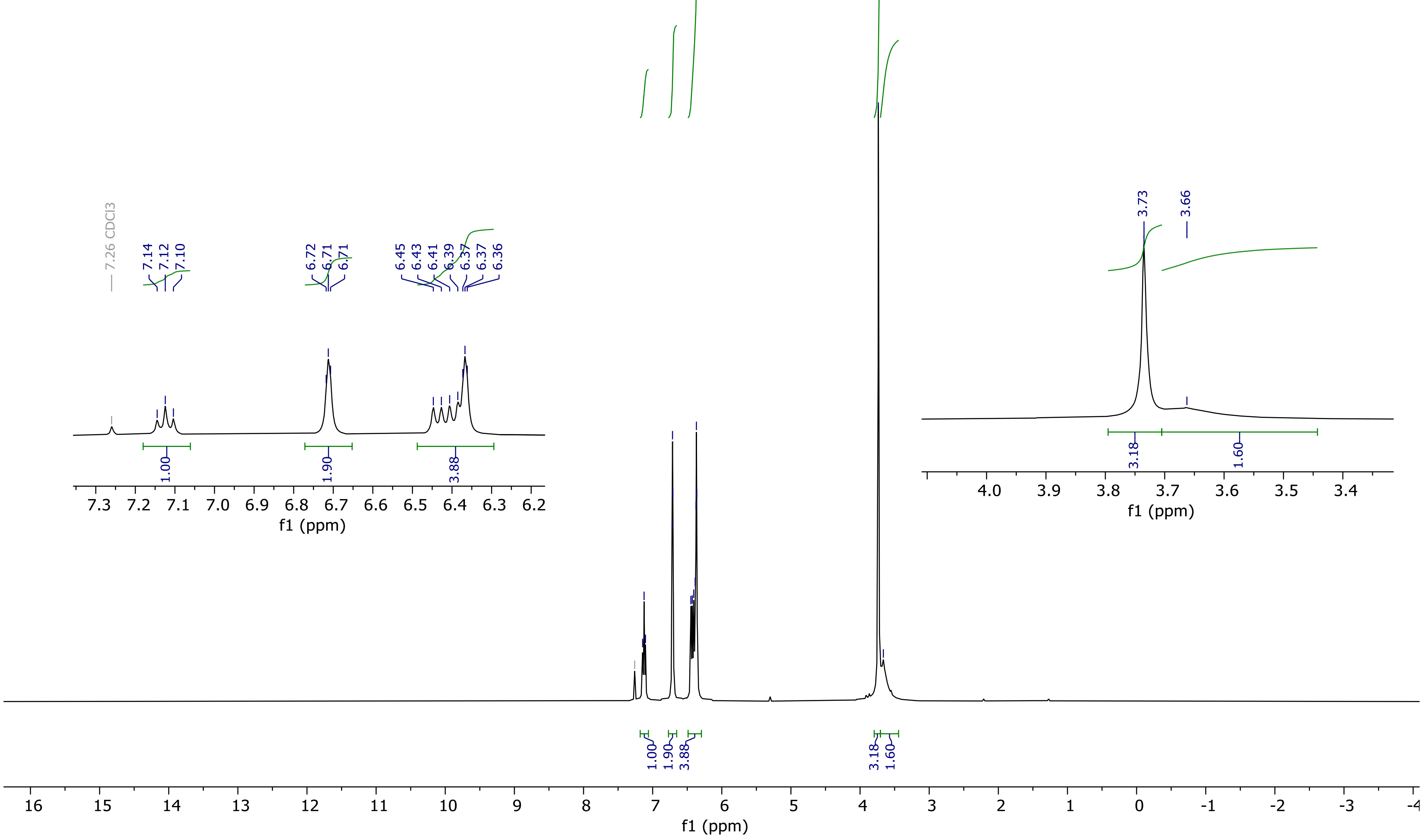

13C{1H} NMR: (101 MHz, CDCl3): 3-methoxy-2-(1H-pyrrol-1-yl)aniline 1e.

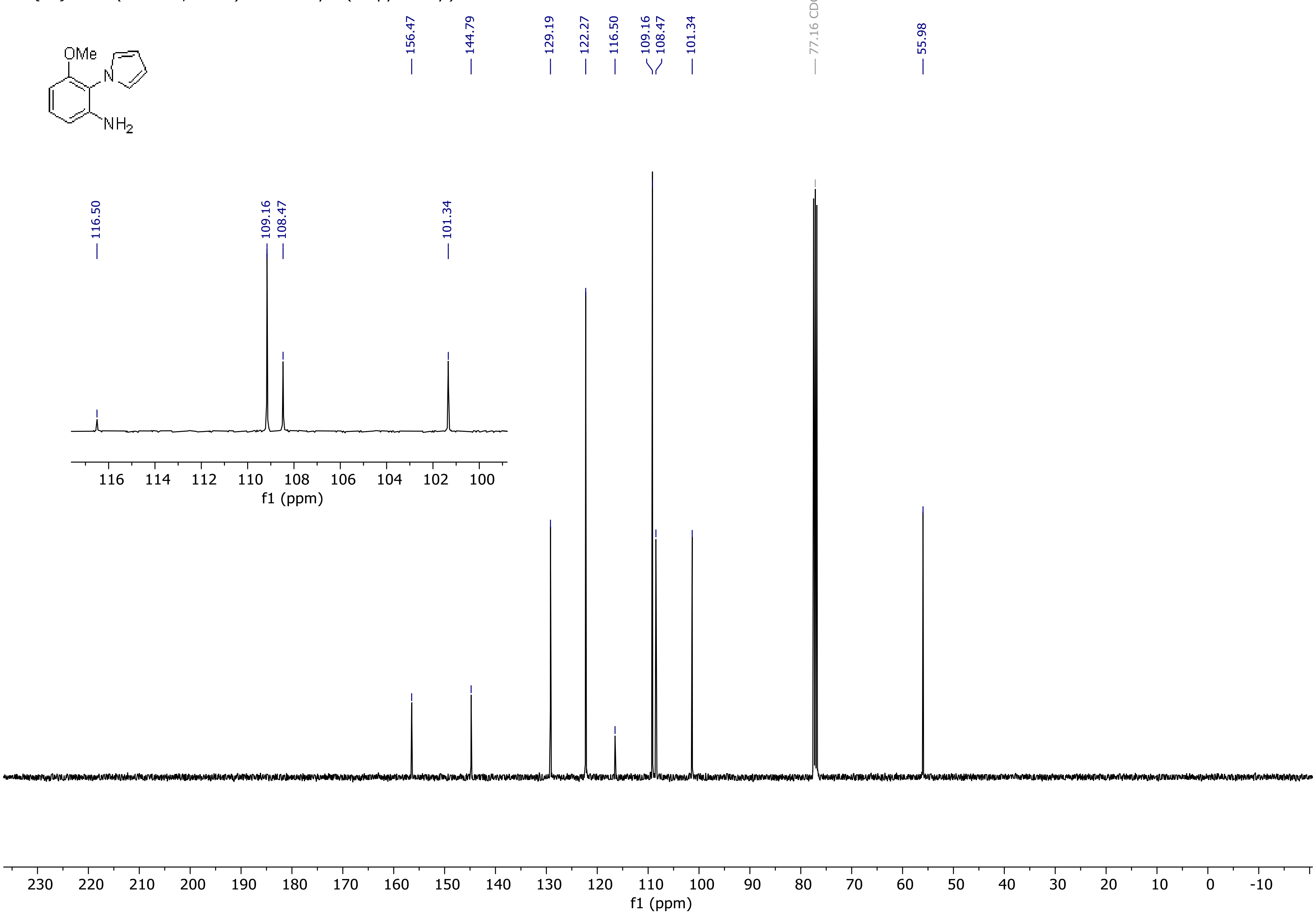

1H NMR (400 MHz, CDCl3): 2-(1H-pyrrol-1-yl)-5-(trifluoromethyl)aniline 1f.

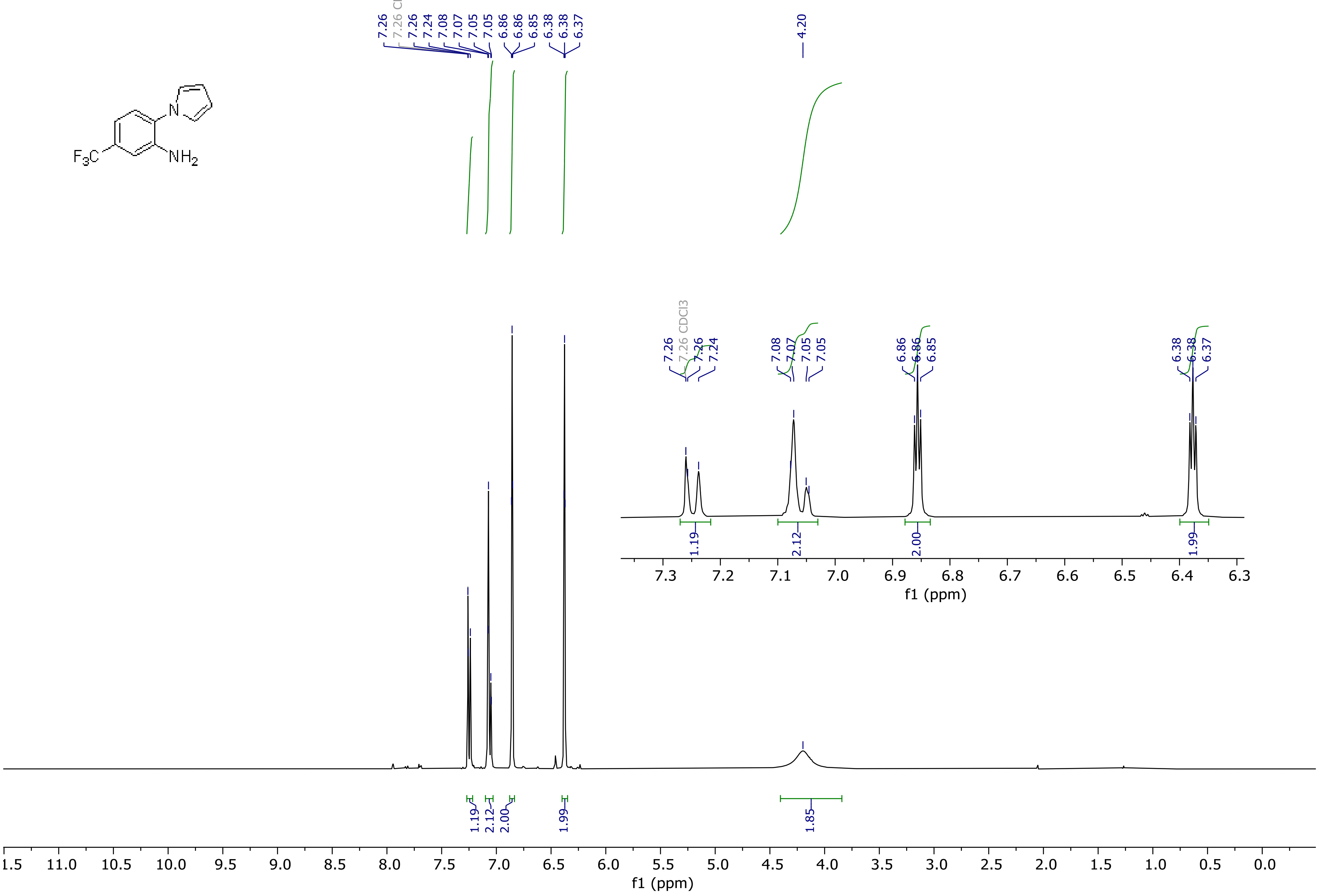

13C{1H} NMR: (101 MHz, CDCl3): 2-(1H-pyrrol-1-yl)-5-(trifluoromethyl)aniline 1f.

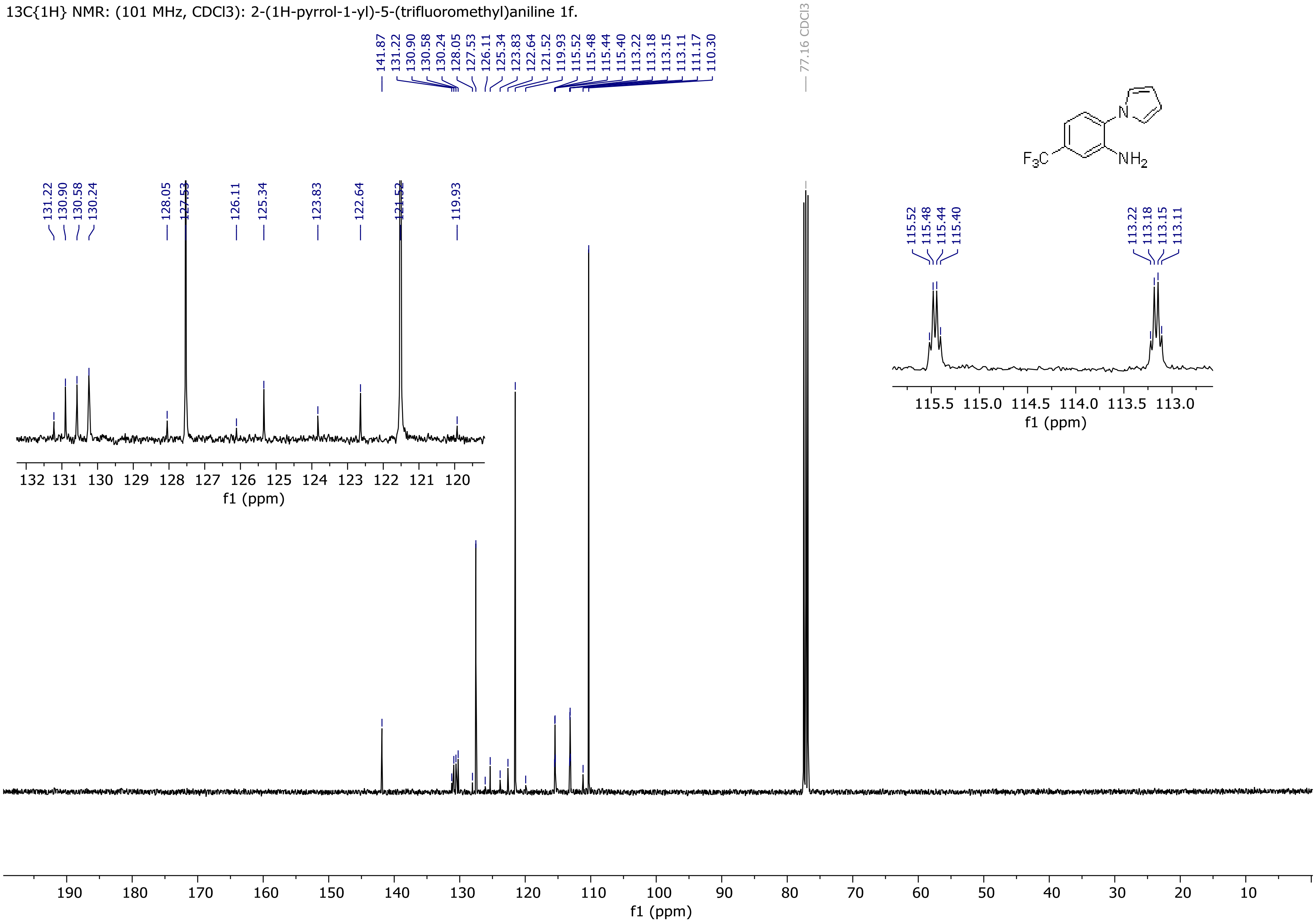

19F NMR: (376 MHz, CDCl3): 2-(1H-pyrrol-1-yl)-5-(trifluoromethyl)aniline 1f.

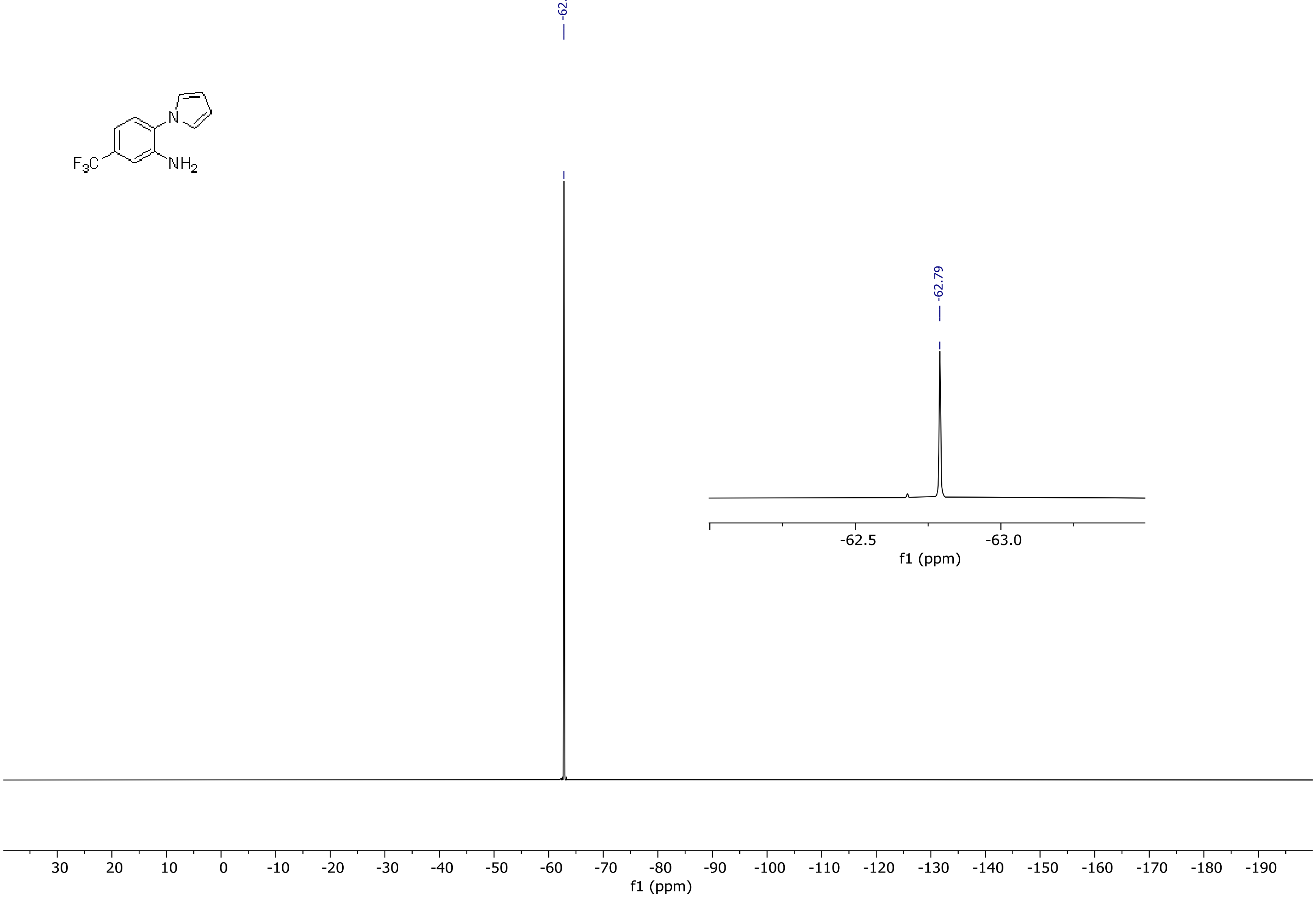

1H NMR (400 MHz, CDCl3): 5-bromo-2-(1H-pyrrol-1-yl)aniline 1g.

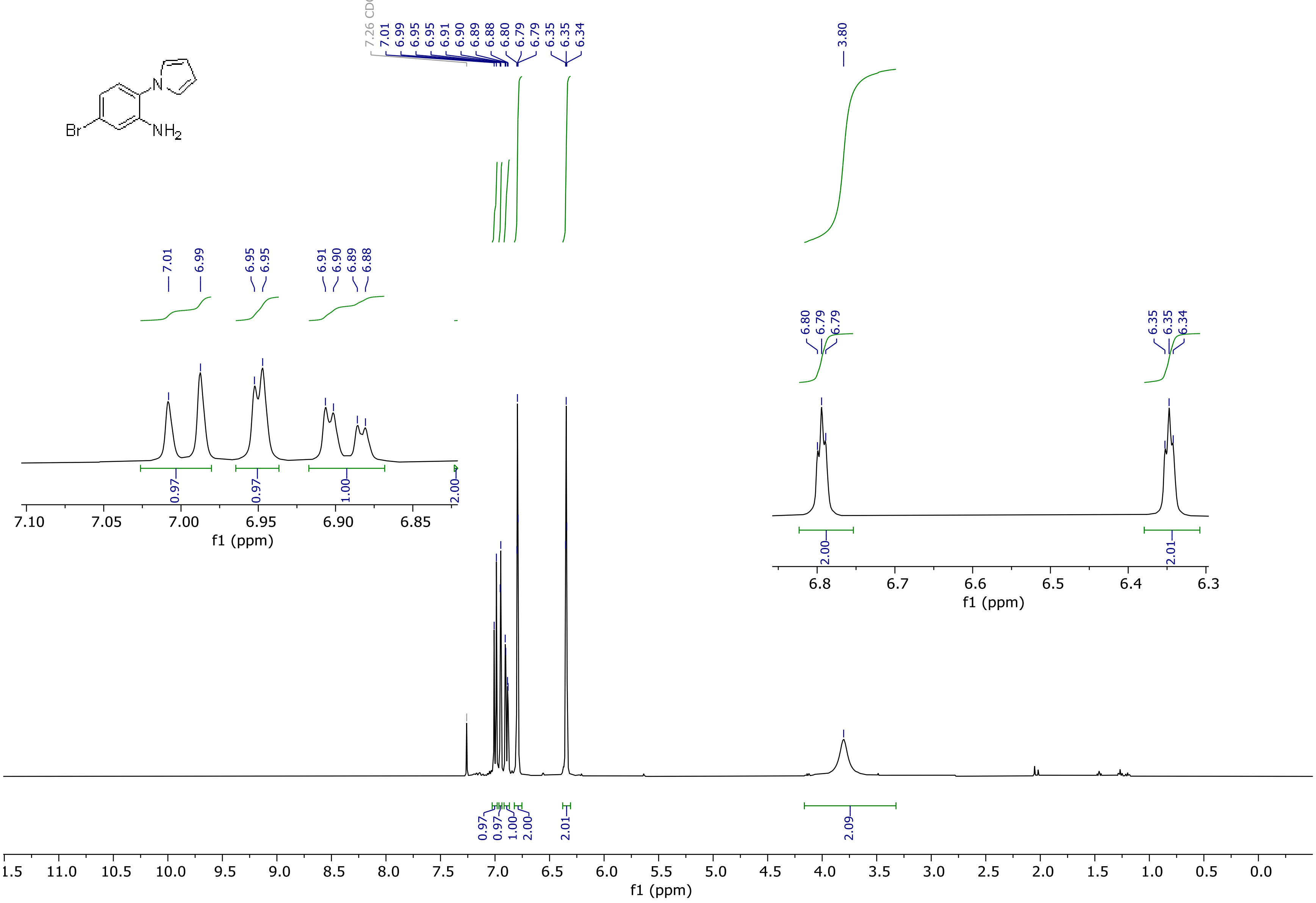

13C{1H} NMR: (101 MHz, CDCl3): 5-bromo-2-(1H-pyrrol-1-yl)aniline 1g.

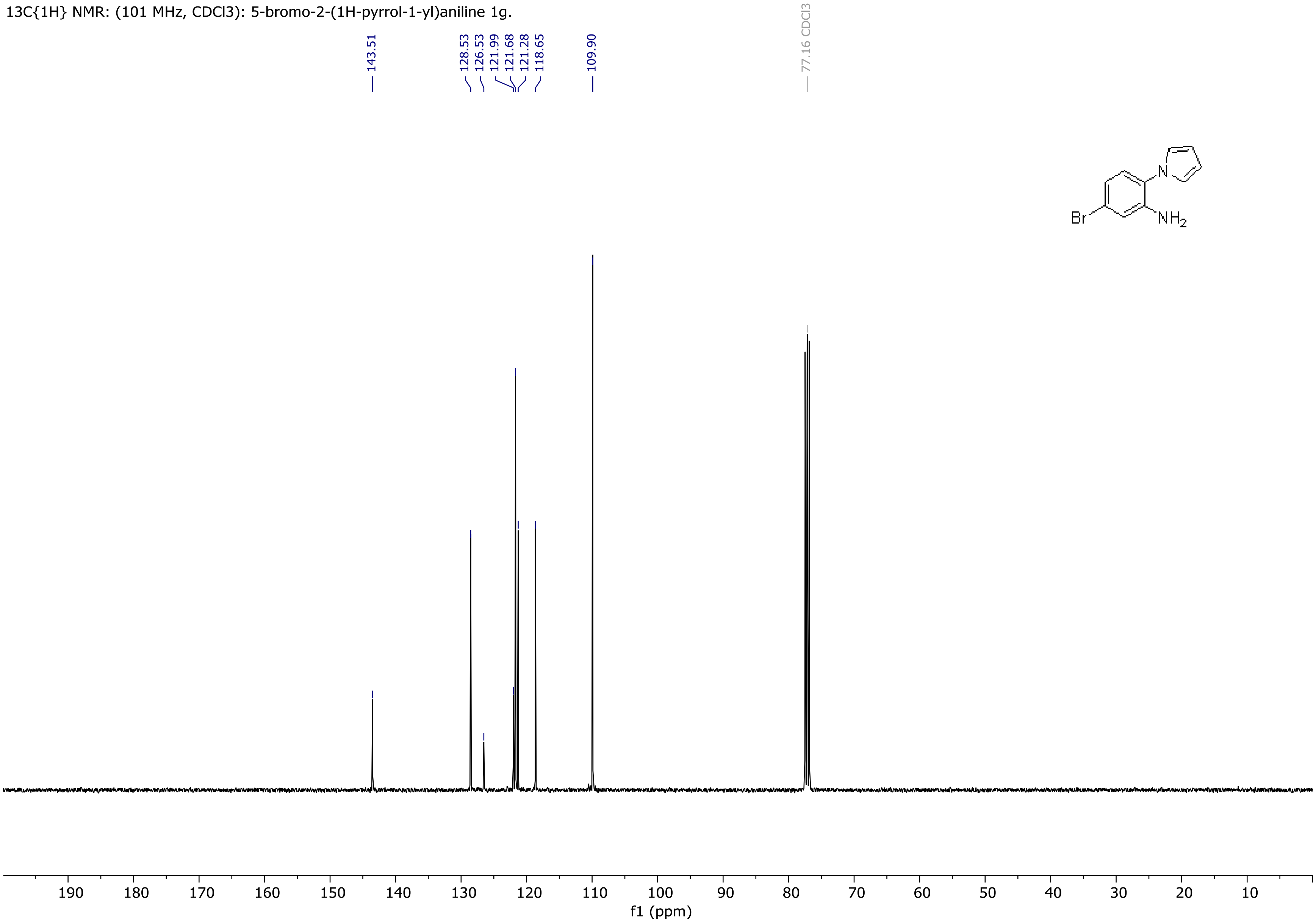

1H NMR (400 MHz, CDCl3): N-(2-(1H-pyrrol-1-yl)phenyl)acetamide 1h.

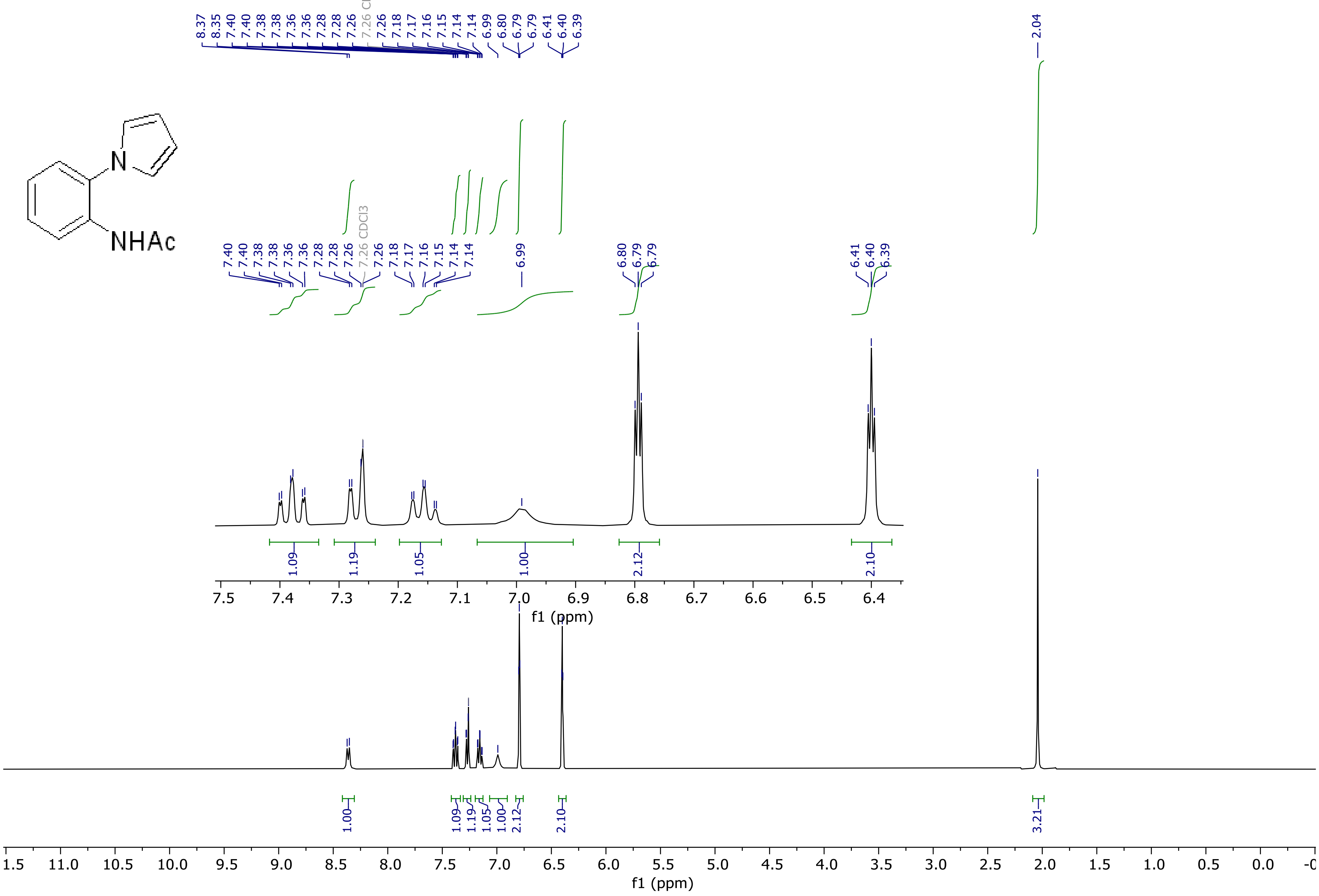

13C{1H} NMR: (101 MHz, CDCl3): N-(2-(1H-pyrrol-1-yl)phenyl)acetamide 1h.

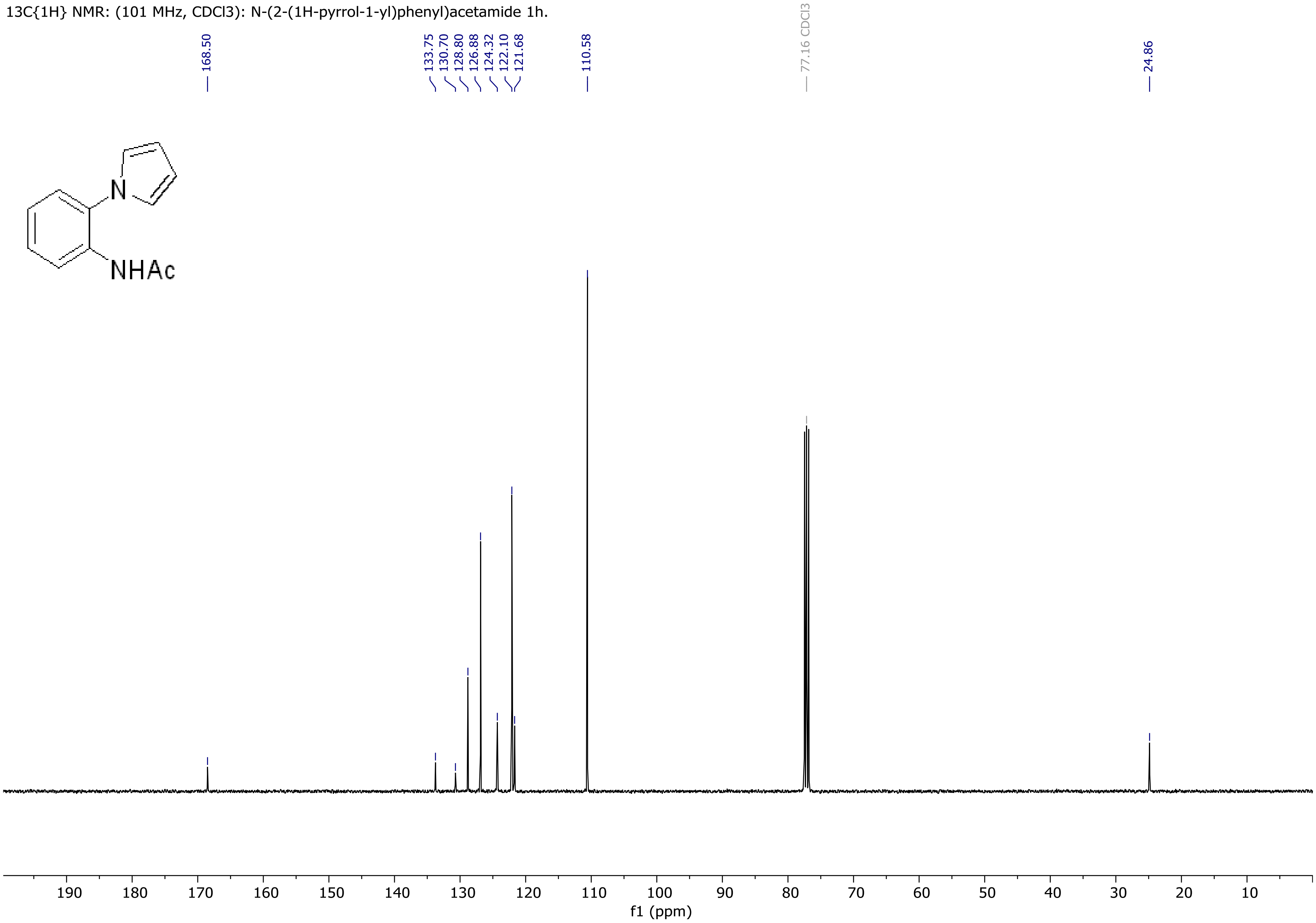

1H NMR (400 MHz, CDCl3): 4-hydroxy-3',5'-dimethoxy-[1,1'-biphenyl]-3-carbaldehyde 2i.

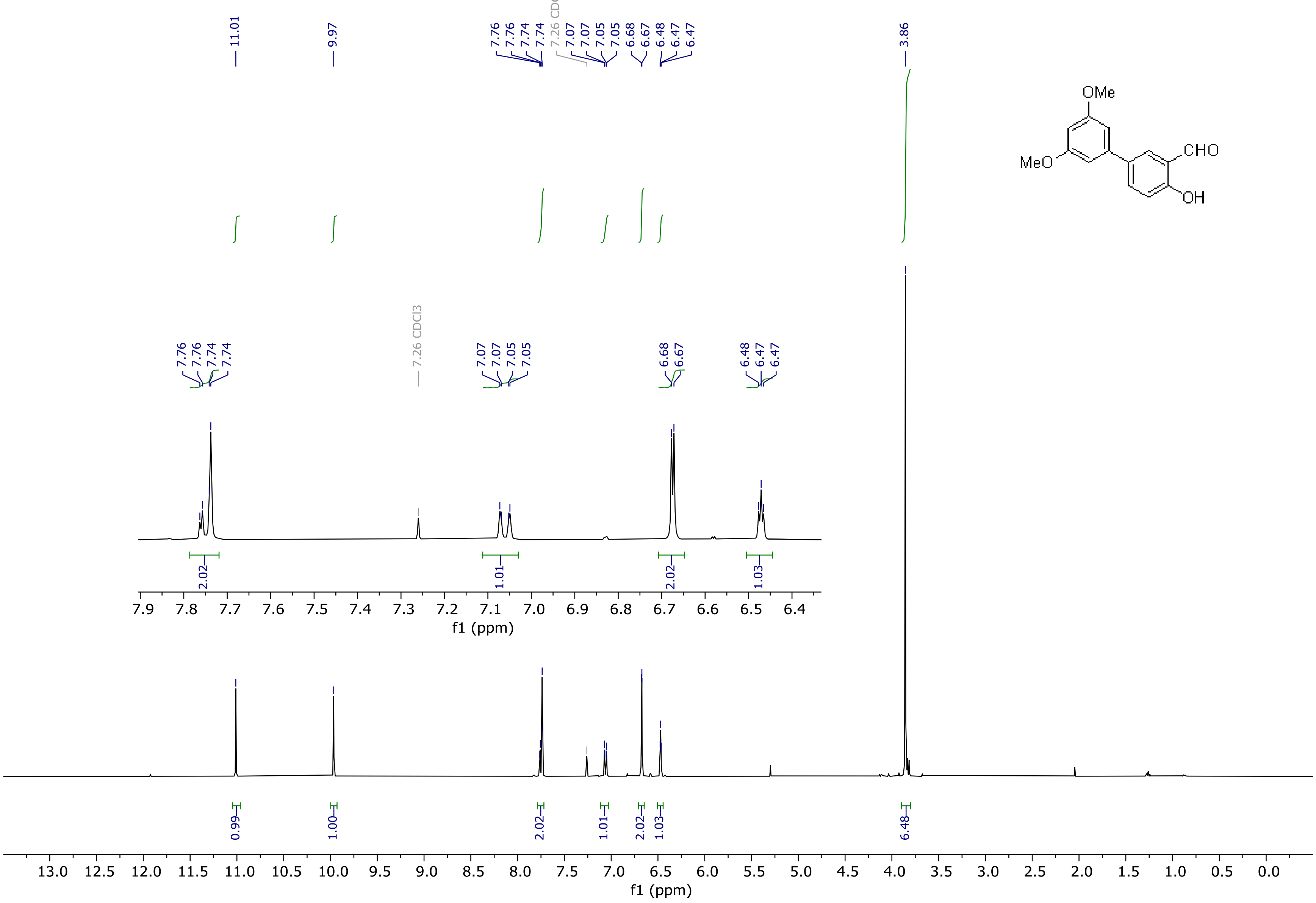

13C{1H} NMR: (101 MHz, CDCl3): 4-hydroxy-3',5'-dimethoxy-[1,1'-biphenyl]-3-carbaldehyde 2i.

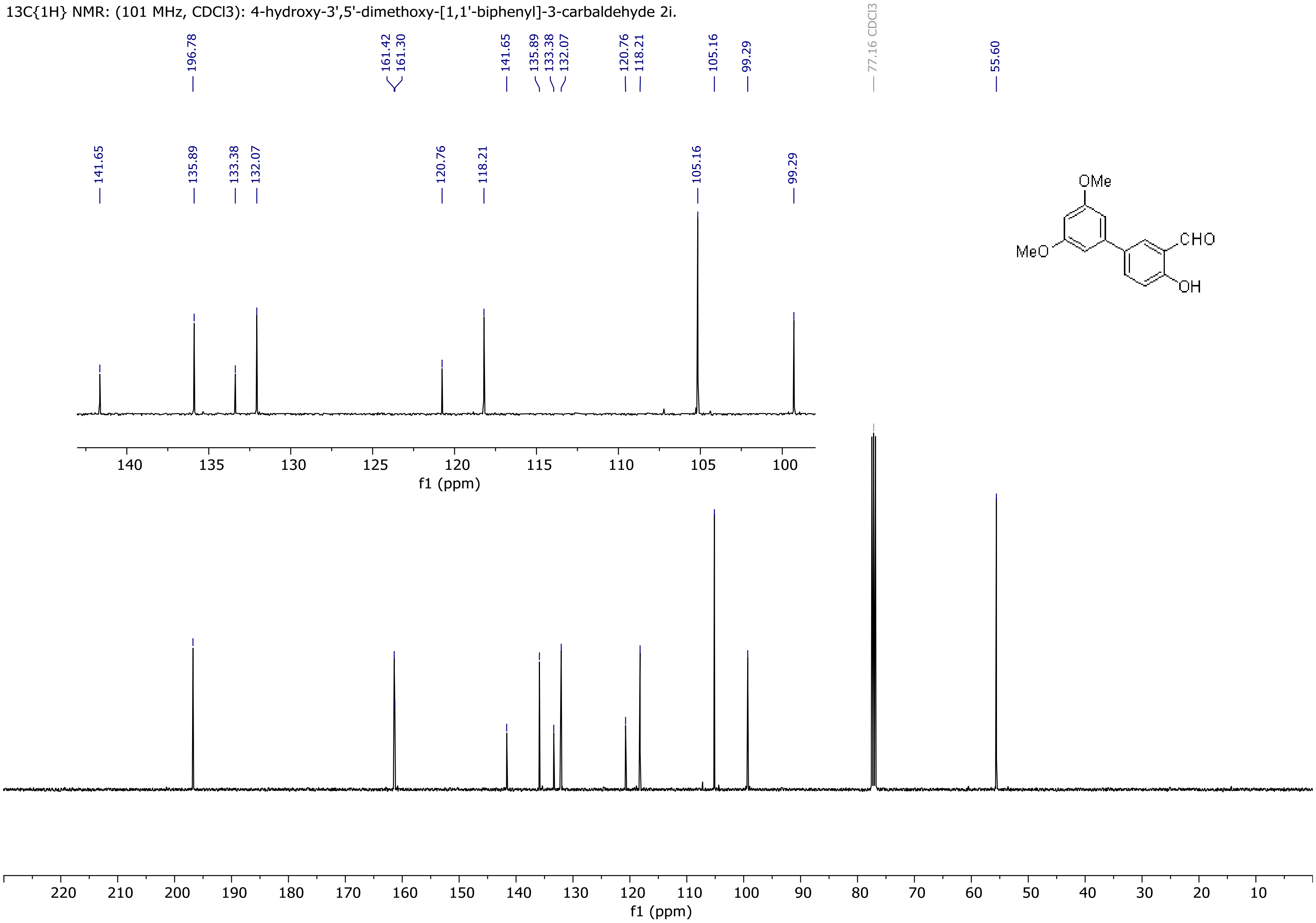

1H NMR (400 MHz, CDCl3): 3-hydroxy-2-naphthaldehyde 2s.

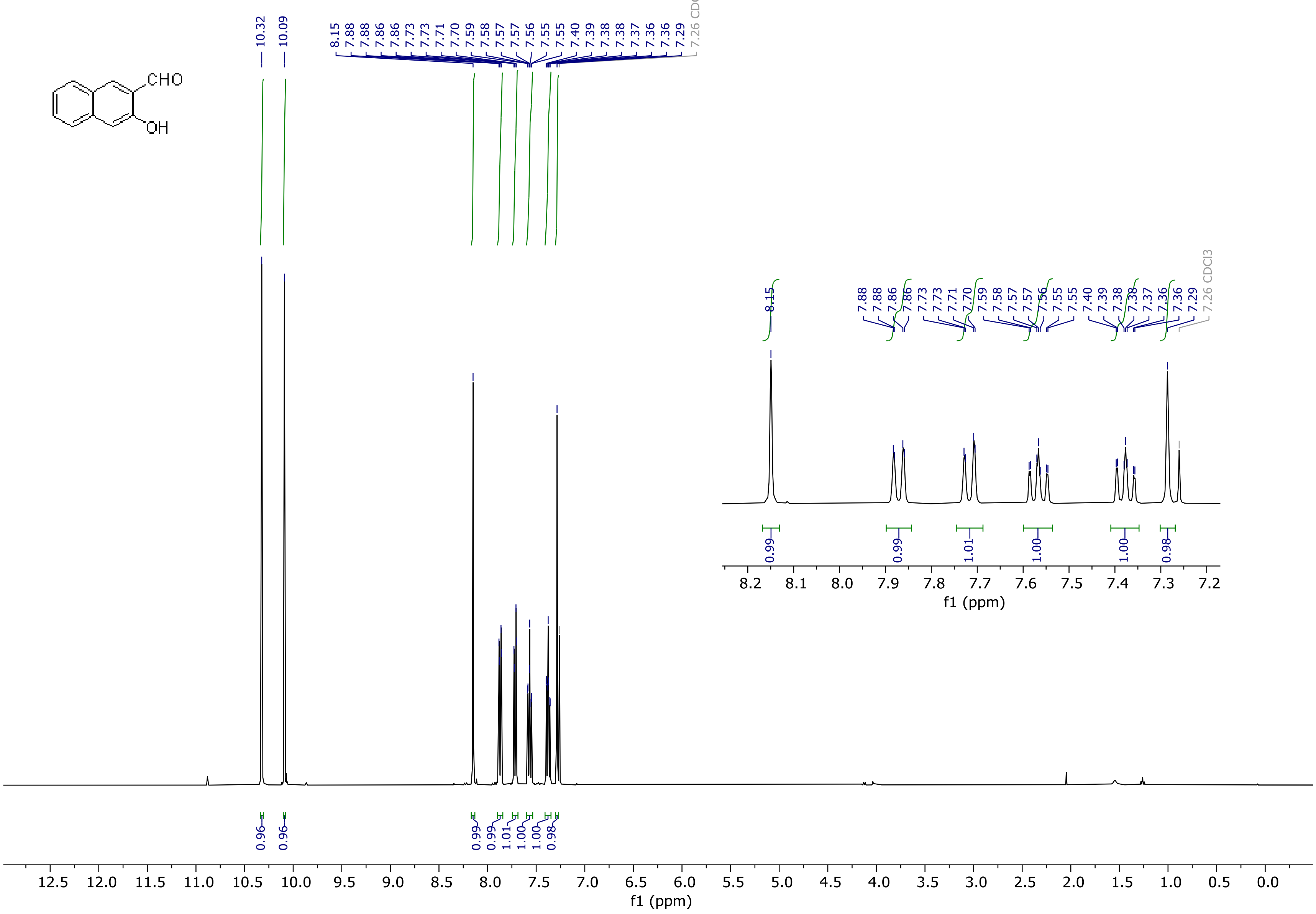

13C{1H} NMR: (101 MHz, CDCl3): 3-hydroxy-2-naphthaldehyde 2s.

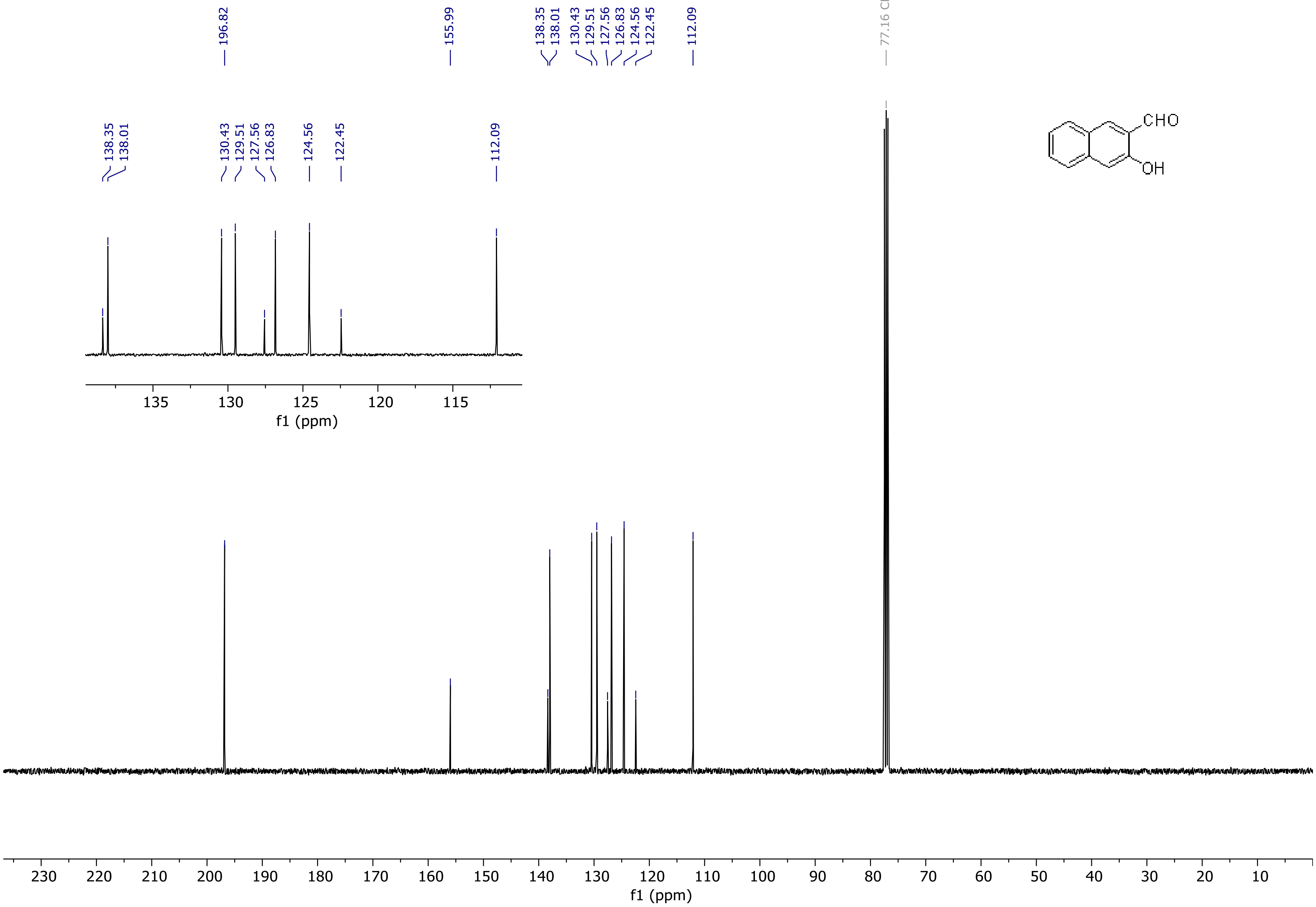

1H NMR (400 MHz, CDCl3): (S)-4-isobutyl-4,5-dihydropyrrolo[1,2-a]quinoxaline 3b.

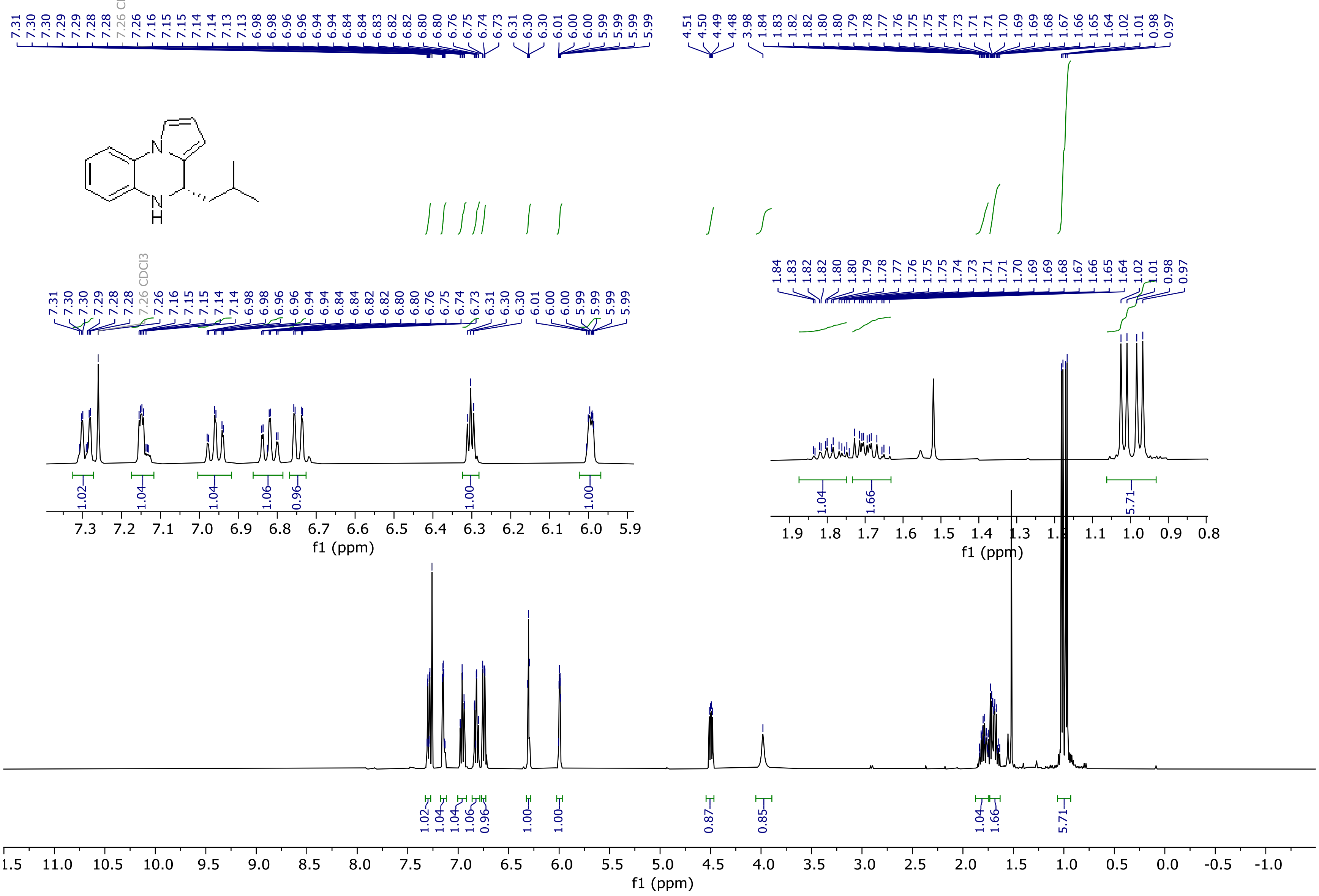

13C{1H} NMR: (101 MHz, CDCl3): (S)-4-isobutyl-4,5-dihydropyrrolo[1,2-a]quinoxaline 3b.

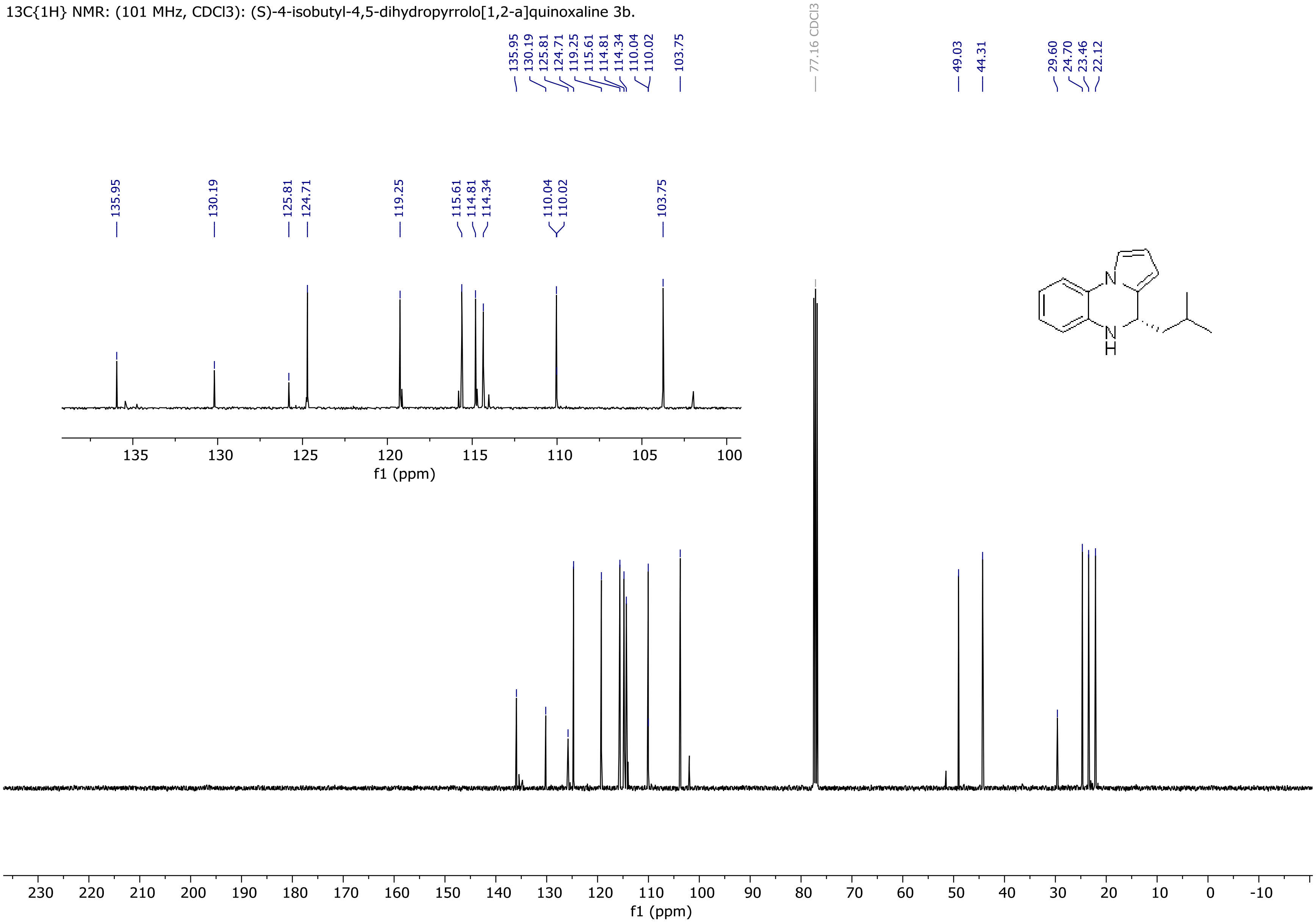

1H NMR (400 MHz, CDCl3): (S)-2-(4,5-dihydropyrrolo[1,2-a]quinoxalin-4-yl)phenol 3c.

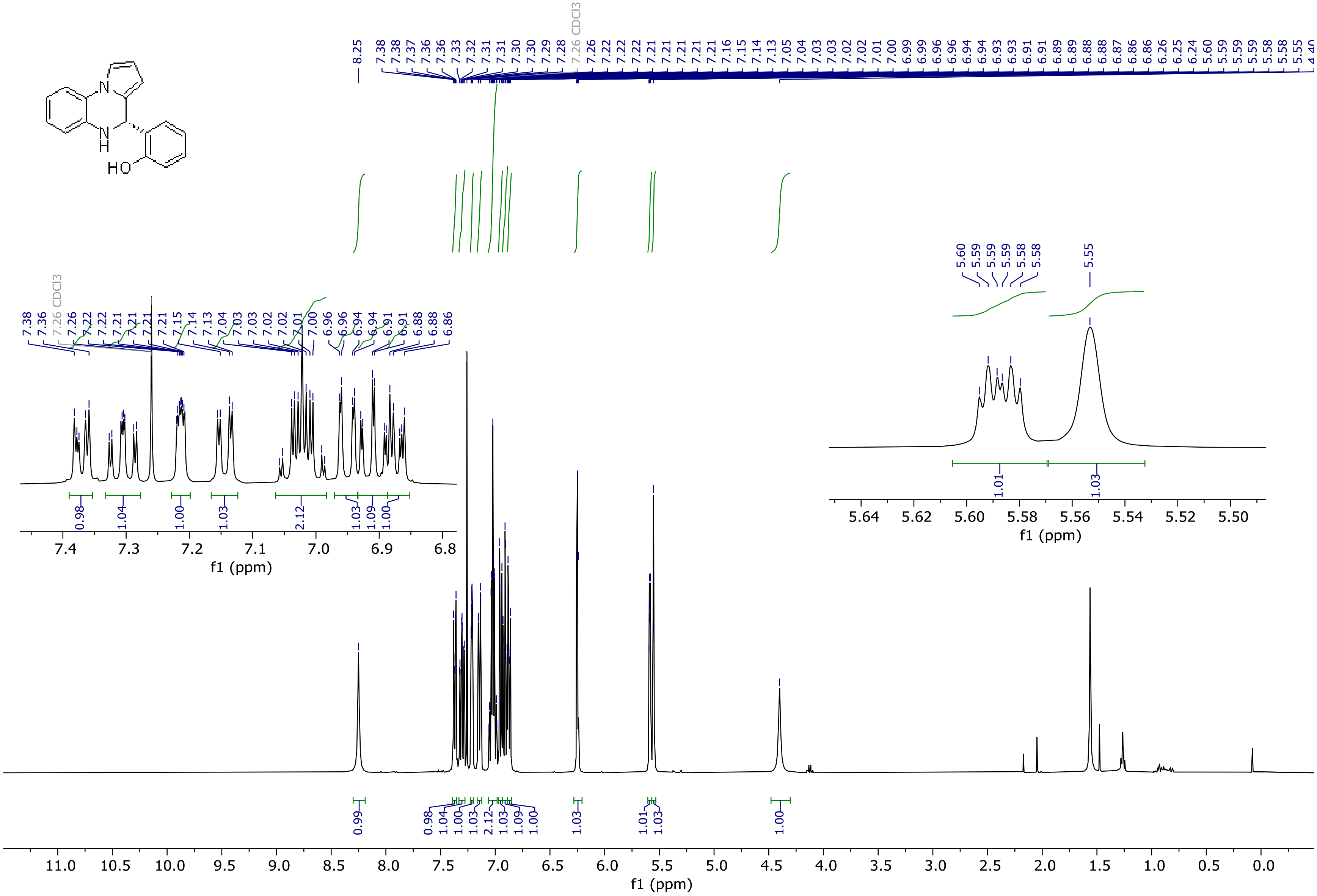

13C{1H} NMR: (101 MHz, CDCl3): (S)-2-(4,5-dihydropyrrolo[1,2-a]quinoxalin-4-yl)phenol 3c.

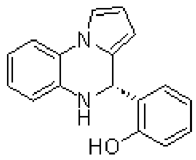

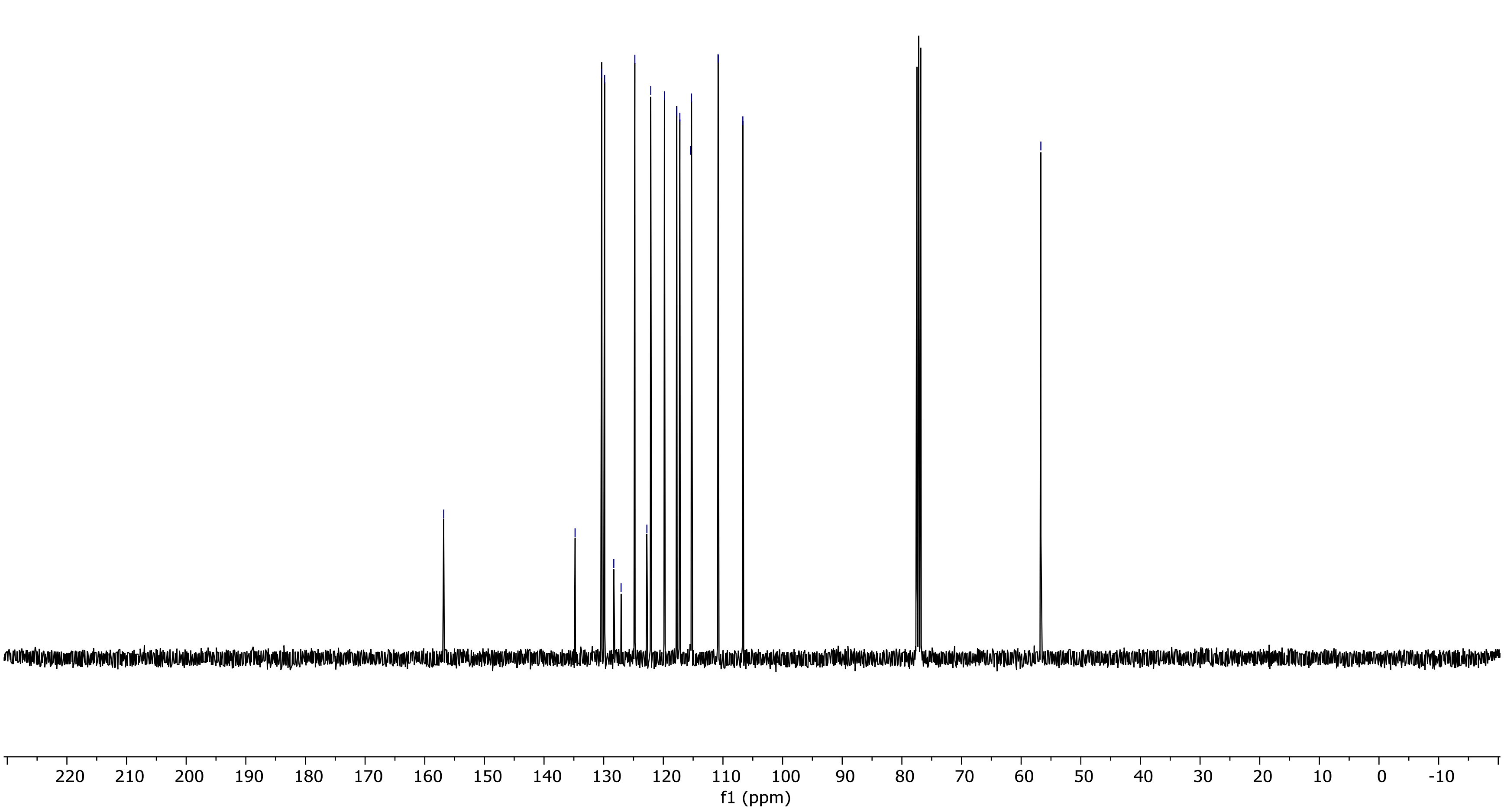

1H NMR (400 MHz, CDCl3): (S)-2-(4,5-dihydropyrrolo[1,2-a]quinoxalin-4-yl)-4-methylphenol 3d.

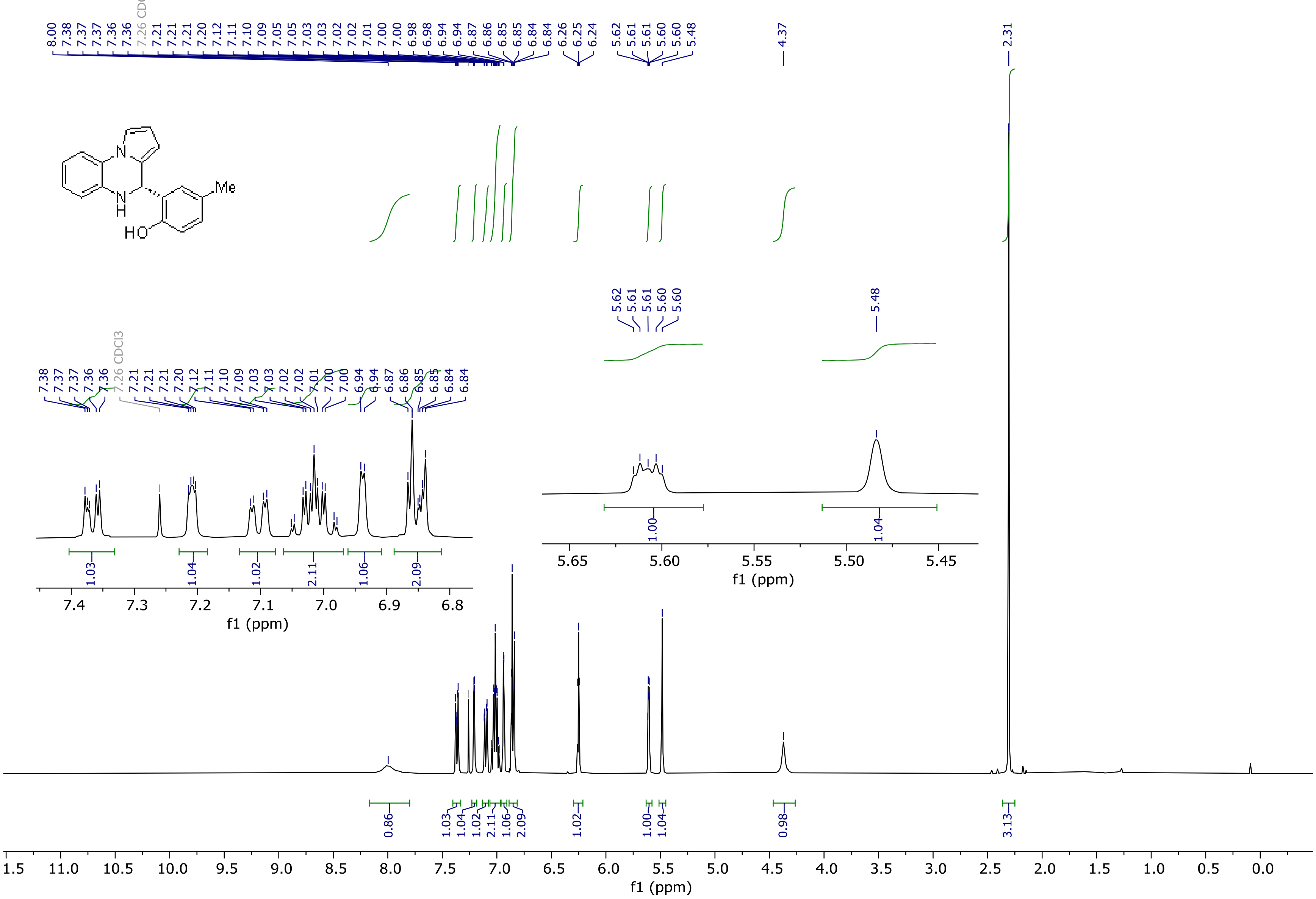

13C{1H} NMR: (101 MHz, CDCl3): (S)-2-(4,5-dihydropyrrolo[1,2-a]quinoxalin-4-yl)-4-methylphenol 3d.

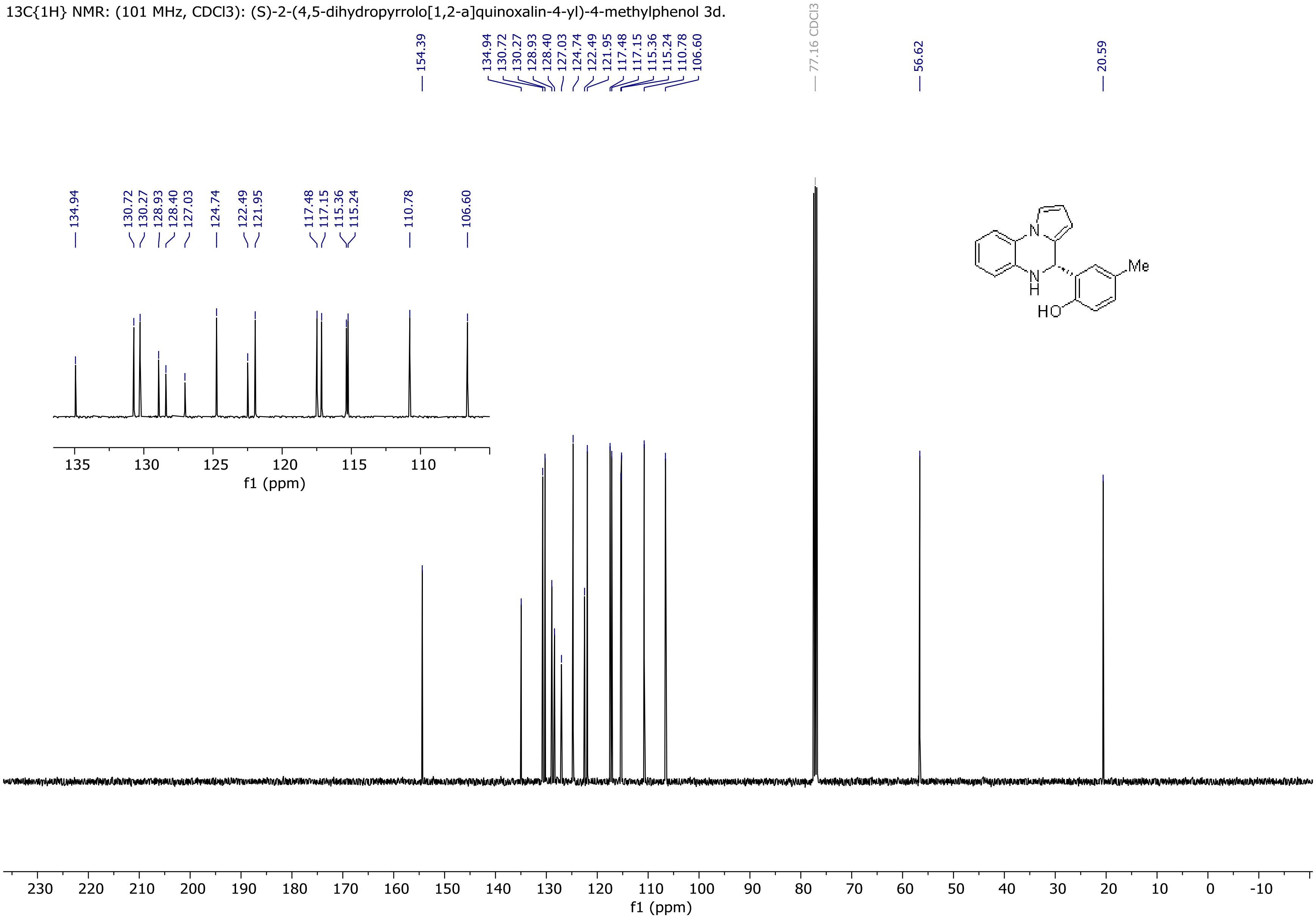

1H NMR (400 MHz, (CD3)2SO)): (S)-2-(4,5-dihydropyrrolo[1,2-a]quinoxalin-4-yl)-5-methylphenol 3e.

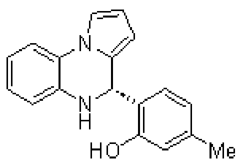

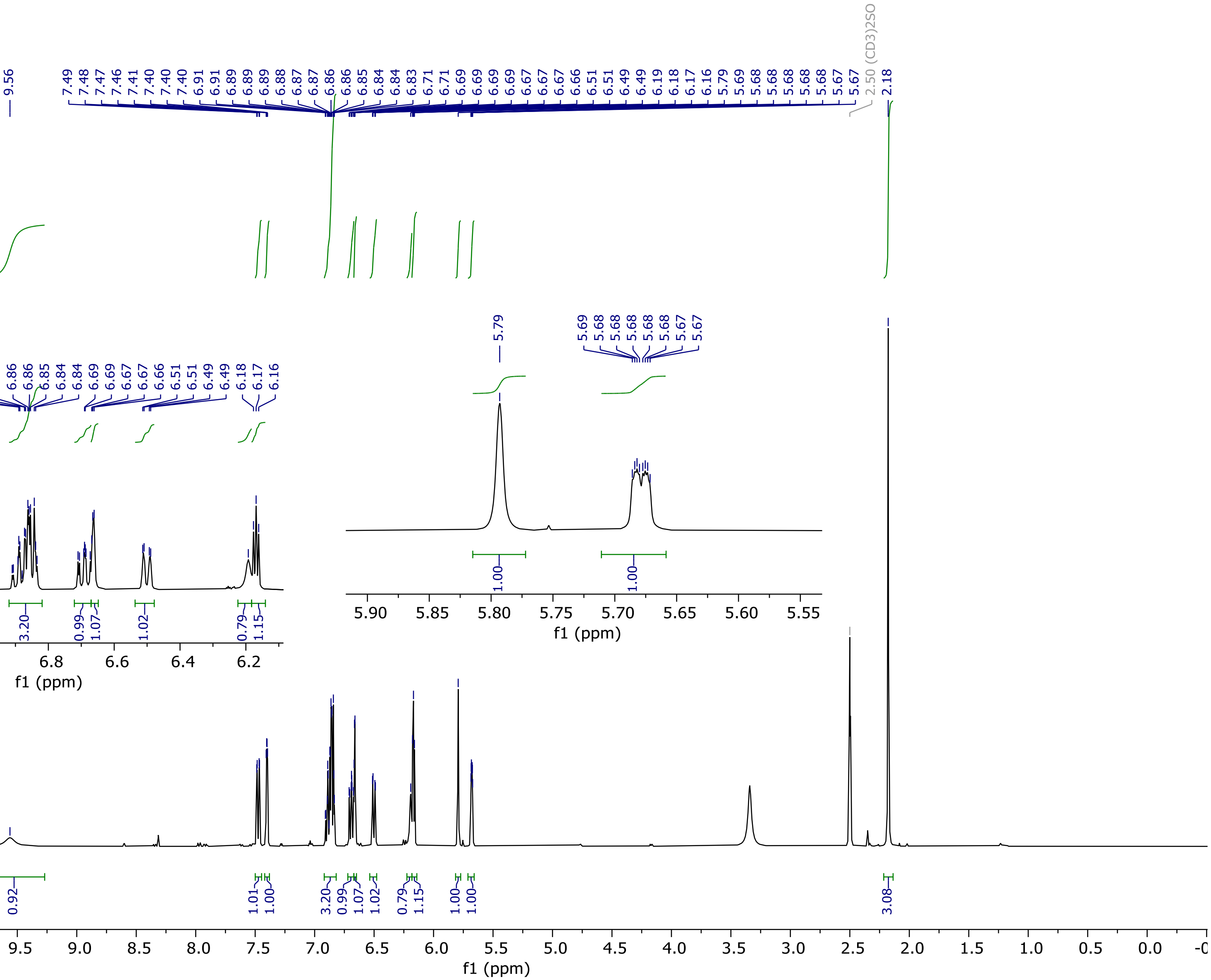

13C{1H} NMR: (101 MHz, (CD3)2SO): (S)-2-(4,5-dihydropyrrolo[1,2-a]quinoxalin-4-yl)-5-methylphenol 3e.

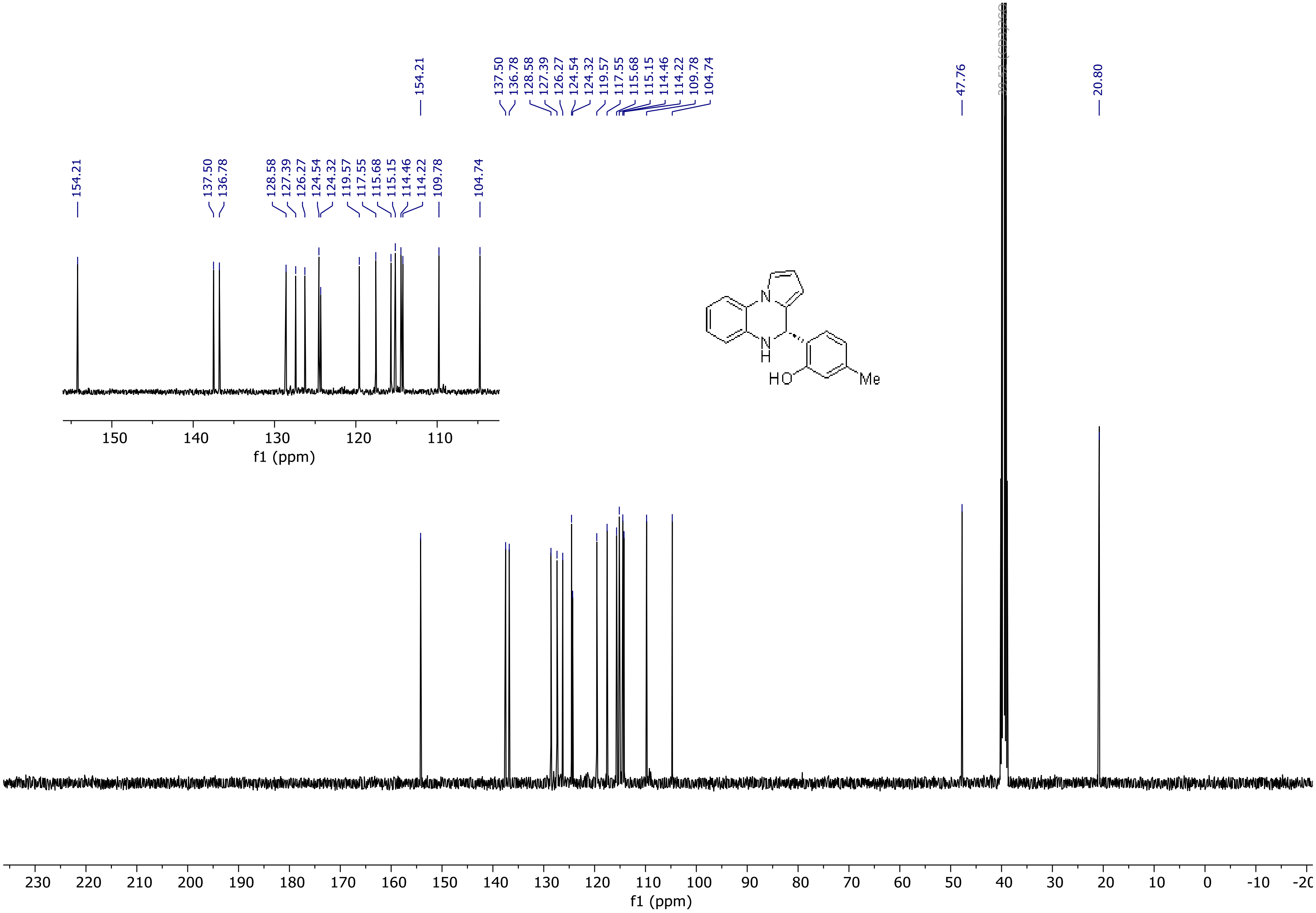

1H NMR (400 MHz, CDCl3): (S)-2-(4,5-dihydropyrrolo[1,2-a]quinoxalin-4-yl)-4-methoxyphenol 3g.

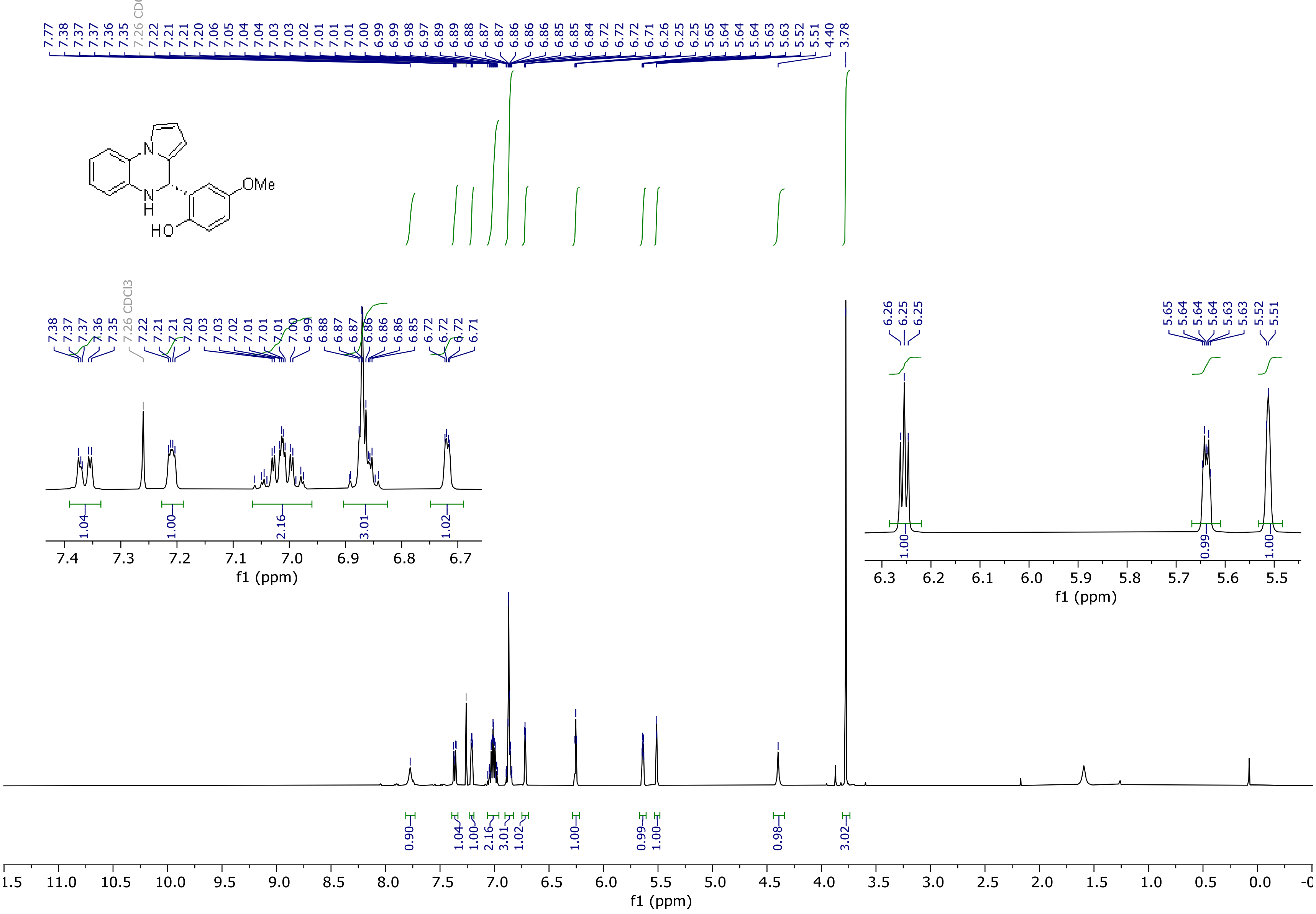

13C{1H} NMR: (101 MHz, CDCl3): (S)-2-(4,5-dihydropyrrolo[1,2-a]quinoxalin-4-yl)-4-methoxyphenol 3g.

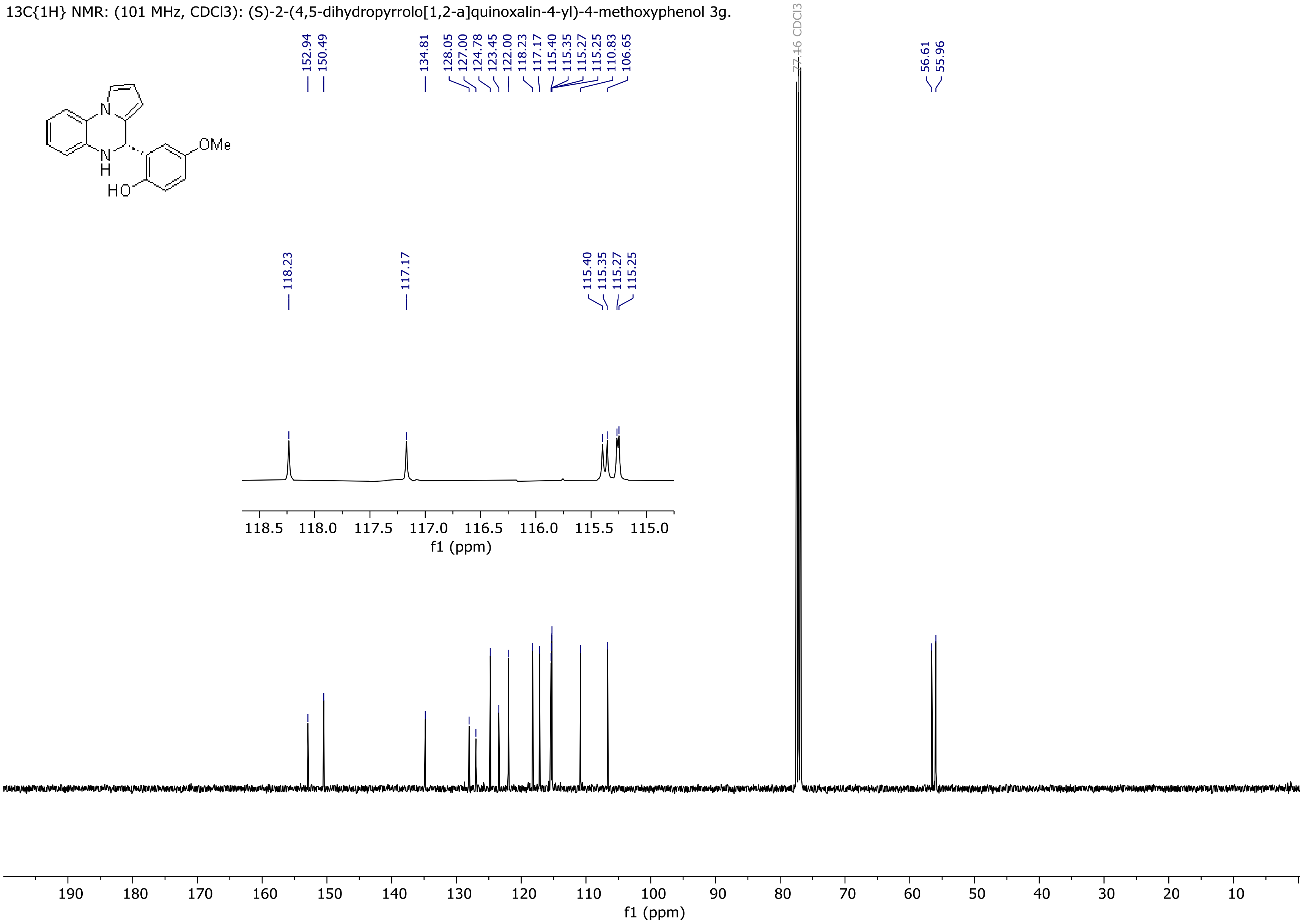

1H NMR (400 MHz, (CD3)2SO)): (S)-2-(4,5-dihydropyrrolo[1,2-a]quinoxalin-4-yl)-5-methoxyphenol 3h.

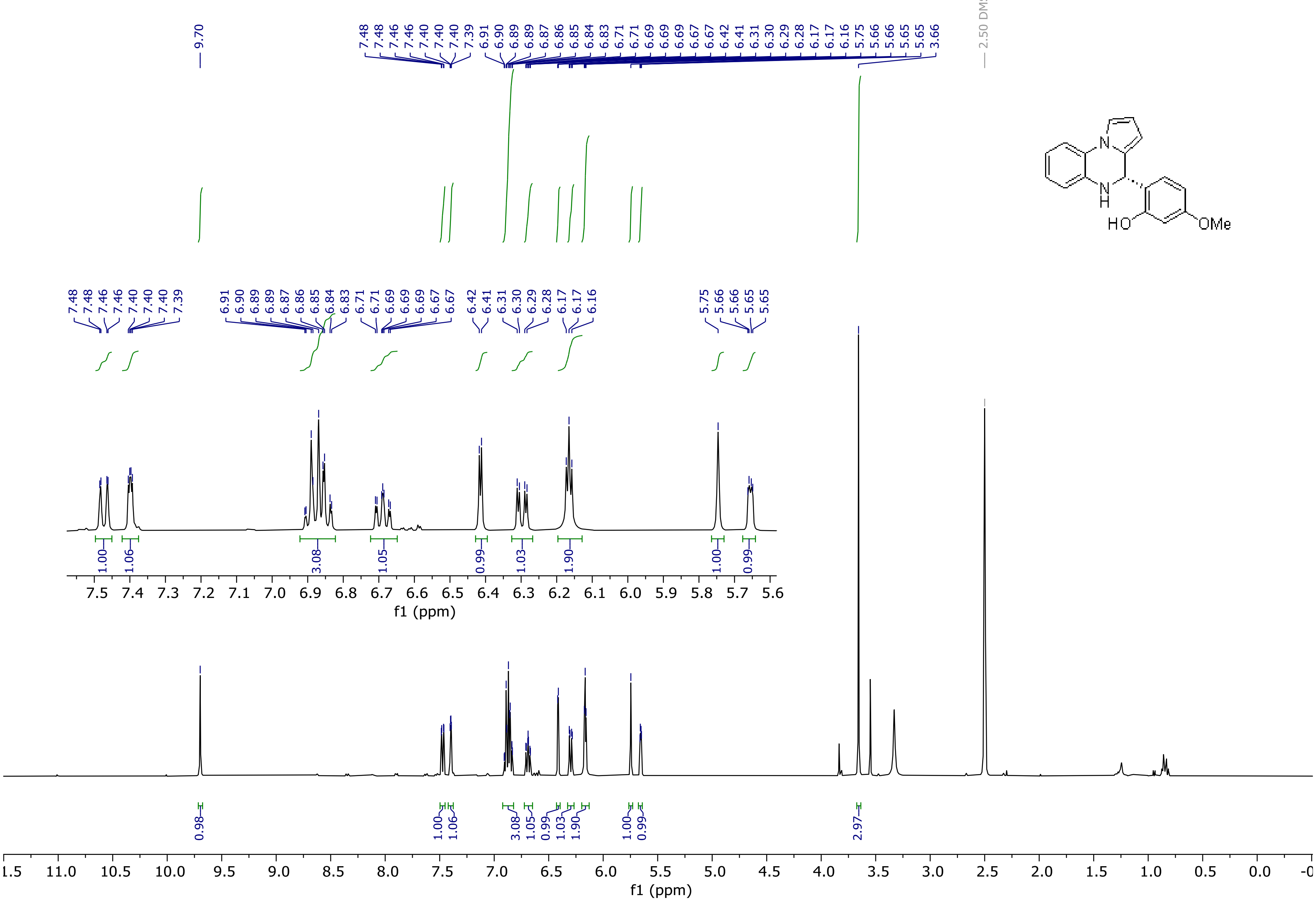

13C{1H} NMR: (101 MHz, (CD3)2SO)): (S)-2-(4,5-dihydropyrrolo[1,2-a]quinoxalin-4-yl)-5-methoxyphenol 3h.

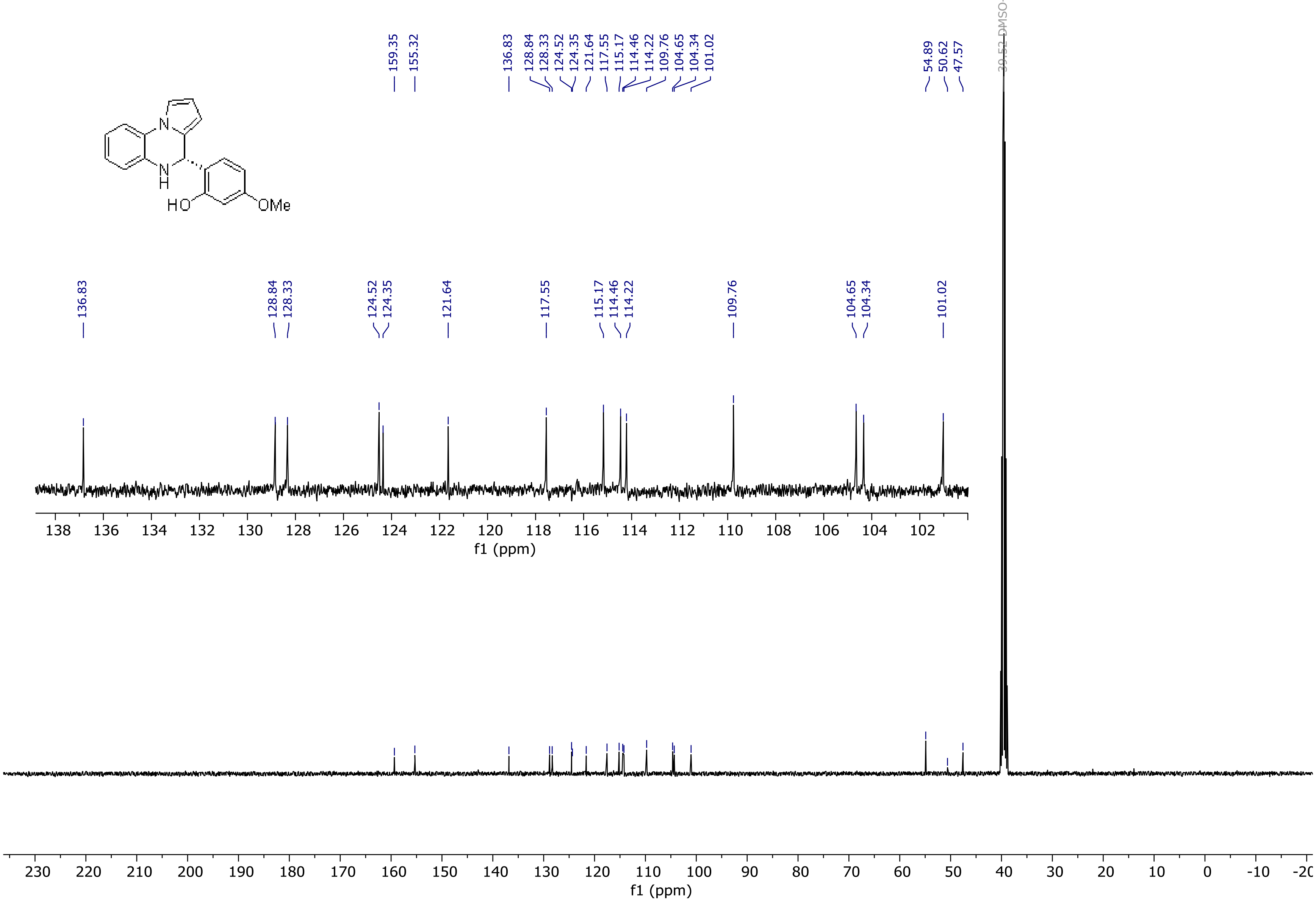

1H NMR (400 MHz, CDCl3): (S)-3-(4,5-dihydropyrrolo[1,2-a]quinoxalin-4-yl)-3',5'-dimethoxy-[1,1'-biphenyl]-4-ol 3i.

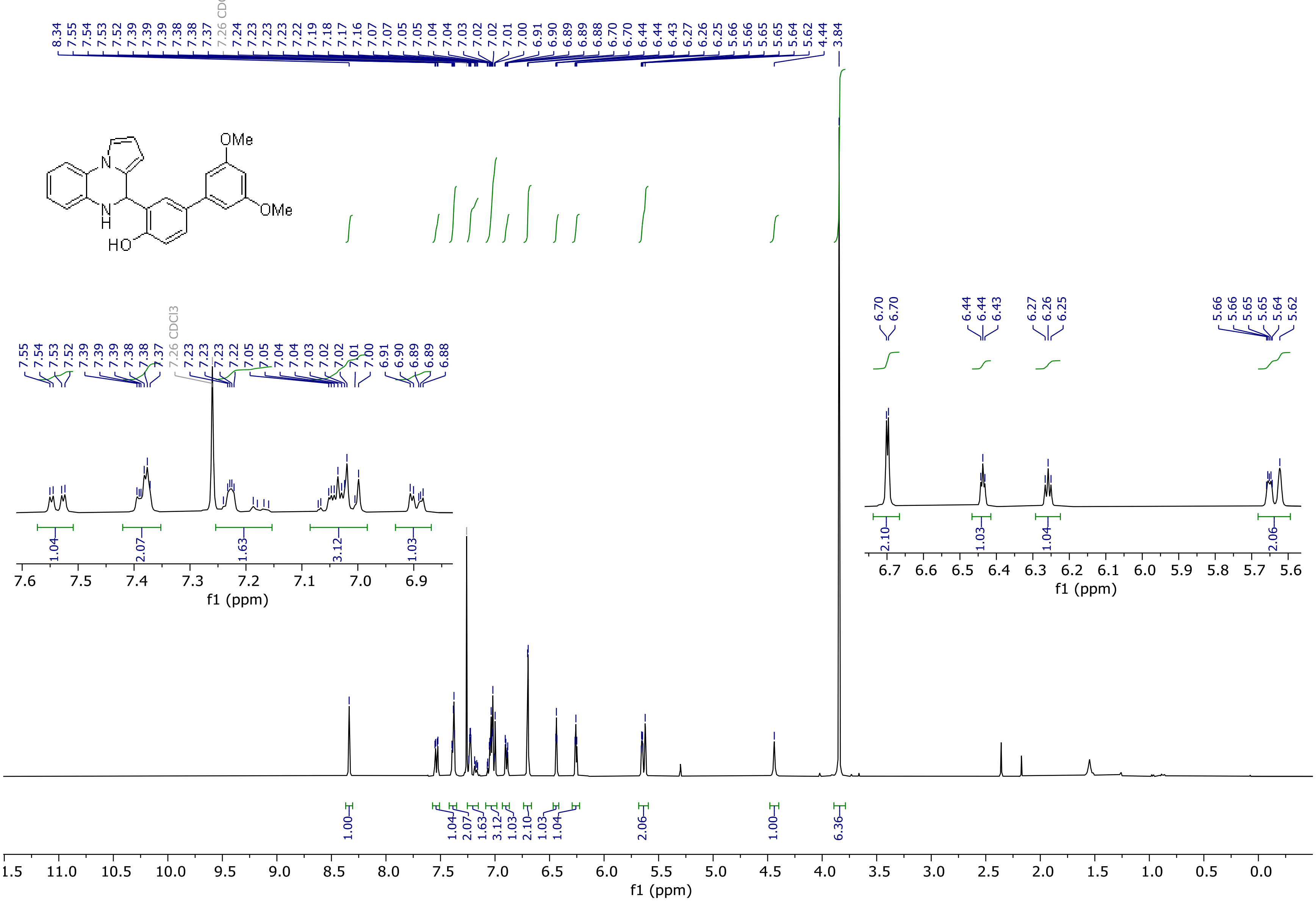

13C{1H} NMR: (101 MHz, CDCl3): (S)-3-(4,5-dihydropyrrolo[1,2-a]quinoxalin-4-yl)-3',5'-dimethoxy-[1,1'-biphenyl]-4-ol 3i.

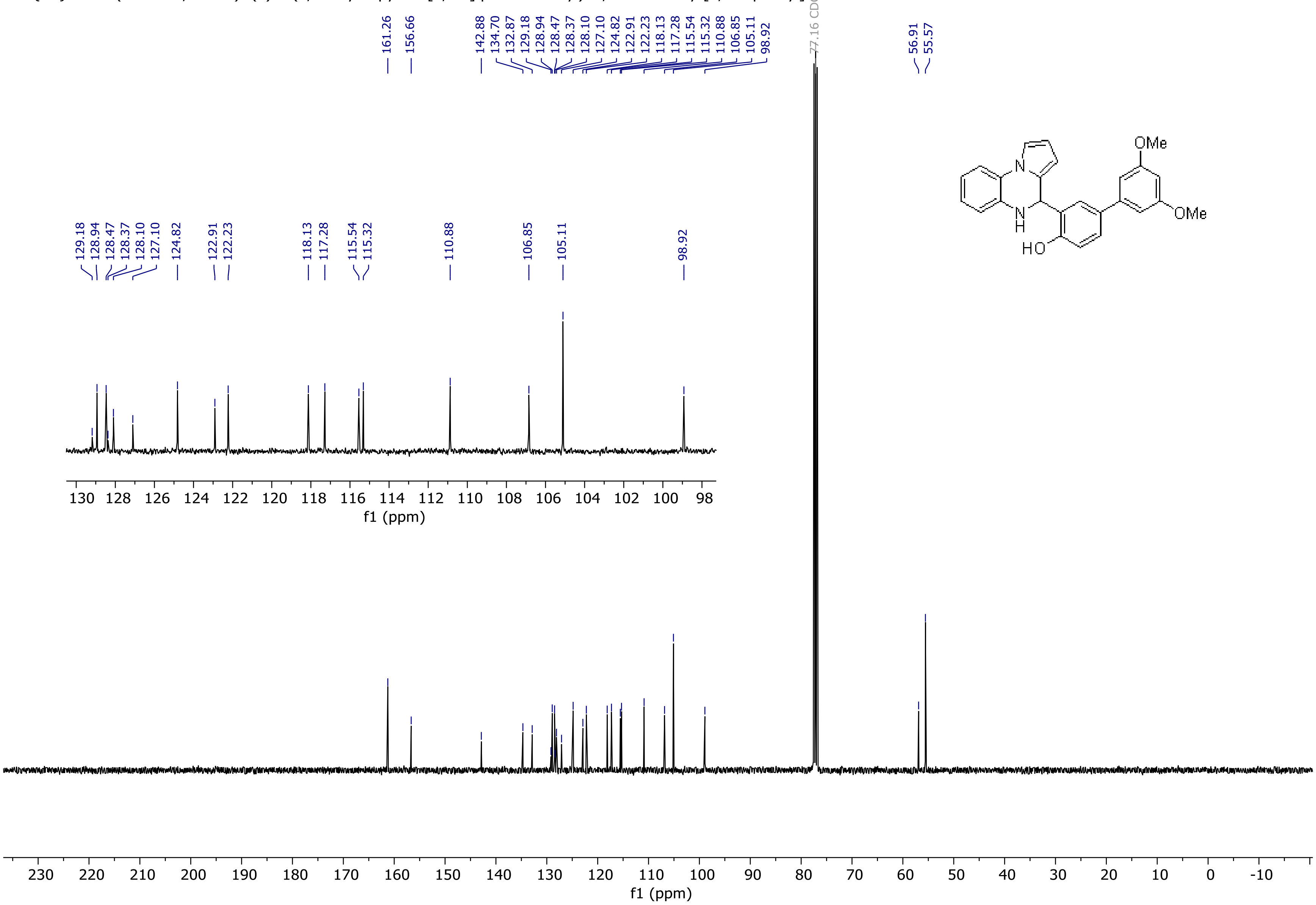

1H NMR (400 MHz, CDCl3): (S)-2-(4,5-dihydropyrrolo[1,2-a]quinoxalin-4-yl)-4-nitrophenol 3j.

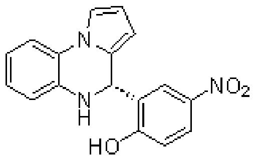

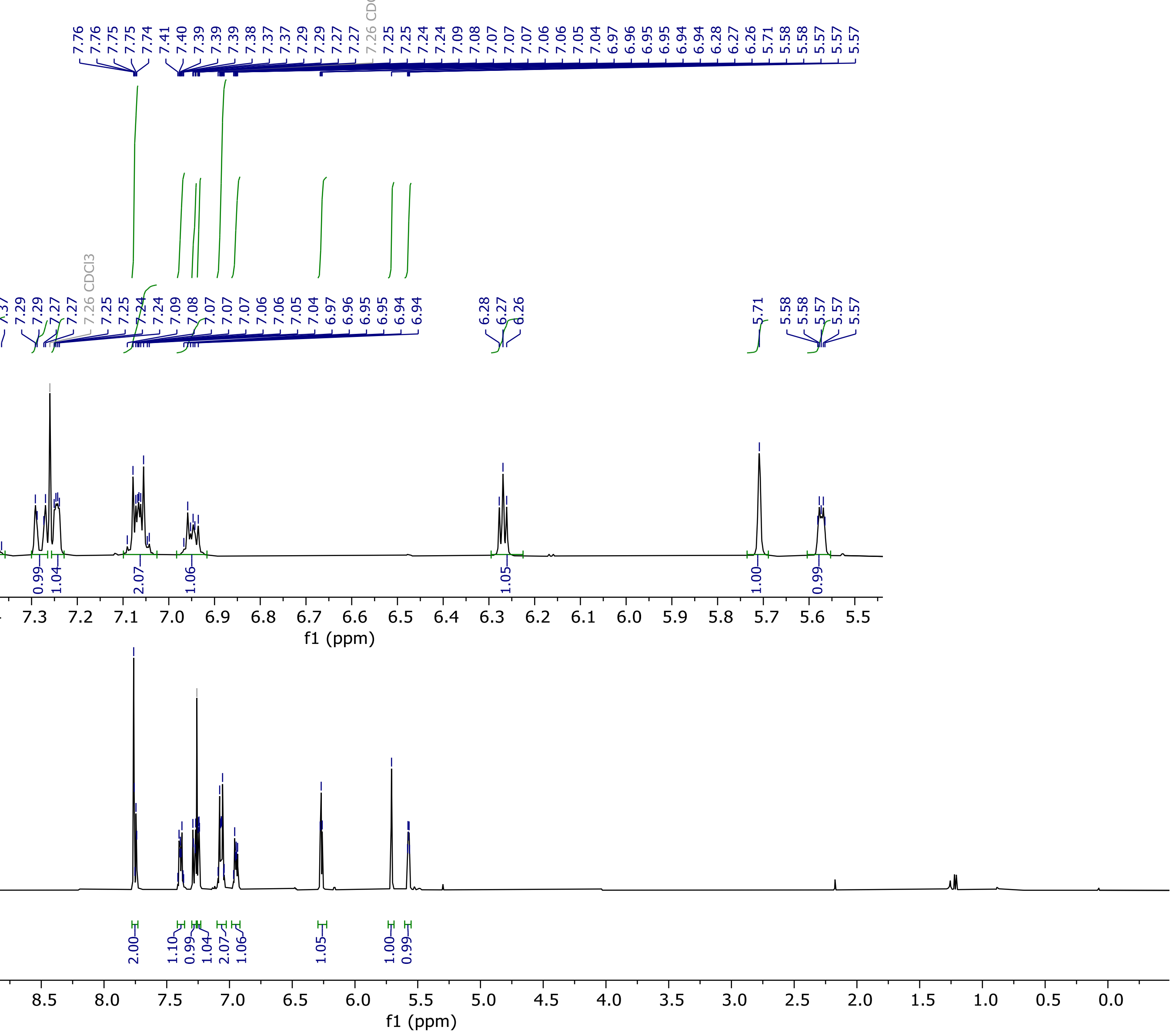

13C{1H} NMR: (101 MHz, CDCl3): (S)-2-(4,5-dihydropyrrolo[1,2-a]quinoxalin-4-yl)-4-nitrophenol 3j.

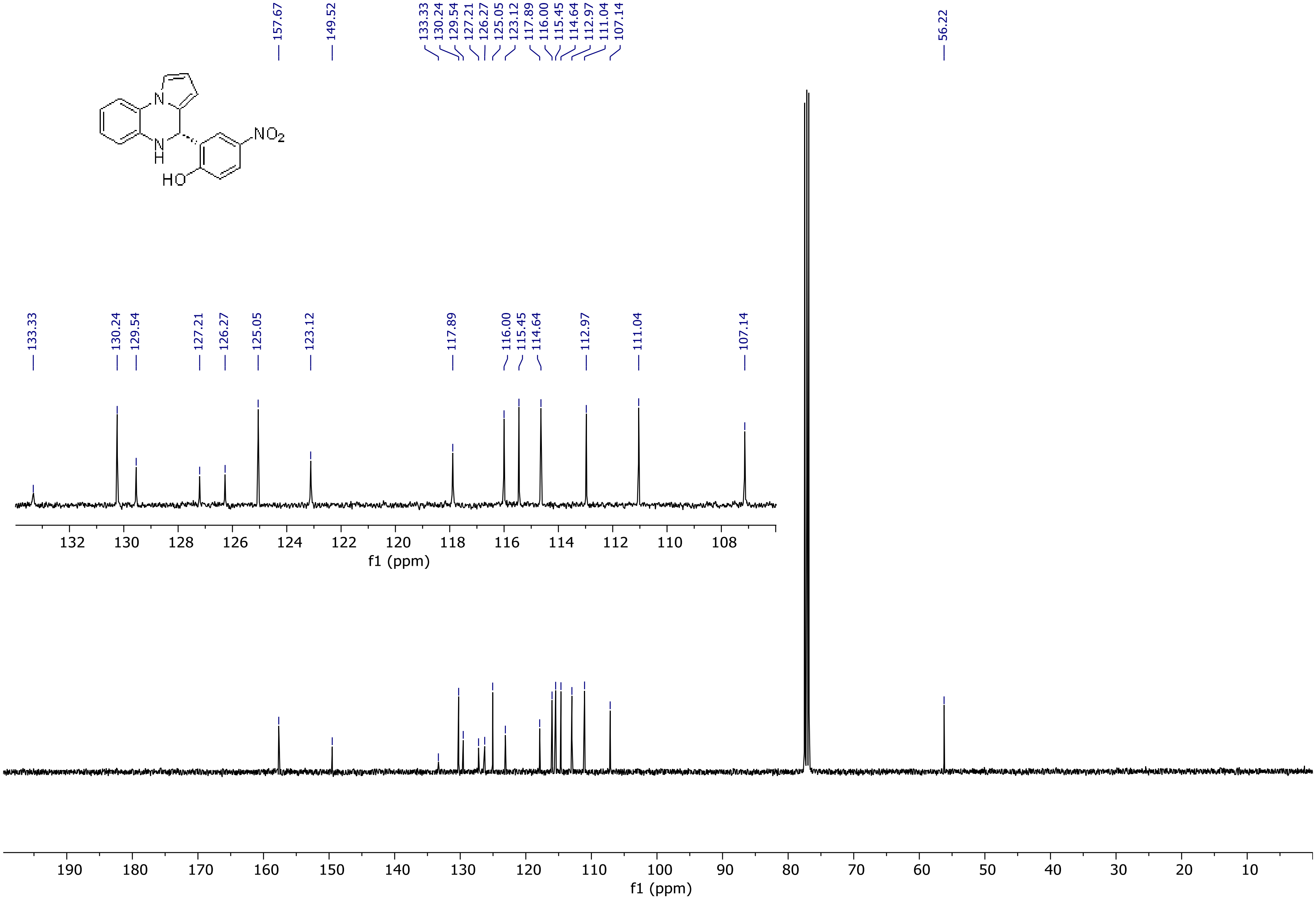

1H NMR (400 MHz, (CD3)2SO)): (S)-3-(4,5-dihydropyrrolo[1,2-a]quinoxalin-4-yl)-4-hydroxybenzonitrile 3k.

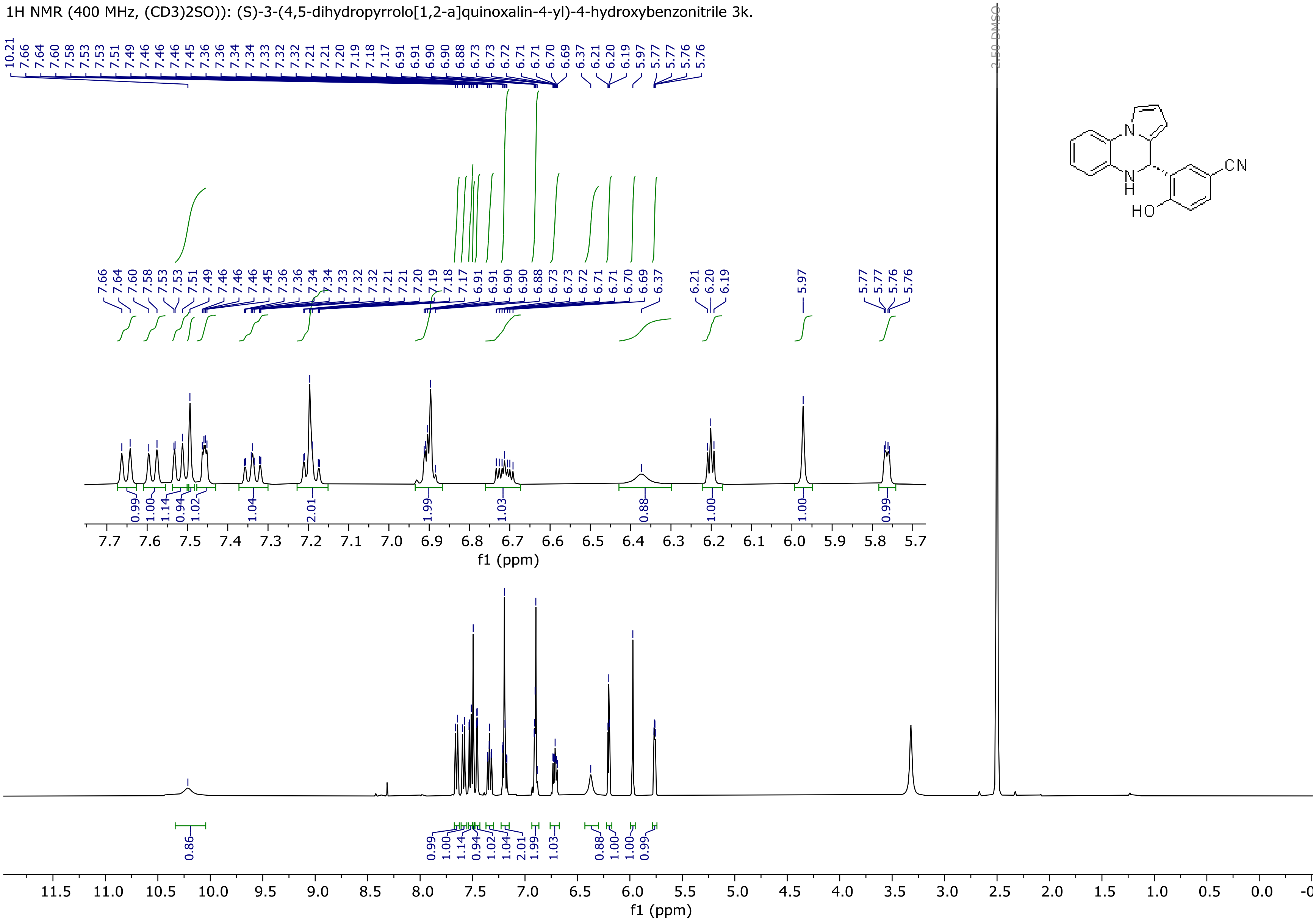

13C{1H} NMR: (101 MHz, (CD3)2SO)): (S)-3-(4,5-dihydropyrrolo[1,2-a]quinoxalin-4-yl)-4-hydroxybenzonitrile 3k.

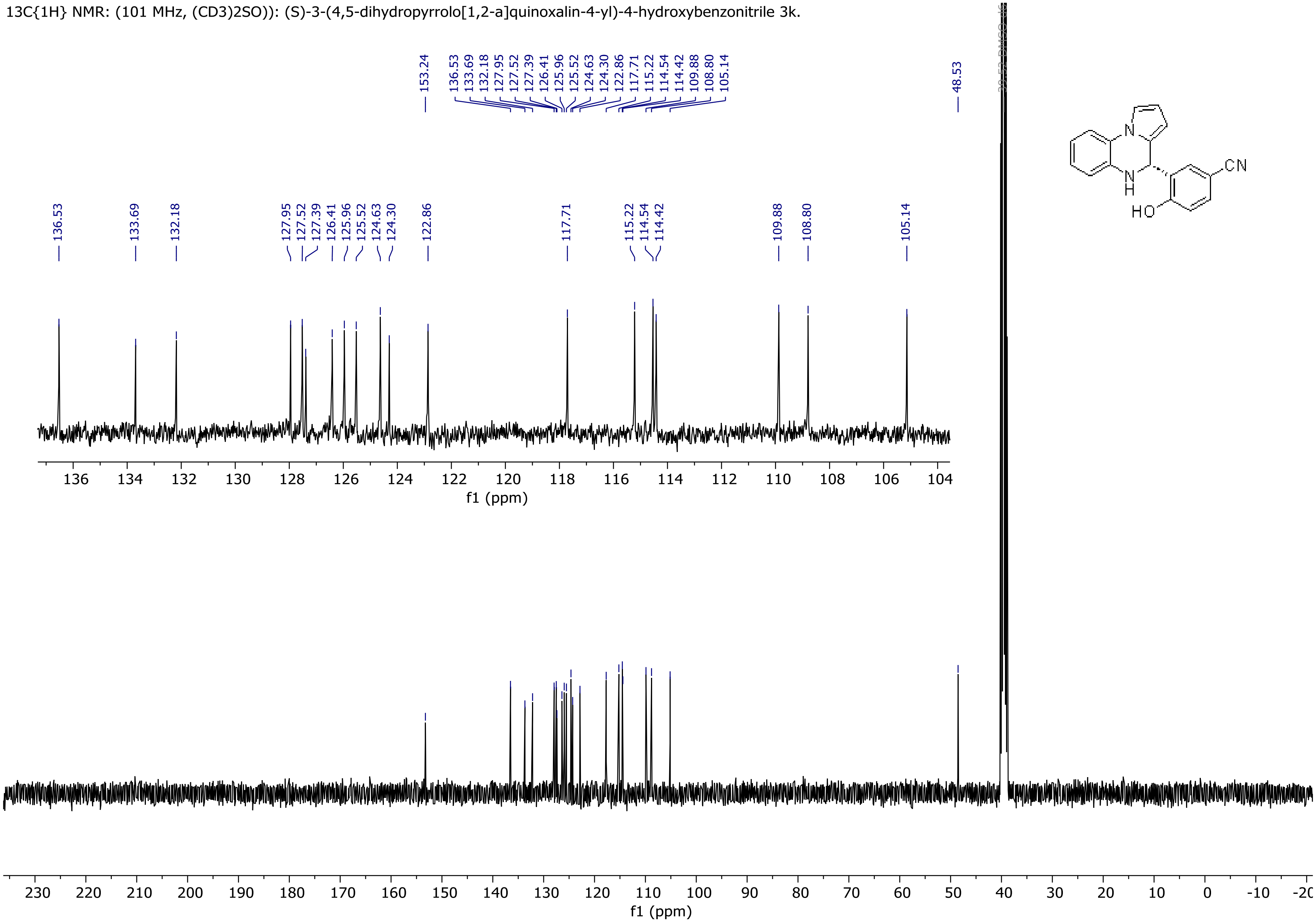

1H NMR (400 MHz, (CD3)2SO)): Methyl (S)-3-(4,5-dihydropyrrolo[1,2-a]quinoxalin-4-yl)-4-hydroxybenzoate 3l.

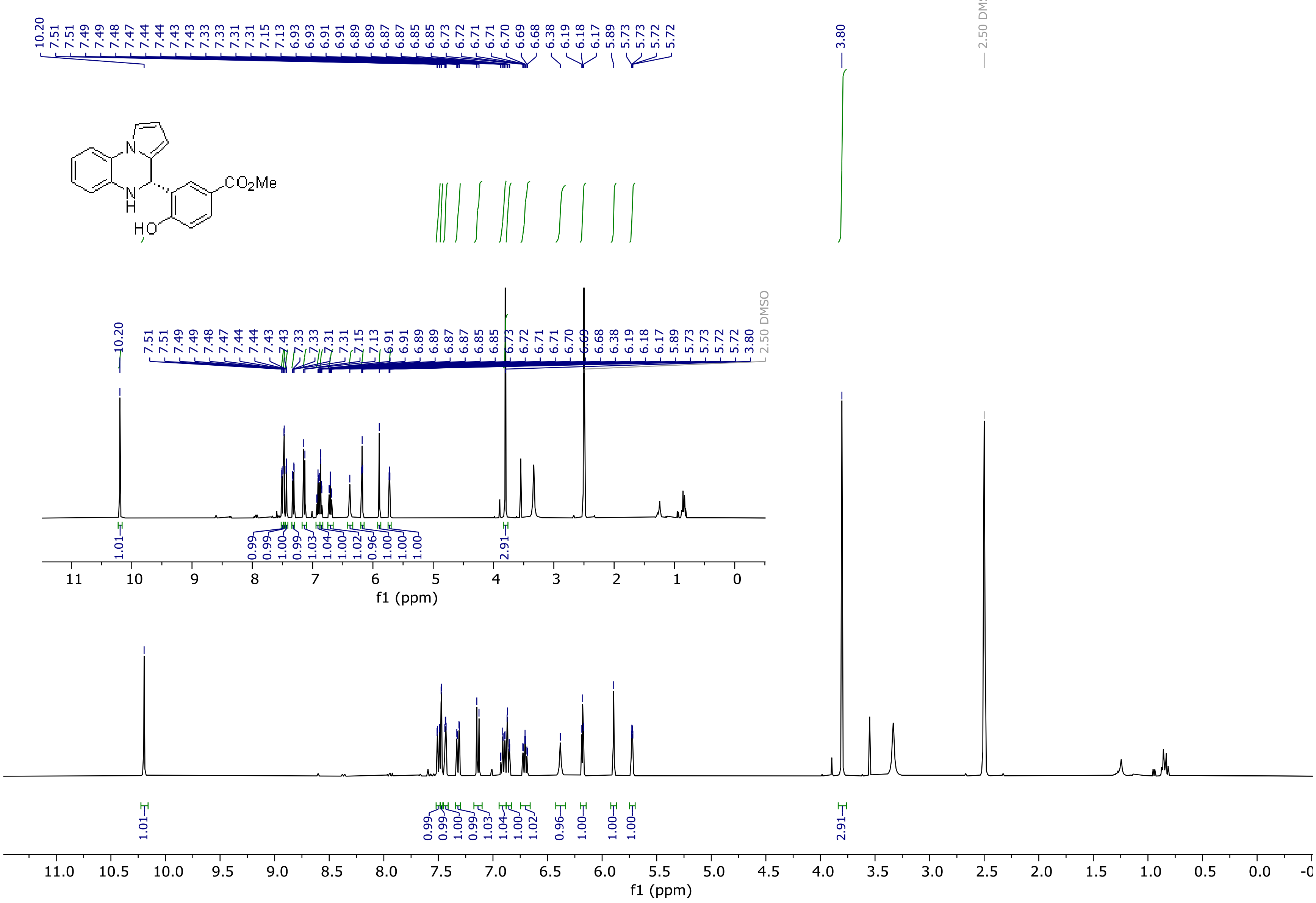

13C{1H} NMR: (101 MHz, (CD3)2SO)): Methyl (S)-3-(4,5-dihydropyrrolo[1,2-a]quinoxalin-4-yl)-4-hydroxybenzoate 3l.

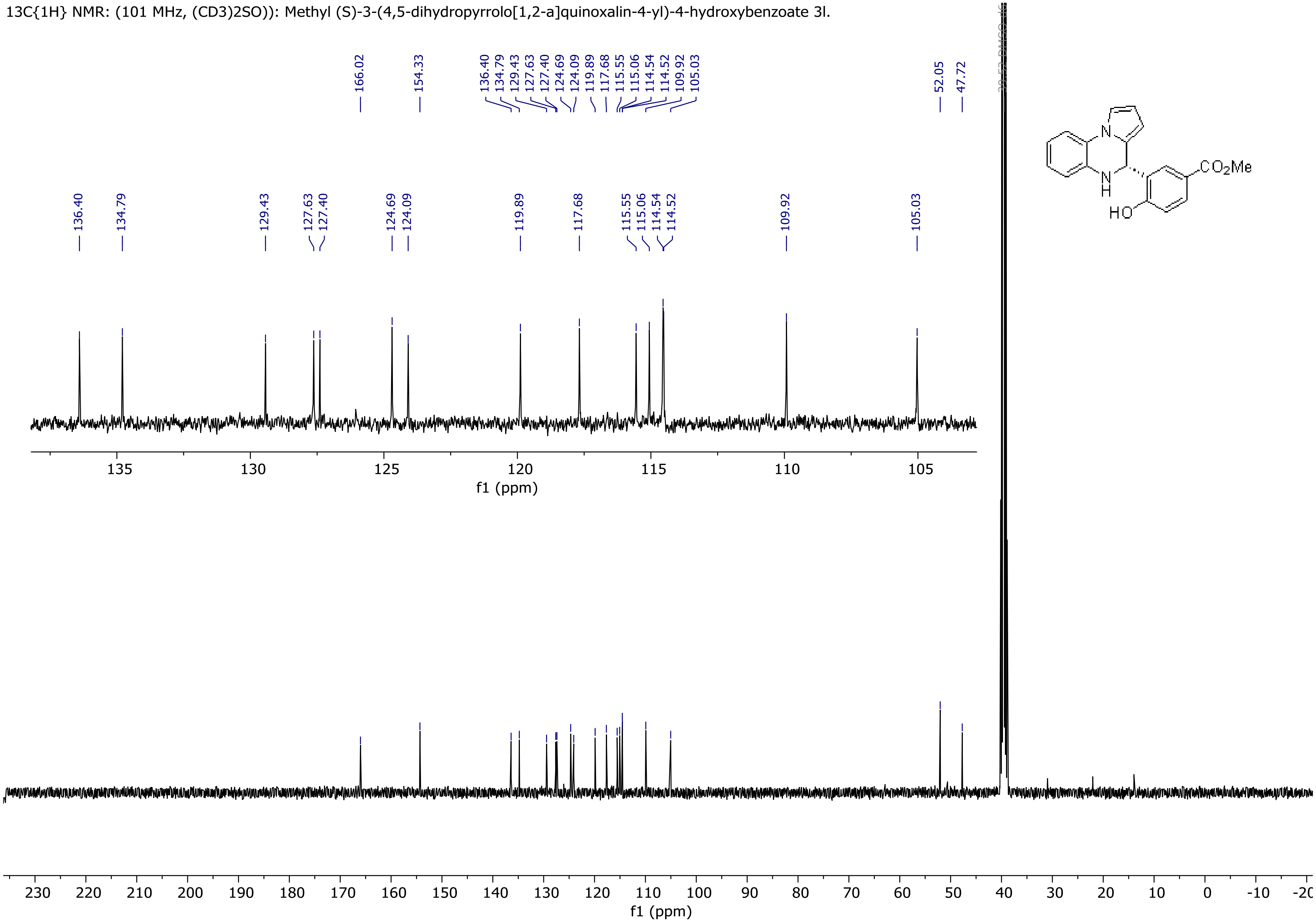

1H NMR (400 MHz, CDCl3): (S)-4-chloro-2-(4,5-dihydropyrrolo[1,2-a]quinoxalin-4-yl)phenol 3m.

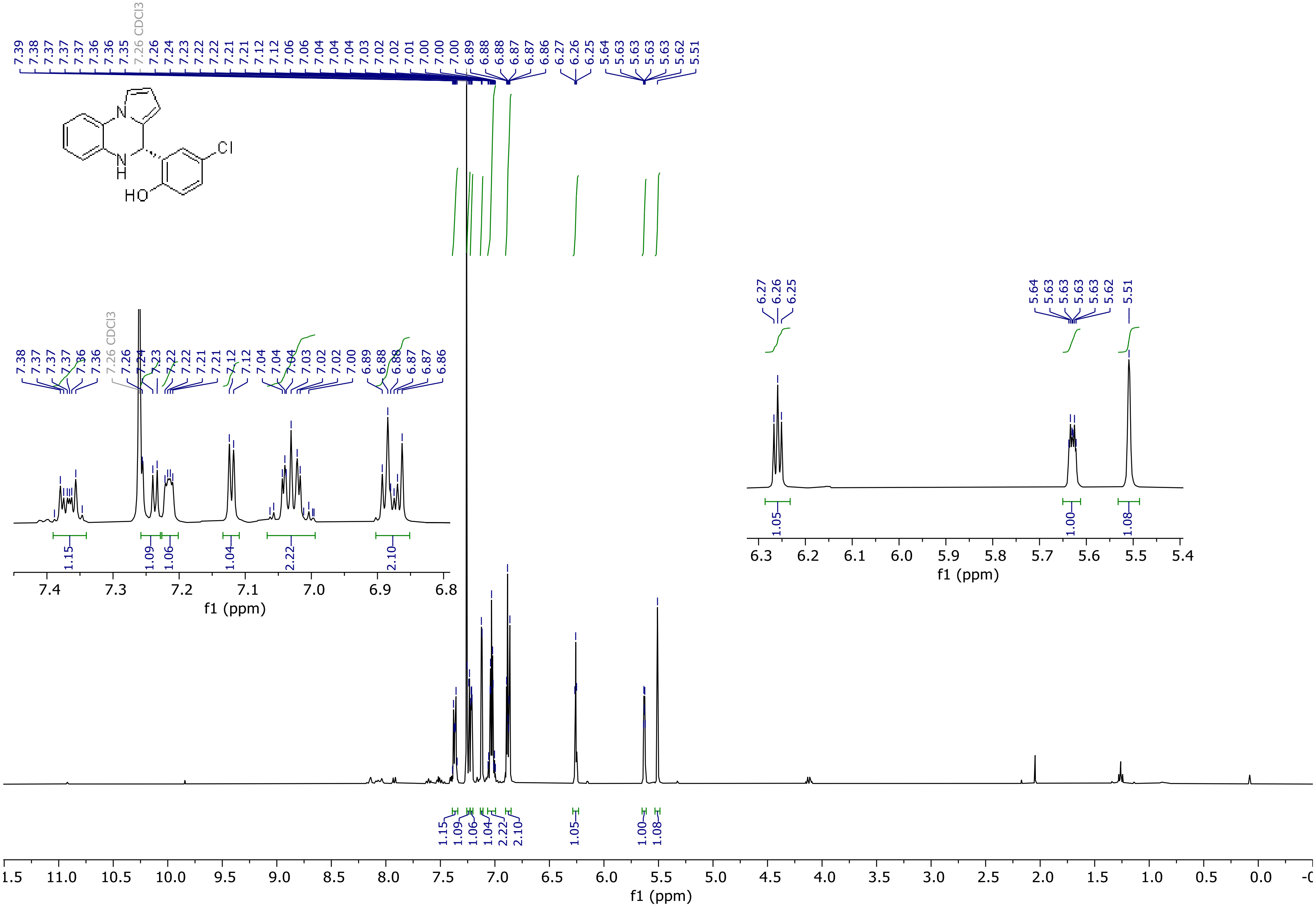

13C{1H} NMR: (101 MHz, CDCl3): (S)-4-chloro-2-(4,5-dihydropyrrolo[1,2-a]quinoxalin-4-yl)phenol 3m.

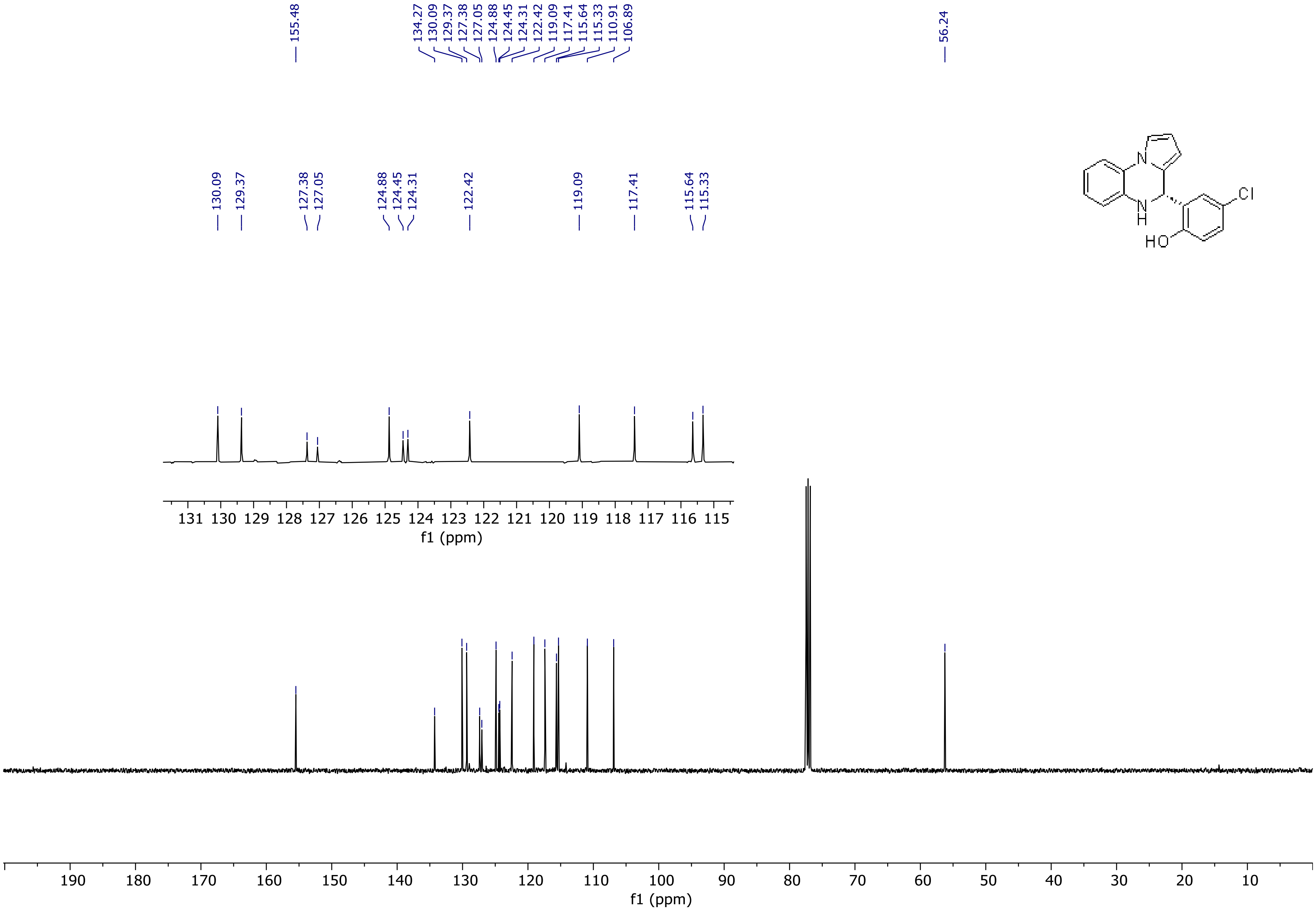

1H NMR (400 MHz, CDCl3): (S)-5-chloro-2-(4,5-dihydropyrrolo[1,2-a]quinoxalin-4-yl)phenol 3n.

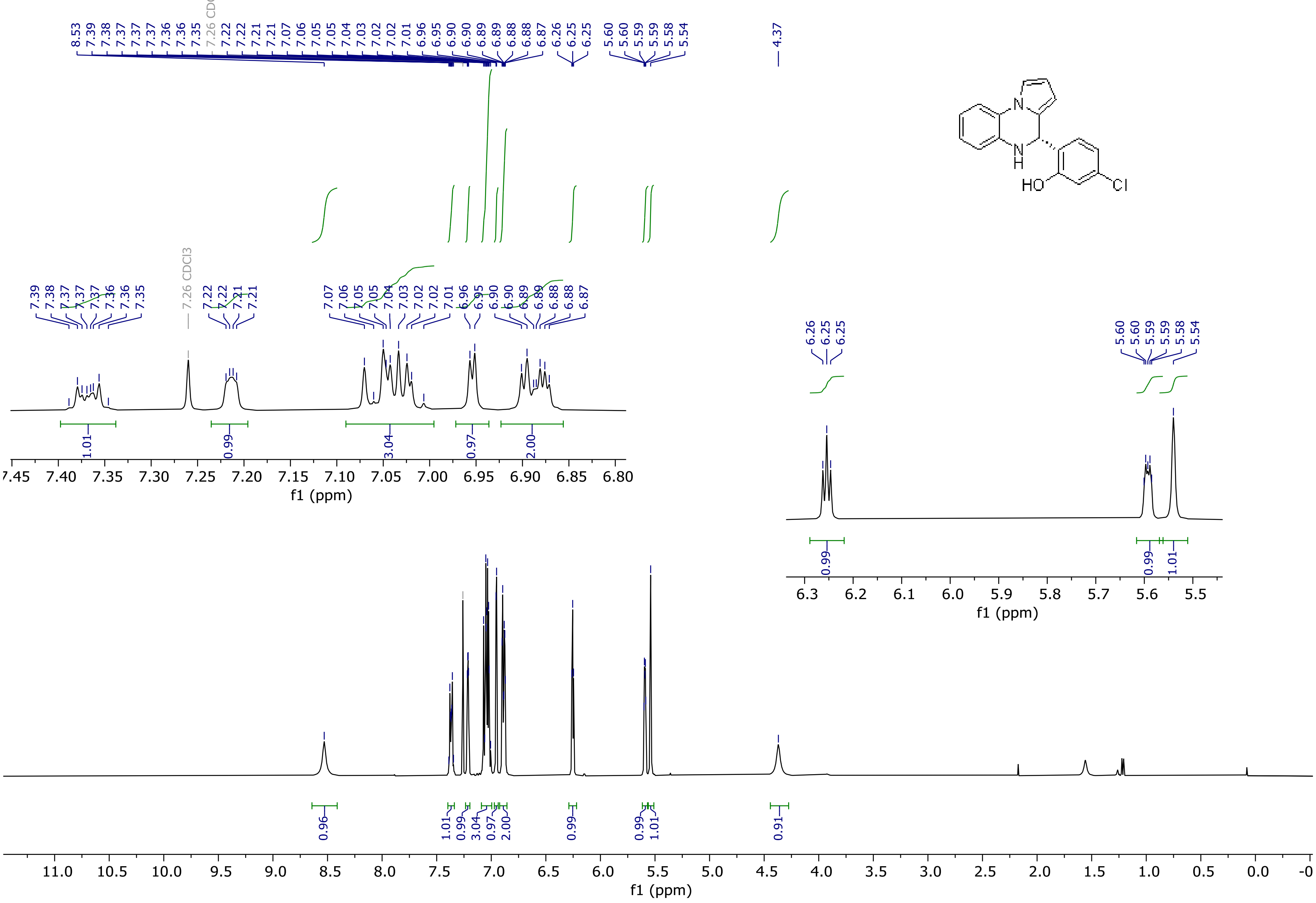

13C{1H} NMR: (101 MHz, CDCl3): (S)-5-chloro-2-(4,5-dihydropyrrolo[1,2-a]quinoxalin-4-yl)phenol 3n.

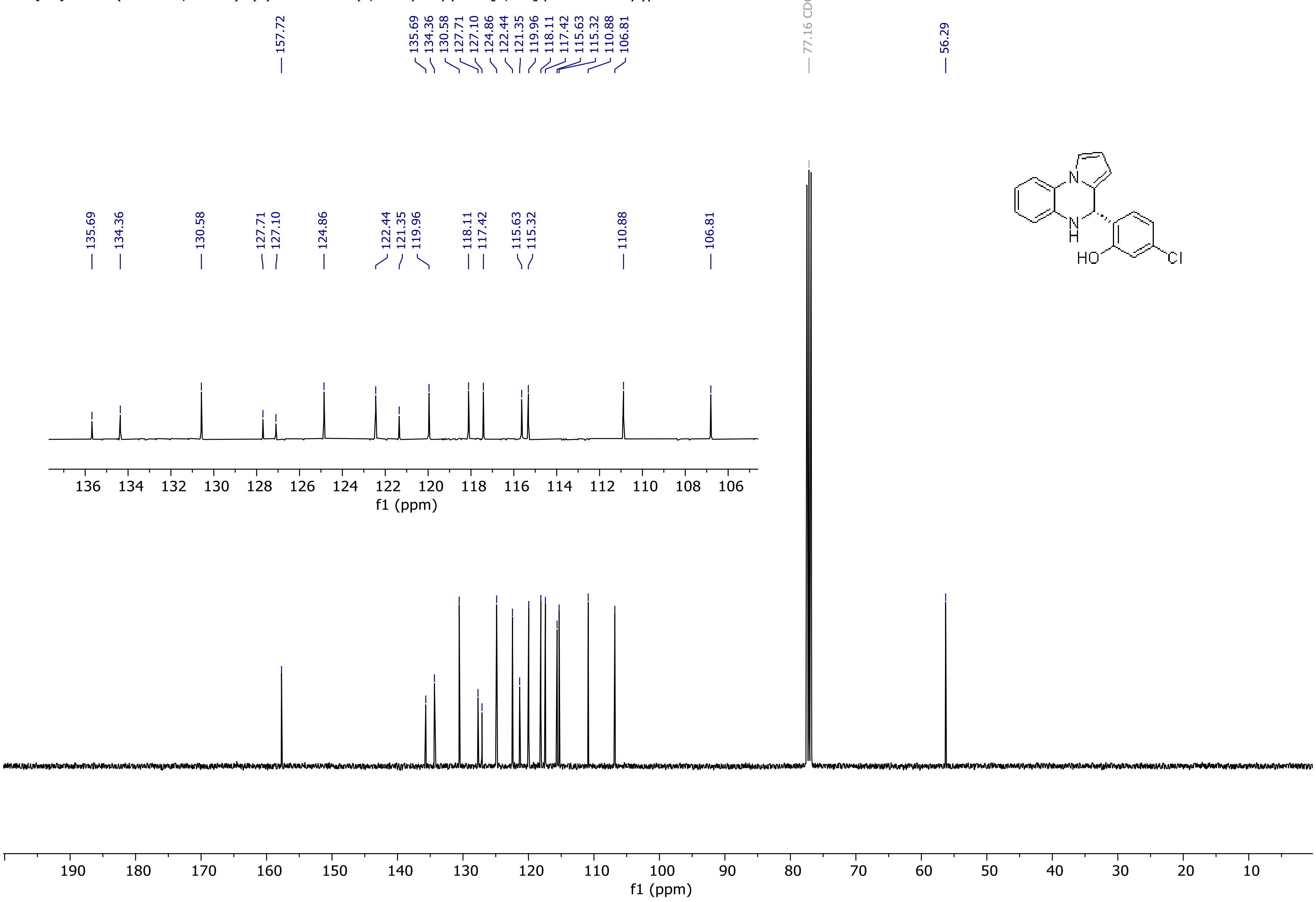

1H NMR (600 MHz, (CD3)2SO)): (S)-4-bromo-2-(4,5-dihydropyrrolo[1,2-a]quinoxalin-4-yl)phenol 3o.

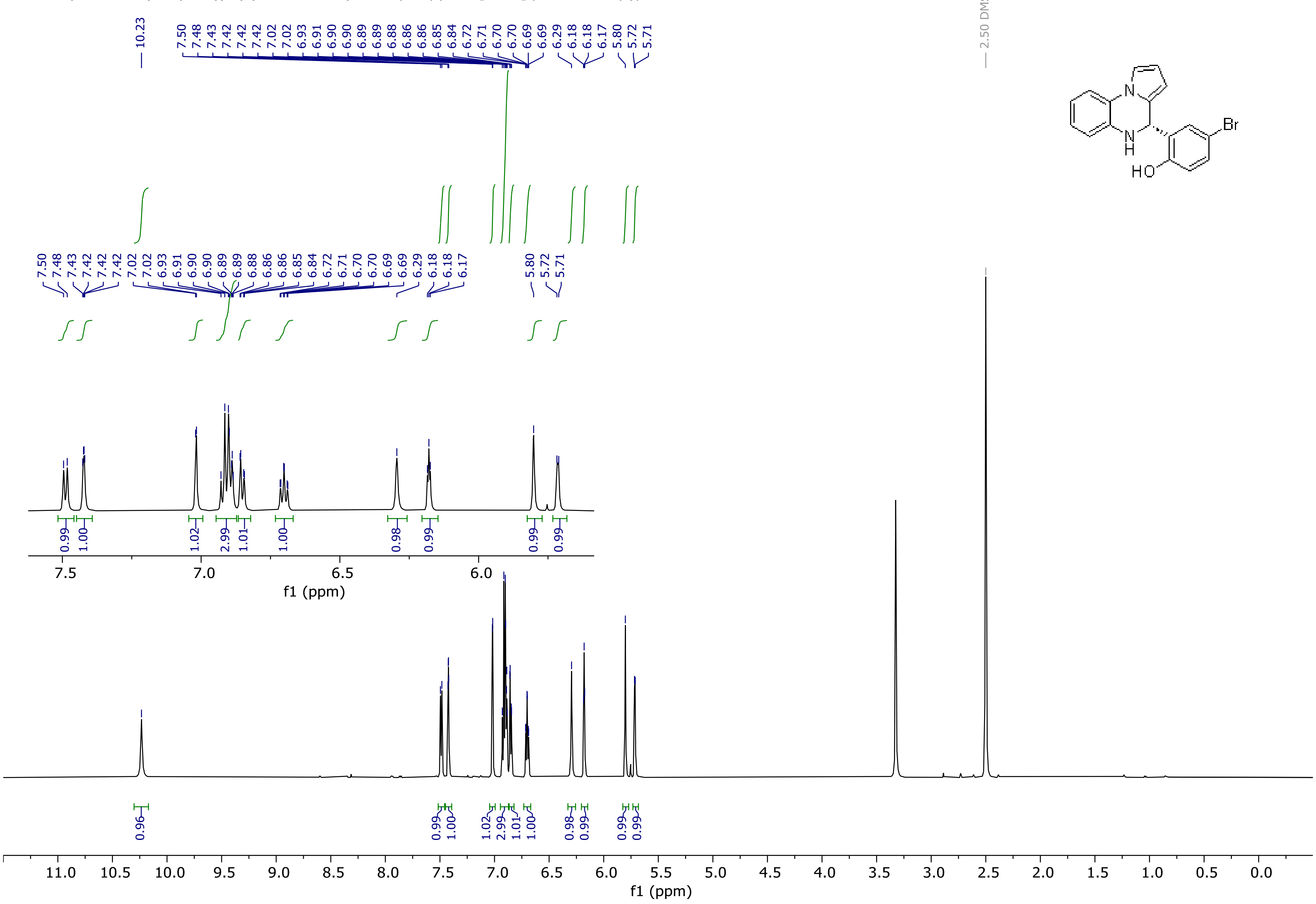

13C{1H} NMR: (151 MHz, (CD3)2SO)): (S)-4-bromo-2-(4,5-dihydropyrrolo[1,2-a]quinoxalin-4-yl)phenol 3o.

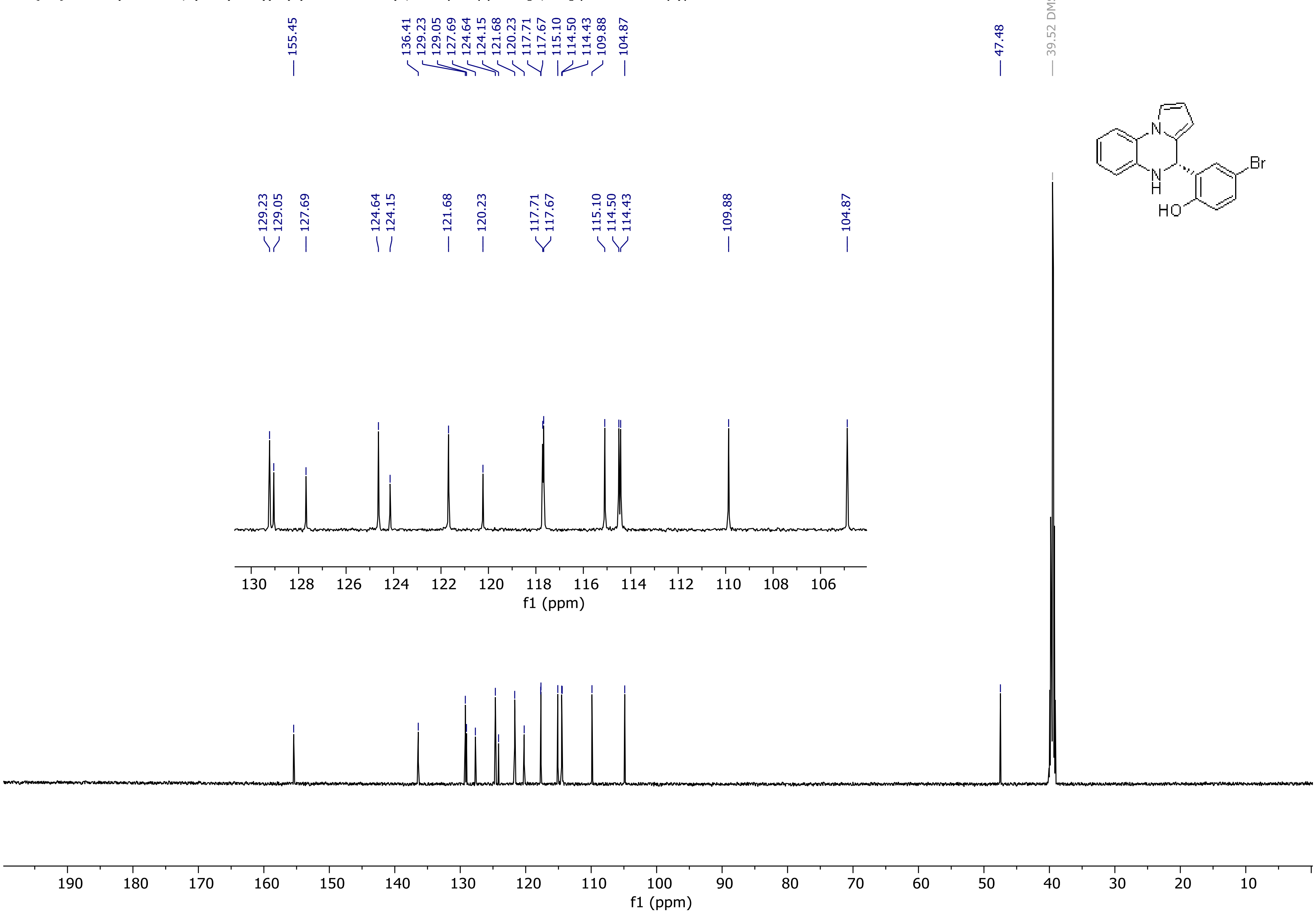

1H NMR (400 MHz, (CD3)SO): (S)-5-bromo-2-(4,5-dihydropyrrolo[1,2-a]quinoxalin-4-yl)phenol 3p.

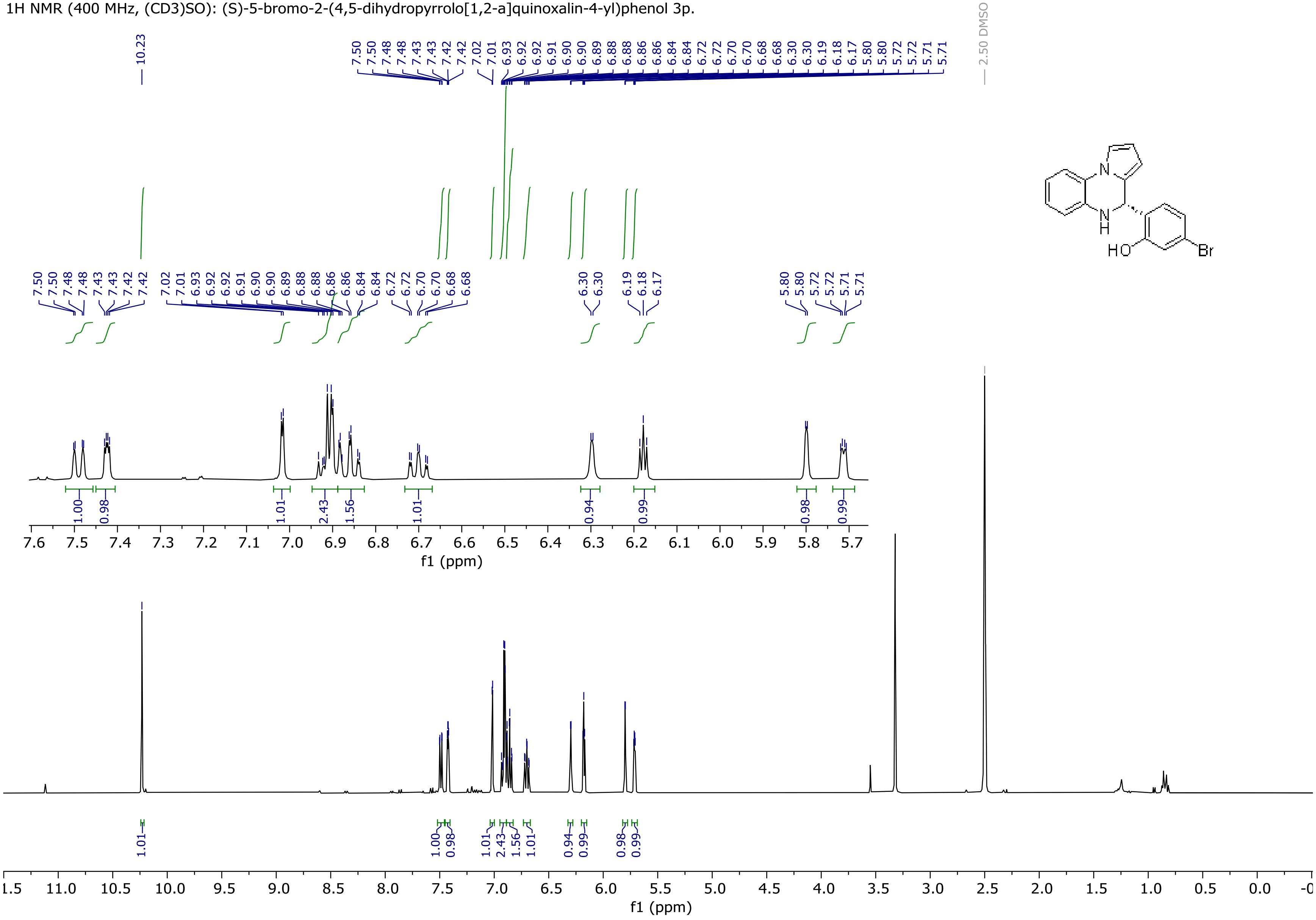

13C{1H} NMR: (101 MHz, (CD3)SO): (S)-5-bromo-2-(4,5-dihydropyrrolo[1,2-a]quinoxalin-4-yl)phenol 3p.

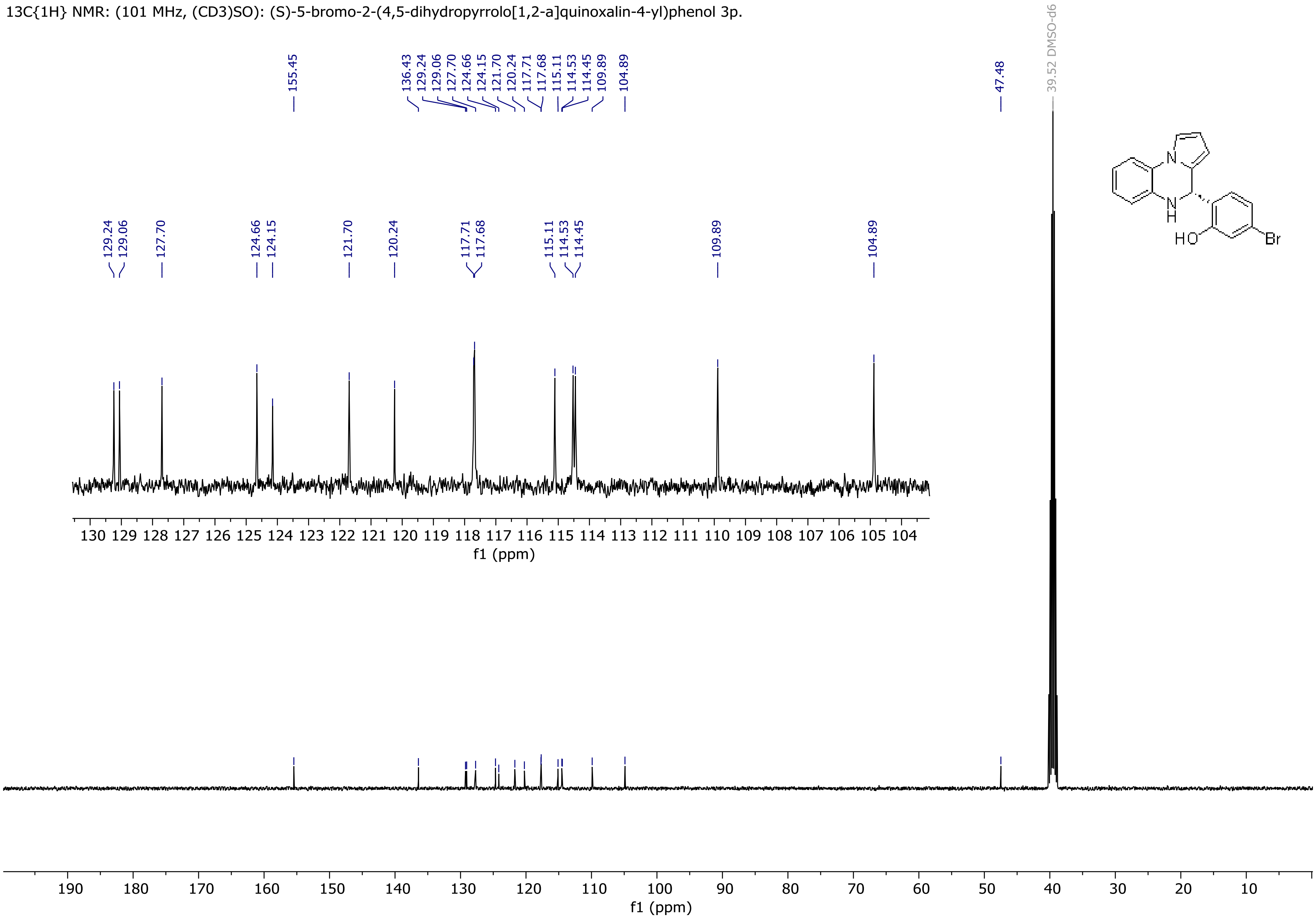

1H NMR: (600 MHz, (CD3)2SO) (S)-2-(4,5-dihydropyrrolo[1,2-a]quinoxalin-4-yl)-4-fluorophenol 3q.

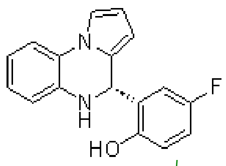

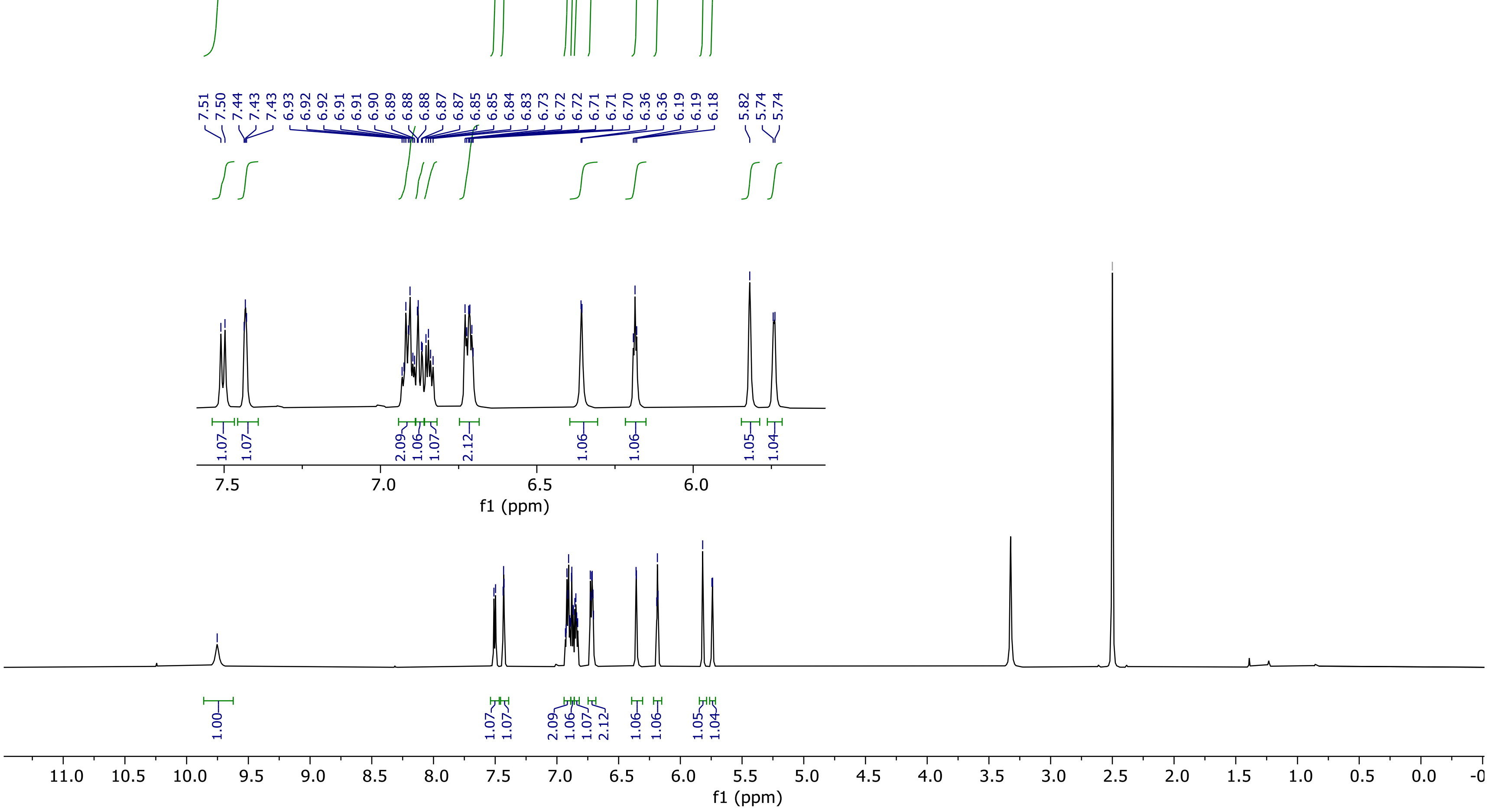

13C{1H} NMR: (151 MHz, (CD3)2SO): (S)-2-(4,5-dihydropyrrolo[1,2-a]quinoxalin-4-yl)-4-fluorophenol 3q.

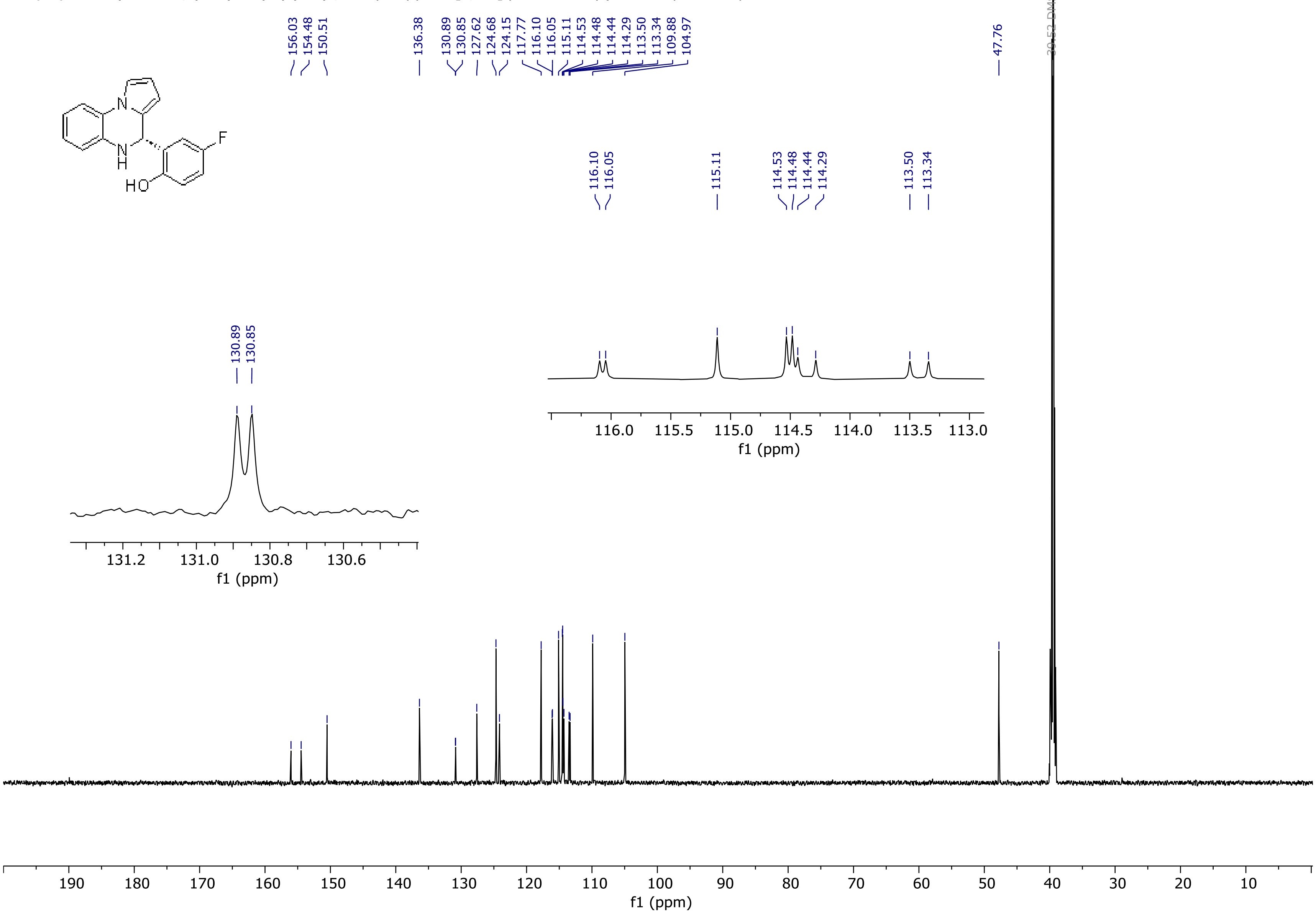

19F NMR: (376 MHz, DMSO) (S)-2-(4,5-dihydropyrrolo[1,2-a]quinoxalin-4-yl)-4-fluorophenol 3q.

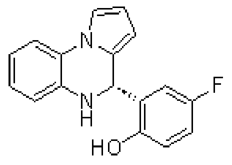

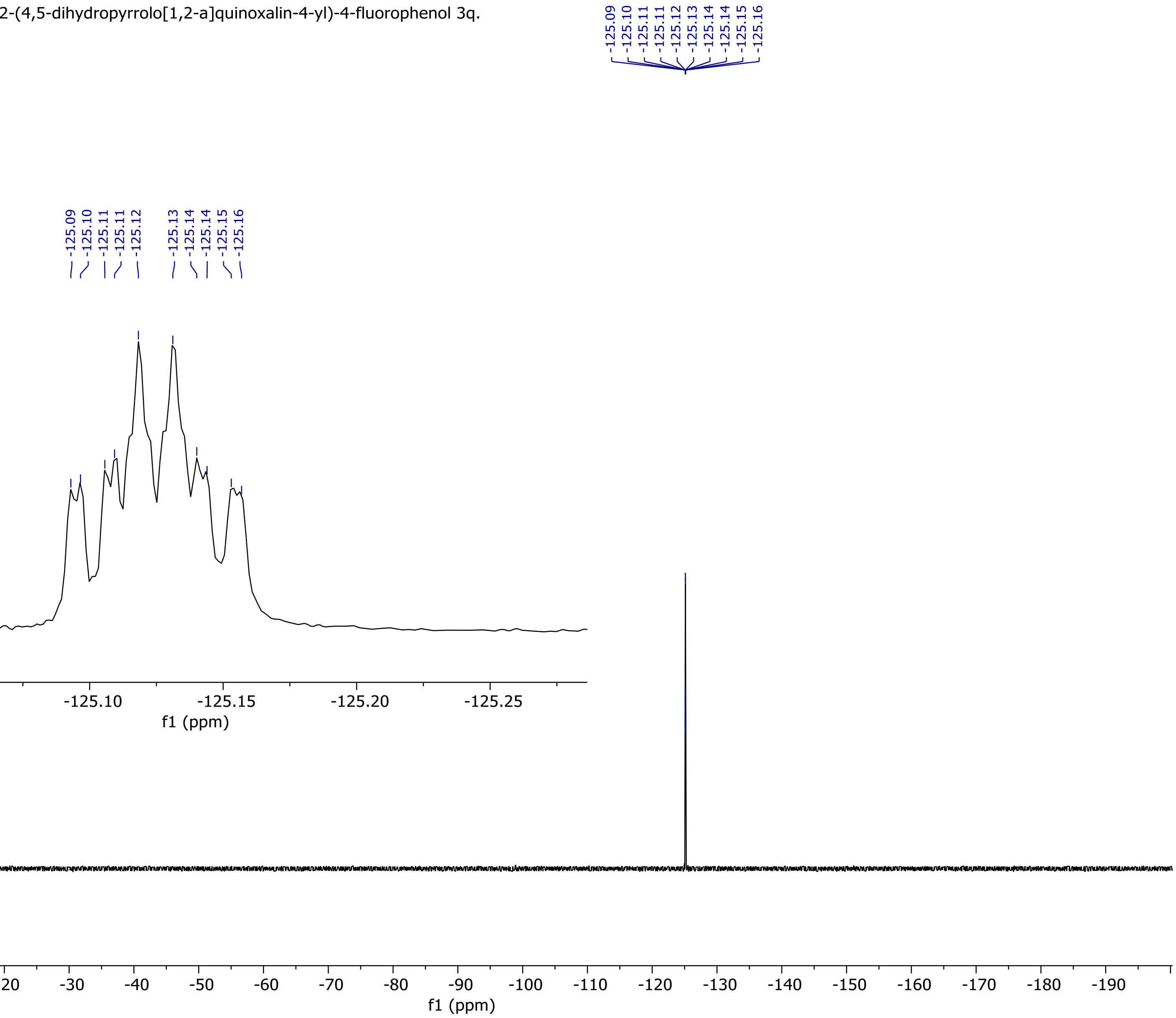

1H NMR: (600 MHz, (CD3)SO) (S)-2-(4,5-dihydropyrrolo[1,2-a]quinoxalin-4-yl)-5-fluorophenol 3r.

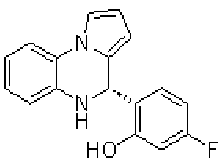

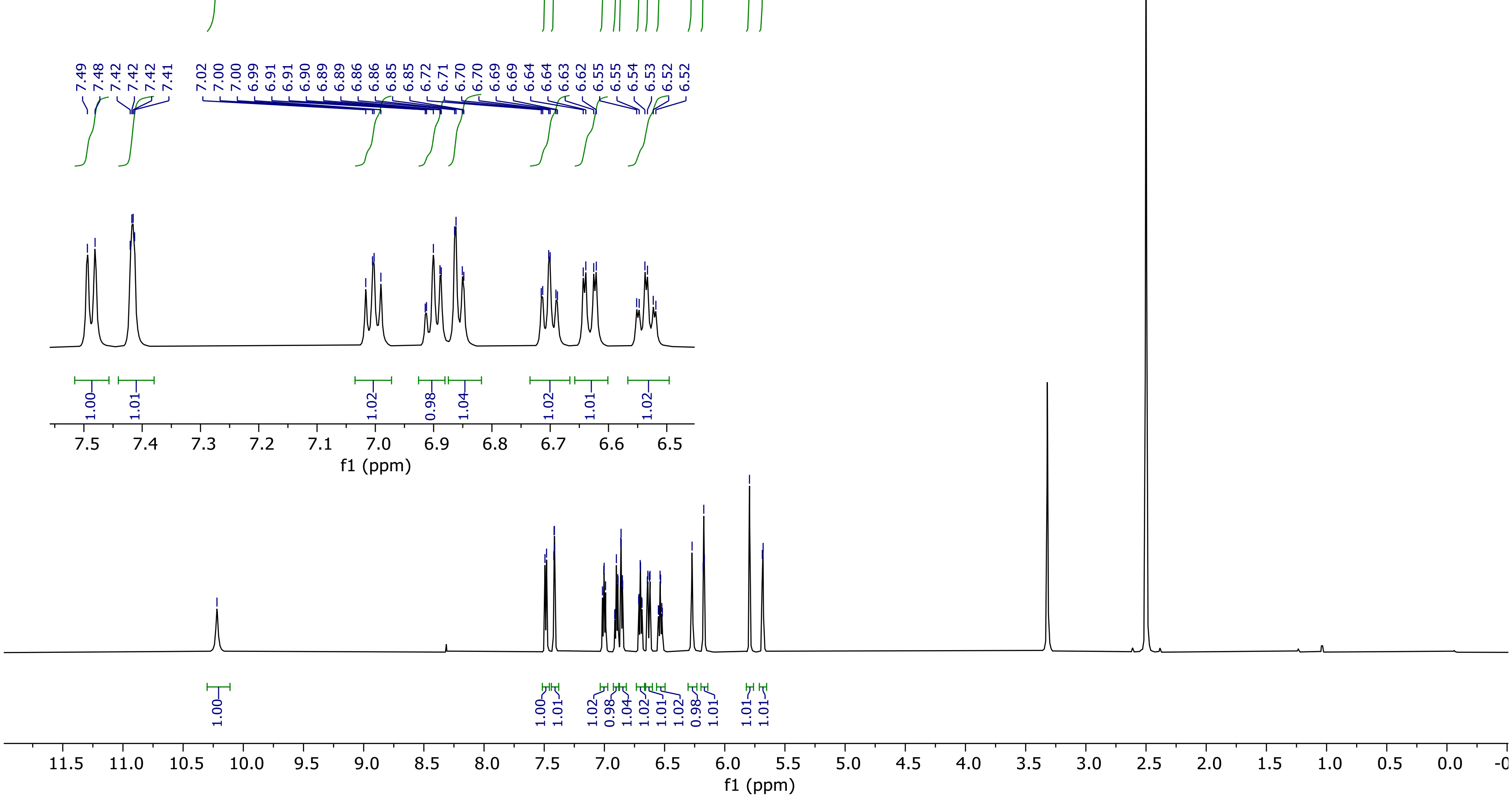

13C{1H} NMR: (151 MHz, (CD3)2SO): (S)-2-(4,5-dihydropyrrolo[1,2-a]quinoxalin-4-yl)-5-fluorophenol 3r.

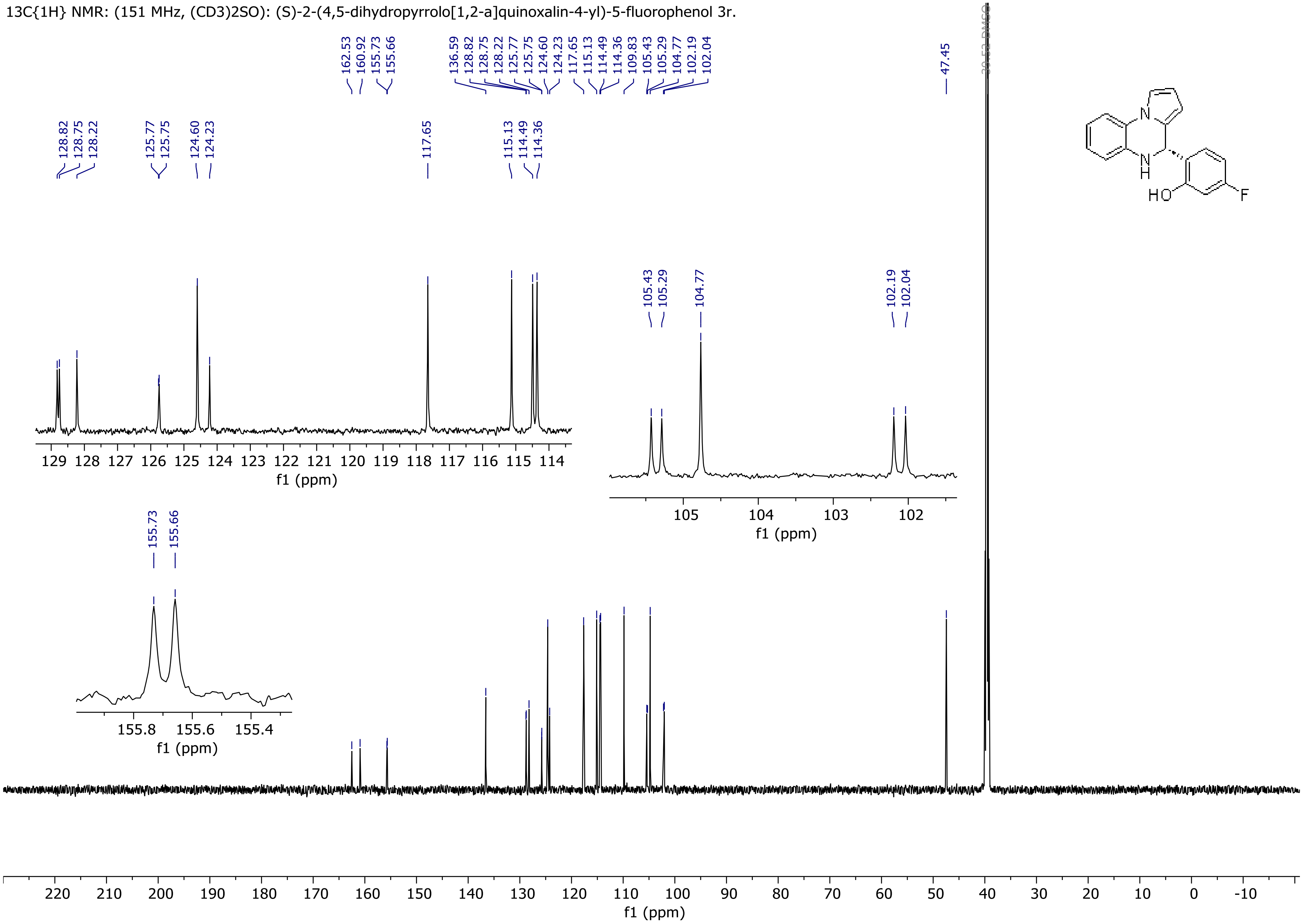

19F NMR: (376 MHz, DMSO) (S)-2-(4,5-dihydropyrrolo[1,2-a]quinoxalin-4-yl)-5-fluorophenol 3r.

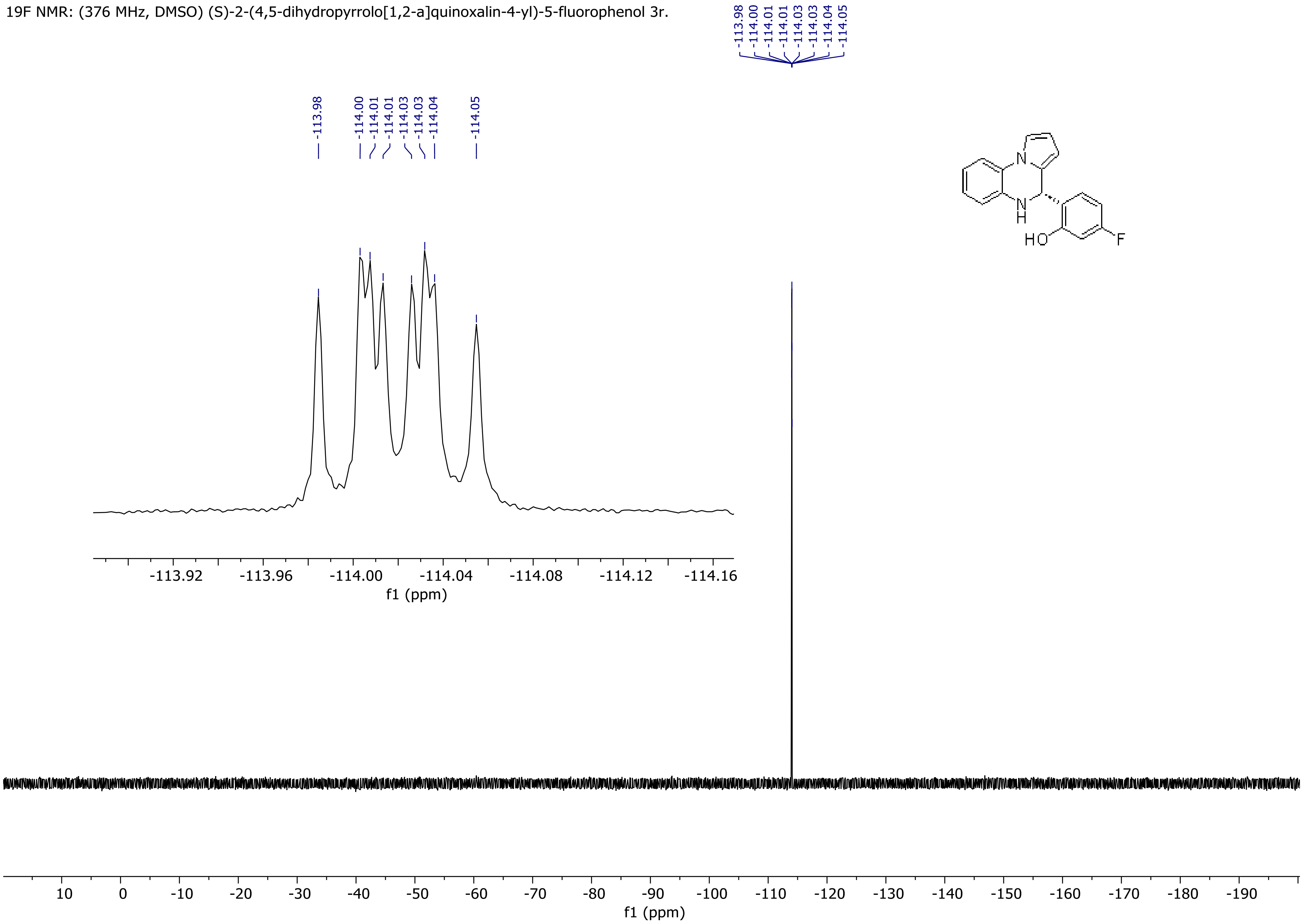

1H NMR (400 MHz, CDCl3): (S)-3-(4,5-dihydropyrrolo[1,2-a]quinoxalin-4-yl)naphthalen-2-ol 3s.

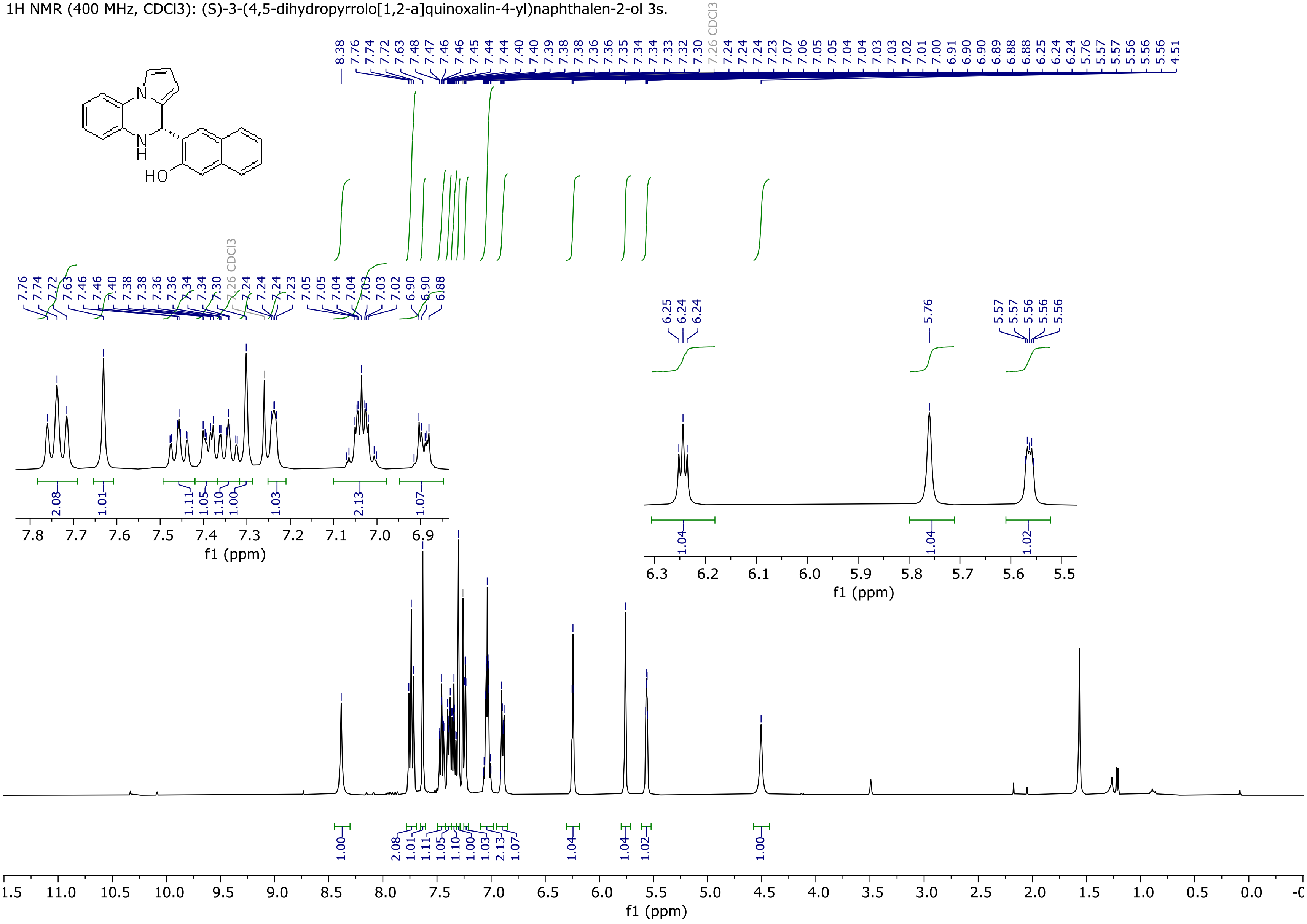

13C{1H} NMR: (101 MHz, CDCl3): (S)-3-(4,5-dihydropyrrolo[1,2-a]quinoxalin-4-yl)naphthalen-2-ol 3s.

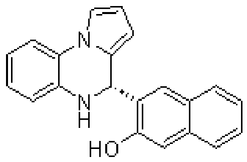

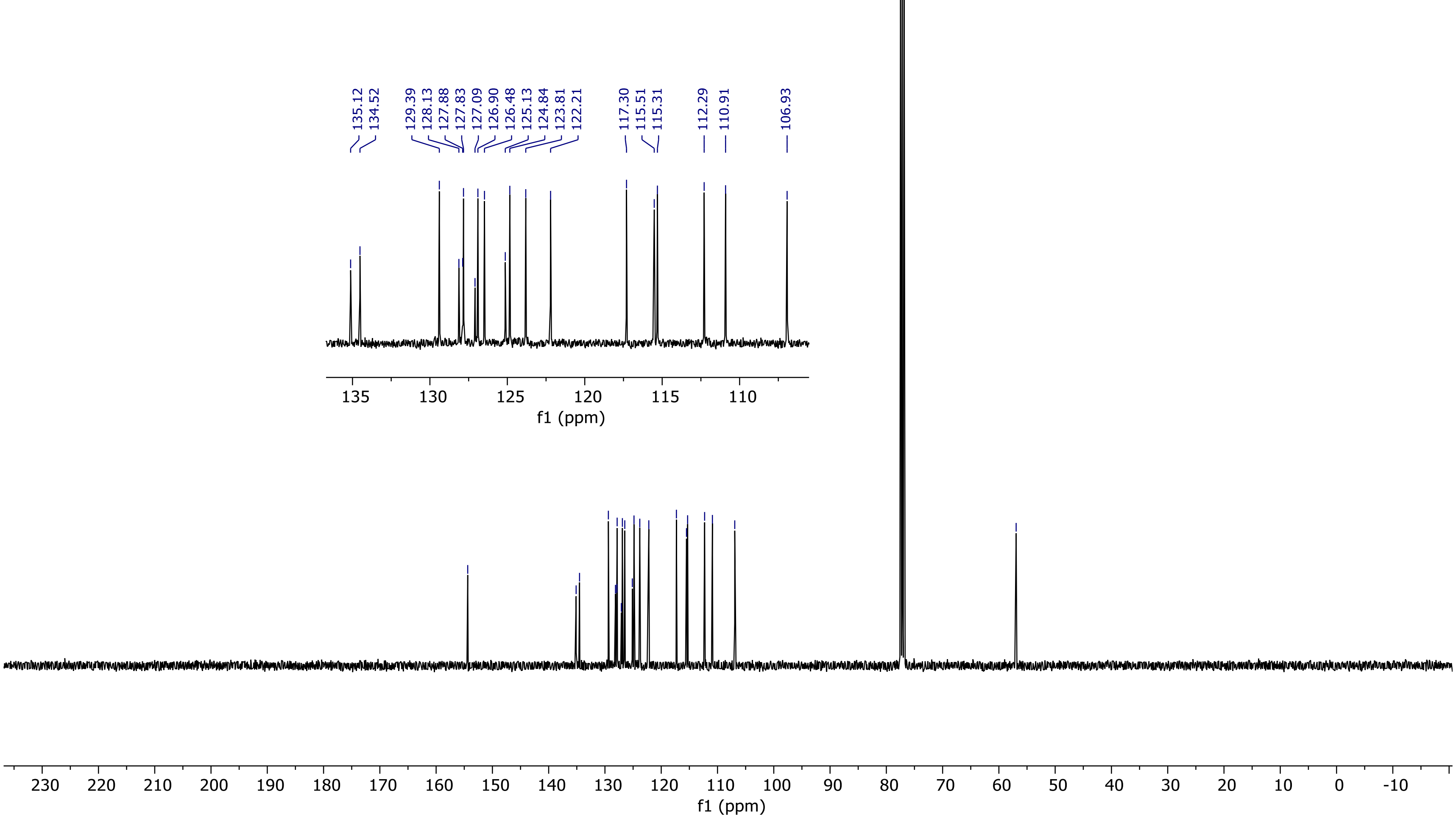

1H NMR: (600 MHz, (CD3)2SO): (S)-4-(1H-indol-2-yl)-4,5-dihydropyrrolo[1,2-a]quinoxaline 3t.

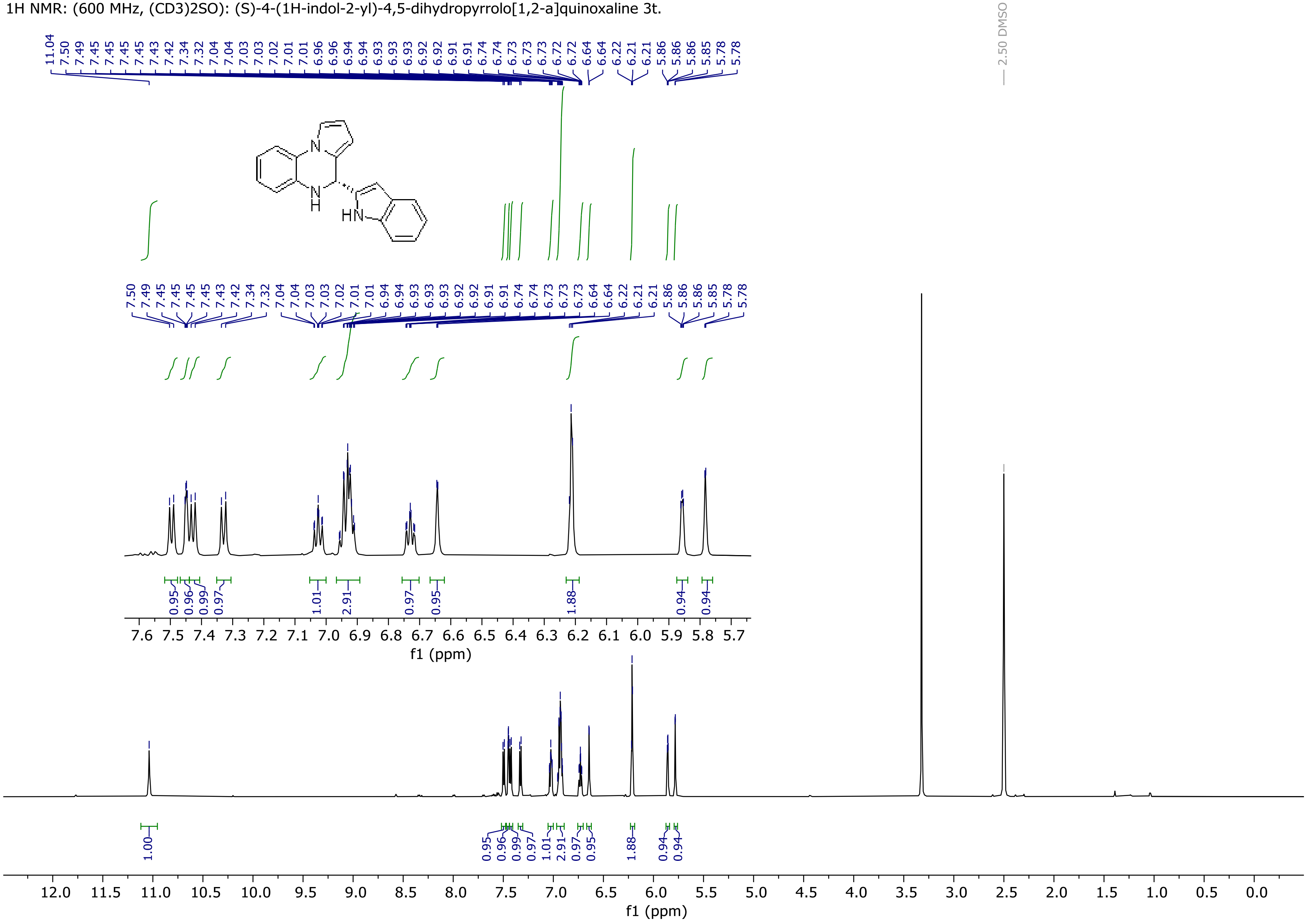

13C{1H} NMR: (151 MHz, (CD3)2SO): (S)-4-(1H-indol-2-yl)-4,5-dihydropyrrolo[1,2-a]quinoxaline 3t.

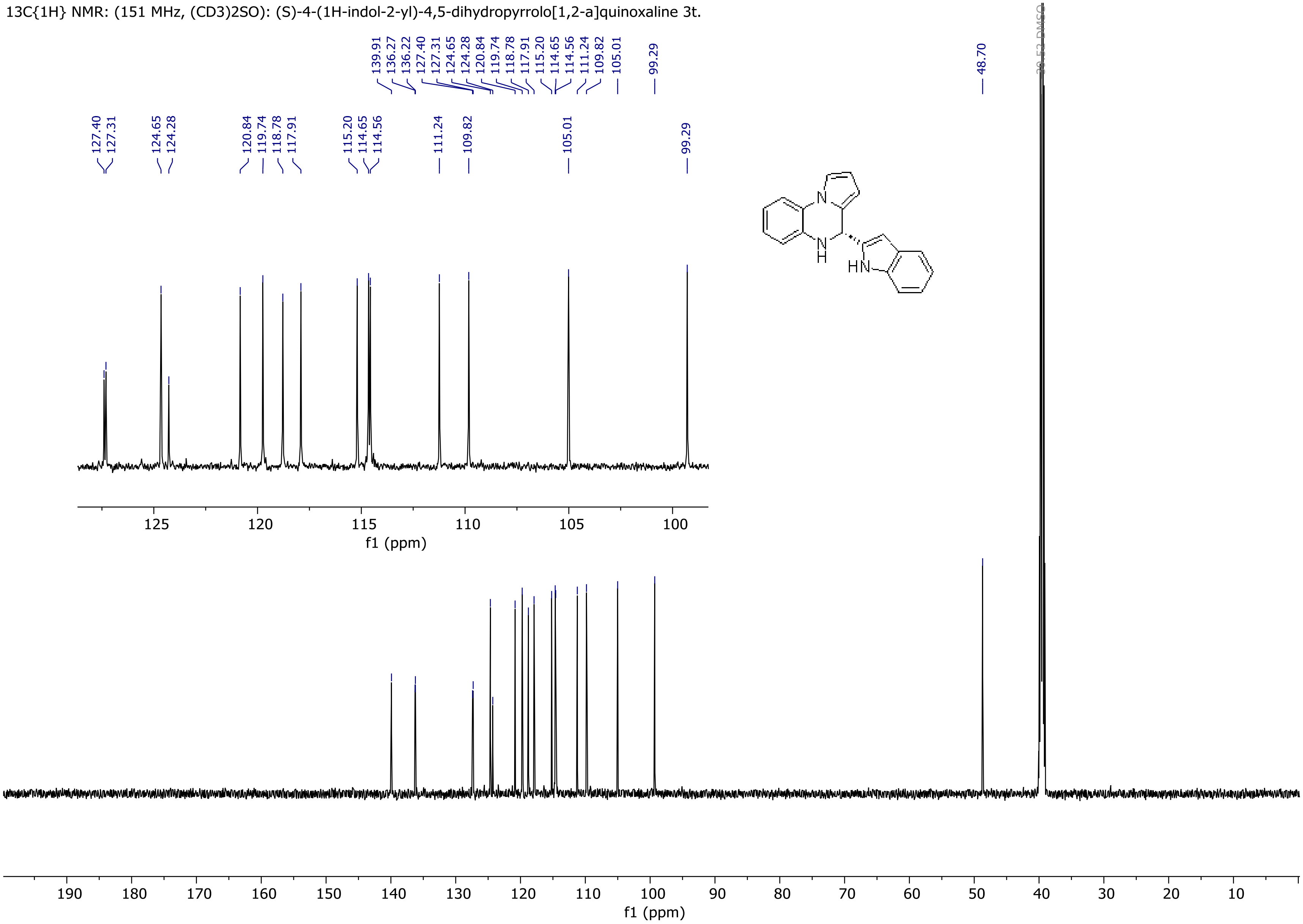

1H NMR (400 MHz, CDCl3): 1-benzyl-5'H-spiro[indoline-3,4'-pyrrolo[1,2-a]quinoxalin]-2-one 3u.

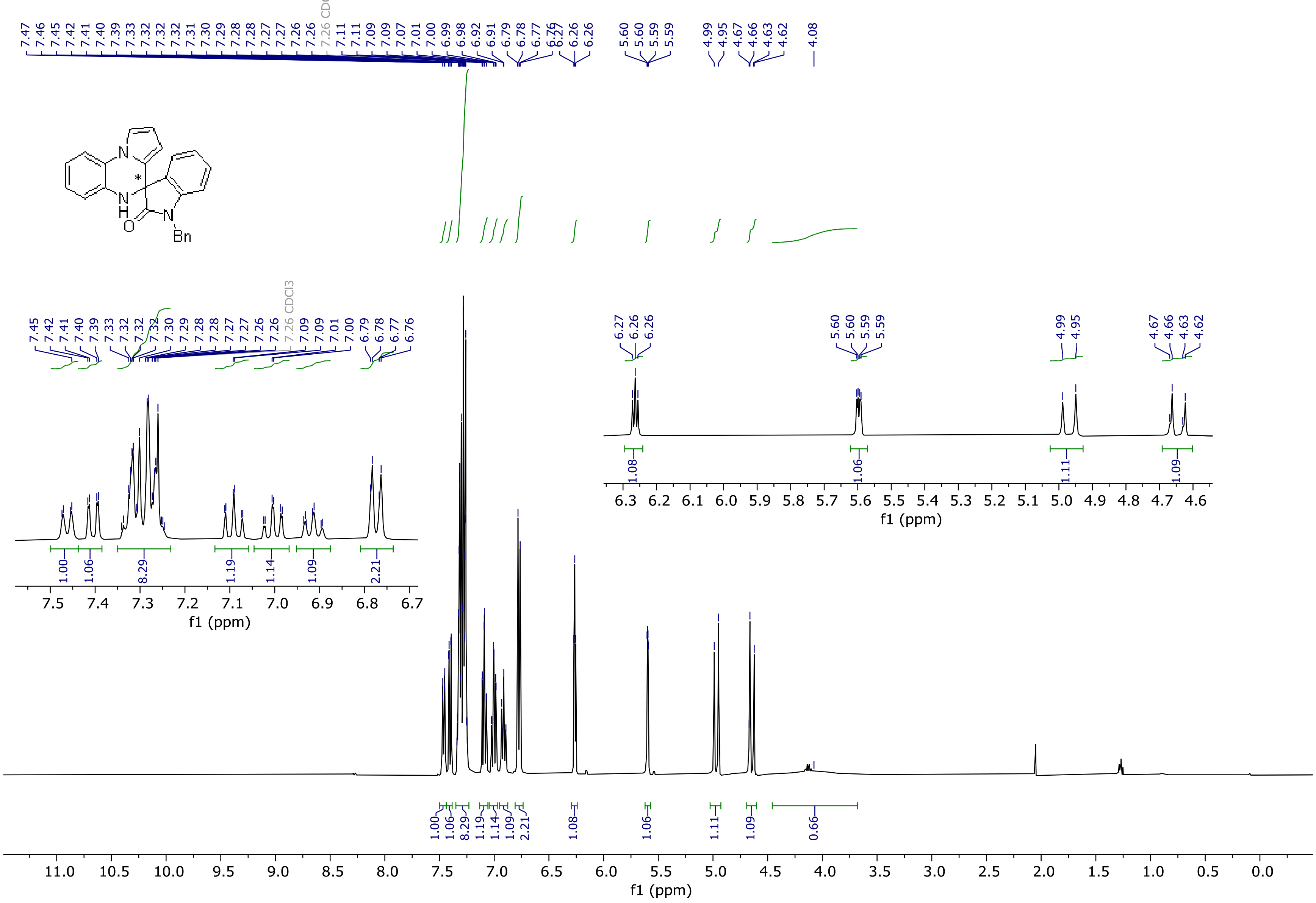

13C{1H} NMR: (101 MHz, CDCl3): 1-benzyl-5'H-spiro[indoline-3,4'-pyrrolo[1,2-a]quinoxalin]-2-one 3u.

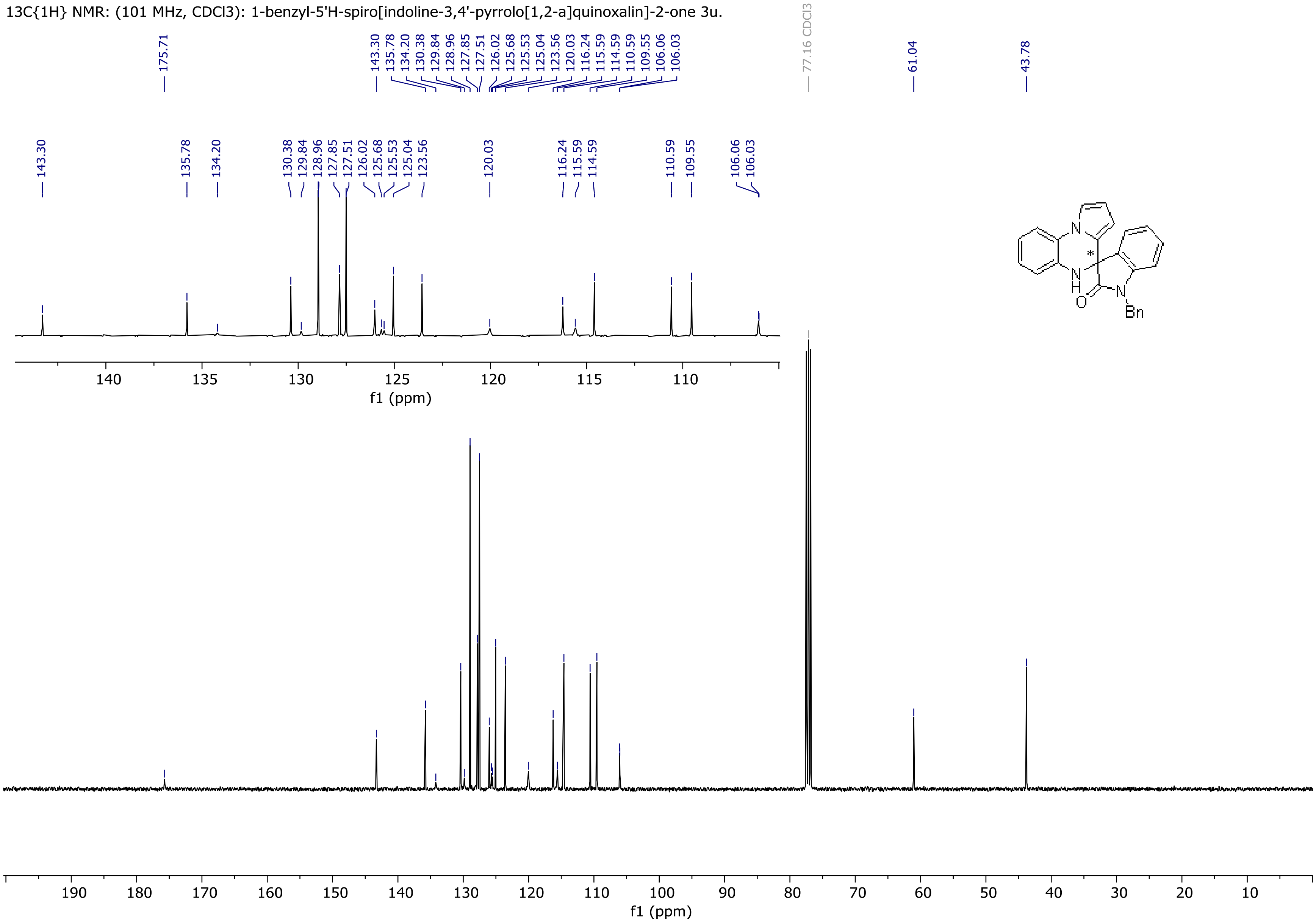

1H NMR: (600 MHz, (CD3)2SO) (S)-2-(9-methyl-4,5-dihydropyrrolo[1,2-a]quinoxalin-4-yl)phenol 3y.

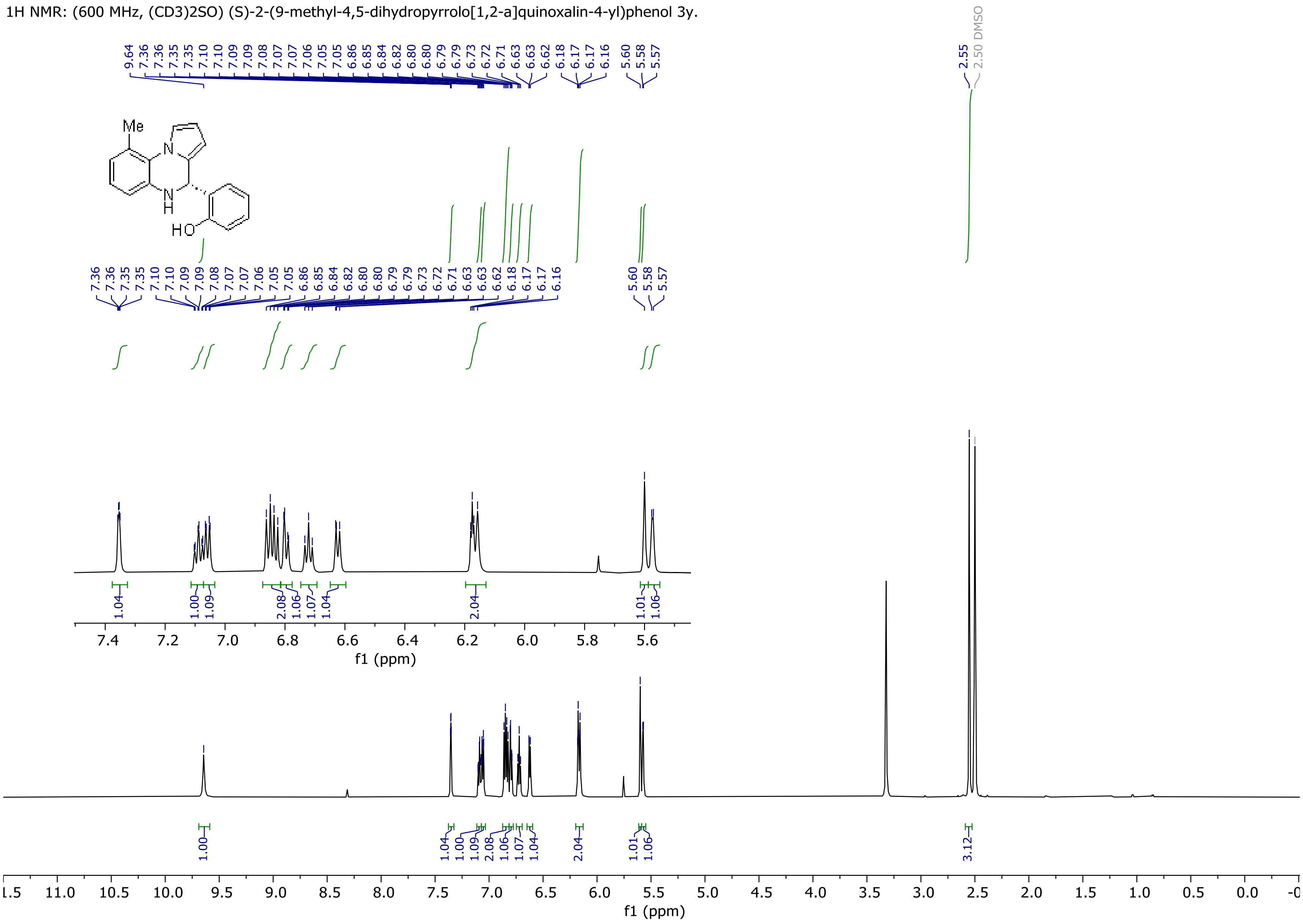

13C{1H} NMR: (151 MHz, (CD3)2SO): (S)-2-(9-methyl-4,5-dihydropyrrolo[1,2-a]quinoxalin-4-yl)phenol 3y.

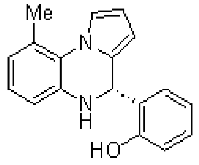

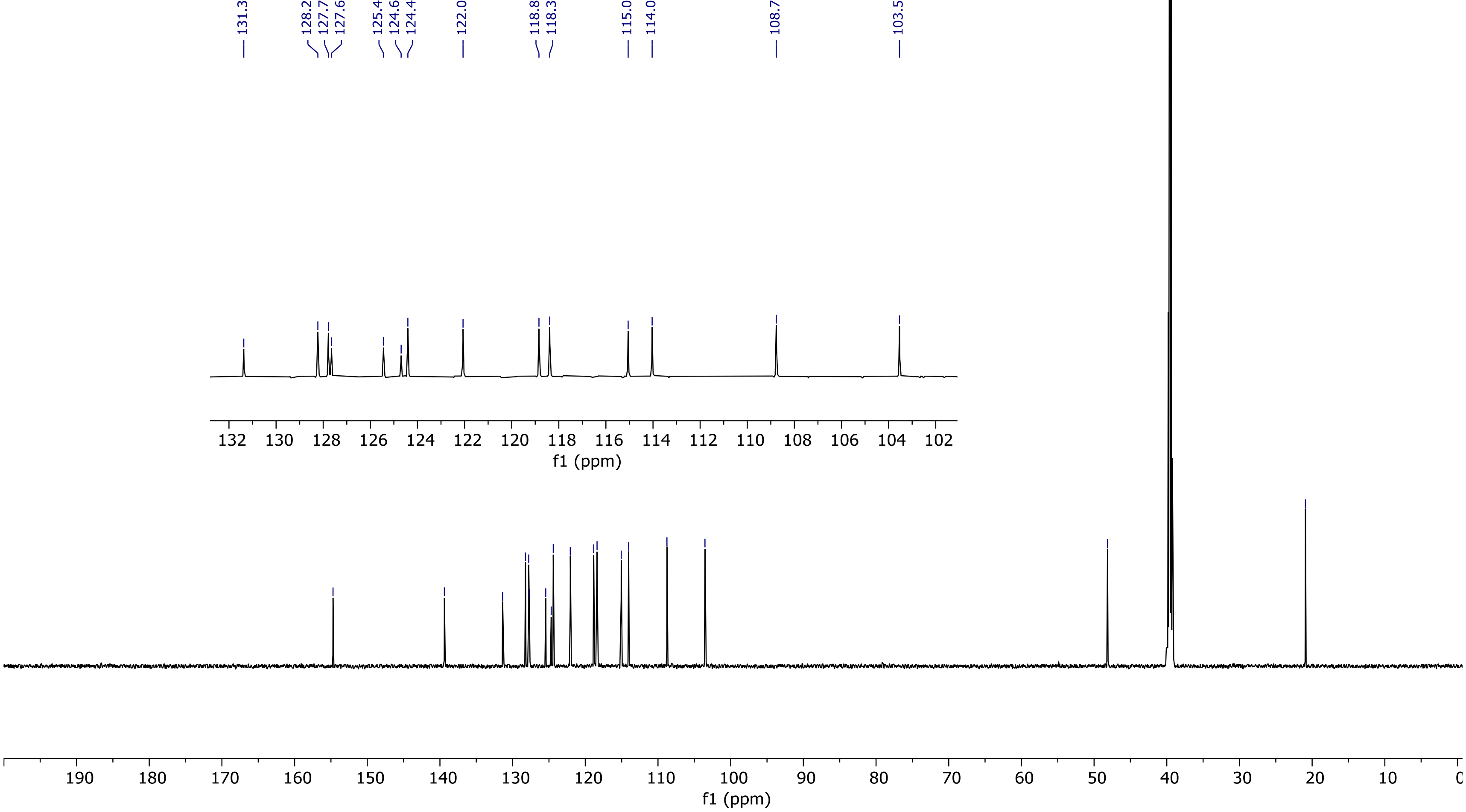

1H NMR (400 MHz, CDCl3): (S)-2-(9-methoxy-4,5-dihydropyrrolo[1,2-a]quinoxalin-4-yl)phenol 3z.

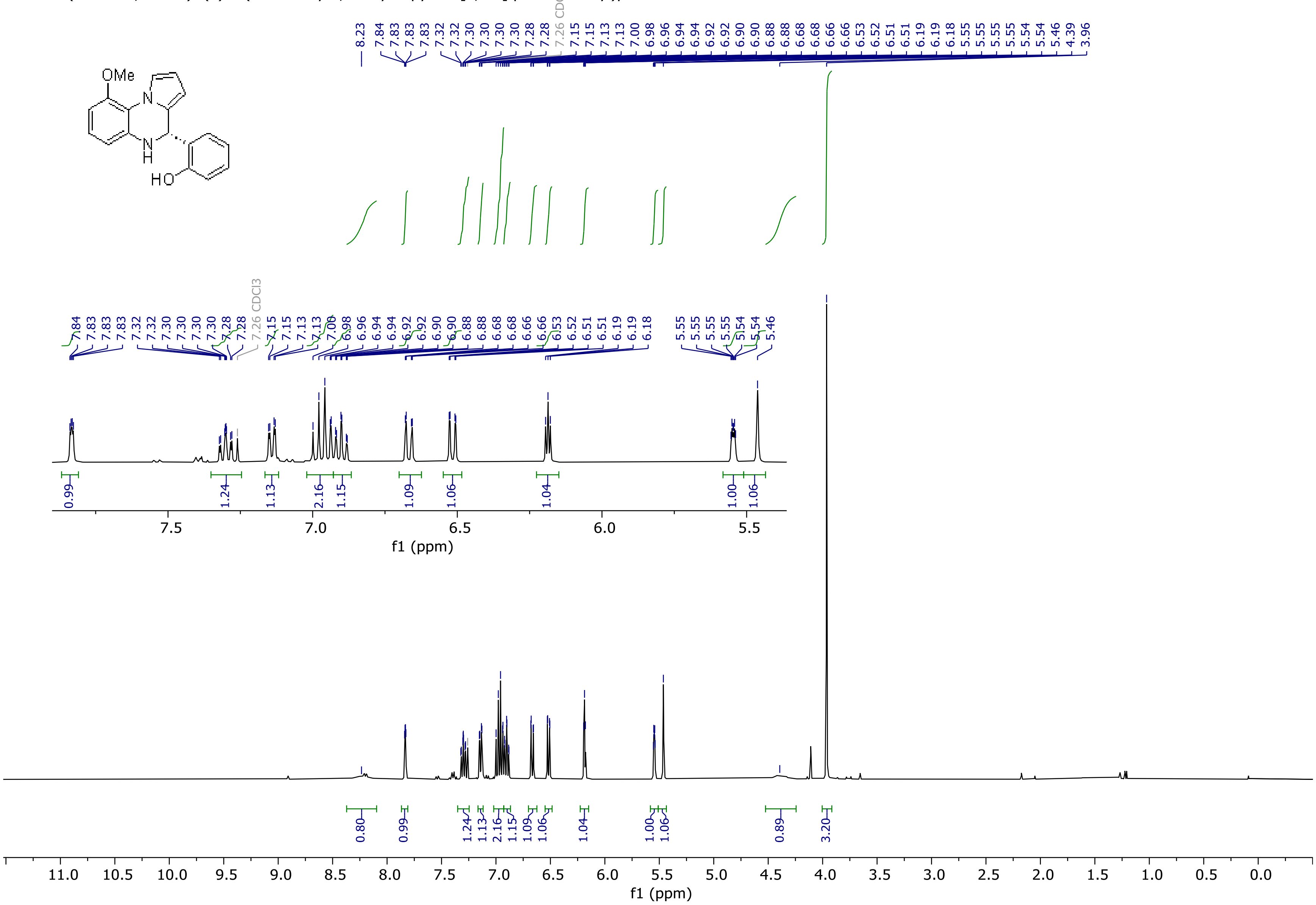

13C{1H} NMR: (151 MHz, (CD3)SO): (S)-2-(9-methoxy-4,5-dihydropyrrolo[1,2-a]quinoxalin-4-yl)phenol 3z.

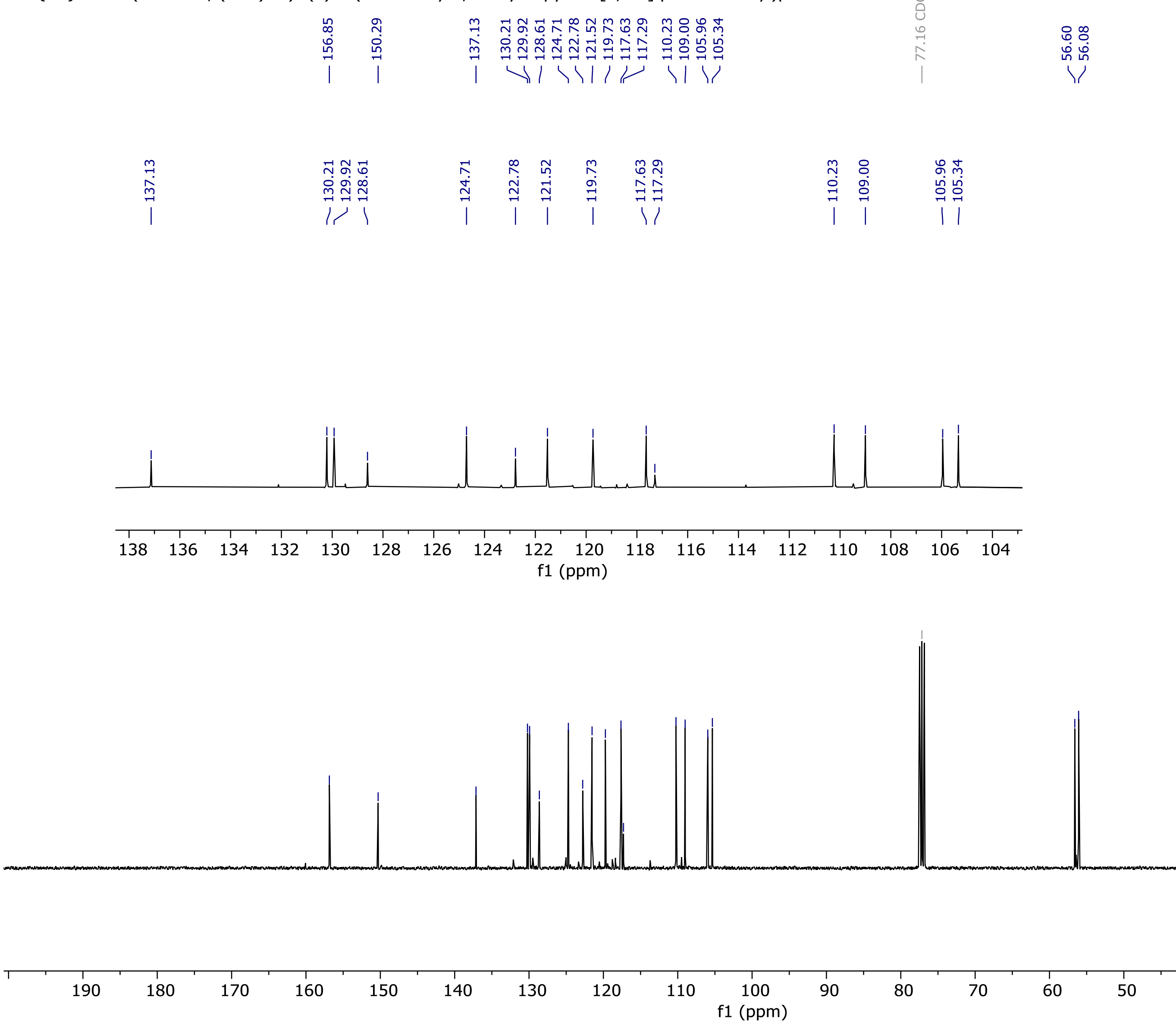

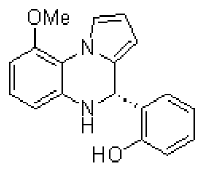

1H NMR (400 MHz, CDCl3): (S)-2-(8-methoxy-4,5-dihydropyrrolo[1,2-a]quinoxalin-4-yl)phenol 3aa.

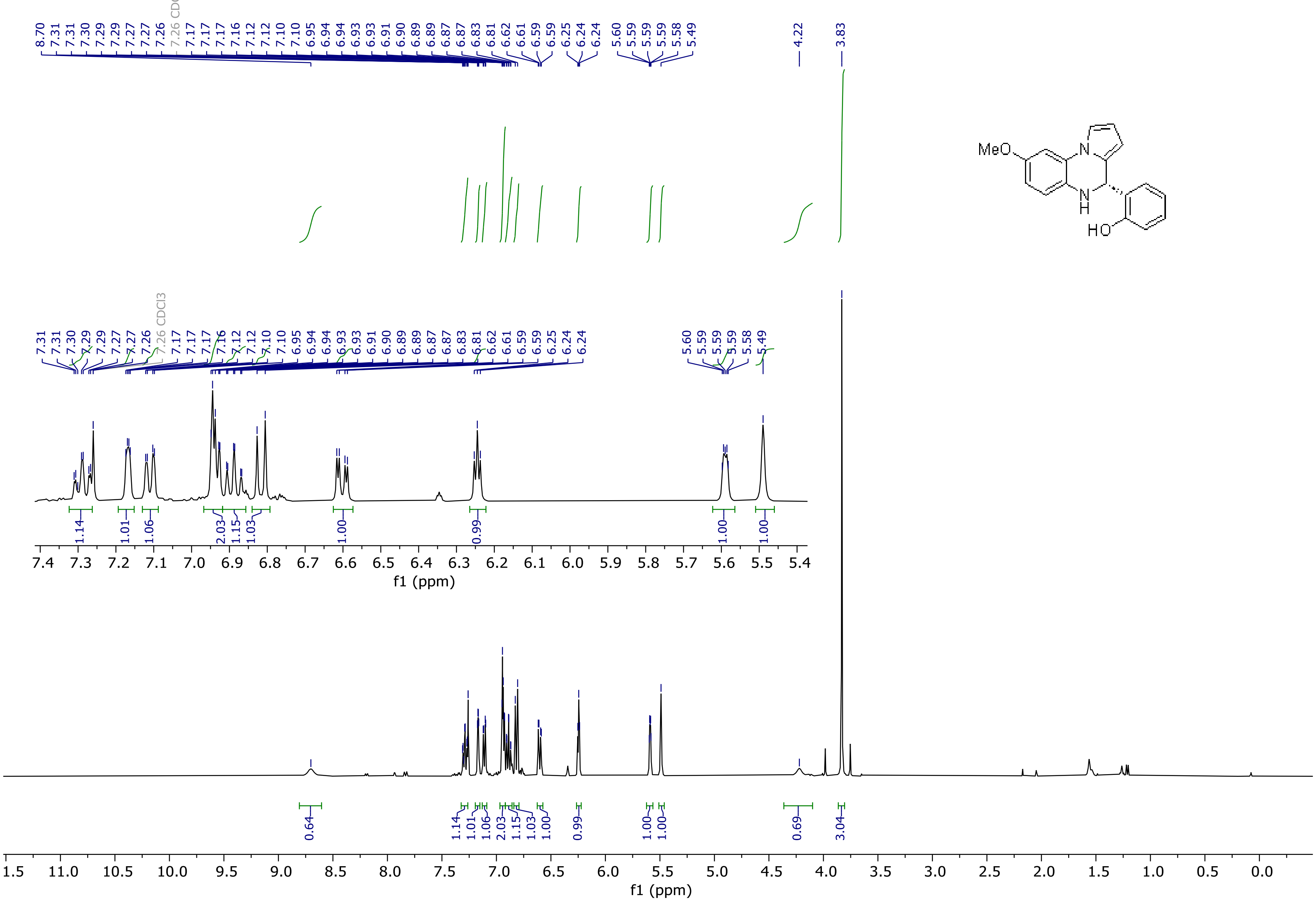

13C{1H} NMR: (101 MHz, CDCl3): (S)-2-(8-methoxy-4,5-dihydropyrrolo[1,2-a]quinoxalin-4-yl)phenol 3aa.

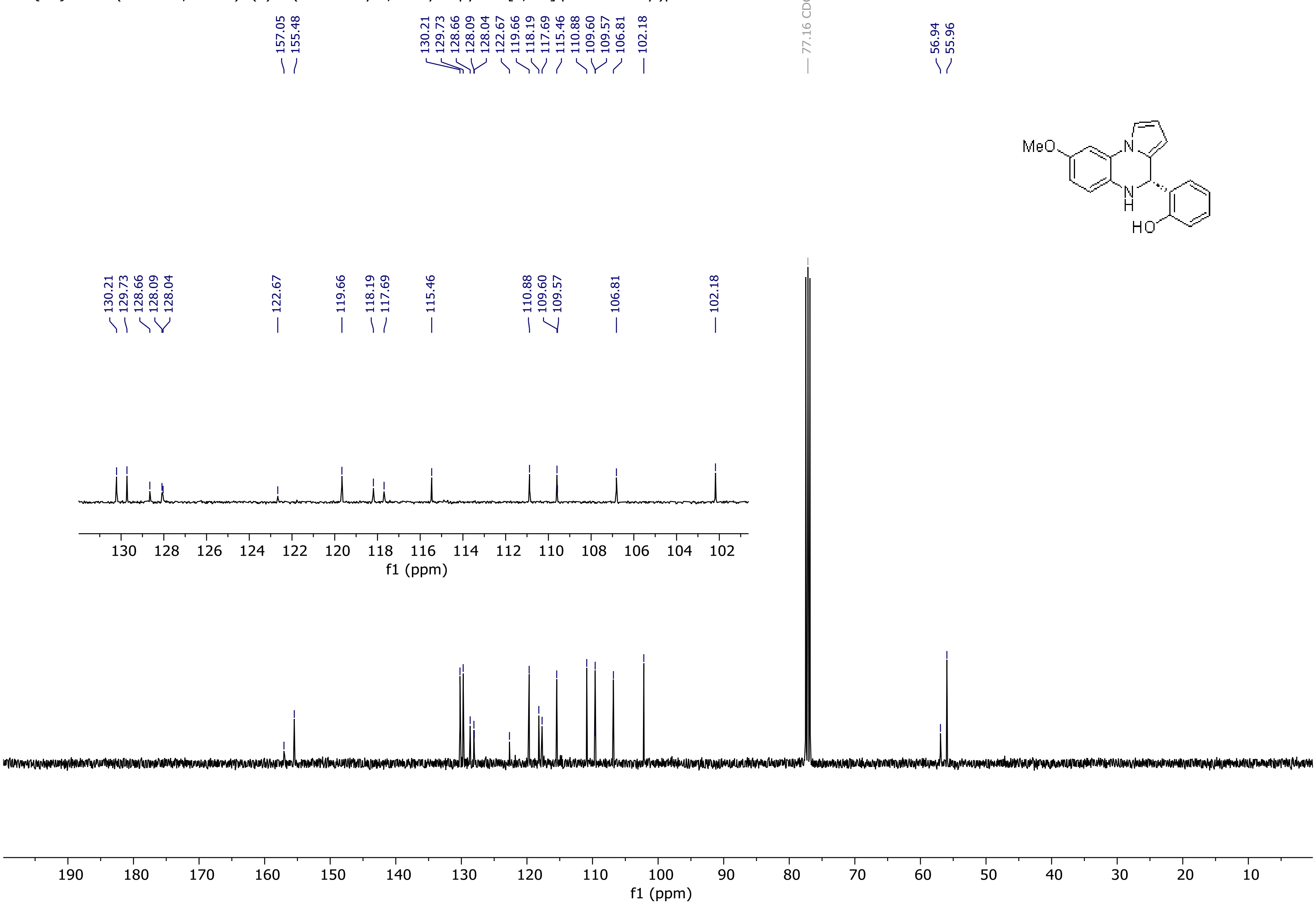

1H NMR (400 MHz, CDCl3): (S)-2-(8-chloro-4,5-dihydropyrrolo[1,2-a]quinoxalin-4-yl)phenol 3bb.

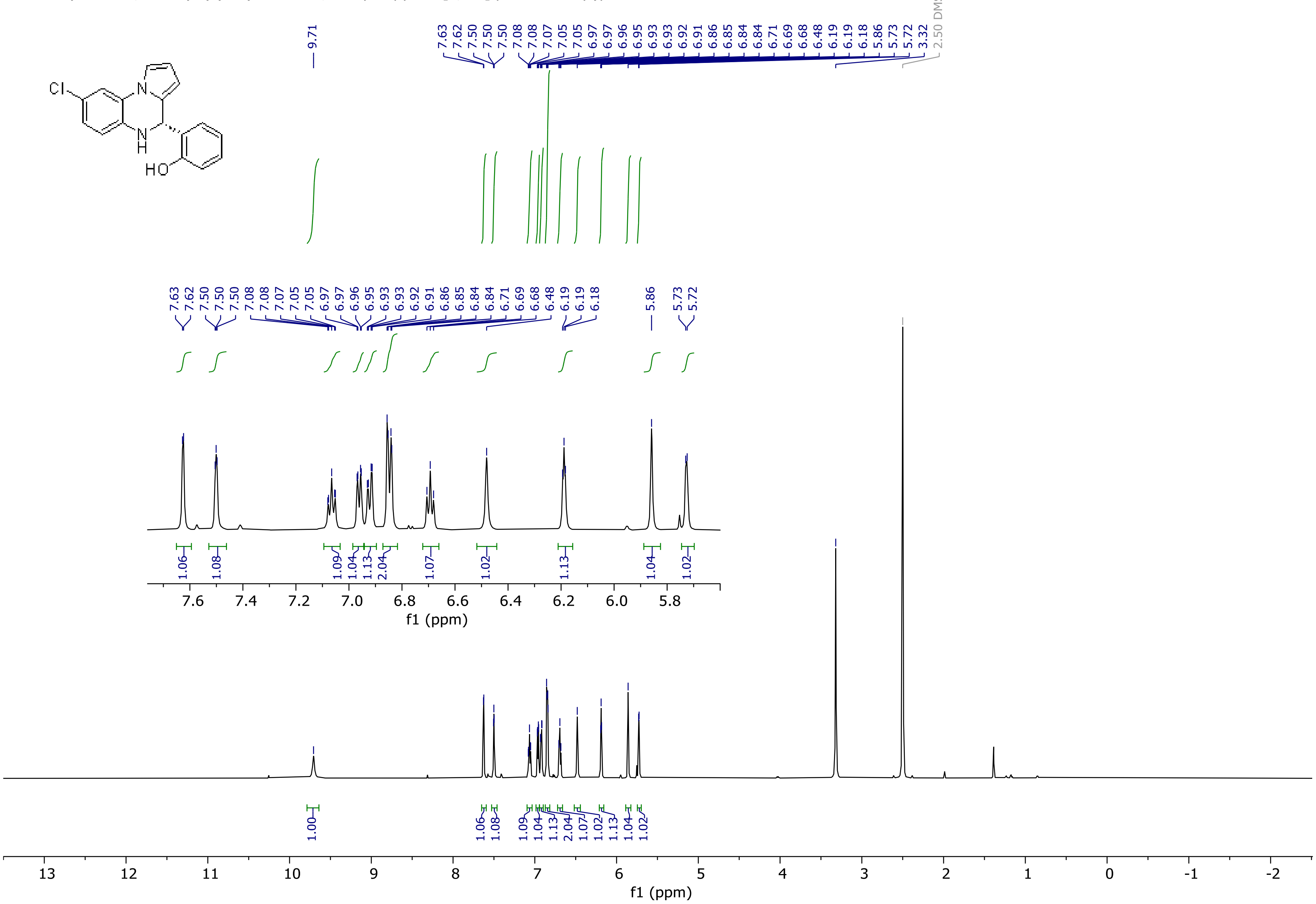

13C{1H} NMR: (101 MHz, (CD3)SO): (S)-2-(8-chloro-4,5-dihydropyrrolo[1,2-a]quinoxalin-4-yl)phenol 3bb.

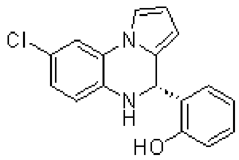

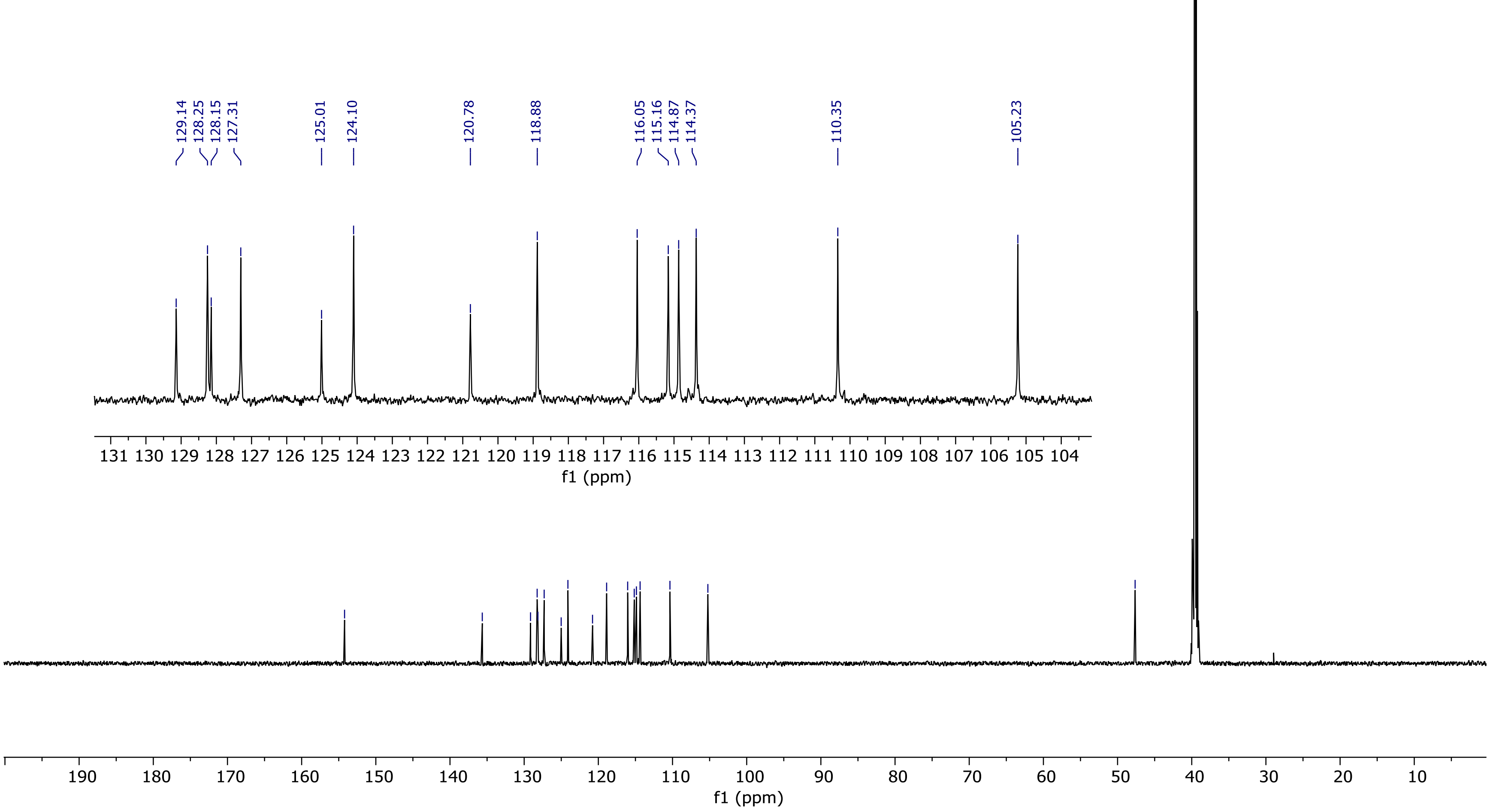

1H NMR (400 MHz, CDCl3): (S)-2-(7-bromo-4,5-dihydropyrrolo[1,2-a]quinoxalin-4-yl)phenol 3cc.

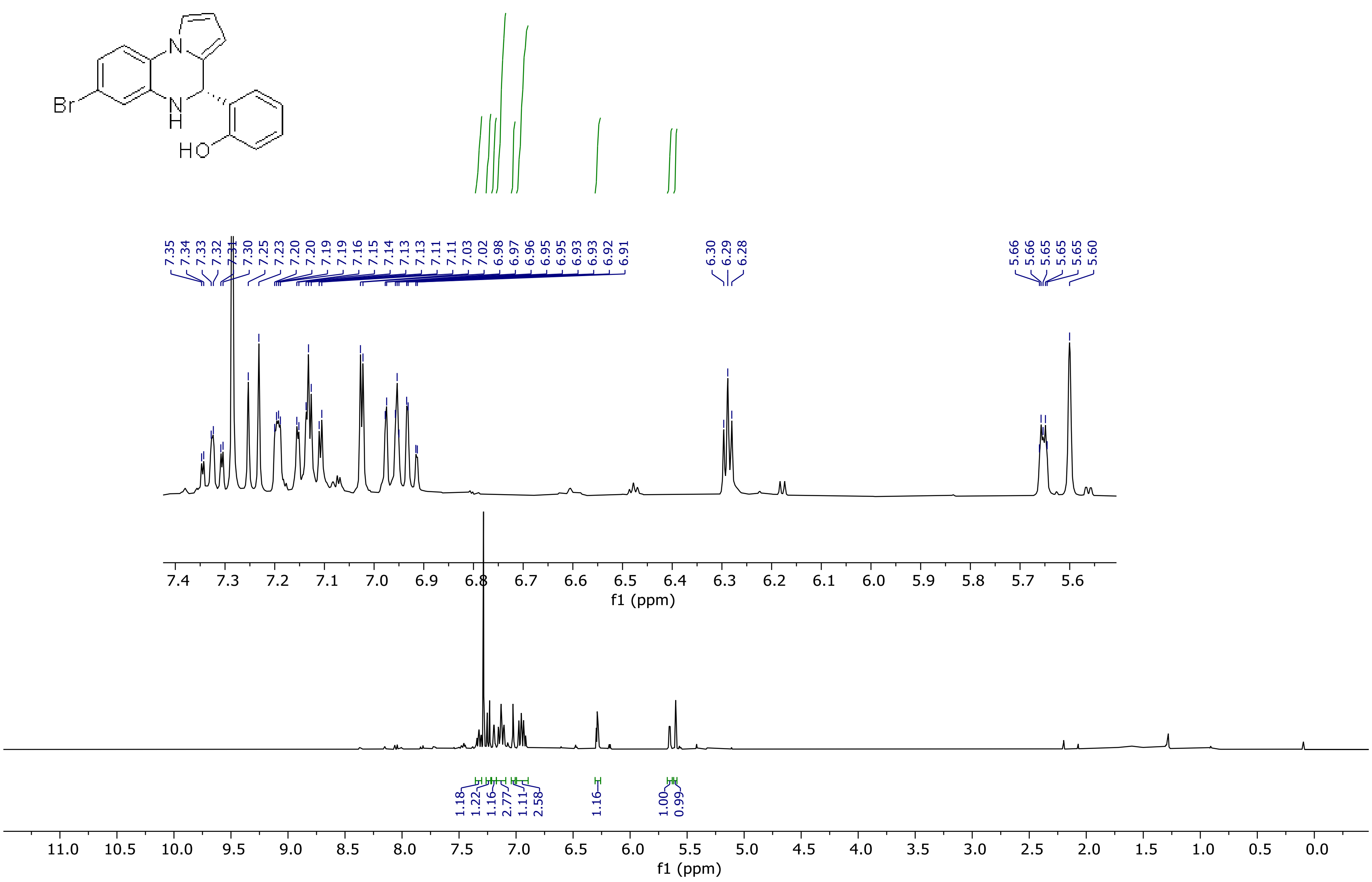

13C{1H} NMR: (101 MHz, CDCl3): (S)-2-(7-bromo-4,5-dihydropyrrolo[1,2-a]quinoxalin-4-yl)phenol 3cc.

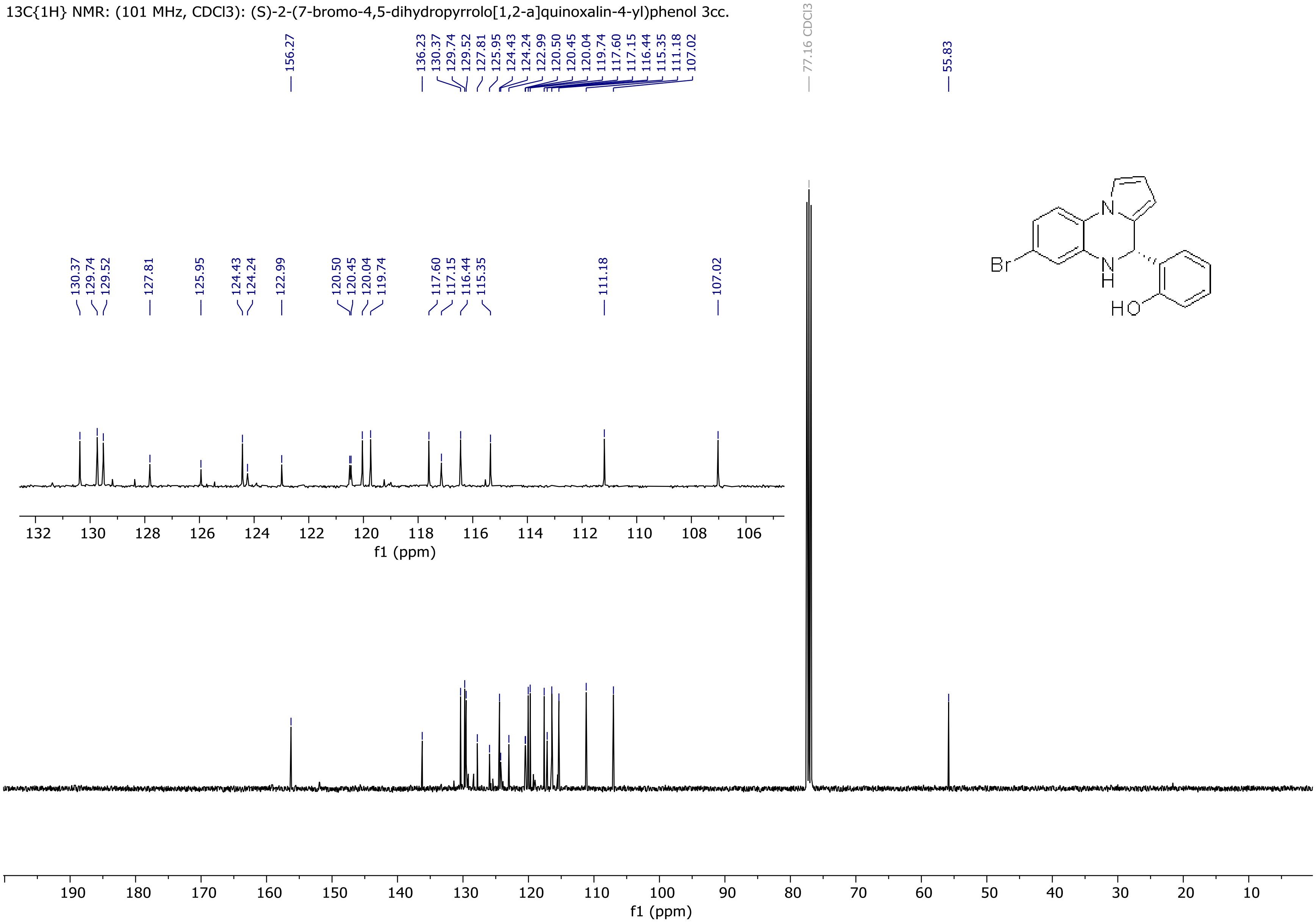

1H NMR (400 MHz, CDCl3): (S)-2-(7-(trifluoromethyl)-4,5-dihydropyrrolo[1,2-a]quinoxalin-4-yl)phenol 3dd.

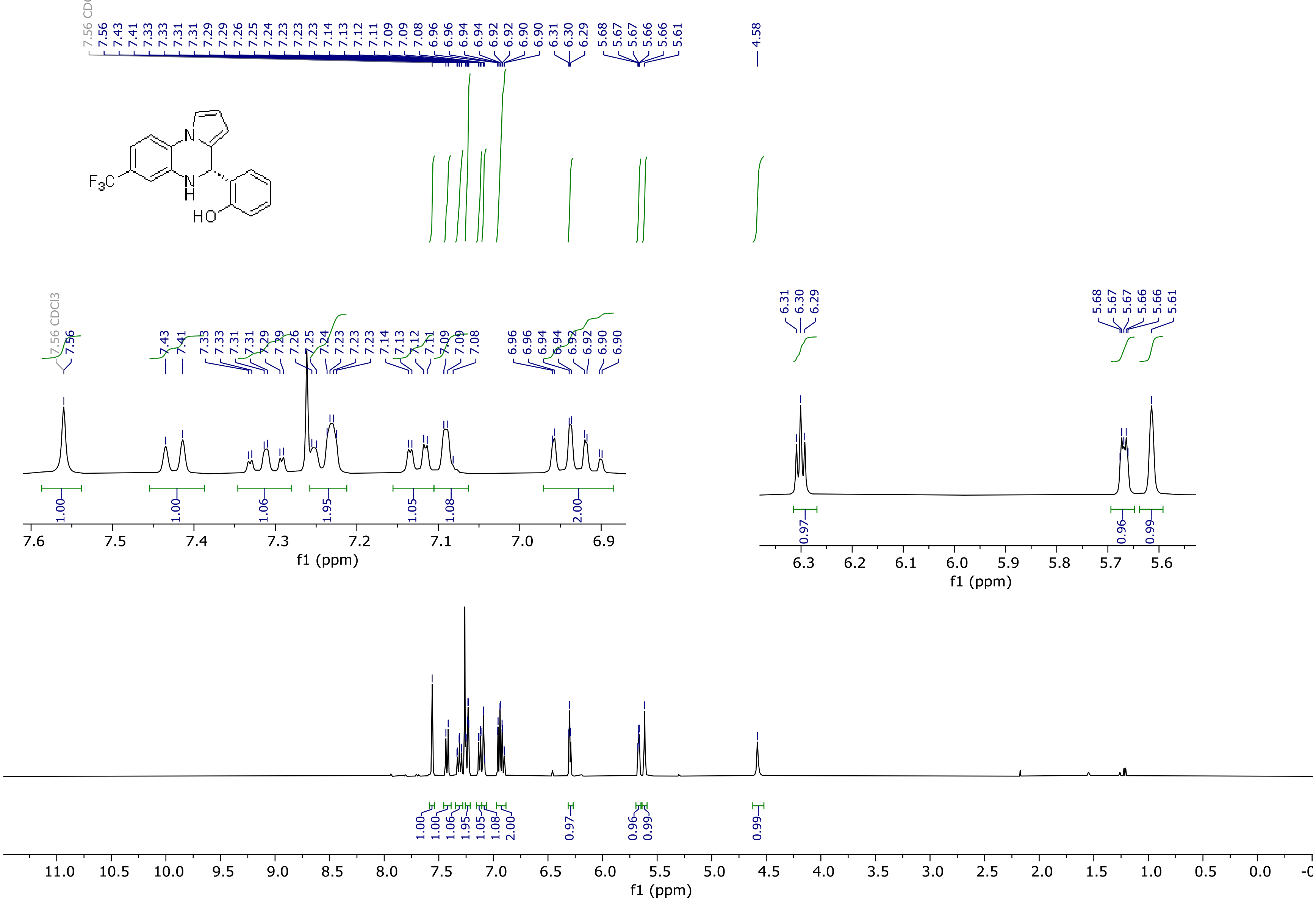

13C{1H} NMR: (101 MHz, CDCl3): (S)-2-(7-(trifluoromethyl)-4,5-dihydropyrrolo[1,2-a]quinoxalin-4-yl)phenol 3dd.

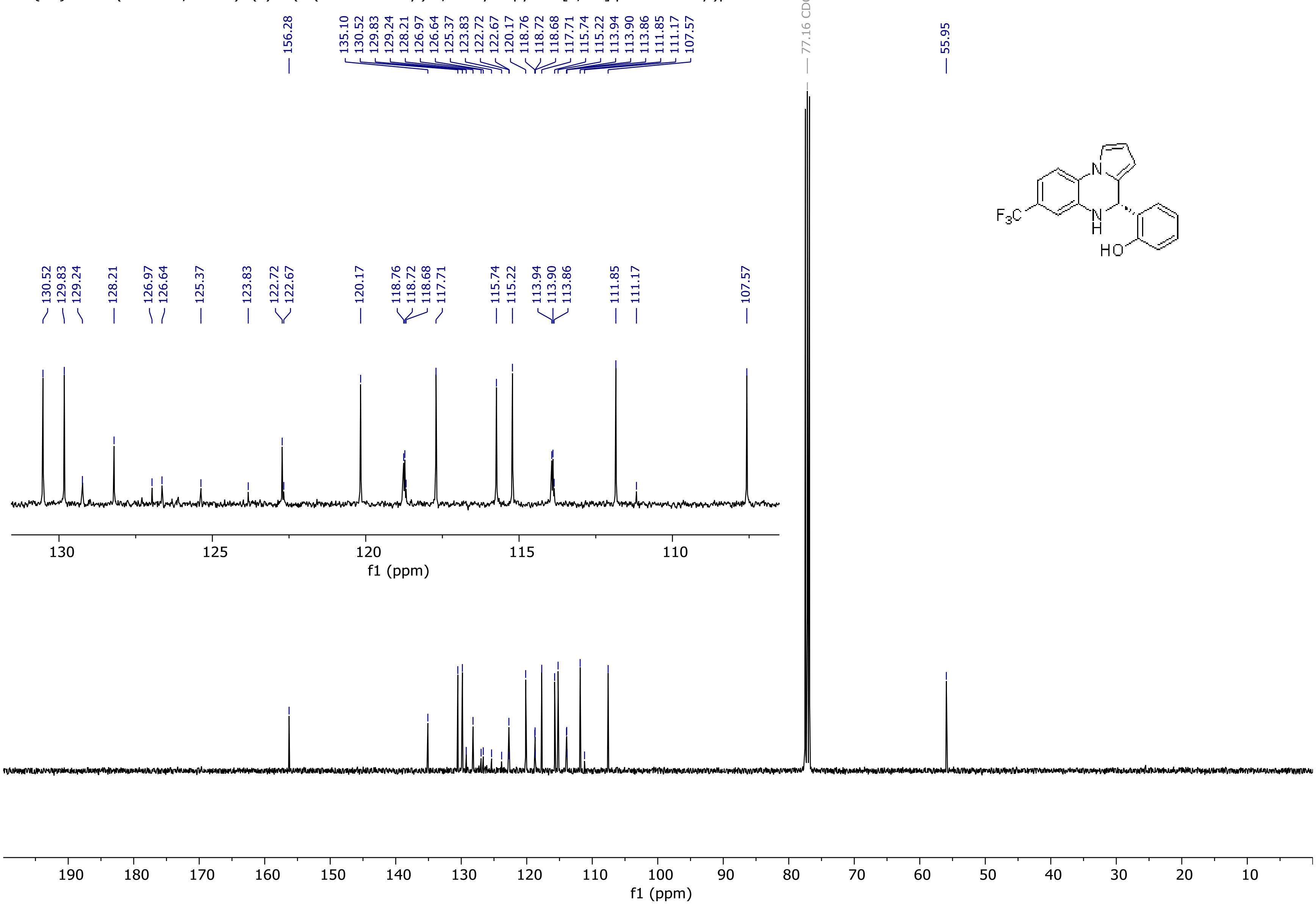

19F NMR (376 MHz, CDCl3) (S)-2-(7-(trifluoromethyl)-4,5-dihydropyrrolo[1,2-a]quinoxalin-4-yl)phenol 3dd.

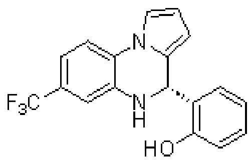

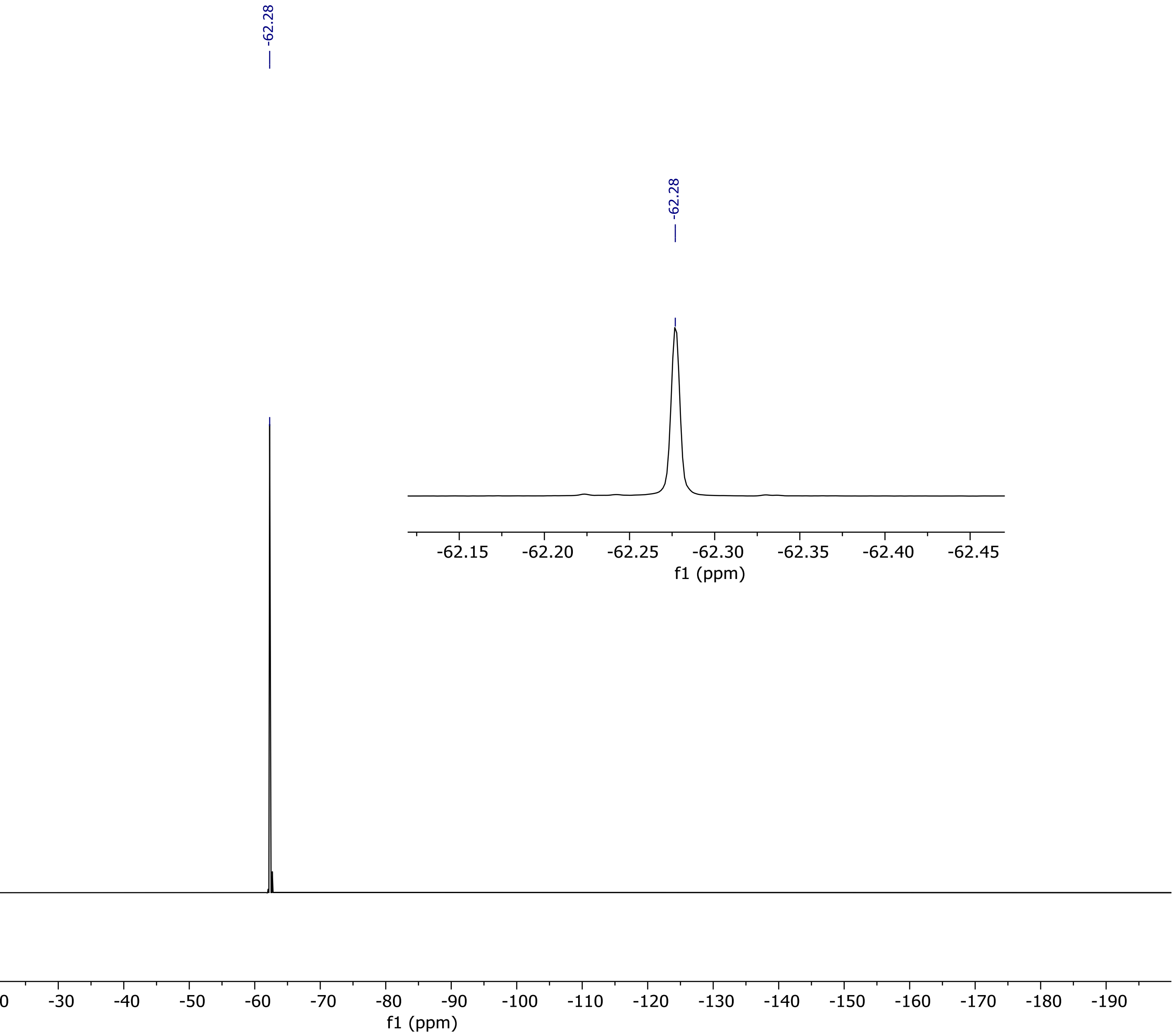

1H NMR (400 MHz, CDCl3): (S)-2-(5-acetyl-4,5-dihydropyrrolo[1,2-a]quinoxalin-4-yl)phenyl acetate 4.

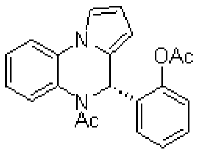

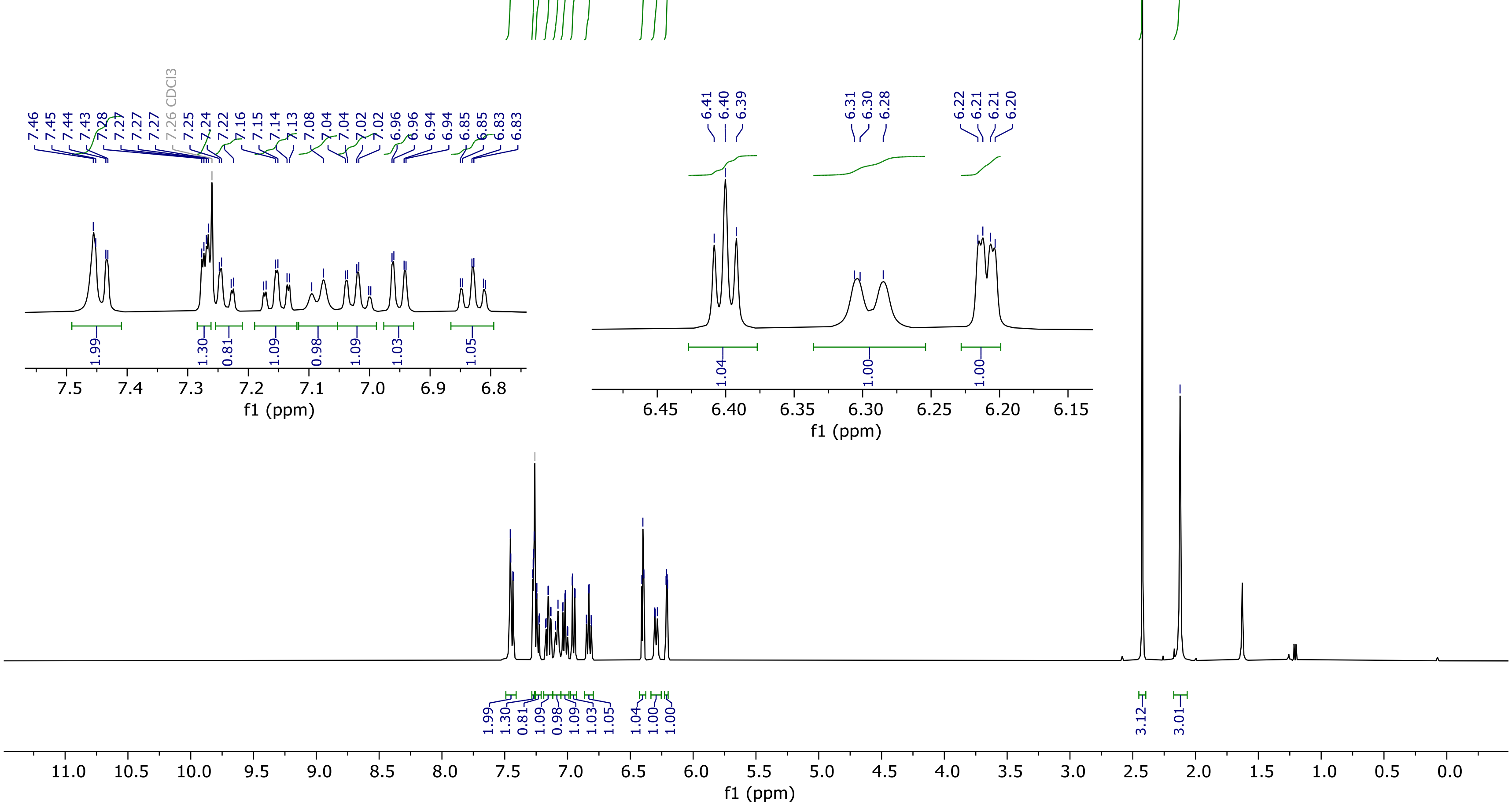

13C{1H} NMR: (101 MHz, CDCl3): (S)-2-(5-acetyl-4,5-dihydropyrrolo[1,2-a]quinoxalin-4-yl)phenyl acetate 4.

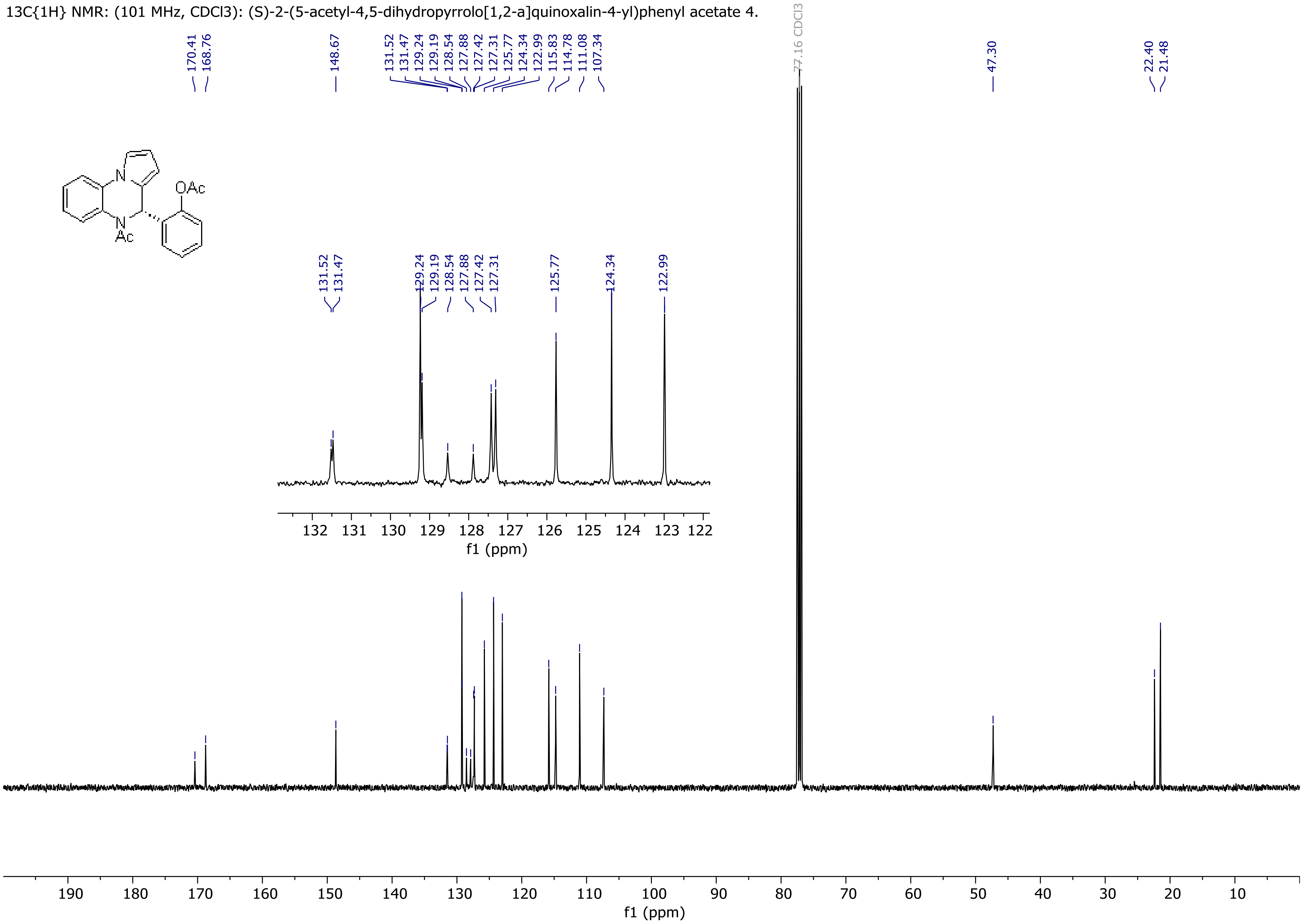

1H NMR (400 MHz, CDCl3): (S)-2-(5-acetyl-1-bromo-4,5-dihydropyrrolo[1,2-a]quinoxalin-4-yl)phenyl acetate 5.

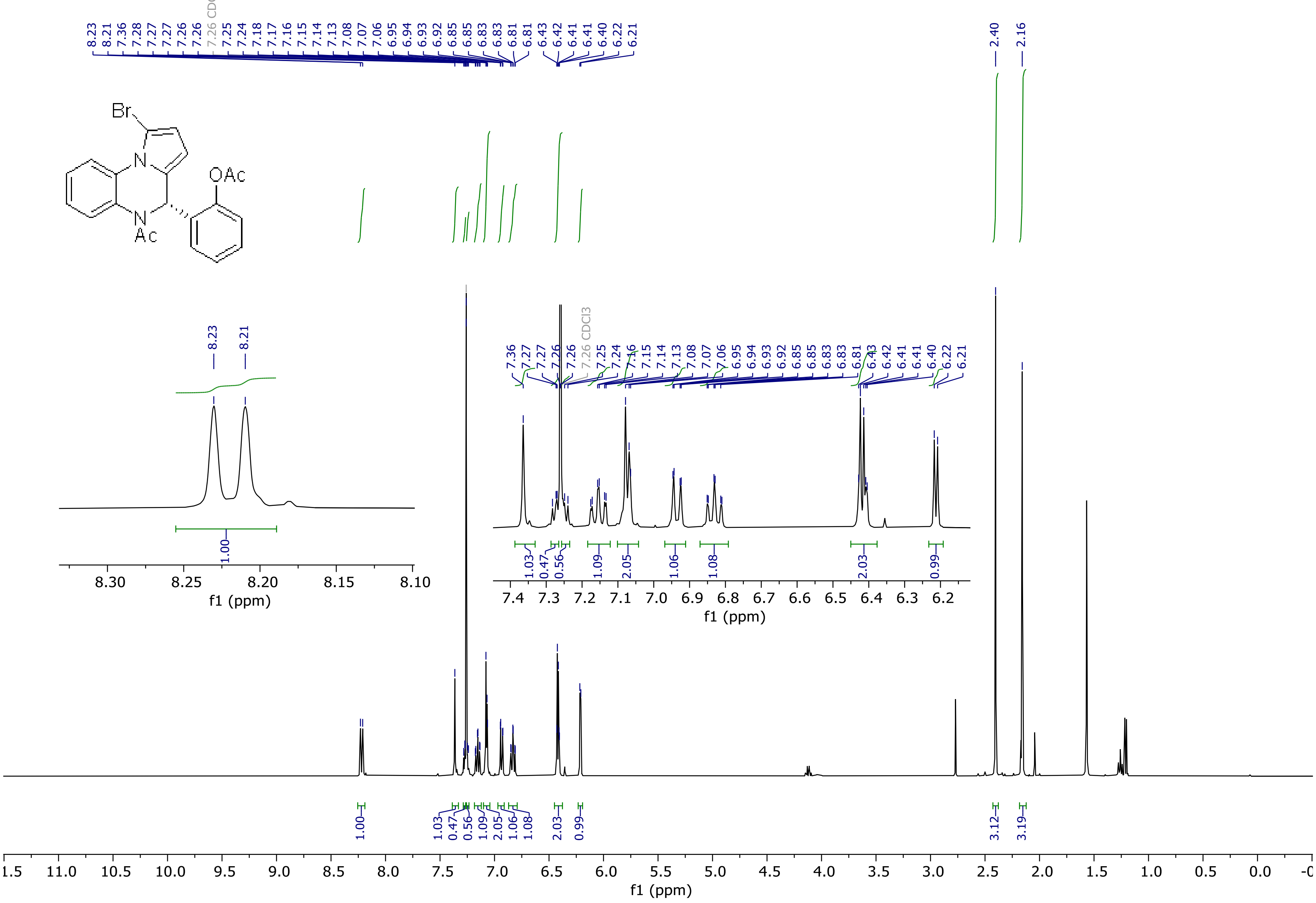

13C{1H} NMR: (101 MHz, CDCl3): (S)-2-(5-acetyl-1-bromo-4,5-dihydropyrrolo[1,2-a]quinoxalin-4-yl)phenyl acetate 5.

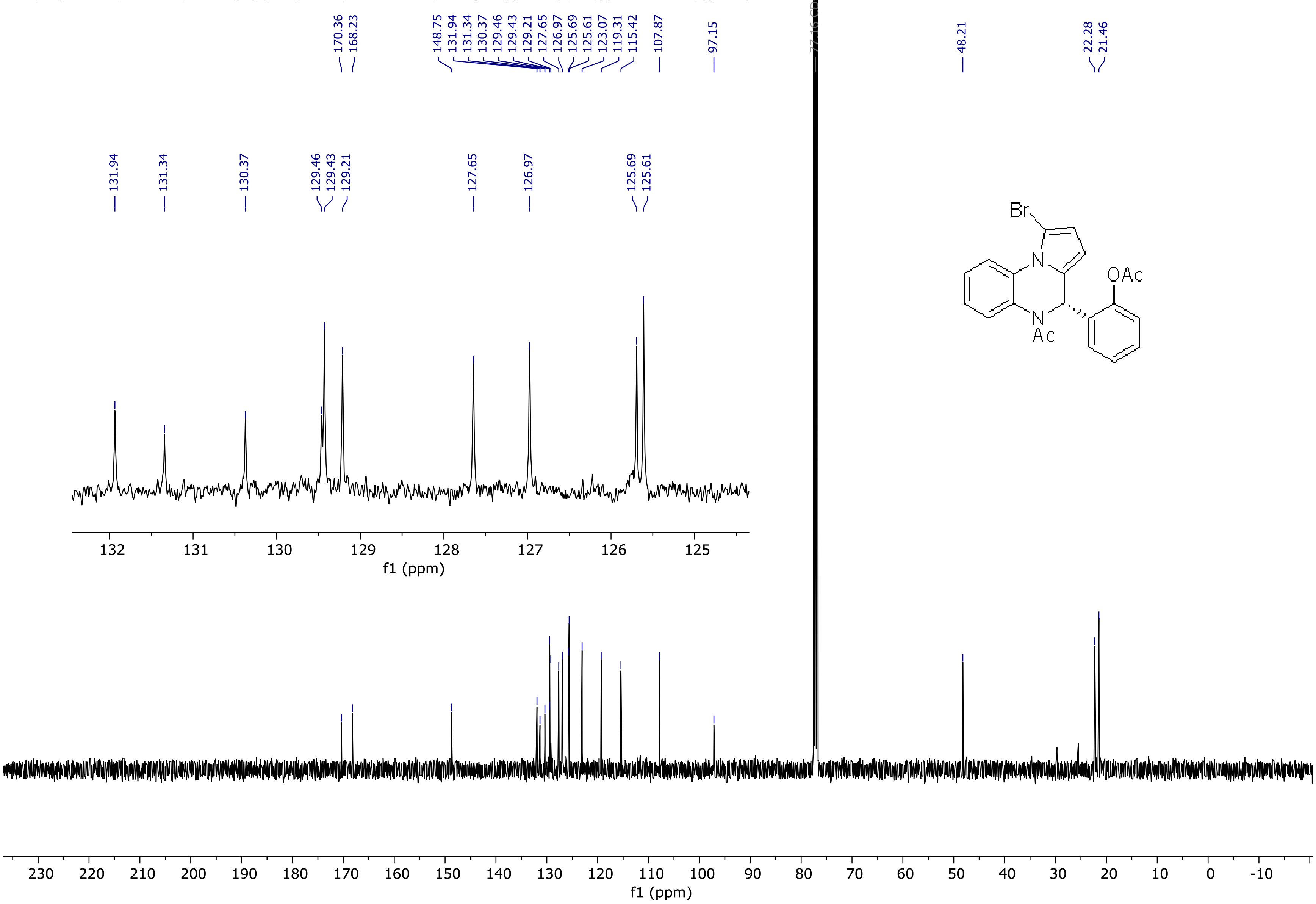

HPLC DATA:

(1R,2S)-2-phenylcyclohexan-1-ol (SM1):

RACEMATE:

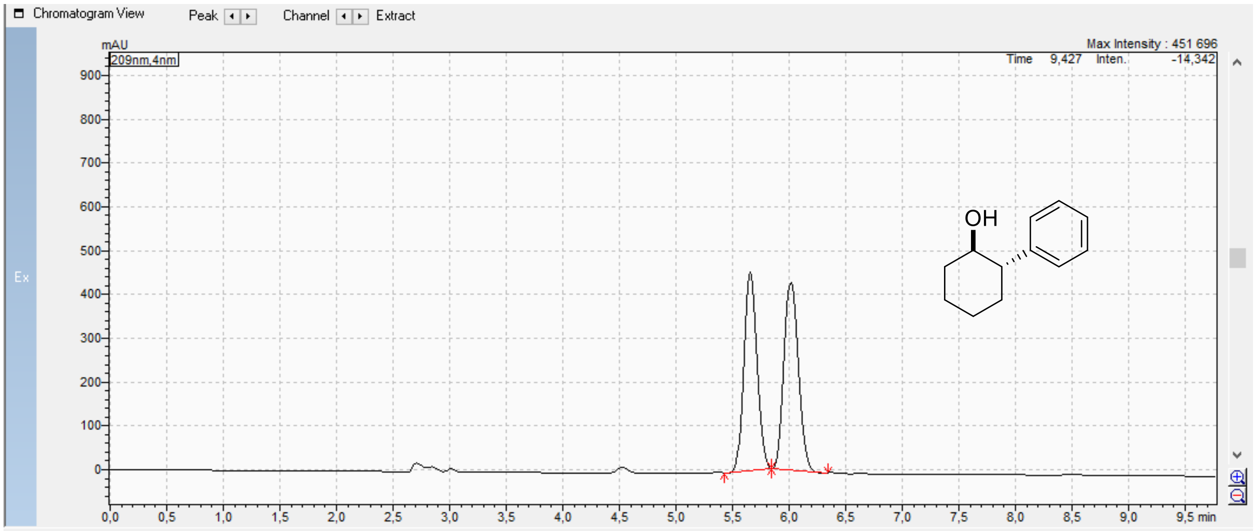

Results View - Peak Table

Peak Table | Compound | Group | Calibration Curve

Peak#	Ret. Time	Area	Height	Peak Start	Peak End	Mark	Conc.	Unit	Area%
1	5,653	3683464	454018	5,429	5,845	M	50,074		50,074
2	6,014	3672602	429553	5,845	6,347	M	49,926		49,926
Total		7356066	883571				100,000		100,000

CHIRAL: *E.r.*: 99:1

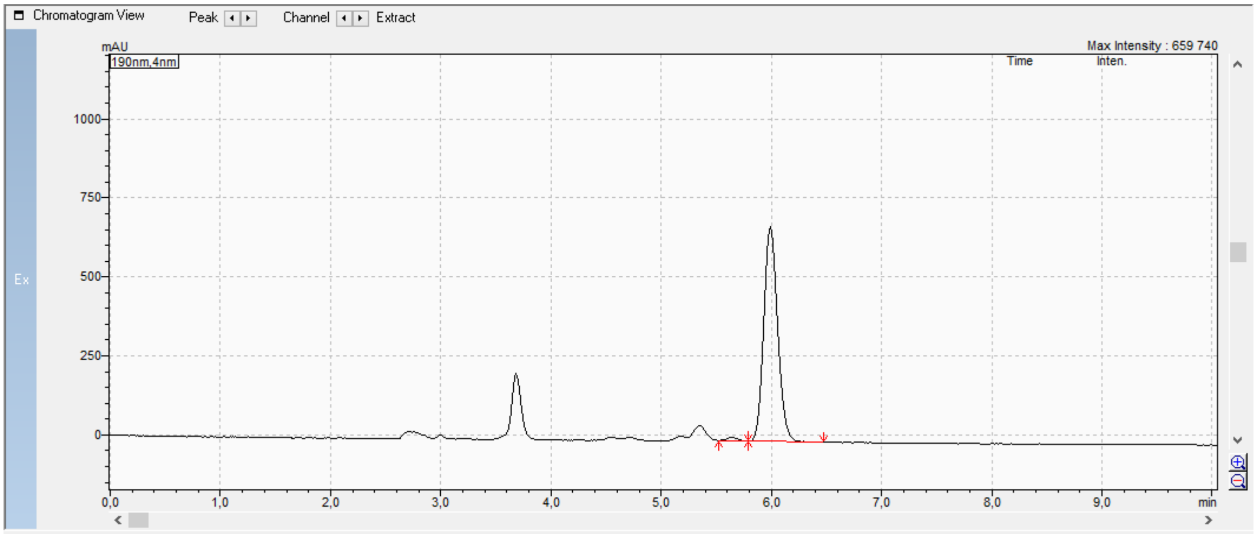

Results View - Peak Table

Peak Table | Compound | Group | Calibration Curve

Peak#	Ret. Time	Area	Height	Peak Start	Peak End	Mark	Conc.	Unit	Area%
1	5,653	71544	10216	5,525	5,792	M	1,195		1,195
2	5,994	5916711	680202	5,792	6,475	M	98,805		98,805
Total		5988255	690419				100,000		100,000

(1R,2S)-2-(4-methoxyphenyl)cyclohexan-1-ol (SM2):

RACEMATE:

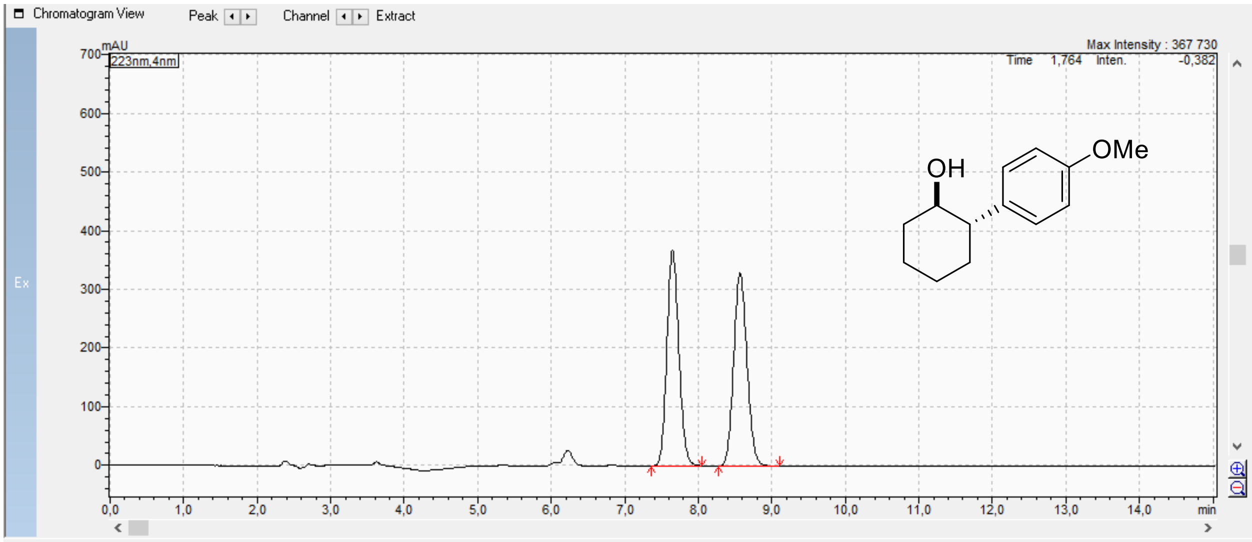

Results View - Peak Table

Peak Table | Compound | Group | Calibration Curve

Peak#	Ret. Time	Area	Height	Peak Start	Peak End	Mark	Conc.	Unit	Area%
1	7,650	4148149	368600	7,360	8,053	M	49,975		49,975
2	8,566	4152318	330091	8,277	9,109	M	50,025		50,025
Total		8300467	698691				100,000		100,000

CHIRAL: *E.r.*: 99:1

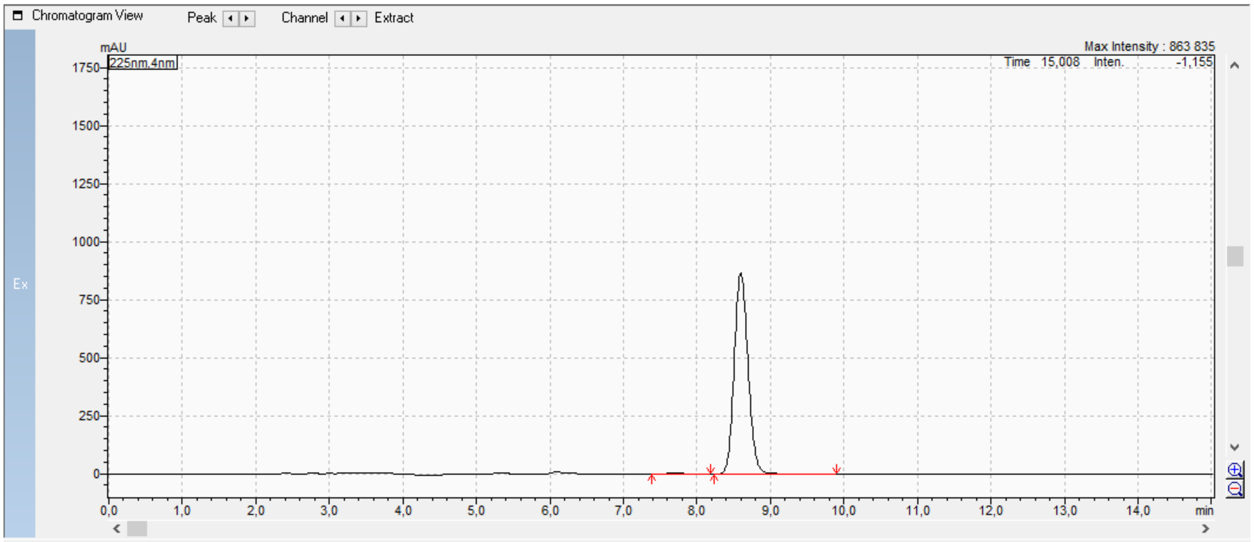

Results View - Peak Table

Peak Table | Compound | Group | Calibration Curve

Peak#	Ret. Time	Area	Height	Peak Start	Peak End	Mark	Conc.	Unit	Area%
1	7,693	38463	2661	7,381	8,181	M	0,341		0,341
2	8,597	11248986	864678	8,235	9,899	M	99,659		99,659
Total		11287450	867339				100,000		100,000

(1R,2S)-2-(p-tolyl)cyclohexan-1-ol (SM3):

RACEMATE:

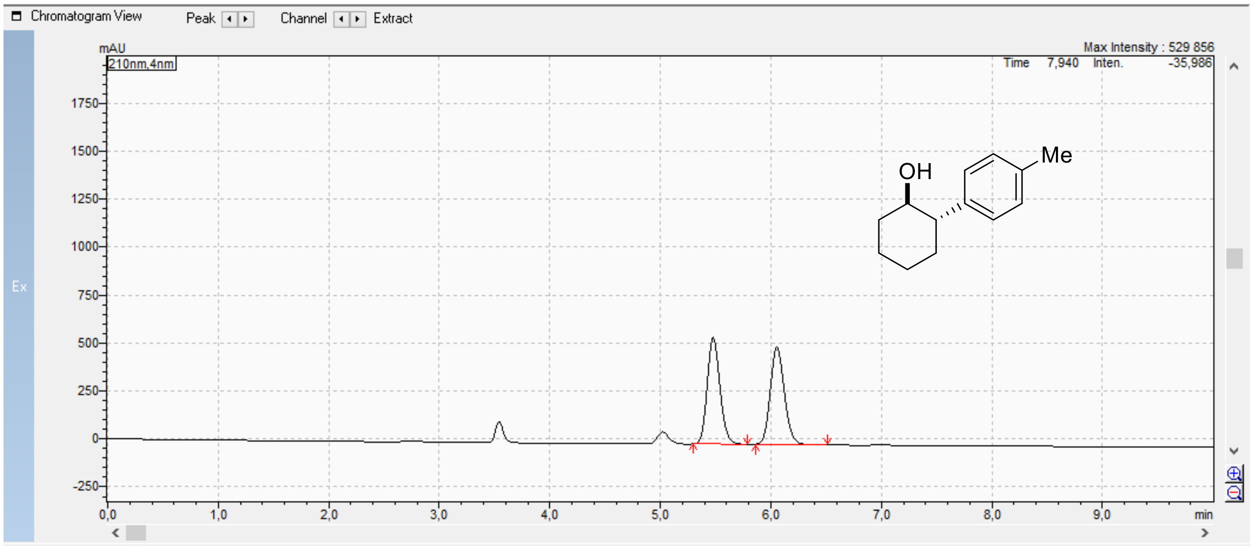

Results View - Peak Table

Peak Table | Compound | Group | Calibration Curve

Peak#	Ret. Time	Area	Height	Peak Start	Peak End	Mark	Conc.	Unit	Area%
1	5,483	4457638	556267	5,301	5,792	M	49,877		49,877
2	6,060	4479610	506413	5,867	6,517	M	50,123		50,123
Total		8937247	1062680				100,000		100,000

CHIRAL: *E.r.*: 99:1

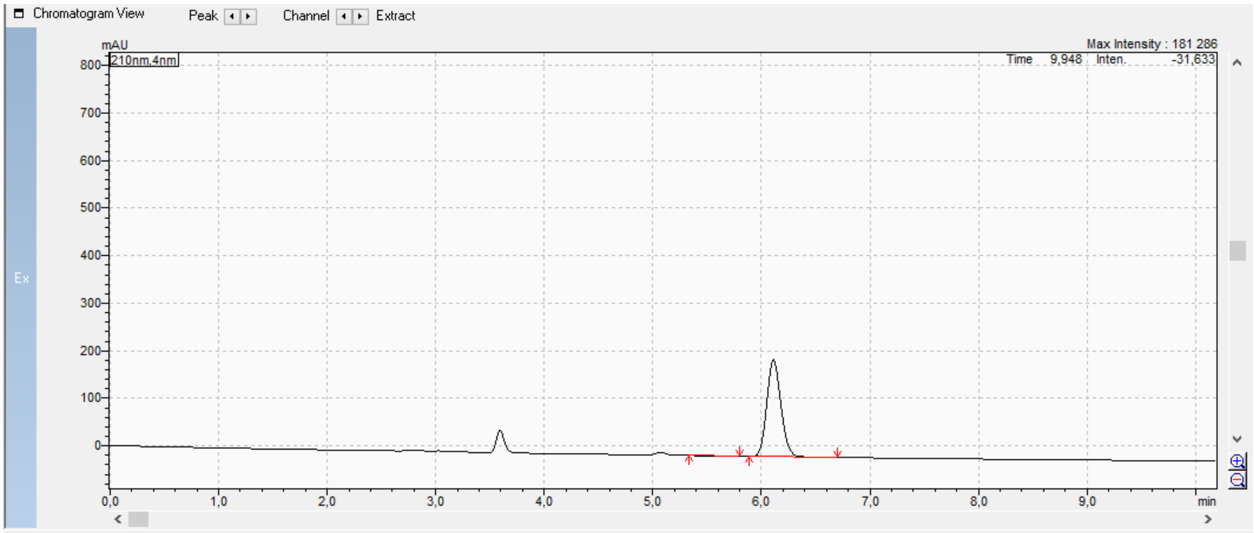

Results View - Peak Table

Peak Table | Compound | Group | Calibration Curve

Peak#	Ret. Time	Area	Height	Peak Start	Peak End	Mark	Conc.	Unit	Area%
1	5,371	5036	105	5,333	5,803	M	0,277		0,277
2	6,109	1811164	204103	5,888	6,699	M	99,723		99,723
Total		1816200	204208				100,000		100,000

(S)-4-isobutyl-4,5-dihydropyrrolo[1,2-a]quinoxaline (3b).

RACEMATE:

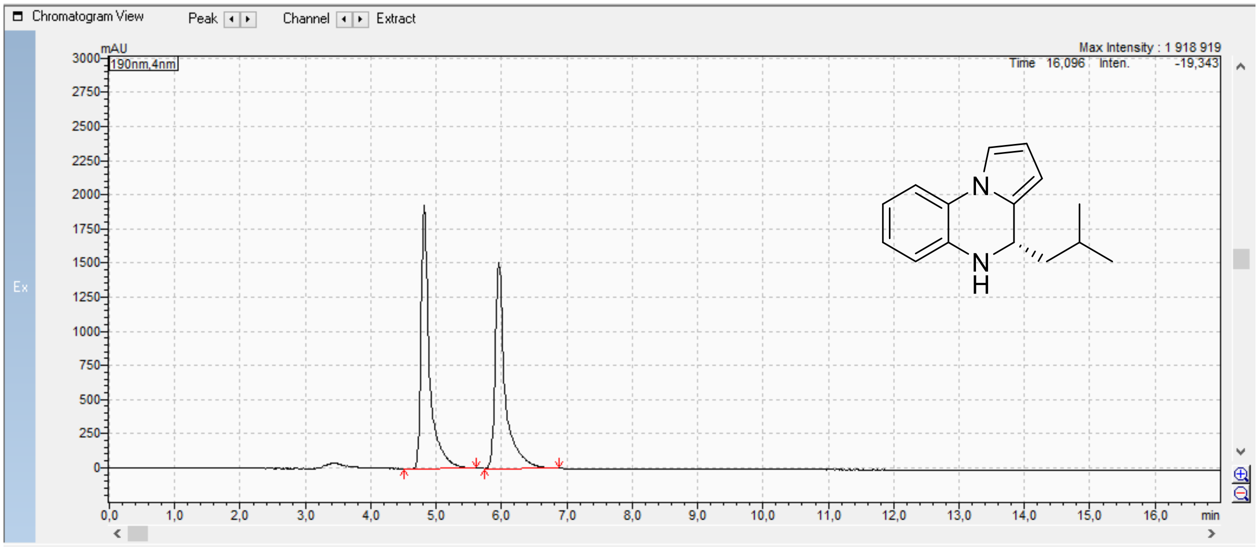

Results View - Peak Table

Peak Table | Compound | Group | Calibration Curve

Peak#	Ret. Time	Area	Height	Peak Start	Peak End	Mark	Conc.	Unit	Area%
1	4,819	17536074	1925474	4,512	5,611	M	52,160		52,160
2	5,959	16083403	1509679	5,739	6,880	M	47,840		47,840
Total		33619477	3435153				100,000		100,000

CHIRAL: *E.r.*: 85:15

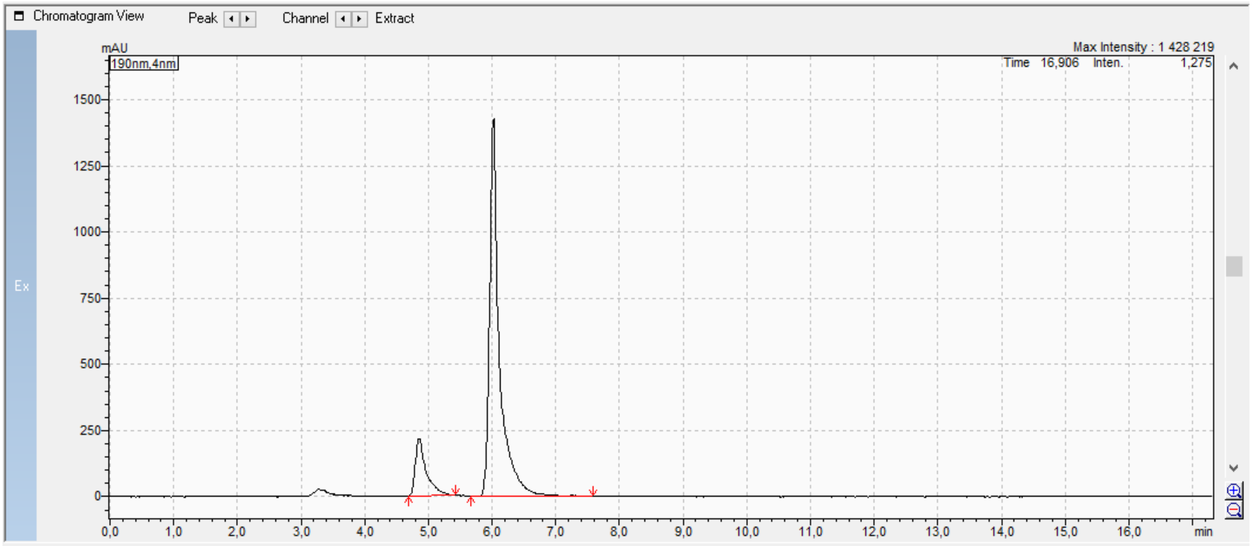

Results View - Peak Table

Peak Table | Compound | Group | Calibration Curve

Peak#	Ret. Time	Area	Height	Peak Start	Peak End	Mark	Conc.	Unit	Area%
1	4,855	2560835	216681	4,693	5,429	M	14,515		14,515
2	6,022	15081637	1422203	5,664	7,584	M	85,485		85,485
Total		17642472	1638883				100,000		100,000

(S)-2-(4,5-dihydropyrrolo[1,2-a]quinoxalin-4-yl)phenol (3c).

RACEMATE:

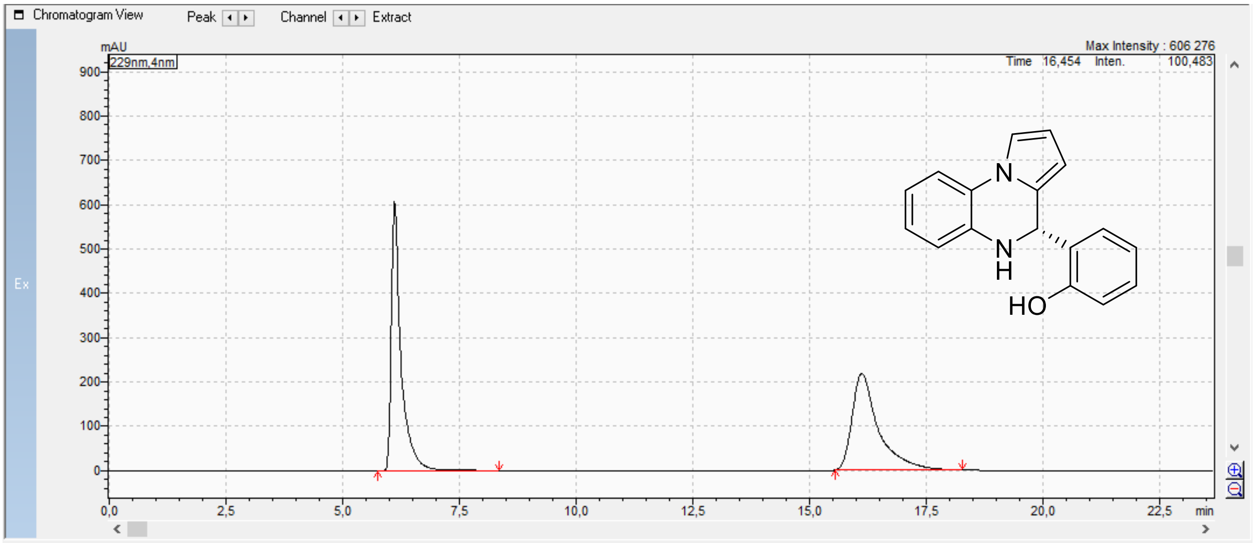

Results View - Peak Table

Peak Table | Compound | Group | Calibration Curve

Peak#	Ret. Time	Area	Height	Peak Start	Peak End	Mark	Conc.	Unit	Area%
1	6,114	8714329	605999	5,760	8,341	M	50,531		50,531
2	16,123	8531031	217323	15,552	18,272	M	49,469		49,469
Total		17245360	823323				100,000		100,000

CHIRAL: *E.r.*: 99:1

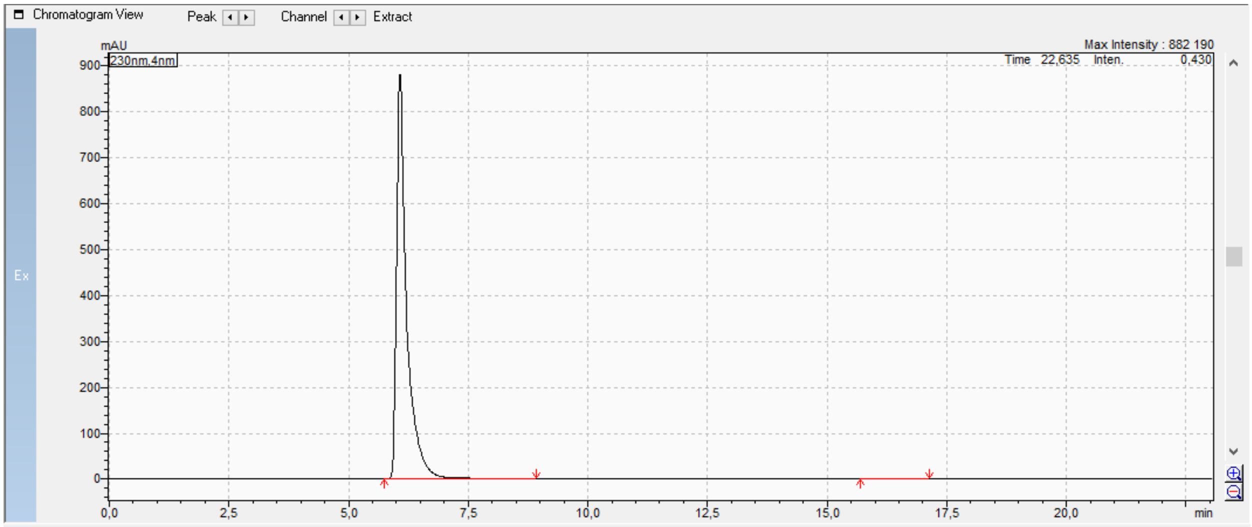

Results View - Peak Table

Peak Table | Compound | Group | Calibration Curve

Peak#	Ret. Time	Area	Height	Peak Start	Peak End	Mark	Conc.	Unit	Area%
1	6,074	12969899	881770	5,749	8,917	M	99,912		99,912
2	16,232	11465	350	15,691	17,131	M	0,088		0,088
Total		12981364	882120				100,000		100,000

(S)-2-(4,5-dihydropyrrolo[1,2-a]quinoxalin-4-yl)-4-methylphenol (3d).

RACEMATE:

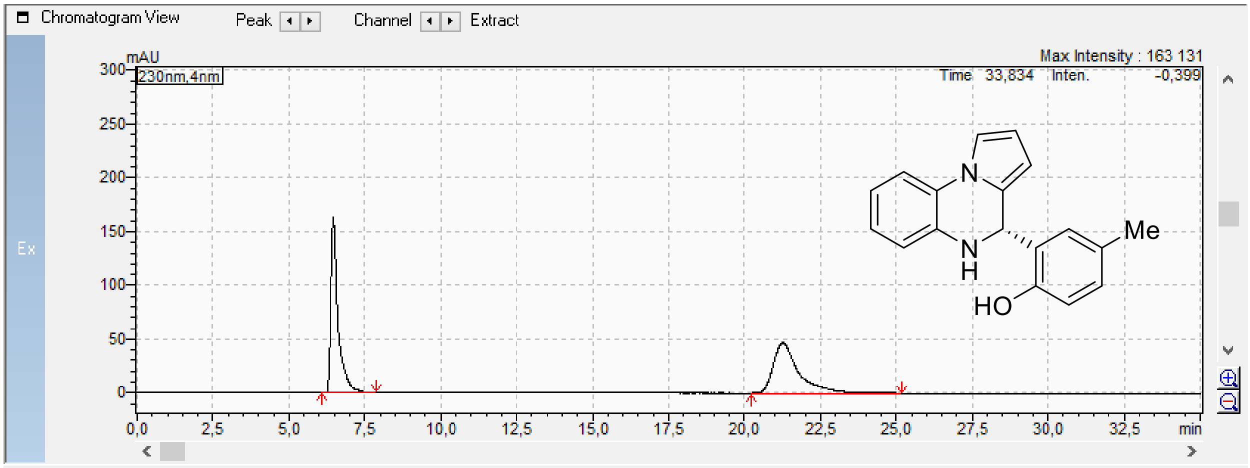

Results View - Peak Table

Peak Table | Compound | Group | Calibration Curve

Peak#	Ret. Time	Area	Height	Peak Start	Peak End	Mark	Conc.	Unit	Area%
1	6,466	2726322	163132	6,080	7,872	M	50,260		50,260
2	21,262	2698105	46743	20,224	25,184	M	49,740		49,740
Total		5424427	209875				100,000		100,000

CHIRAL: *E.r.*: 82:18

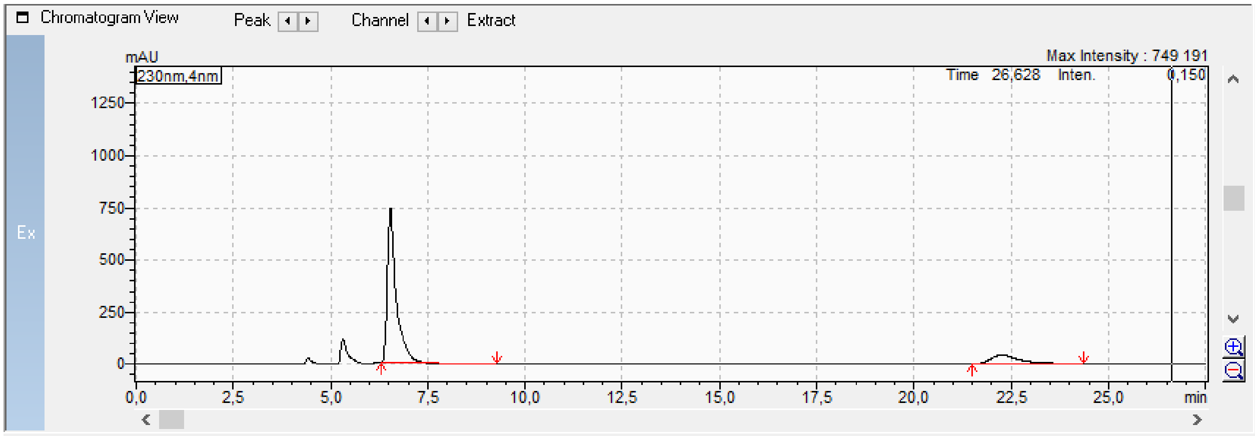

Results View - Peak Table

Peak Table | Compound | Group | Calibration Curve

Peak#	Ret. Time	Area	Height	Peak Start	Peak End	Mark	Conc.	Unit	Area%
1	6,540	11668408	742642	6,304	9,280	M	82,522		82,522
2	22,270	2471294	44463	21,493	24,363	M	17,478		17,478
Total		14139702	787105				100,000		100,000

(*S*)-2-(4,5-dihydropyrrolo[1,2-a]quinoxalin-4-yl)-5-methylphenol (3e):

RACEMATE:

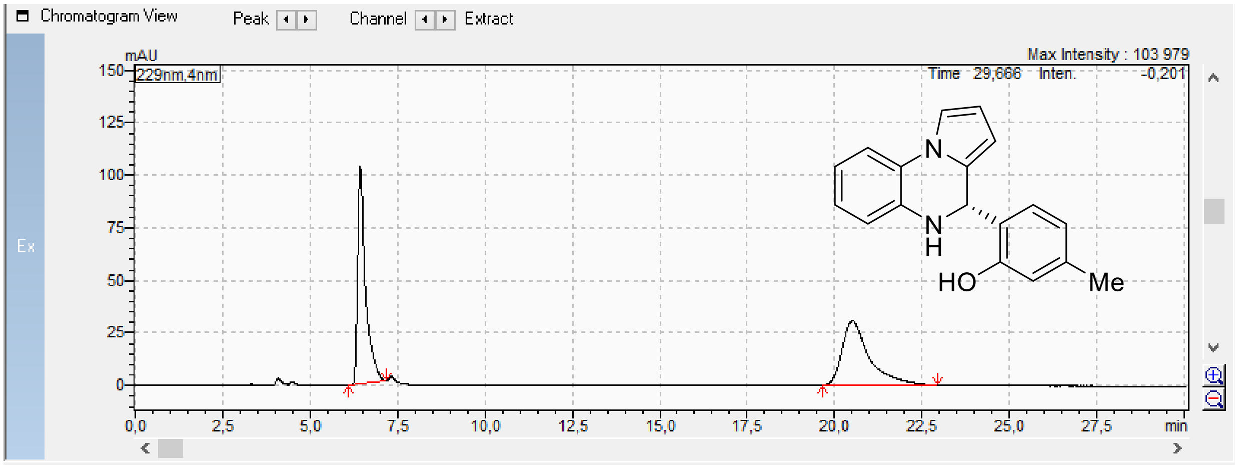

Results View - Peak Table

Peak Table | Compound | Group | Calibration Curve

Peak#	Ret. Time	Area	Height	Peak Start	Peak End	Mark	Conc.	Unit	Area%
1	6,443	1642307	103209	6,101	7,189	M	49,401		49,401
2	20,507	1682152	30777	19,648	22,944	M	50,599		50,599
Total		3324459	133986				100,000		100,000

CHIRAL: *E.r.*: 93:7

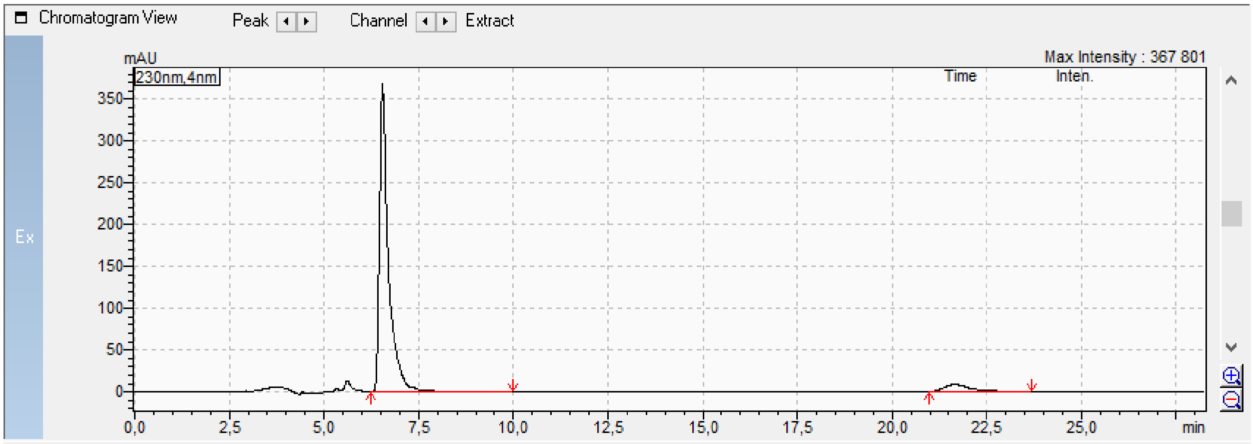

Results View - Peak Table

Peak Table | Compound | Group | Calibration Curve

Peak#	Ret. Time	Area	Height	Peak Start	Peak End	Mark	Conc.	Unit	Area%
1	6,547	5952737	367429	6,229	9,984	M	92,866		92,866
2	21,646	457298	8720	20,981	23,691	M	7,134		7,134
Total		6410036	376149				100,000		100,000

(S)-2-(4,5-dihydropyrrolo[1,2-a]quinoxalin-4-yl)-4-methoxyphenol (3g):

RACEMATE:

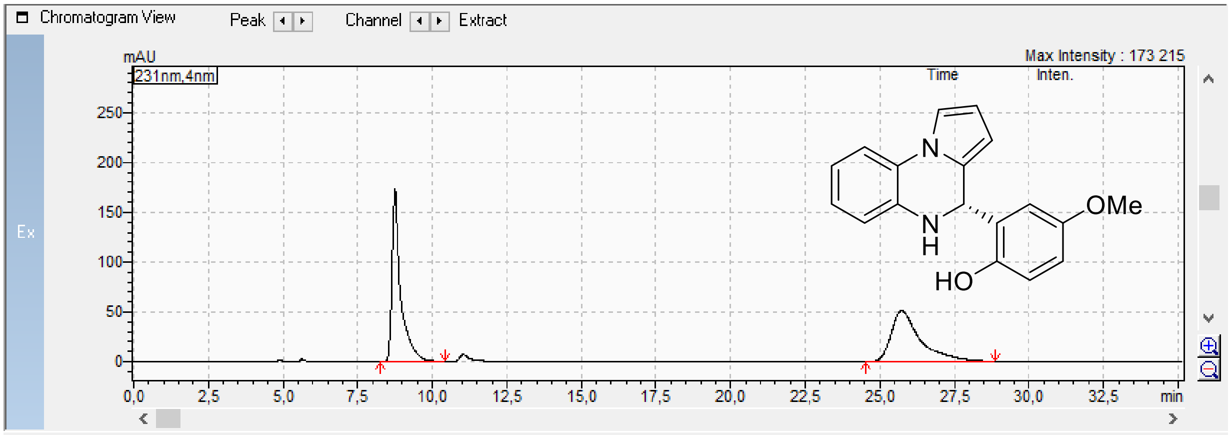

Results View - Peak Table

Peak Table | Compound | Group | Calibration Curve

Peak#	Ret. Time	Area	Height	Peak Start	Peak End	Mark	Conc.	Unit	Area%
1	8,750	3523199	173093	8,256	10,432	M	50,383		50,383
2	25,740	3469683	51103	24,523	28,875	M	49,617		49,617
Total		6992882	224197				100,000		100,000

CHIRAL: *E.r.*: 95:5

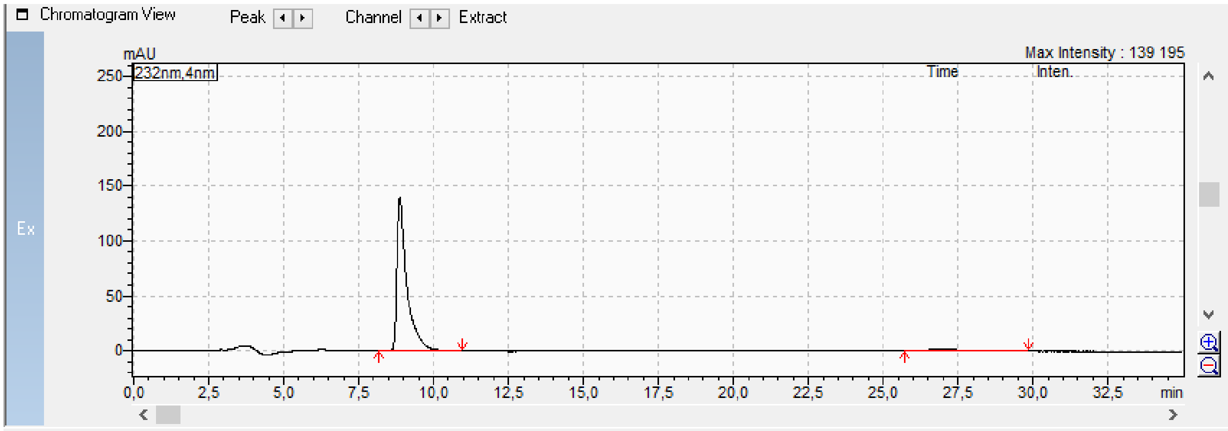

Results View - Peak Table

Peak Table | Compound | Group | Calibration Curve

Peak#	Ret. Time	Area	Height	Peak Start	Peak End	Mark	Conc.	Unit	Area%
1	8,875	3214887	139198	8,171	10,955	M	95,017		95,017
2	26,895	168604	2379	25,707	29,835	M	4,983		4,983
Total		3383491	141577				100,000		100,000

(S)-2-(4,5-dihydropyrrolo[1,2-a]quinoxalin-4-yl)-4-methoxyphenol (3h).

RACEMATE:

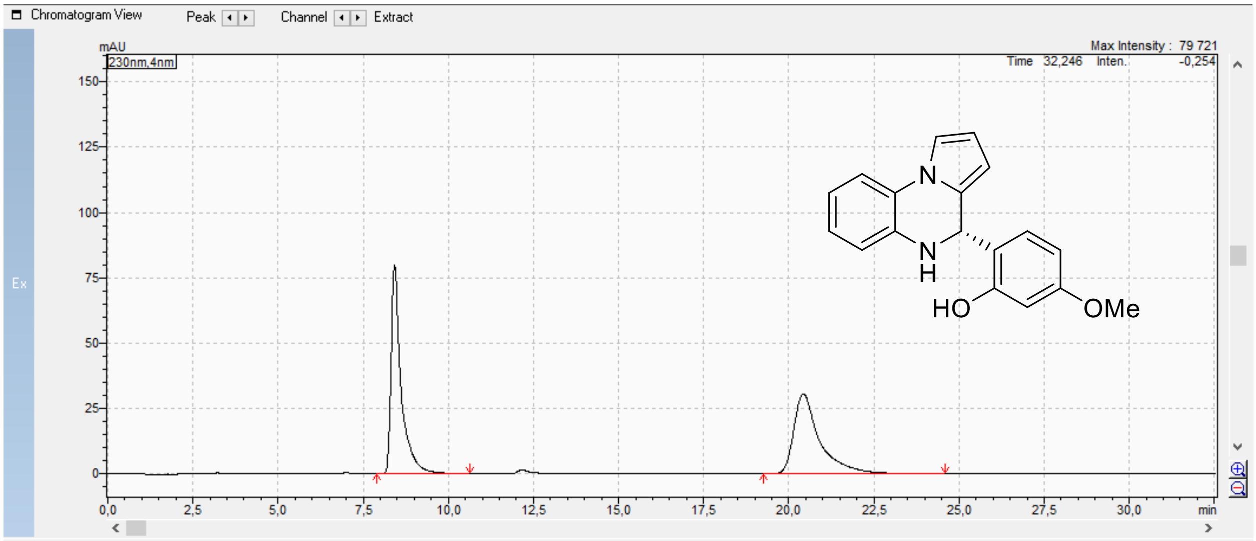

Results View - Peak Table

Peak Table | Compound | Group | Calibration Curve

Peak#	Ret. Time	Area	Height	Peak Start	Peak End	Mark	Conc.	Unit	Area%
1	8,423	1639132	79864	7,904	10,645	M	50,086		50,086
2	20,425	1633503	30745	19,253	24,608	M	49,914		49,914
Total		3272635	110609				100,000		100,000

CHIRAL: *E.r.*: 63:37

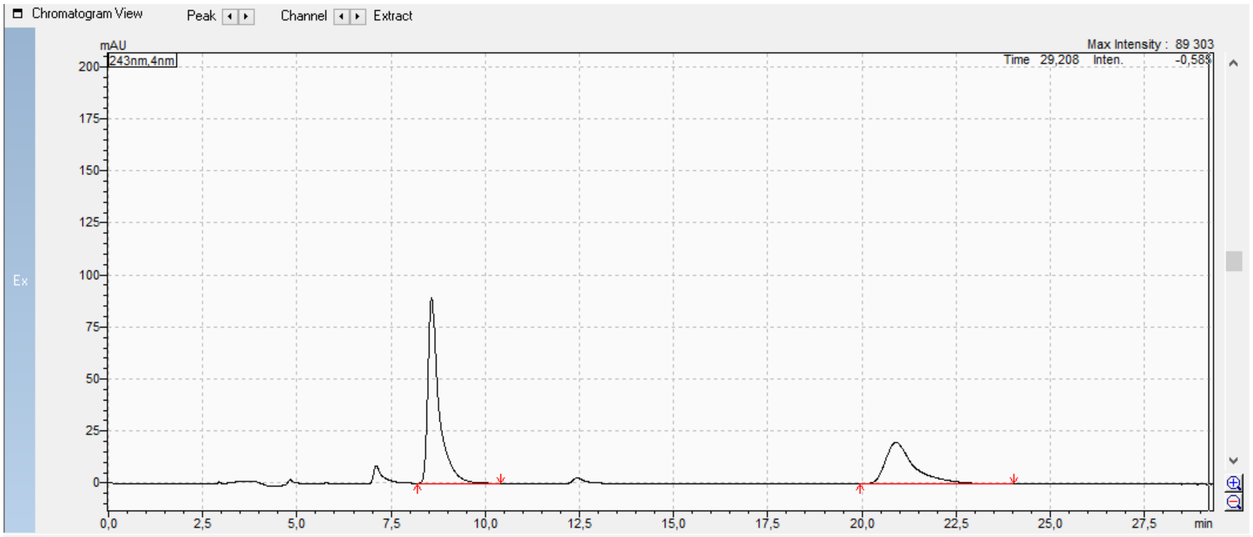

Results View - Peak Table

Peak Table | Compound | Group | Calibration Curve

Peak#	Ret. Time	Area	Height	Peak Start	Peak End	Mark	Conc.	Unit	Area%
1	8,580	1822347	89387	8,203	10,411	M	62,749		62,749
2	20,901	1081831	20151	19,947	24,032	M	37,251		37,251
Total		2904178	109537				100,000		100,000

(S)-2-(4,5-dihydropyrrolo[1,2-a]quinoxalin-4-yl)-4-methoxyphenol (3i).

RACEMATE:

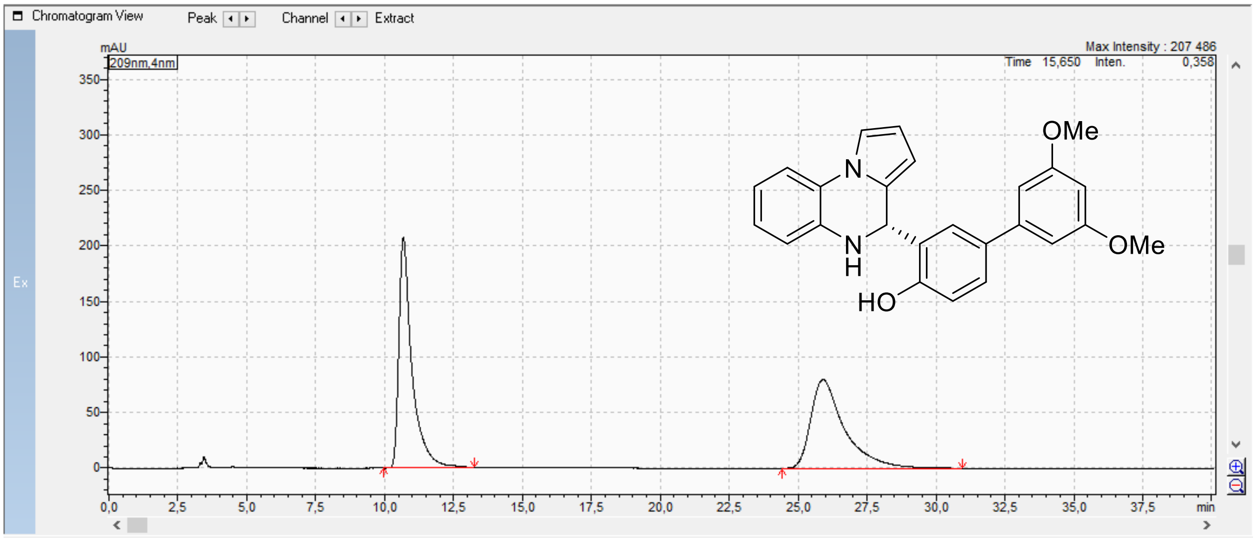

Results View - Peak Table

Peak Table | Compound | Group | Calibration Curve

Peak#	Ret. Time	Area	Height	Peak Start	Peak End	Mark	Conc.	Unit	Area%
1	10,685	6838418	207376	9,952	13,259	M	50,282		50,282
2	25,900	6761719	79920	24,405	30,965	M	49,718		49,718
Total		13600137	287296				100,000		100,000

CHIRAL: *E.r.*: 66:34

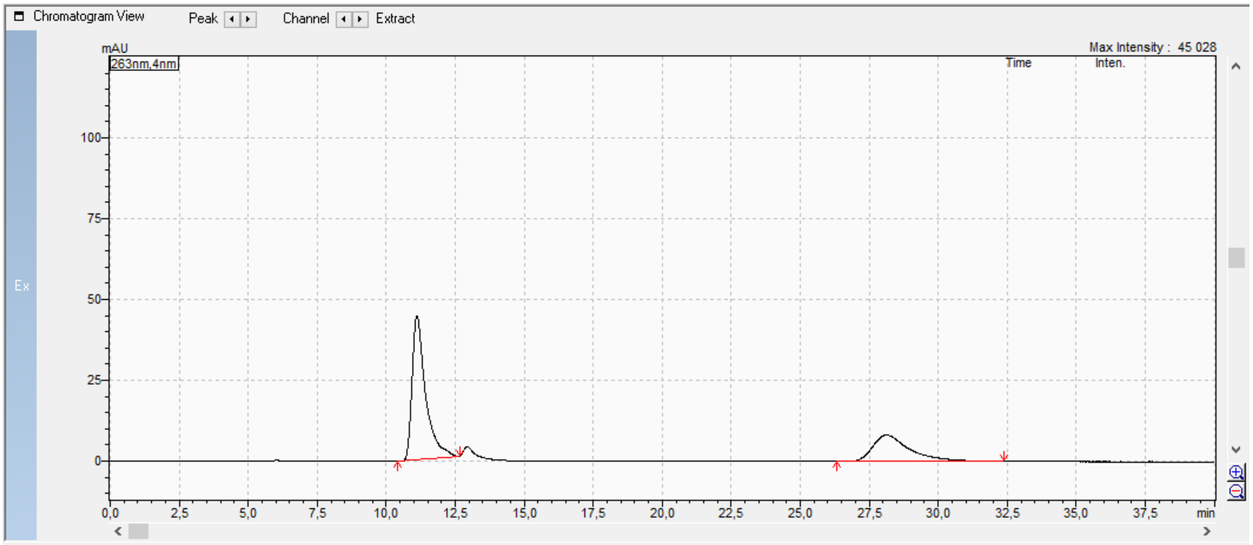

Results View - Peak Table

Peak Table | Compound | Group | Calibration Curve

Peak#	Ret. Time	Area	Height	Peak Start	Peak End	Mark	Conc.	Unit	Area%
1	11,109	1512994	44580	10,400	12,651	M	66,428		66,428
2	28,147	764642	8154	26,304	32,395	M	33,572		33,572
Total		2277635	52734				100,000		100,000

(S)-2-(4,5-dihydropyrrolo[1,2-a]quinoxalin-4-yl)-4-nitrophenol (3j).

RACEMATE:

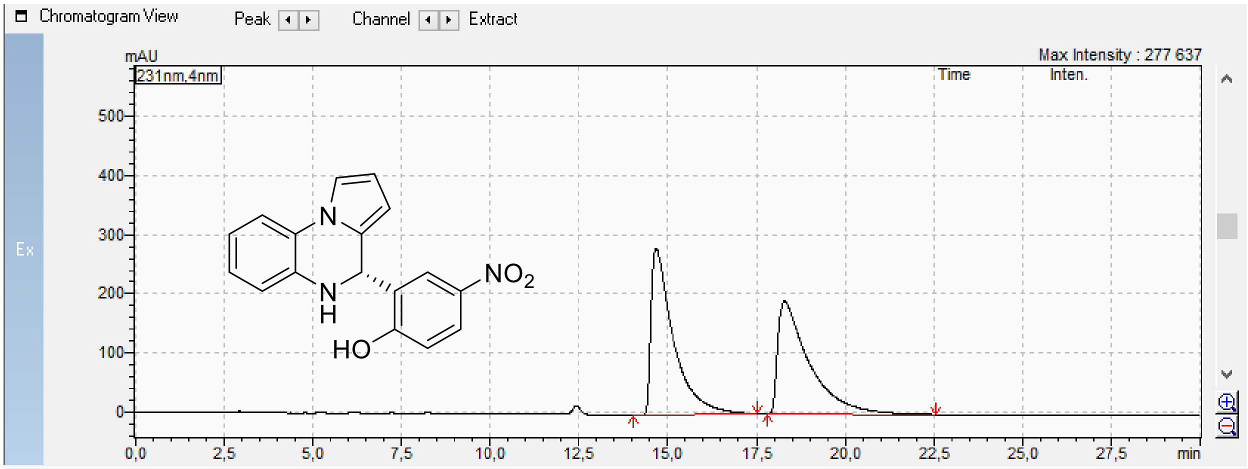

Results View - Peak Table

Peak Table | Compound | Group | Calibration Curve

Peak#	Ret. Time	Area	Height	Peak Start	Peak End	Mark	Conc.	Unit	Area%
1	14,677	12383735	282671	14,027	17,525	M	51,241		51,241
2	18,293	11783981	191100	17,813	22,560	M	48,759		48,759
Total		24167716	473772				100,000		100,000

CHIRAL: *E.r.*: 51:49

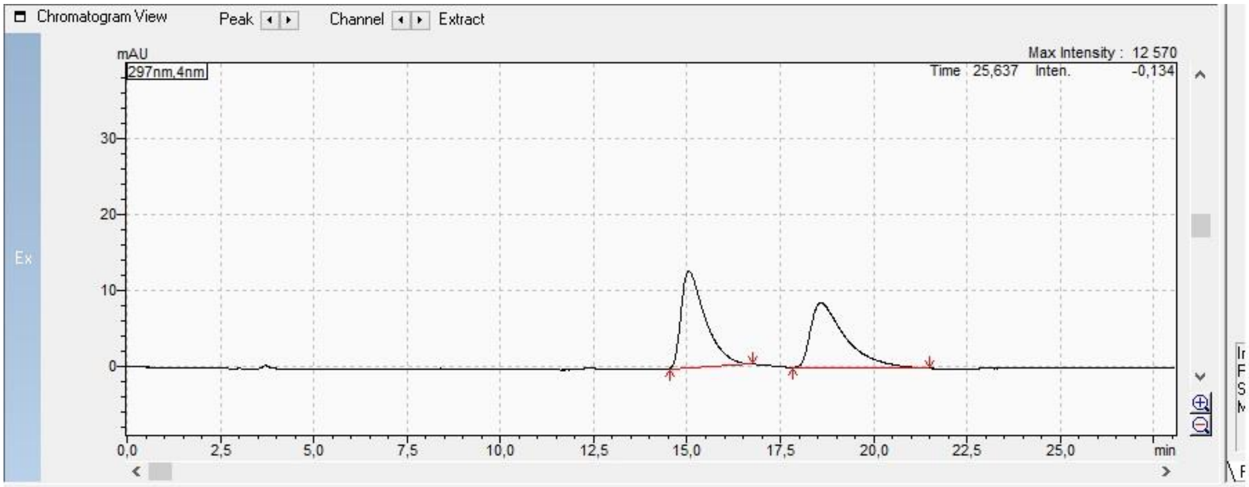

Results View - Peak Table

Peak Table | Compound | Group | Calibration Curve

Peak#	Ret. Time	Area	Height	Peak Start	Peak End	Mark	Conc.	Unit	Area%
1	15,051	574780	12717	14,539	16,757	M	50,853		50,853
2	18,574	555490	8550	17,845	21,504	M	49,147		49,147
Total		1130270	21267				100,000		100,000

(S)-3-(4,5-dihydropyrrolo[1,2-a]quinoxalin-4-yl)-4-hydroxybenzonitrile (3k).

RACEMATE:

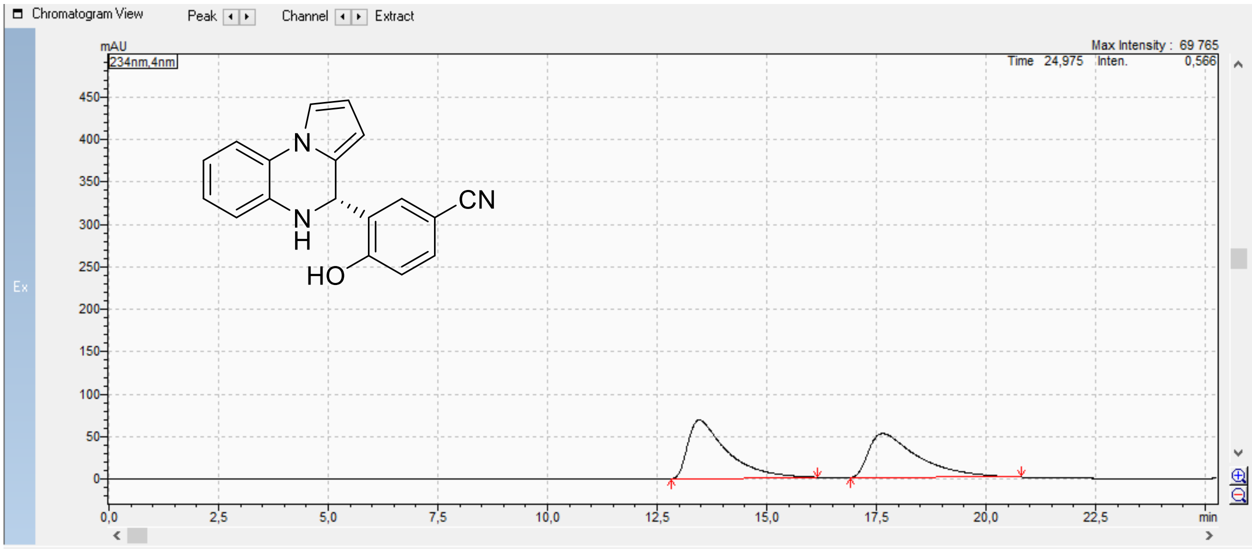

Results View - Peak Table

Peak Table | Compound | Group | Calibration Curve

Peak#	Ret. Time	Area	Height	Peak Start	Peak End	Mark	Conc.	Unit	Area%
1	13,462	4261472	68988	12,821	16,160	M	51,139		51,139
2	17,644	4071636	51812	16,907	20,811	M	48,861		48,861
Total		8333108	120800				100,000		100,000

CHIRAL: *E.r.*: 52:48

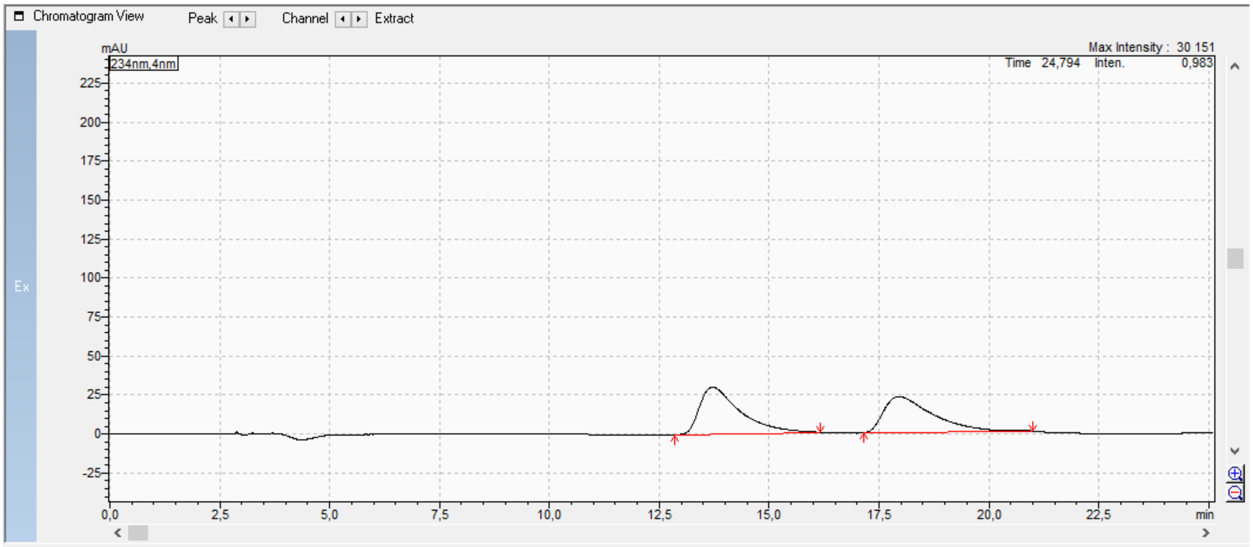

Results View - Peak Table

Peak Table | Compound | Group | Calibration Curve

Peak#	Ret. Time	Area	Height	Peak Start	Peak End	Mark	Conc.	Unit	Area%
1	13,712	1924221	30096	12,853	16,160	M	51,645		51,645
2	17,957	1801666	23105	17,152	21,003	M	48,355		48,355
Total		3725887	53201				100,000		100,000

Methyl (S)-3-(4,5-dihydropyrrolo[1,2-a]quinoxalin-4-yl)-4-hydroxybenzoate (3l).

RACEMATE:

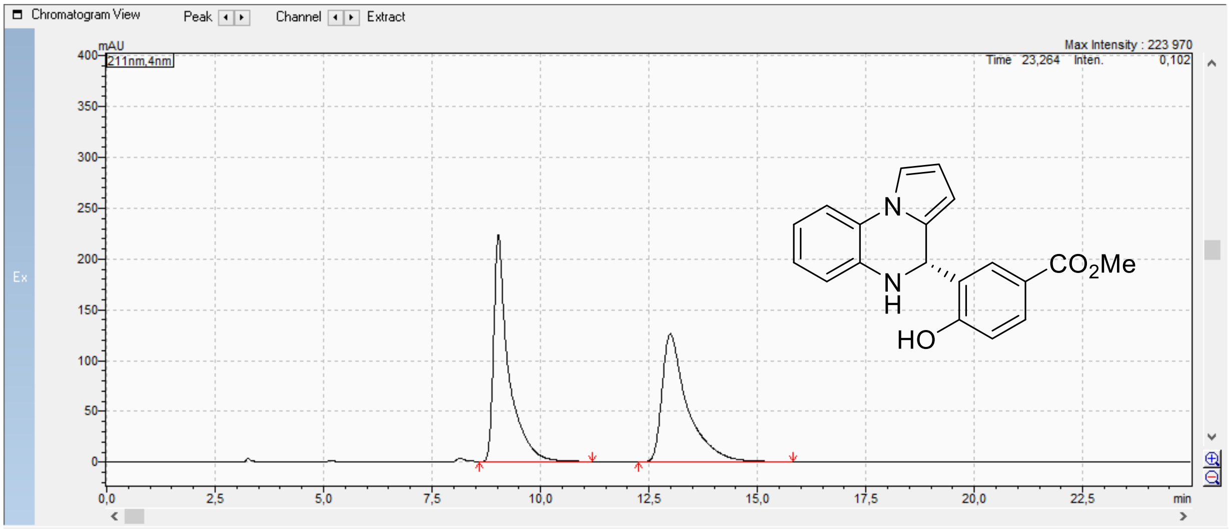

Results View - Peak Table

Peak Table | Compound | Group | Calibration Curve

Peak#	Ret. Time	Area	Height	Peak Start	Peak End	Mark	Conc.	Unit	Area%
1	9,028	5303003	223358	8,587	11,189	M	50,971		50,971
2	12,994	5101059	125993	12,256	15,819	M	49,029		49,029
Total		10404063	349351				100,000		100,000

CHIRAL: *E.r.*: 76:24

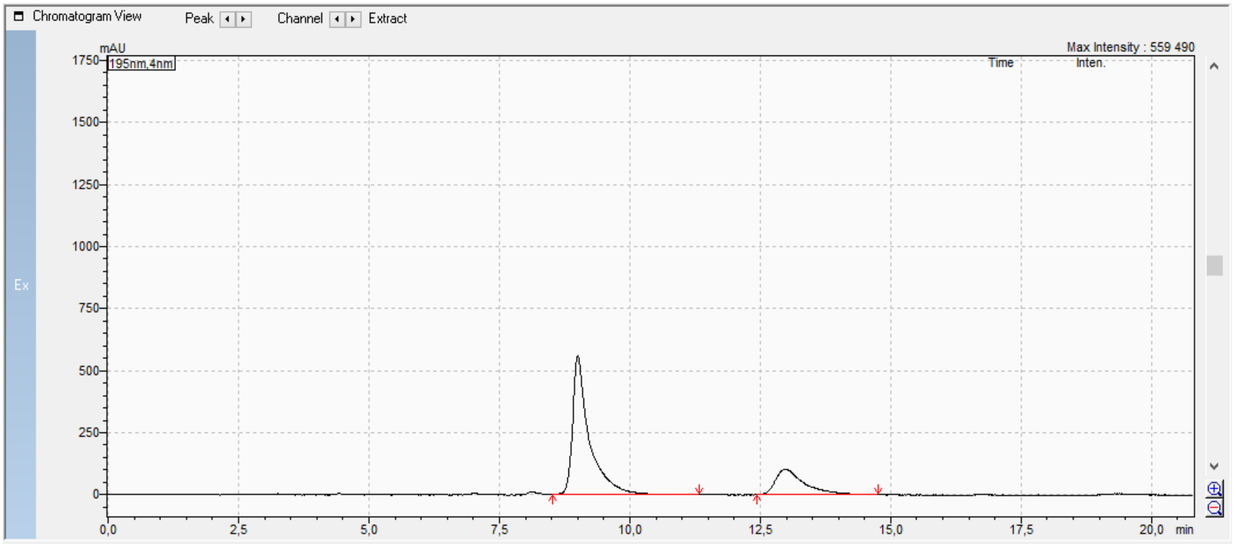

Results View - Peak Table

Peak Table | Compound | Group | Calibration Curve

Peak#	Ret. Time	Area	Height	Peak Start	Peak End	Mark	Conc.	Unit	Area%
1	9,005	12404706	558608	8,523	11,328	M	75,518		75,518
2	12,982	4021422	102672	12,437	14,763	M	24,482		24,482
Total		16426129	661280				100,000		100,000

(S)-4-chloro-2-(4,5-dihydropyrrolo[1,2-a]quinoxalin-4-yl)phenol (3m).

RACEMATE:

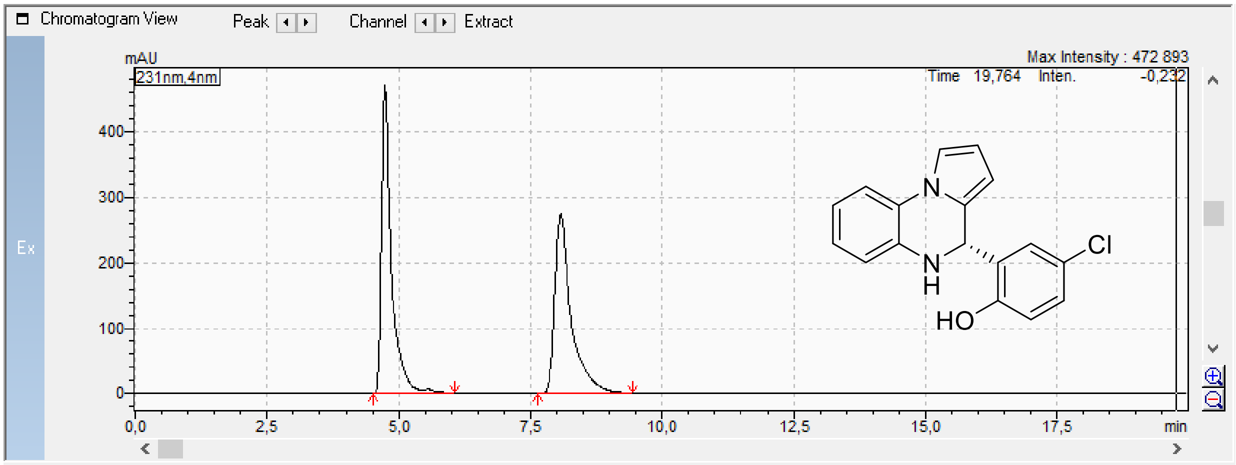

Results View - Peak Table

Peak Table | Compound | Group | Calibration Curve

Peak#	Ret. Time	Area	Height	Peak Start	Peak End	Mark	Conc.	Unit	Area%
1	4,740	6065374	472716	4,512	6,059	M	50,556		50,556
2	8,076	5932065	275134	7,637	9,440	M	49,444		49,444
Total		11997439	747849				100,000		100,000

CHIRAL: *E.r*: 92:8

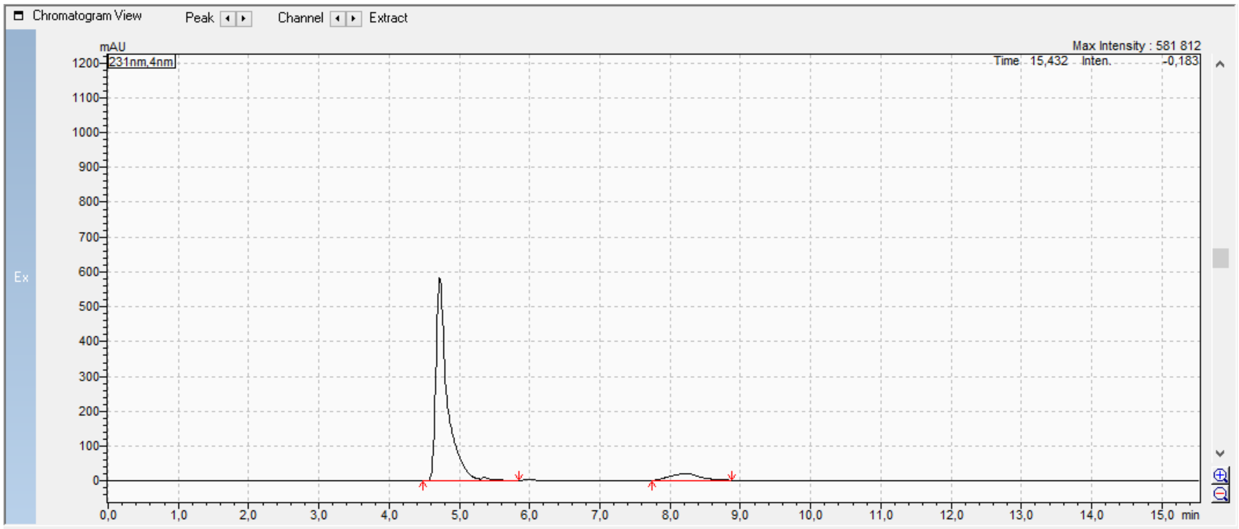

Results View - Peak Table

Peak Table | Compound | Group | Calibration Curve

Peak#	Ret. Time	Area	Height	Peak Start	Peak End	Mark	Conc.	Unit	Area%
1	4,719	6582694	581265	4,480	5,845	M	91,952		91,952
2	8,218	576106	19067	7,744	8,875	M	8,048		8,048
Total		7158800	600332				100,000		100,000

(S)-5-chloro-2-(4,5-dihydropyrrolo[1,2-a]quinoxalin-4-yl)phenol (3n).

RACEMATE:

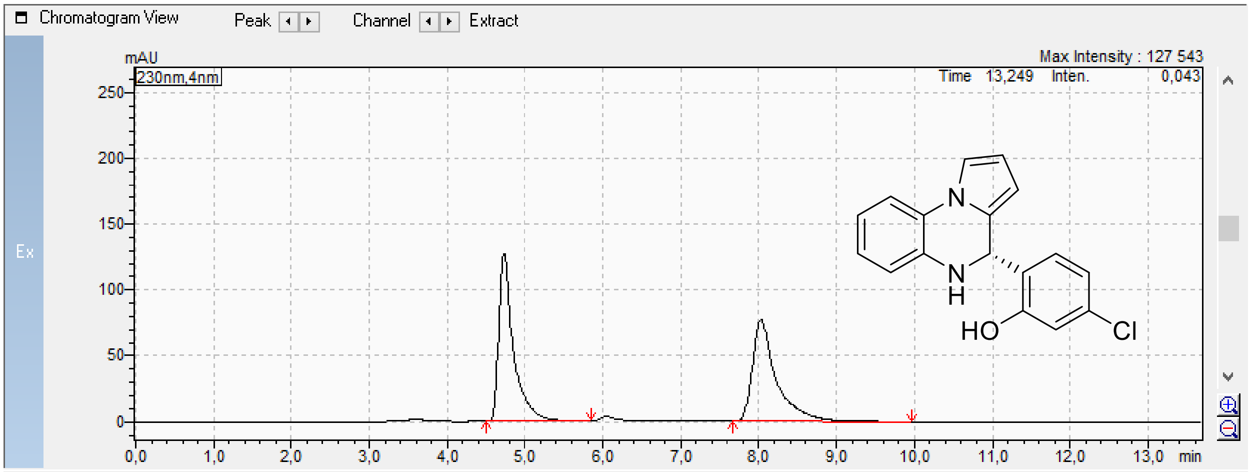

Results View - Peak Table

Peak Table | Compound | Group | Calibration Curve

Peak#	Ret. Time	Area	Height	Peak Start	Peak End	Mark	Conc.	Unit	Area%
1	4,731	1659689	127244	4,501	5,845	M	50,120		50,120
2	8,031	1651723	77578	7,669	9,963	M	49,880		49,880
Total		3311412	204822				100,000		100,000

CHIRAL: *E.r.*: 91:9

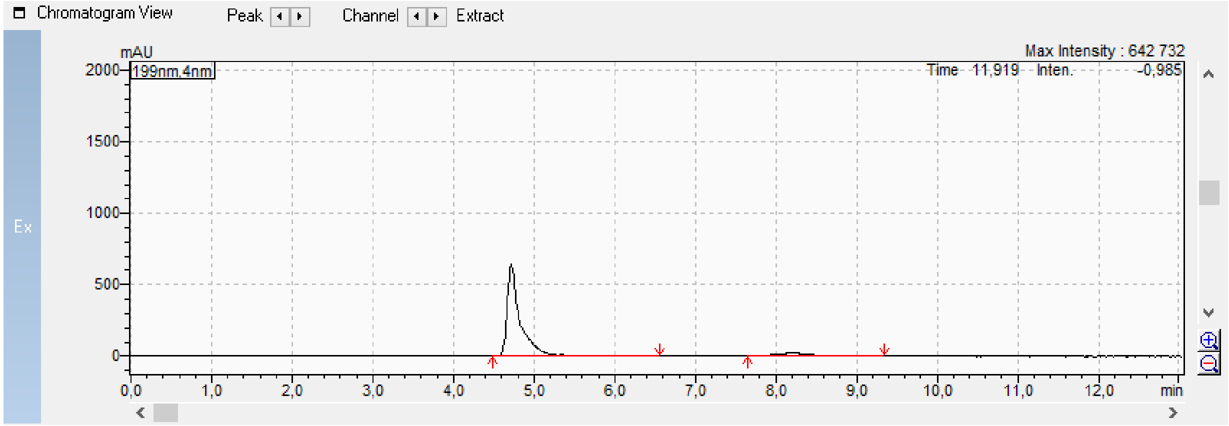

Results View - Peak Table

Peak Table | Compound | Group | Calibration Curve

Peak#	Ret. Time	Area	Height	Peak Start	Peak End	Mark	Conc.	Unit	Area%
1	4,719	7205292	642281	4,491	6,560	M	91,122		91,122
2	8,196	702029	20479	7,648	9,344	M	8,878		8,878
Total		7907321	662760				100,000		100,000

(S)-4-bromo-2-(4,5-dihydropyrrolo[1,2-a]quinoxalin-4-yl)phenol (3o)

RACEMATE:

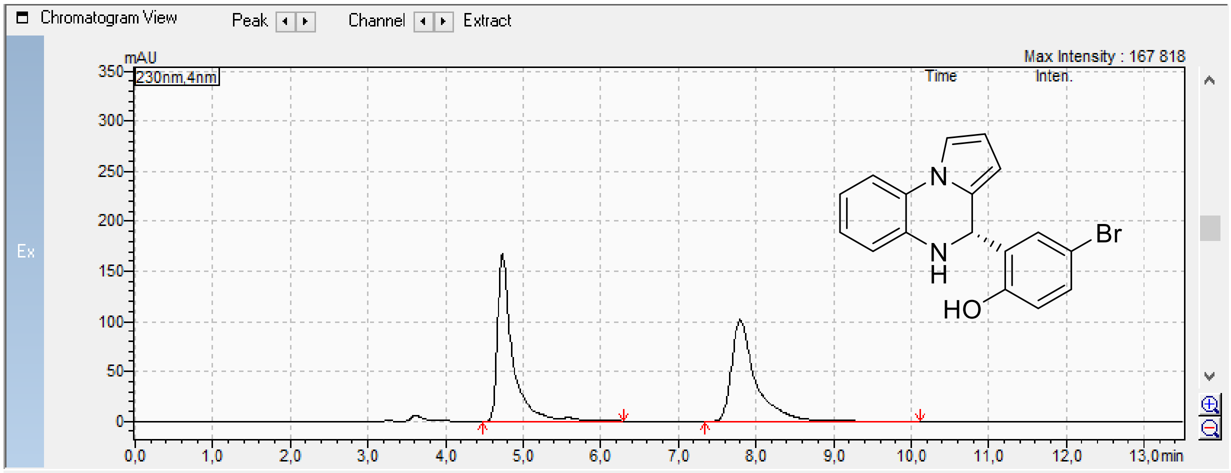

Results View - Peak Table

Peak Table | Compound | Group | Calibration Curve

Peak#	Ret. Time	Area	Height	Peak Start	Peak End	Mark	Conc.	Unit	Area%
1	4,736	2241023	167767	4,480	6,293	M	51,004		51,004
2	7,797	2152794	101386	7,339	10,112	M	48,996		48,996
Total		4393816	269154				100,000		100,000

CHIRAL: *E.r.*: 91:9

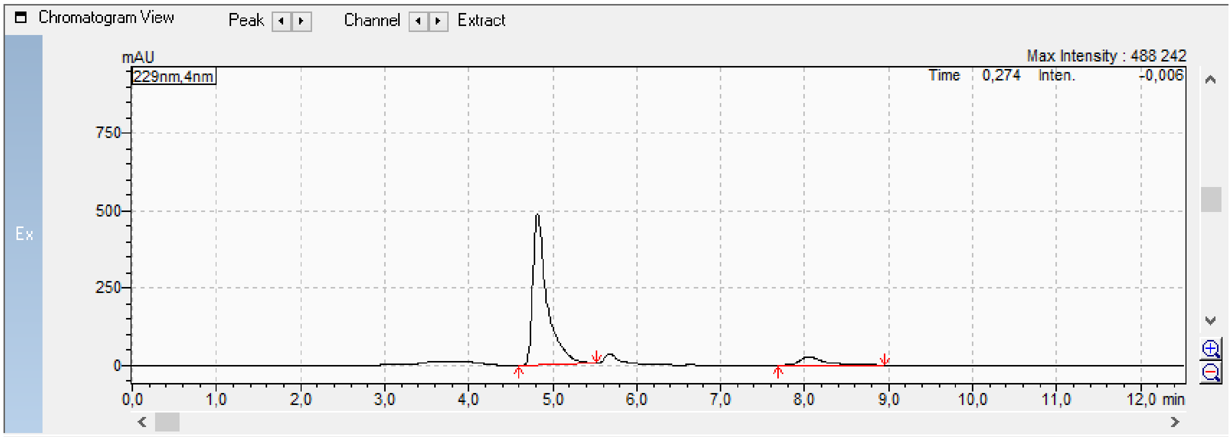

Results View - Peak Table

Peak Table | Compound | Group | Calibration Curve

Peak#	Ret. Time	Area	Height	Peak Start	Peak End	Mark	Conc.	Unit	Area%
1	4,823	5859024	488330	4,597	5,525	M	91,167		91,167
2	8,051	567674	27632	7,680	8,960	M	8,833		8,833
Total		6426698	515961				100,000		100,000

(S)-5-bromo-2-(4,5-dihydropyrrolo[1,2-a]quinoxalin-4-yl)phenol (3p).

RACEMATE:

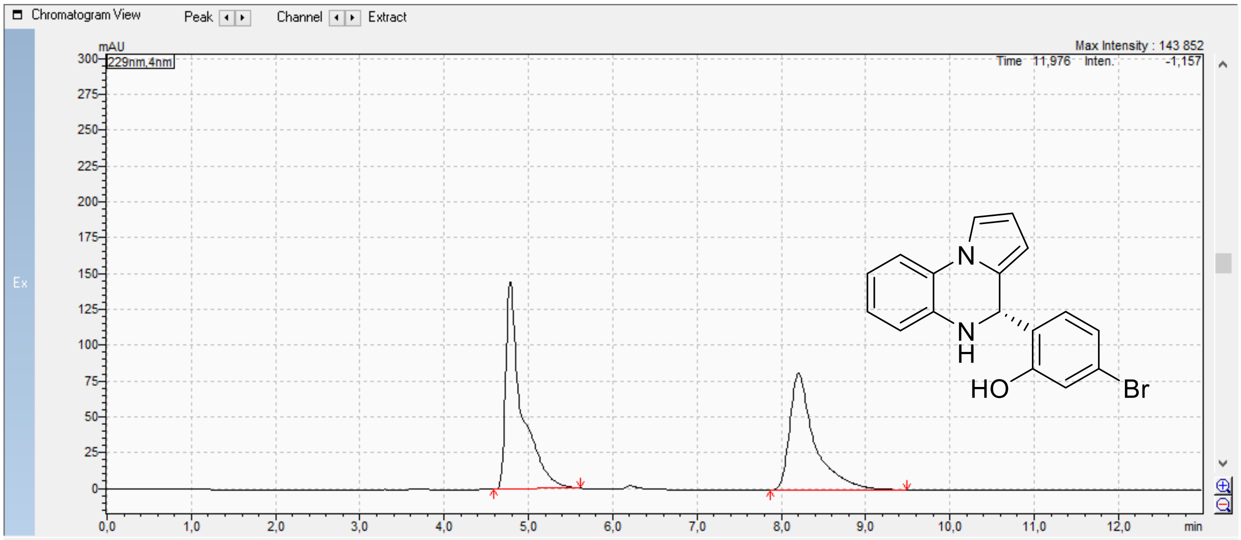

Results View - Peak Table

Peak Table | Compound | Group | Calibration Curve

Peak#	Ret. Time	Area	Height	Peak Start	Peak End	Mark	Conc.	Unit	Area%
1	4,784	1920397	144218	4,587	5,621	M	53,459		53,459
2	8,205	1671851	81452	7,872	9,493	M	46,541		46,541
Total		3592248	225670				100,000		100,000

CHIRAL: *E.r.*: 90:10

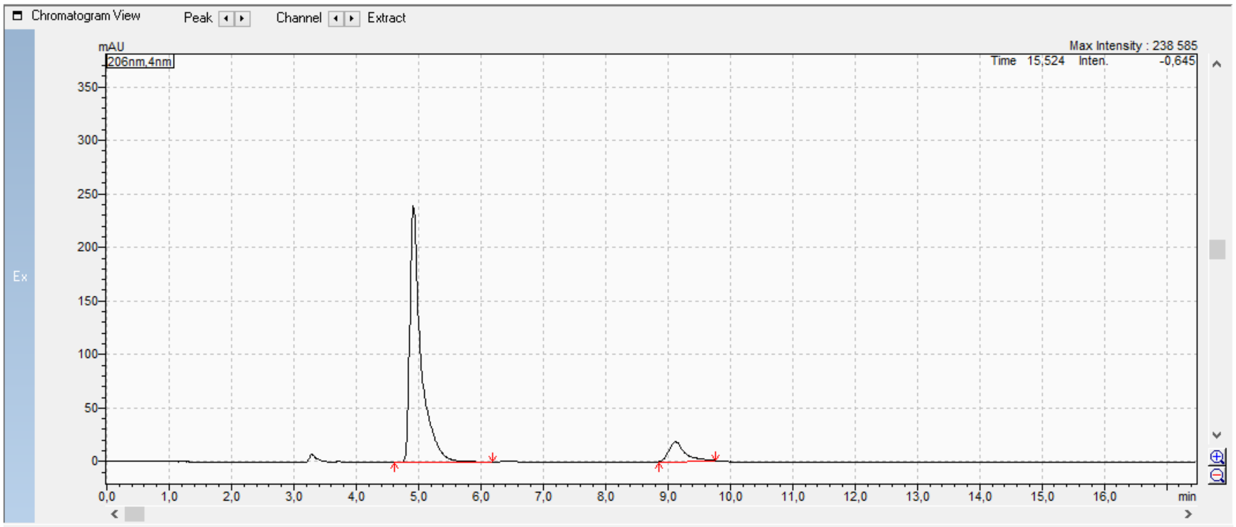

Results View - Peak Table

Peak Table | Compound | Group | Calibration Curve

Peak#	Ret. Time	Area	Height	Peak Start	Peak End	Mark	Conc.	Unit	Area%
1	4,918	2987736	239443	4,619	6,187	M	90,072		90,072
2	9,122	329307	18328	8,853	9,760	M	9,928		9,928
Total		3317042	257772				100,000		100,000

(S)-2-(4,5-dihydropyrrolo[1,2-a]quinoxalin-4-yl)-4-fluorophenol (3q).

RACEMATE:

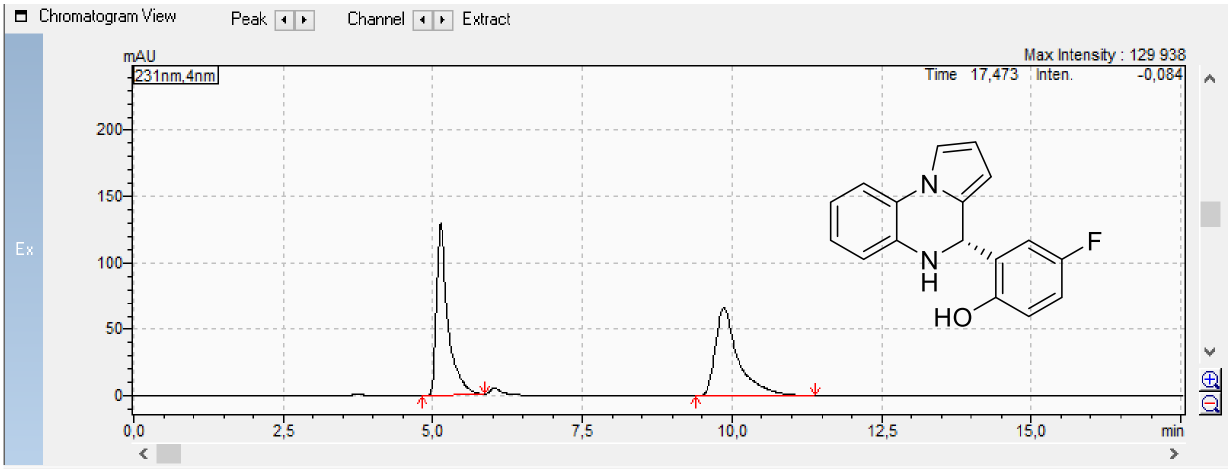

Results View - Peak Table

Peak Table | Compound | Group | Calibration Curve

Peak#	Ret. Time	Area	Height	Peak Start	Peak End	Mark	Conc.	Unit	Area%
1	5,129	1726702	129444	4,821	5,867	M	49,568		49,568
2	9,860	1756820	66645	9,397	11,392	M	50,432		50,432
Total		3483521	196088				100,000		100,000

CHIRAL: *E.r.*: 71:29

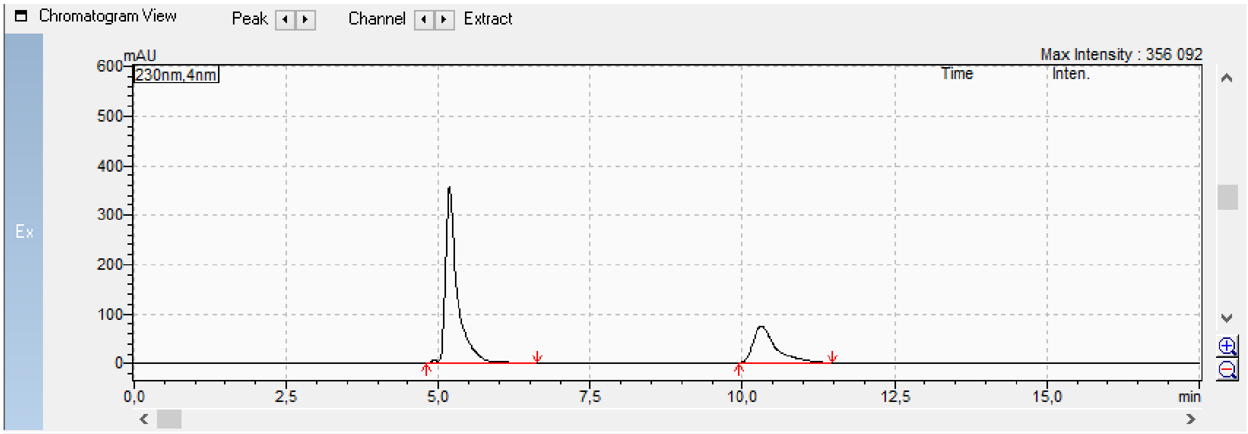

Results View - Peak Table

Peak Table | Compound | Group | Calibration Curve

Peak#	Ret. Time	Area	Height	Peak Start	Peak End	Mark	Conc.	Unit	Area%
1	5,184	4769333	355369	4,800	6,624	M	70,800		70,800
2	10,311	1967049	75253	9,941	11,477	M	29,200		29,200
Total		6736382	430622				100,000		100,000

(S)-2-(4,5-dihydropyrrolo[1,2-a]quinoxalin-4-yl)-5-fluorophenol (3r).

RACEMATE:

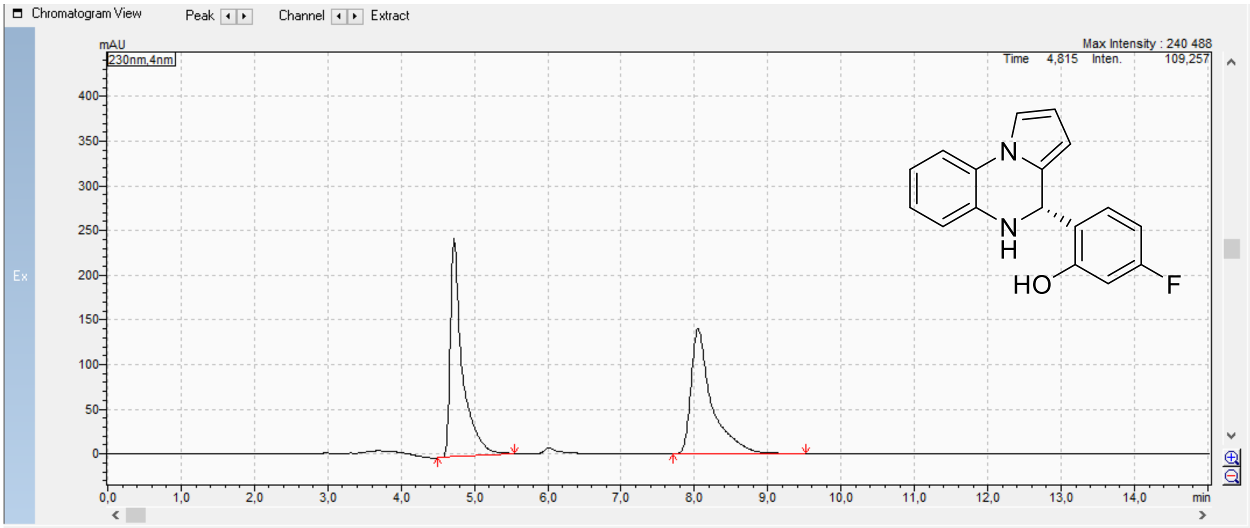

Results View - Peak Table

Peak Table | Compound | Group | Calibration Curve

Peak#	Ret. Time	Area	Height	Peak Start	Peak End	Mark	Conc.	Unit	Area%
1	4,723	2787763	243727	4,501	5,547	M	50,465		50,465
2	8,052	2736385	139801	7,712	9,515	M	49,535		49,535
Total		5524148	383527				100,000		100,000

CHIRAL: *E.r.*: 92:8

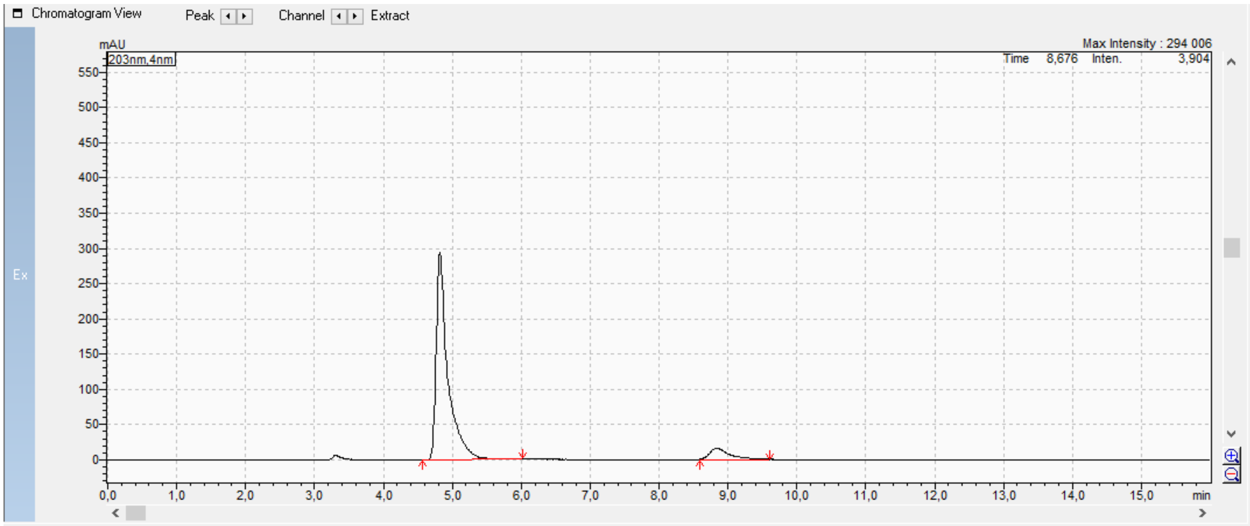

Results View - Peak Table

Peak Table | Compound | Group | Calibration Curve

Peak#	Ret. Time	Area	Height	Peak Start	Peak End	Mark	Conc.	Unit	Area%
1	4,821	3449074	293647	4,565	6,016	M	91,677		91,677
2	8,843	313124	15908	8,587	9,600	M	8,323		8,323
Total		3762198	309556				100,000		100,000

(S)-3-(4,5-dihydropyrrolo[1,2-a]quinoxalin-4-yl)naphthalen-2-ol (3s).

RACEMATE:

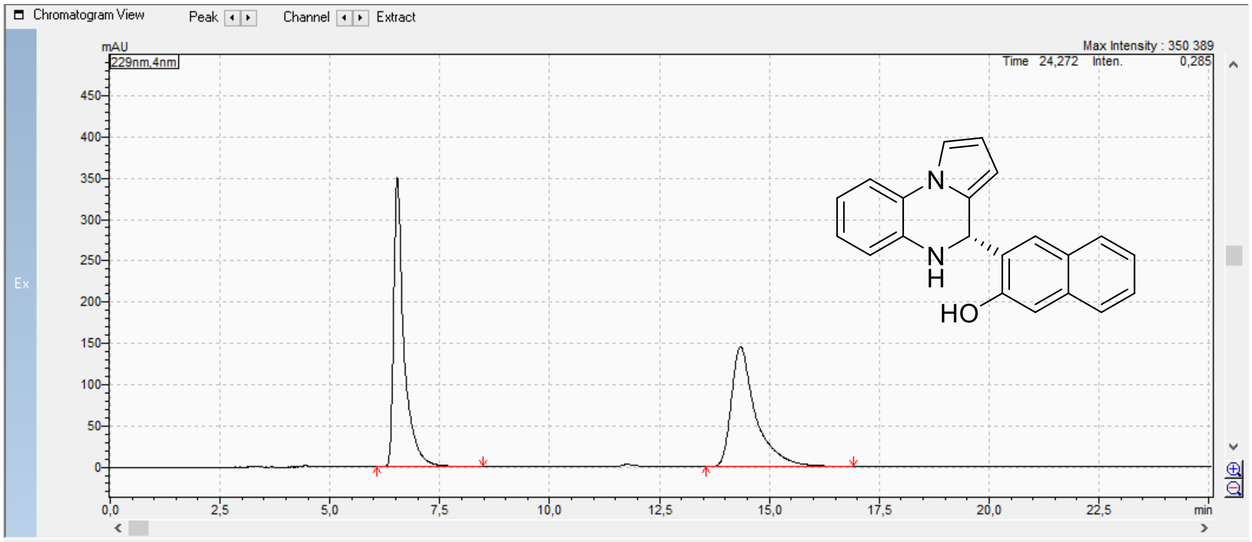

Results View - Peak Table

Peak Table | Compound | Group | Calibration Curve

Peak#	Ret. Time	Area	Height	Peak Start	Peak End	Mark	Conc.	Unit	Area%
1	6,533	5766005	350075	6,059	8,480	M	50,564		50,564
2	14,343	5637450	145309	13,547	16,917	M	49,436		49,436
Total		11403455	495384				100,000		100,000

CHIRAL: *E.r.*: 99:1

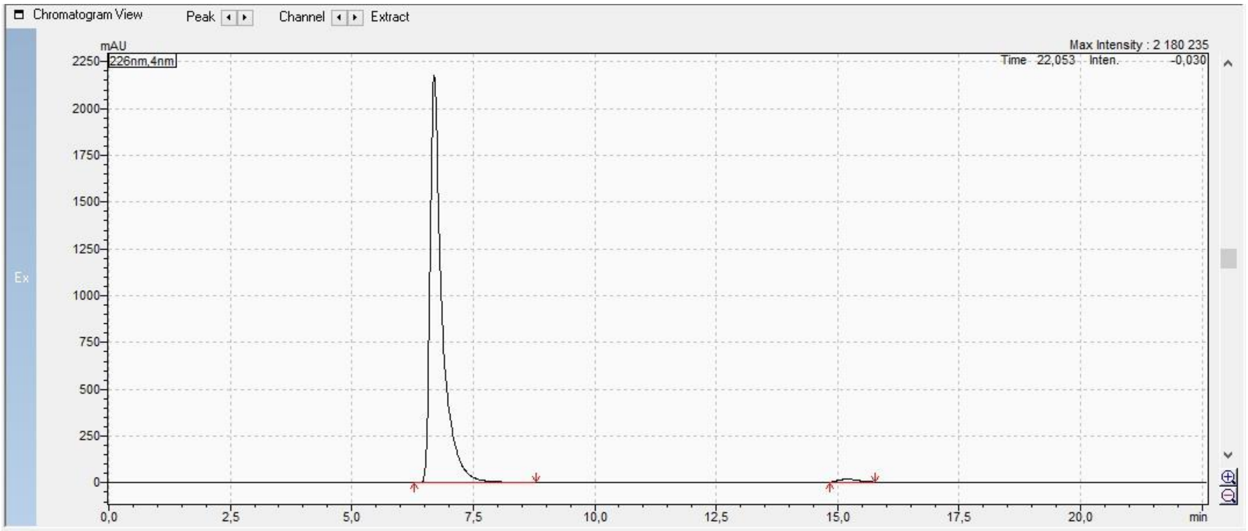

Results View - Peak Table

Peak Table | Compound | Group | Calibration Curve

Peak#	Ret. Time	Area	Height	Peak Start	Peak End	Mark	Conc.	Unit	Area%
1	6,697	37069226	2179238	6,283	8,789	M	98,824		98,824
2	15,198	441071	15654	14,837	15,765	M	1,176		1,176
Total		37510297	2194892				100,000		100,000

(S)-4-(1H-indol-2-yl)-4,5-dihydropyrrolo[1,2-a]quinoxaline (3t).

RACEMATE:

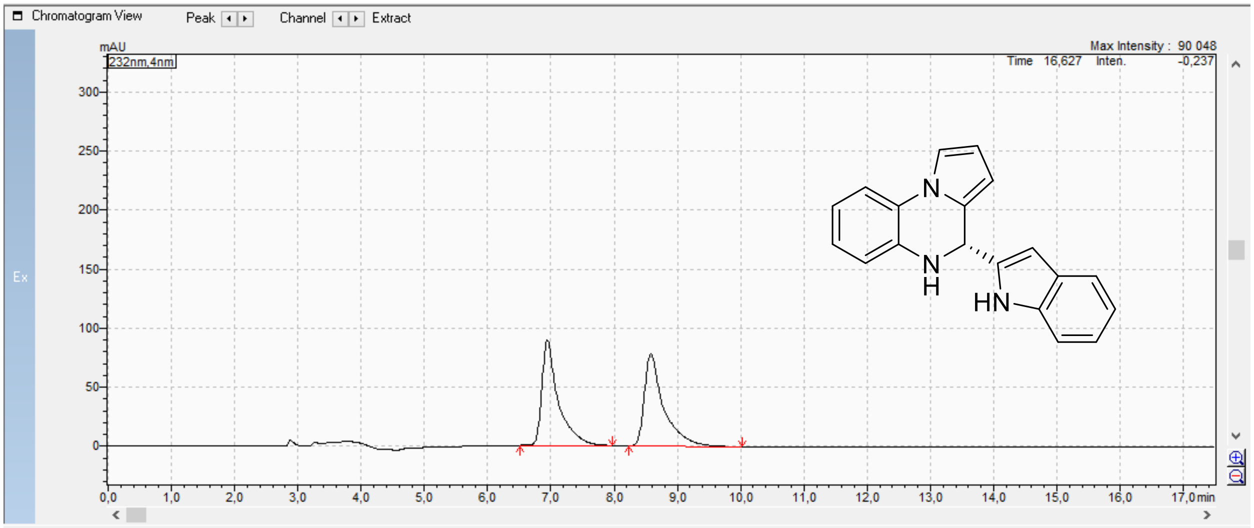

Results View - Peak Table

Peak Table | Compound | Group | Calibration Curve

Peak#	Ret. Time	Area	Height	Peak Start	Peak End	Mark	Conc.	Unit	Area%
1	6,945	1615630	89301	6,507	7,968	M	49,513		49,513
2	8,581	1647439	77959	8,235	10,027	M	50,487		50,487
Total		3263068	167260				100,000		100,000

CHIRAL: *E.r.*: 84:16

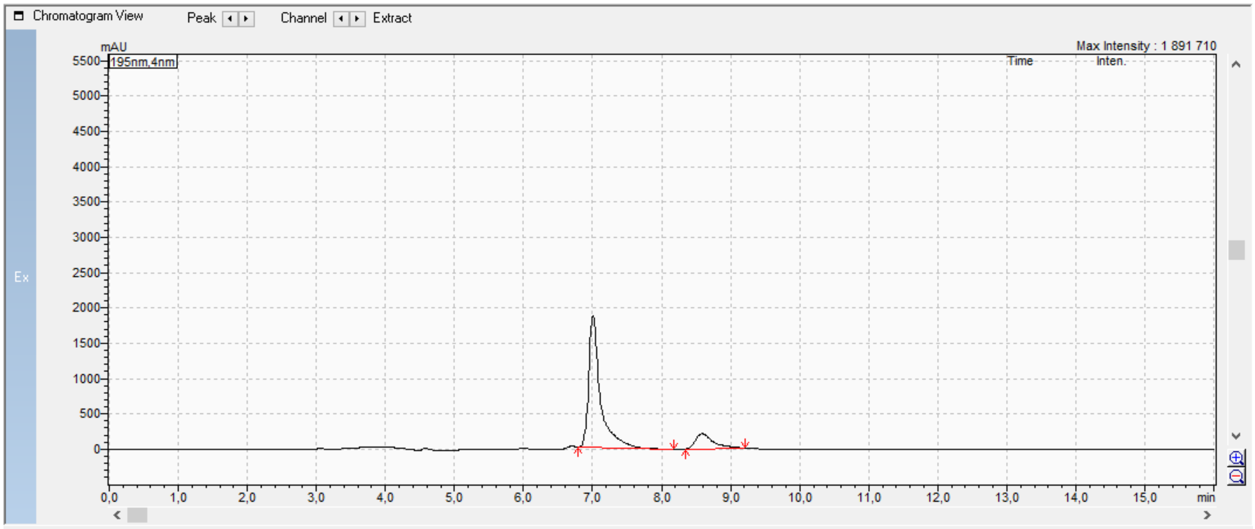

Results View - Peak Table

Peak Table | Compound | Group | Calibration Curve

Peak#	Ret. Time	Area	Height	Peak Start	Peak End	Mark	Conc.	Unit	Area%
1	7,007	21945319	1866679	6,795	8,171	M	85,092		85,092
2	8,588	3844712	215242	8,341	9,216	M	14,908		14,908
Total		25790032	2081921				100,000		100,000

1-benzyl-5'H-spiro[indoline-3,4'-pyrrolo[1,2-a]quinoxalin]-2-one (3u).

RACEMATE:

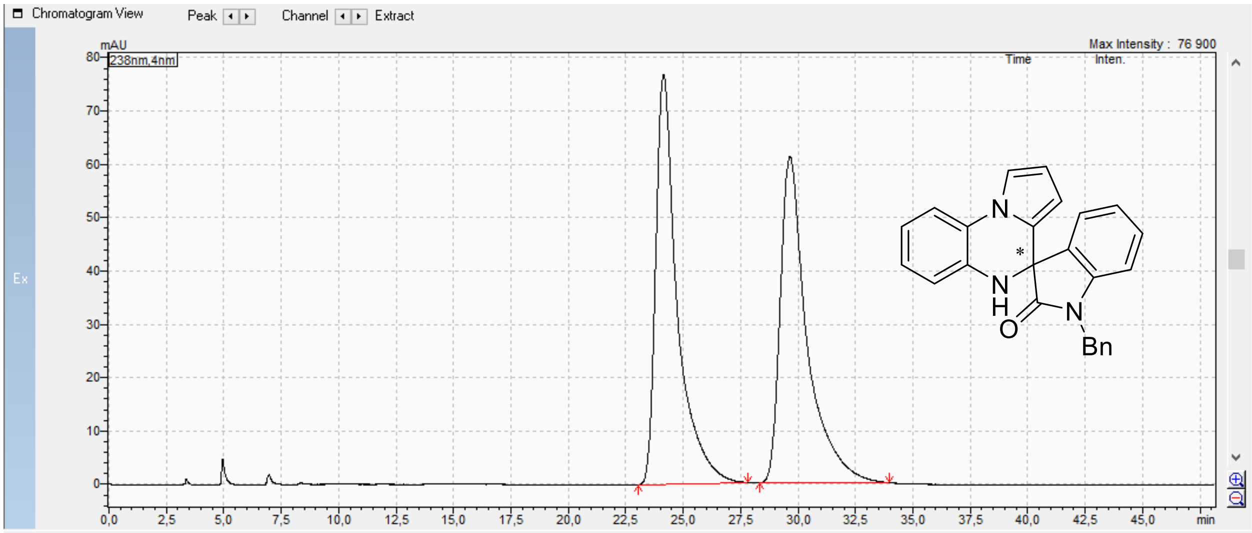

Results View - Peak Table

Peak Table | Compound | Group | Calibration Curve

Peak#	Ret. Time	Area	Height	Peak Start	Peak End	Mark	Conc.	Unit	Area%
1	24,151	5013129	76936	23,019	27,797		50,177		50,177
2	29,653	4977767	61250	28,331	33,995		49,823		49,823
Total		9990895	138185				100,000		100,000

CHIRAL: *E.r.*: 85:15

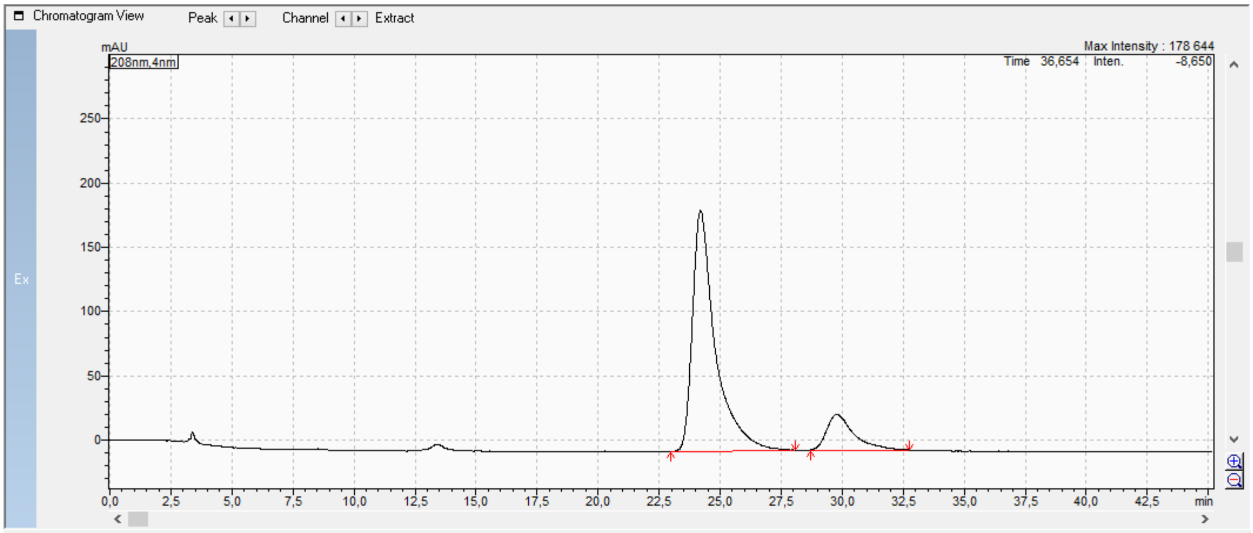

Results View - Peak Table

Peak Table | Compound | Group | Calibration Curve

Peak#	Ret. Time	Area	Height	Peak Start	Peak End	Mark	Conc.	Unit	Area%
1	24,202	12187264	187150	22,976	28,085	M	84,983		84,983
2	29,757	2153531	27786	28,704	32,757	M	15,017		15,017
Total		14340795	214936				100,000		100,000

(S)-2-(9-methyl-4,5-dihydropyrrolo[1,2-a]quinoxalin-4-yl)phenol (3y).

RACEMATE:

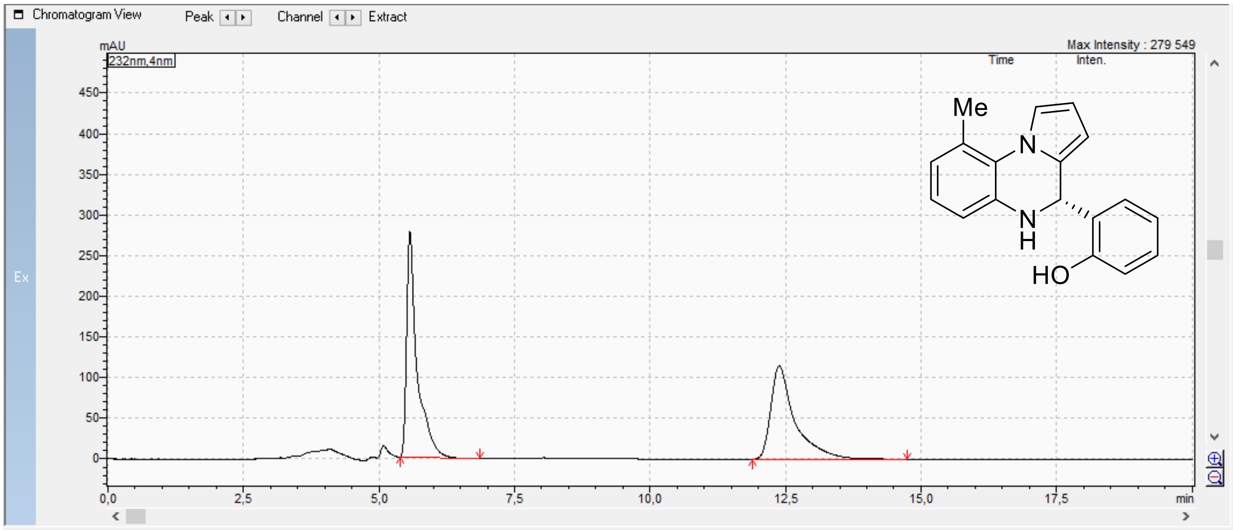

Results View - Peak Table

Peak Table | Compound | Group | Calibration Curve

Peak#	Ret. Time	Area	Height	Peak Start	Peak End	Mark	Conc.	Unit	Area%
1	5,573	3717409	277343	5,387	6,859	M	51,090		51,090
2	12,385	3558718	115004	11,883	14,741	M	48,910		48,910
Total		7276127	392348				100,000		100,000

CHIRAL: *E.r.*: 63:37

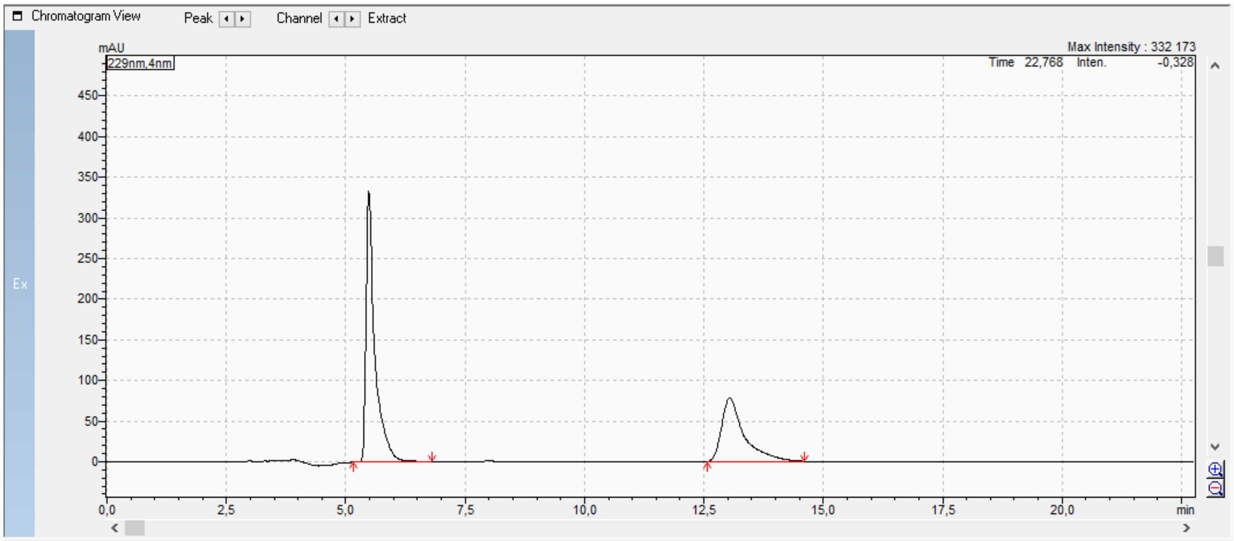

Results View - Peak Table

Peak Table | Compound | Group | Calibration Curve

Peak#	Ret. Time	Area	Height	Peak Start	Peak End	Mark	Conc.	Unit	Area%
1	5,486	4261270	332681	5,163	6,805	M	62,475		62,475
2	13,042	2559533	78462	12,576	14,603	M	37,525		37,525
Total		6820802	411143				100,000		100,000

(S)-2-(9-methoxy-4,5-dihydropyrrolo[1,2-a]quinoxalin-4-yl)phenol (3z).

RACEMATE:

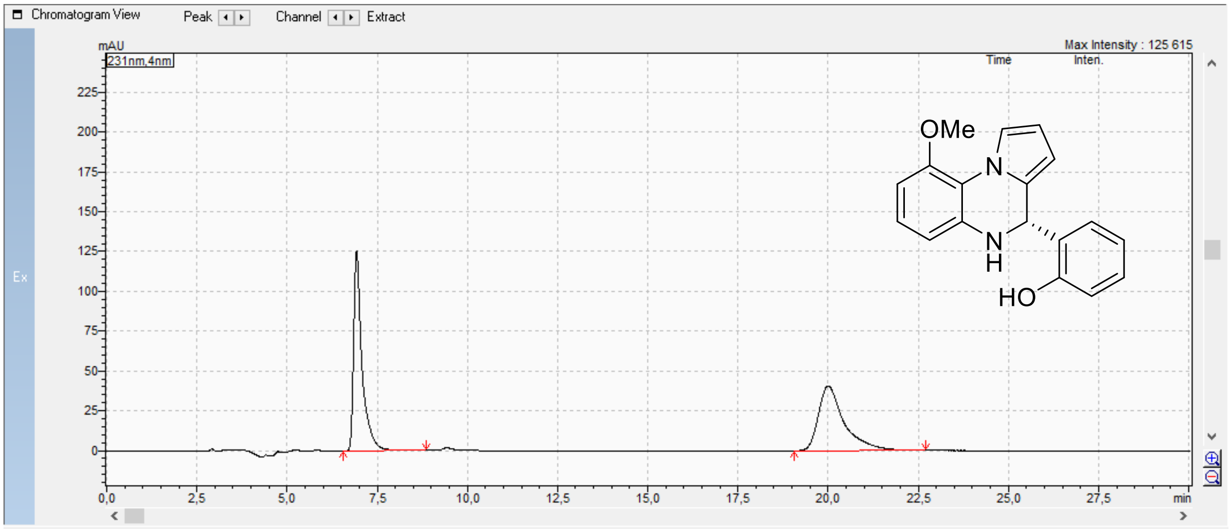

Results View - Peak Table

Peak Table | Compound | Group | Calibration Curve

Peak#	Ret. Time	Area	Height	Peak Start	Peak End	Mark	Conc.	Unit	Area%
1	6,934	2033679	125705	6,560	8,843	M	50,287		50,287
2	20,002	2010498	40706	19,061	22,709	M	49,713		49,713
Total		4044178	166411				100,000		100,000

CHIRAL: *E.r.*: 87:13

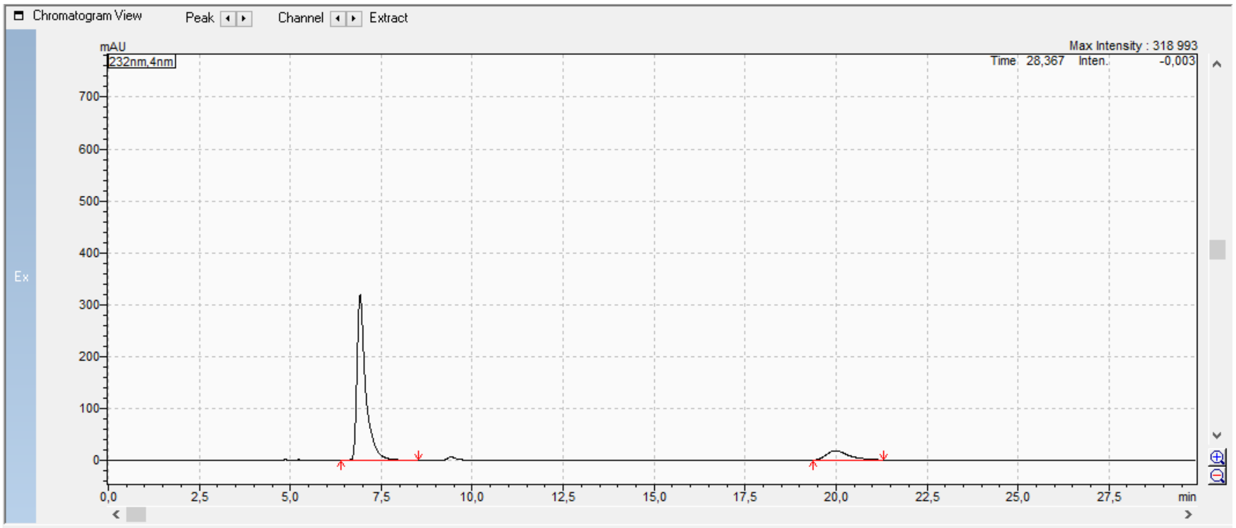

Results View - Peak Table

Peak Table | Compound | Group | Calibration Curve

Peak#	Ret. Time	Area	Height	Peak Start	Peak End	Mark	Conc.	Unit	Area%
1	6,920	5346186	318156	6,389	8,523	M	86,648		86,648
2	19,980	823834	18590	19,371	21,312	M	13,352		13,352
Total		6170020	336746				100,000		100,000

(S)-2-(8-methoxy-4,5-dihydropyrrolo[1,2-a]quinoxalin-4-yl)phenol (3aa).

RACEMATE:

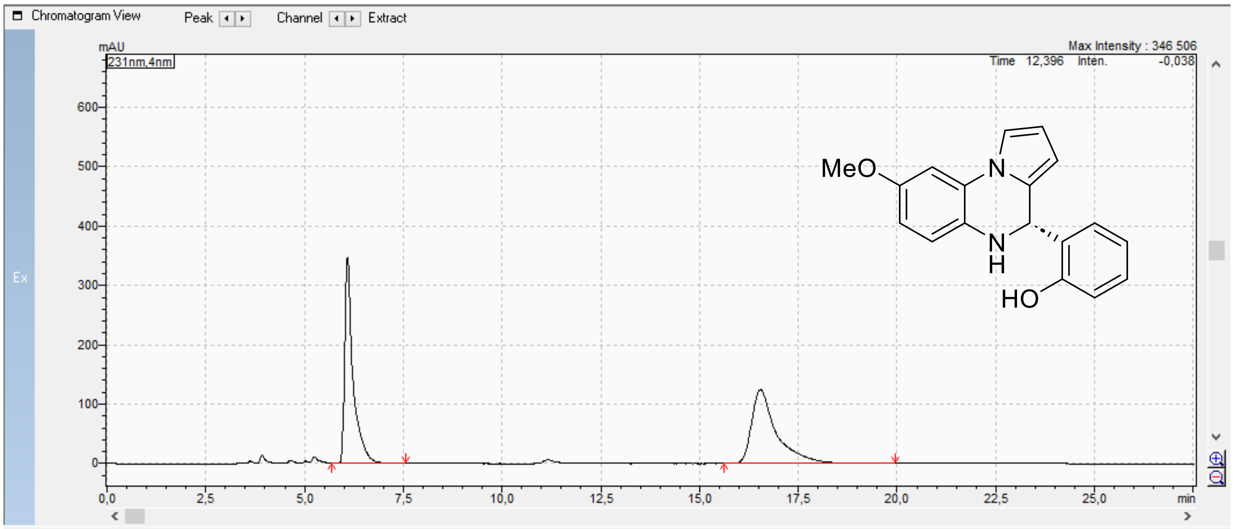

Results View - Peak Table

Peak Table | Compound | Group | Calibration Curve

Peak#	Ret. Time	Area	Height	Peak Start	Peak End	Mark	Conc.	Unit	Area%
1	6,087	5084035	345963	5,685	7,552	M	48,370		48,370
2	16,542	5426732	124523	15,627	19,957	M	51,630		51,630
Total		10510767	470486				100,000		100,000

CHIRAL: *E.r.*: 84:16

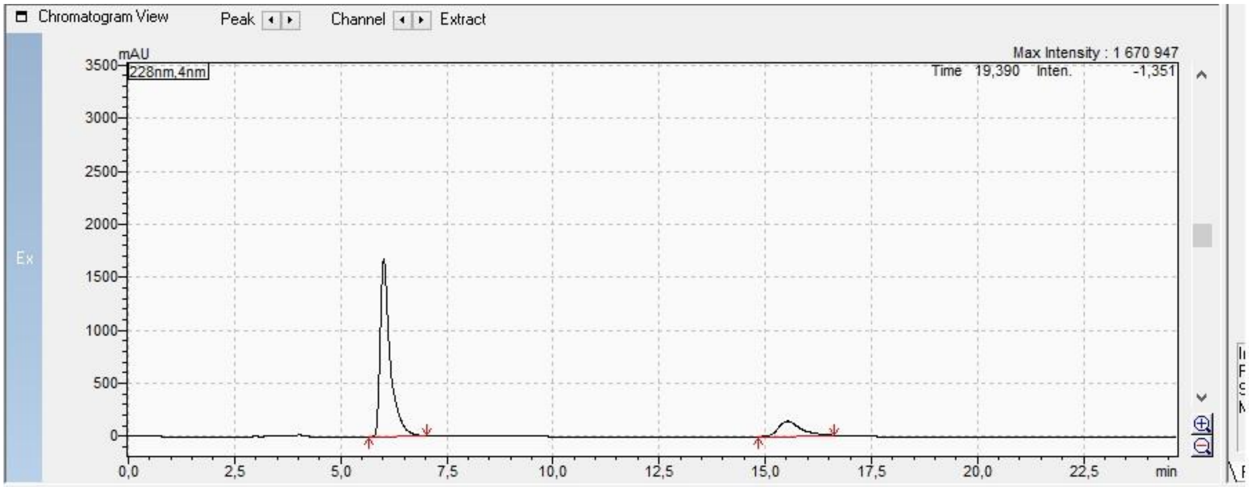

Results View - Peak Table

Peak Table | Compound | Group | Calibration Curve

Peak#	Ret. Time	Area	Height	Peak Start	Peak End	Mark	Conc.	Unit	Area%
1	6,012	27130164	1670048	5,664	7,029	M	83,986		83,986
2	15,528	5172938	139964	14,837	16,629	M	16,014		16,014
Total		32303102	1810013				100,000		100,000

((S)-2-(8-chloro-4,5-dihydropyrrolo[1,2-a]quinoxalin-4-yl)phenol (3bb).

RACEMATE:

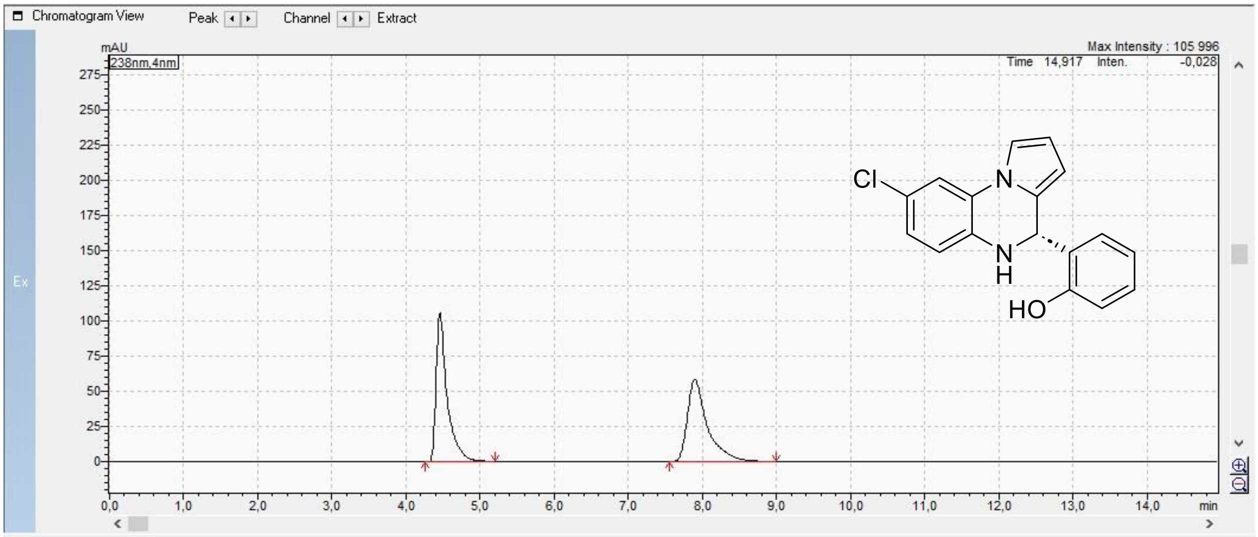

Results View - Peak Table

Peak Table | Compound | Group | Calibration Curve

Peak#	Ret. Time	Area	Height	Peak Start	Peak End	Mark	Conc.	Unit	Area%
1	4,456	1093297	105906	4,256	5,205	M	50,045		50,045
2	7,895	1091317	58506	7,552	8,992	M	49,955		49,955
Total		2184614	164413				100,000		100,000

CHIRAL: *E.r.*: 74:26

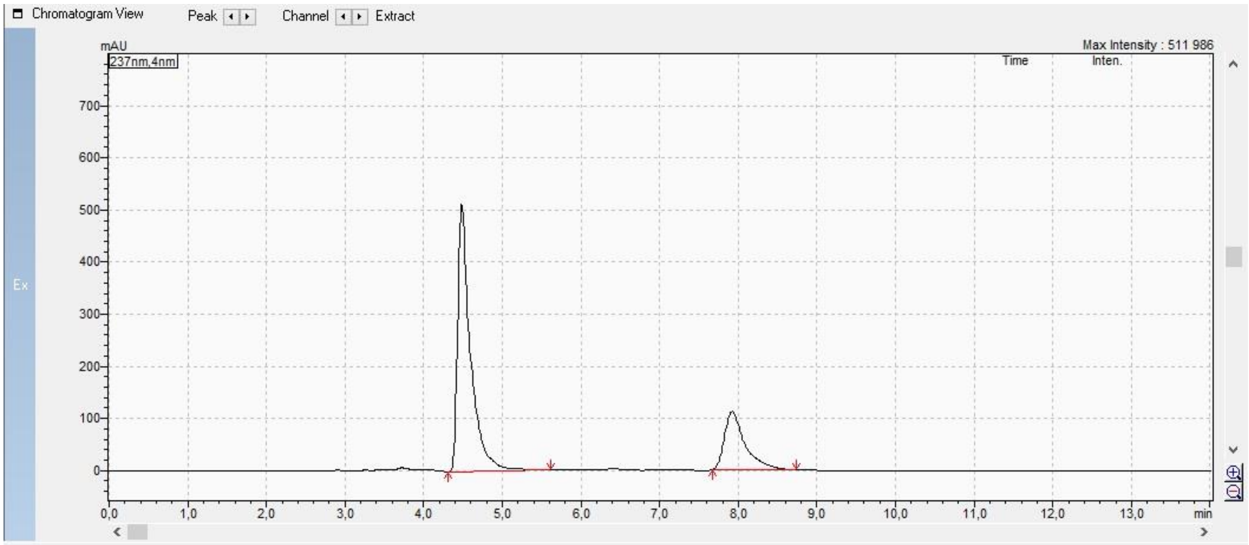

Results View - Peak Table

Peak Table | Compound | Group | Calibration Curve

Peak#	Ret. Time	Area	Height	Peak Start	Peak End	Mark	Conc.	Unit	Area%
1	4,483	5814503	513649	4,309	5,621	M	74,105		74,105
2	7,922	2031761	111567	7,669	8,736	M	25,895		25,895
Total		7846264	625216				100,000		100,000

(S)-2-(7-bromo-4,5-dihydropyrrolo[1,2-a]quinoxalin-4-yl)phenol (3cc).

RACEMATE:

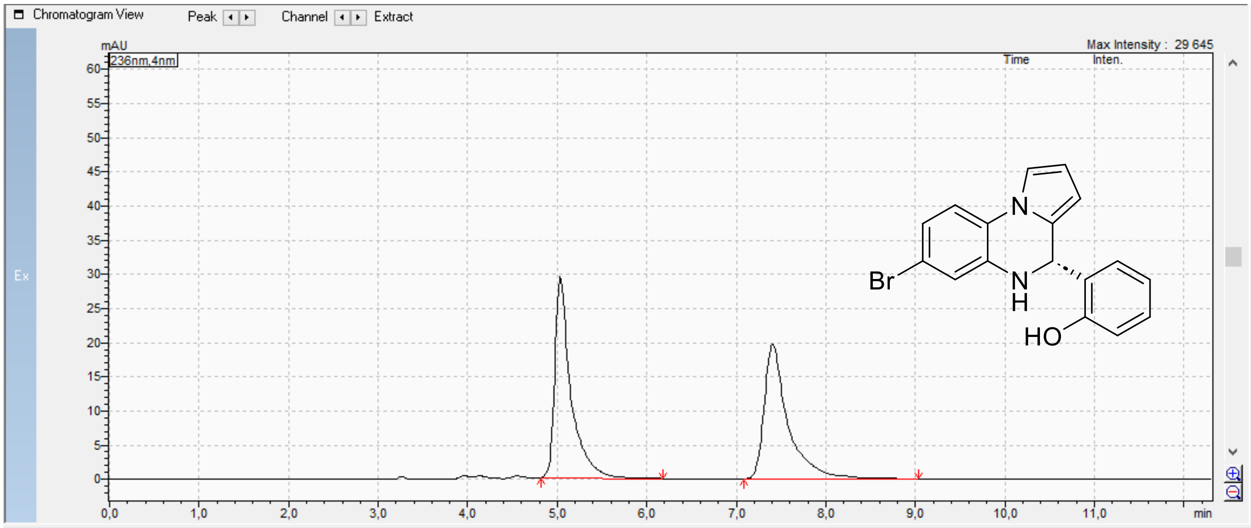

Results View - Peak Table

Peak Table | Compound | Group | Calibration Curve

Peak#	Ret. Time	Area	Height	Peak Start	Peak End	Mark	Conc.	Unit	Area%
1	5,035	365620	29418	4,821	6,176	M	50,297		50,297
2	7,407	361305	19669	7,083	9,035	M	49,703		49,703
Total		726925	49087				100,000		100,000

CHIRAL: *E.r.*: 64:36

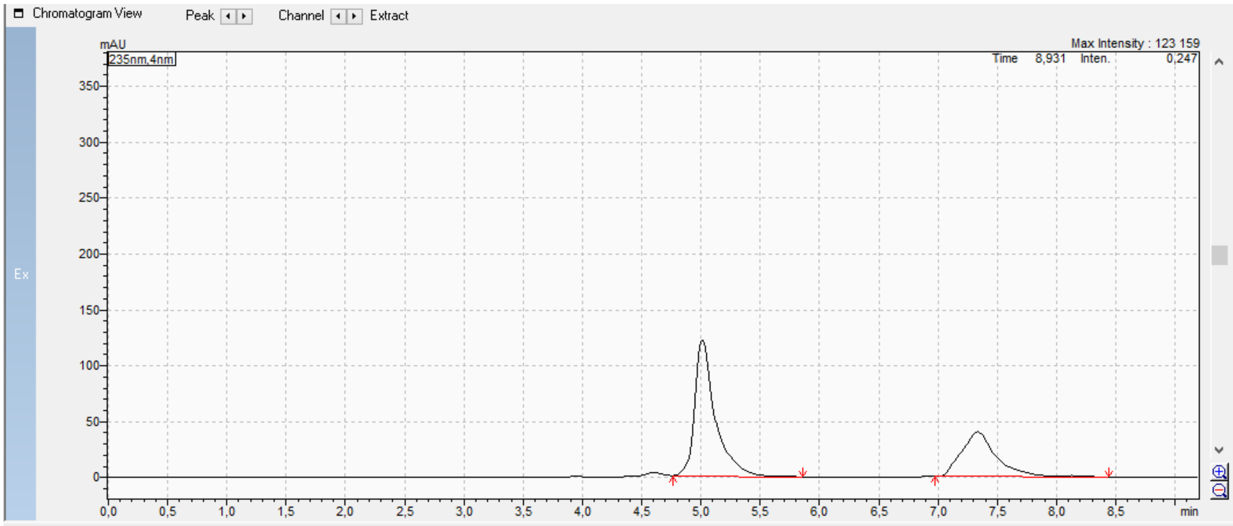

Results View - Peak Table

Peak Table | Compound | Group | Calibration Curve

Peak#	Ret. Time	Area	Height	Peak Start	Peak End	Mark	Conc.	Unit	Area%
1	5,012	1460109	121576	4,768	5,856	M	64,045		64,045
2	7,335	819696	40055	6,976	8,437	M	35,955		35,955
Total		2279805	161631				100,000		100,000

(S)-2-(7-(trifluoromethyl)-4,5-dihydropyrrolo[1,2-a]quinoxalin-4-yl)phenol (3dd).

RACEMATE:

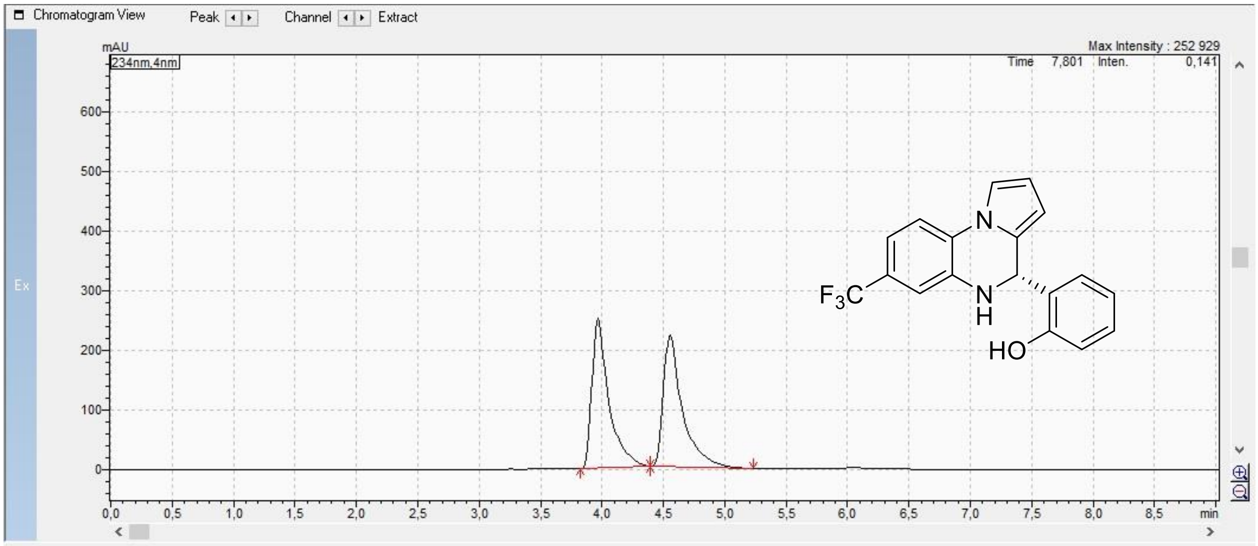

Results View - Peak Table

Peak Table | Compound | Group | Calibration Curve

Peak#	Ret. Time	Area	Height	Peak Start	Peak End	Mark	Conc.	Unit	Area%
1	3,967	2401107	251061	3,819	4,395	M	49,893		49,893
2	4,555	2411360	221746	4,395	5,237	M	50,107		50,107
Total		4812467	472807				100,000		100,000

CHIRAL: *E.r.*: 63:37

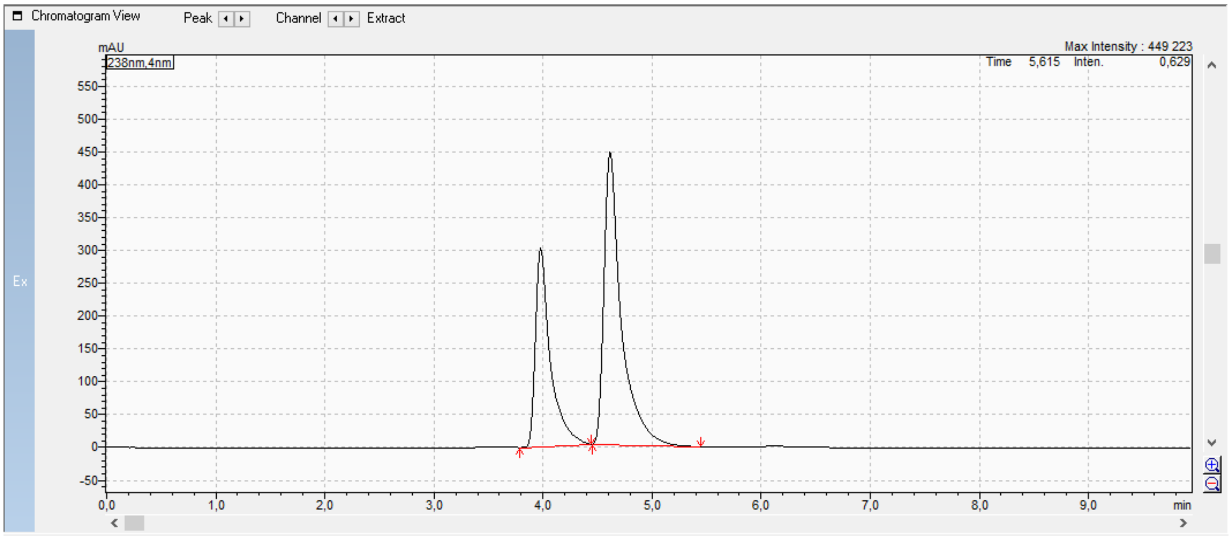

Results View - Peak Table

Peak Table | Compound | Group | Calibration Curve

Peak#	Ret. Time	Area	Height	Peak Start	Peak End	Mark	Conc.	Unit	Area%
1	3,982	2888051	302766	3,787	4,437	M	36,829		36,829
2	4,612	4953830	444959	4,448	5,451	M	63,171		63,171
Total		7841881	747726				100,000		100,000

(S)-2-(5-acetyl-4,5-dihydropyrrolo[1,2-a]quinoxalin-4-yl)phenyl acetate (4).

RACEMATE:

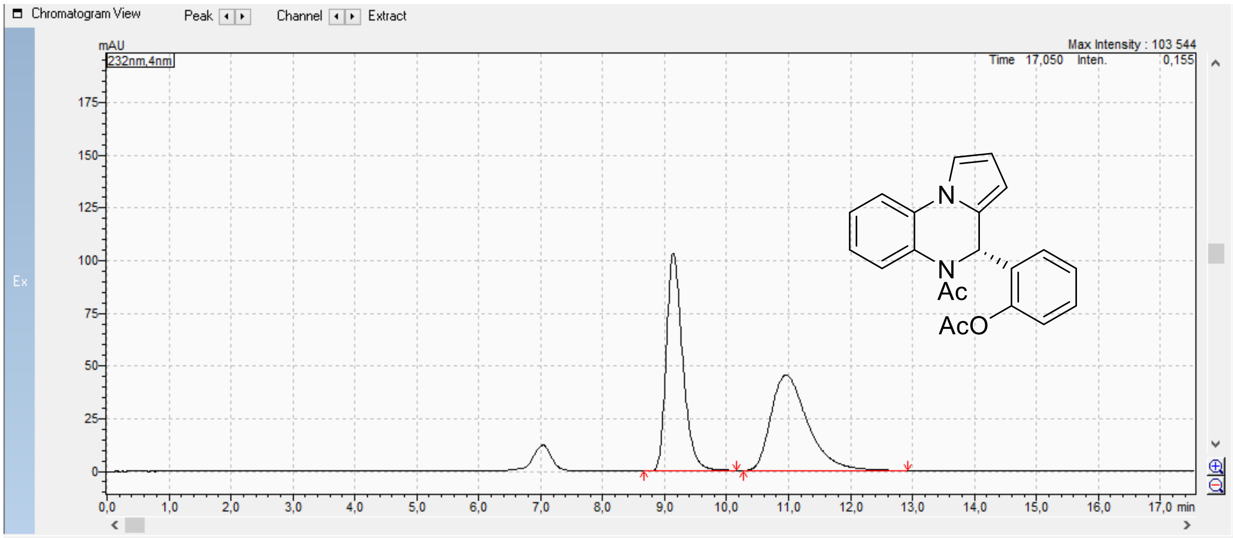

Results View - Peak Table

Peak Table | Compound | Group | Calibration Curve

Peak#	Ret. Time	Area	Height	Peak Start	Peak End	Mark	Conc.	Unit	Area%
1	9,139	1902320	103349	8,661	10,155	M	50,415		50,415
2	10,958	1871005	45364	10,272	12,928	M	49,585		49,585
Total		3773326	148713				100,000		100,000

CHIRAL: *E.r.*: 99:1

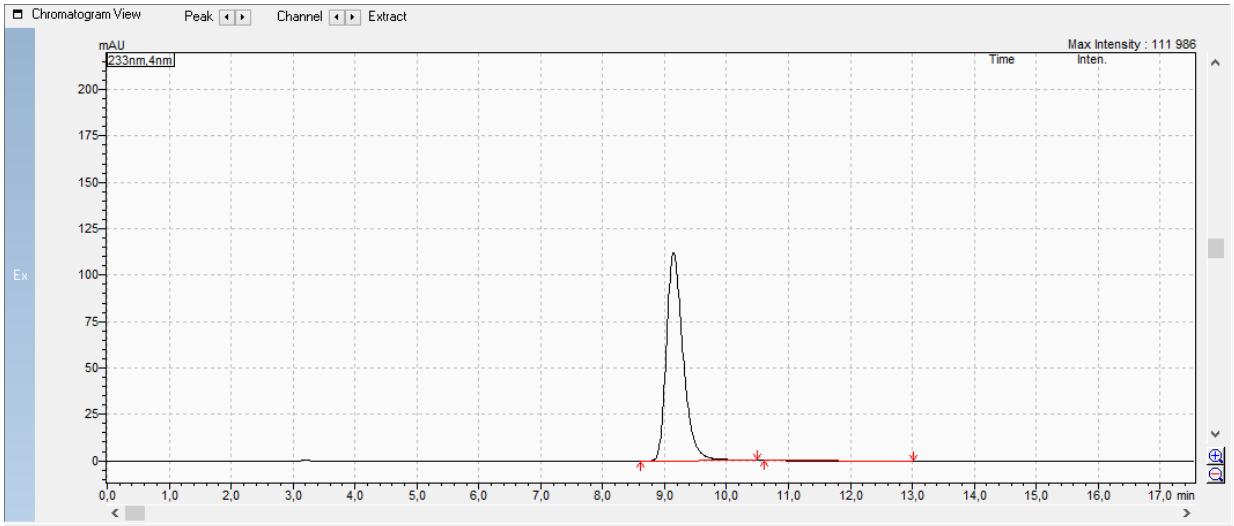

Results View - Peak Table

Peak Table | Compound | Group | Calibration Curve

Peak#	Ret. Time	Area	Height	Peak Start	Peak End	Mark	Conc.	Unit	Area%
1	9,145	2105749	111984	8,608	10,496	M	100,322		100,322
2	10,630	-6766	-3	10,613	13,013	M	-0,322		-0,322
Total		2098984	111981				100,000		100,000

(S)-2-(5-acetyl-1-bromo-4,5-dihydropyrrolo[1,2-a]quinoxalin-4-yl)phenyl acetate (5).

RACEMATE:

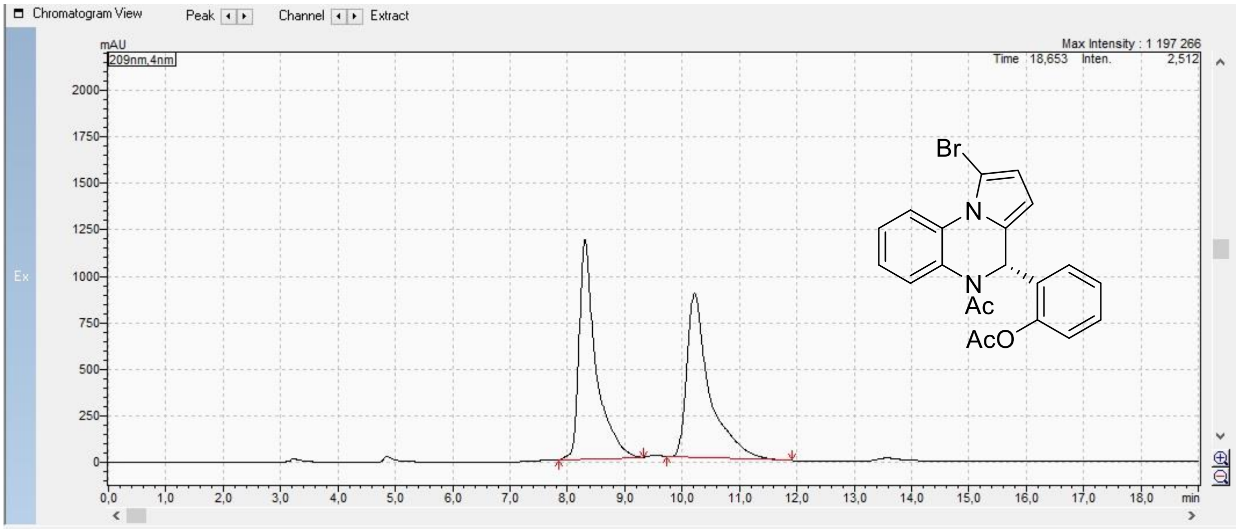

Results View - Peak Table

Peak Table | Compound | Group | Calibration Curve

Peak#	Ret. Time	Area	Height	Peak Start	Peak End	Mark	Conc.	Unit	Area%
1	8,306	24199248	1182154	7,840	9,333	M	49,903		49,903
2	10,217	24293024	884212	9,728	11,915	M	50,097		50,097
Total		48492272	2066366				100,000		100,000

CHIRAL: *E.r.*: 95:5

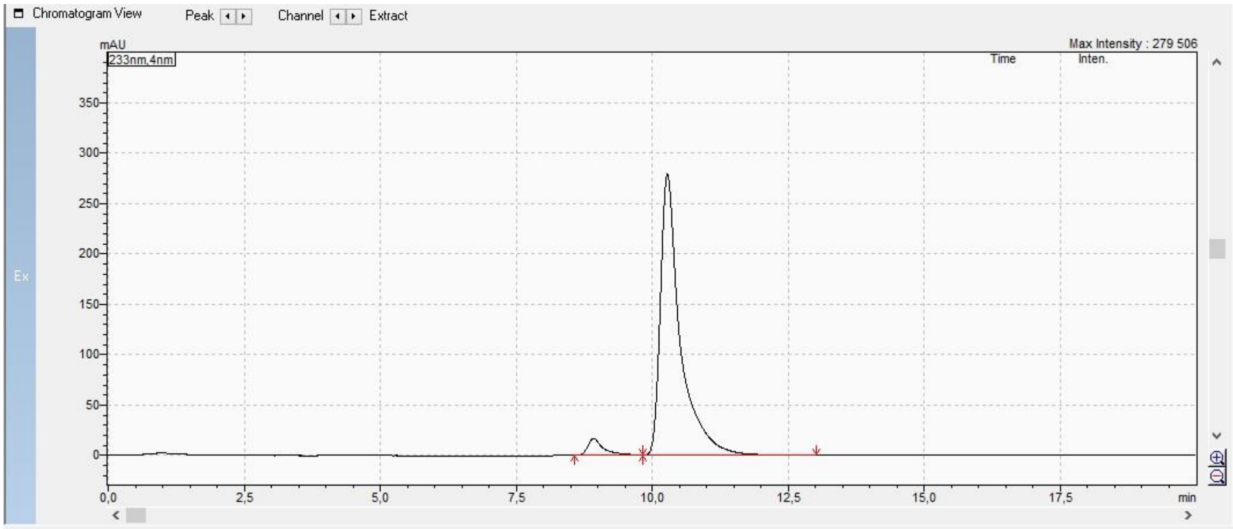

Results View - Peak Table

Peak Table | Compound | Group | Calibration Curve

Peak#	Ret. Time	Area	Height	Peak Start	Peak End	Mark	Conc.	Unit	Area%
1	8,924	338361	16866	8,565	9,835	M	4,552		4,552
2	10,283	7095511	279071	9,835	13,013	M	95,448		95,448
Total		7433872	295938				100,000		100,000
